# Turnover Rate of Lipids, Metabolites and Proteins Revealed by 156-Day-Long D_2_O Administration in a Guinea Pig

**DOI:** 10.3390/ijms27041944

**Published:** 2026-02-18

**Authors:** Yury Kostyukevich, Anastasia Malyukova, Nikita Malyshev, Anna Vishnevskaya, Anna Levashova, Anna Kovalenko, Albert Kireev, Azat Meshcherov, Liudmila Borisova, Boris Tupertsev, Anton Bashilov, Sergey Osipenko

**Affiliations:** 1 The Center for Bio- and Medical Technologies Nobela 3, 121205 Moscow, Russia; anastasija.maluckowa@yandex.ru (A.M.); nv.malyshev@yandex.ru (N.M.); ai.vish@yandex.ru (A.V.); annalevashova3@gmail.com (A.L.); kovalenko.a.ed@muctr.ru (A.K.); albertkireev@mail.ru (A.K.); strosaz125@gmail.com (A.M.); btoupersev@gmail.com (B.T.); anton_bashilov@mail.ru (A.B.); ossipenko9191@gmail.com (S.O.); 2 Russian Presidential Academy of National Economy and Public Administration, 82, Vernadsky Ave., 119571 Moscow, Russia; borisova-la@ranepa.ru

**Keywords:** D_2_O, metabolism, lipids, LC-MS/MS

## Abstract

Measurement of the turnover rate of proteins, different metabolites and lipids in living organisms is important for the understanding of biochemical pathways and physiology studies. Such experiments can be performed by administering isotopically labeled substances (food or water) to the organism and measuring the amount of the isotopes in the endogenous compounds. Here, we administered 20% heavy water (D_2_O) to a guinea pig for 156 days and regularly measured the deuterium uptake in C-H groups in the different compounds of blood, urine and feces using high-resolution mass spectrometry. We successfully measured the time required for reaching the maximum deuteration level for several classes of compounds: 10 days for blood lipids (PC, PE, TAG); 60 days for sterol derivatives, heme B and hemoglobin; and 70 days for stercobilin. Also, for those compounds, we measured the deuterium elimination time from the organism when deuterium administration was stopped. The turnover of lipids was also studied by administering deuterated oat leaves grown at 10% D_2_O to the guinea pig. The analysis of blood revealed that triglycerides demonstrate the inclusion of the deuterium after 5 h. All experiments were performed on a single guinea pig that remained alive and in good health after all experiments. The current research demonstrates the possibility of using long-term D_2_O administration for the investigation of metabolism.

## 1. Introduction

The use of isotopes for the investigation of biochemical pathways began in the thirties of the 20th century. Calvin [1], Krebs [2], Schoenheimer [3,4,5,6], Ussing [7,8] and many others [9] used stable and radioactive isotopic labels (carbon, nitrogen, oxygen, sulfur, phosphorus, etc.) for the investigation of the transformation of biochemical compounds in living organisms. Isotopic labels can be administered in different forms, including water, amino acids, acetate, gases, isotopically labeled food, etc. Tracing the inclusion of different compounds can be performed using various chemical methods, such as radiography (for radioactive isotopes), mass spectrometry, NMR, etc.

The important application of isotopic labeling is the investigation of the turnover time of biological compounds, especially lipids and proteins. Usually, such experiments are performed using radioactive isotopes. For example, M.E. Smith reported the use of [^14^C]glucose to measure the turnover rate of many lipids and proteins in the myelin of adult rats [10]. Jungalwala and Dawson investigated the turnover rate of phospholipids in the brain using [^14^C]glycerol [11].

The application of stable isotopes (^13^C, ^15^N, etc.) for the investigation of the turnover rate was also widely researched [12,13,14,15]. Such investigations were considerably powered by the simultaneous development of mass spectrometric techniques, making it possible to obtain the molecular weight and isotopic distribution for a wide range of biological compounds. Hellerstein and Neese developed a mathematical model for the analysis of the mass spectrum changes after stable isotope administration to living organisms and used it for the estimation of the endogenous synthesis of lipids and glucose [16]. Approaches to the measurement of the turnover rate of proteins were summarized by Doherty and Beynon [17]. In 2004, the Stable Isotope Labeling in Mammals (SILAM) approach was introduced and developed by J. Yates’s laboratory [18]. This approach aims to raise a fully isotopically labeled animal by feeding it an isotope-rich diet. Currently, commercially available foods exist that are based on ^15^N spirulina or ^13^C labeled amino acids (lysine or leucine). Price et al., using ^15^N-labeled spirulina, determined the turnover rate of proteins in mouse brains [19]. Recently, Rolfs et al. presented an atlas of protein turnover rates in mouse tissues created by measuring the ^13^C lysine incorporation in proteins [20]. Using this technique, Harasimov et al. discovered the unusual stability of proteins in oocytes and the ovaries [21].

Deuterium oxide, probably the most available and cheap source of isotopes, was also widely used for the measurement of the turnover rate. Deuterium, along with hydrogen, participates in many biological reactions and is included in C-H groups of biological compounds. The inclusion of deuterium in the -OH, -SH, or NH groups is not useful for turnover rate studies because of the extremely fast exchange (and back exchange) time.

Allister et al. investigated triglyceride storage in the adipose tissue of insulin-resistant humans. Subjects consumed D_2_O for 4 weeks to achieve and maintain a total body D_2_O water enrichment of 2%. The authors investigated the biosynthesis rate of fatty acids for insulin-resistant and insulin-sensitive subjects [22]. Castro-Perez et al. used D_2_O administration to measure the changes in cholesterol synthesis for high-carbohydrate and high-fat diets [23]. Ando et al. investigated the turnover rate of myelin lipids in the aging brain and found that the rate of cholesterol incorporation decreases with age [24]. With the recent development in ultrahigh-resolution mass spectrometry, it has become routinely possible to measure the deuterium uptake even for low concentrations (~1%) and distinguish it from the natural ^13^C isotope, as demonstrated by Fu et al. [25].

In our laboratory, we are working on the analytical and bioanalytical applications of isotopic labels combined with high-resolution mass spectrometry using mostly heavy water (D_2_O and H_2_^18^O). We have developed the analytical platform for increasing the reliability of compound identification using isotope exchange [26,27,28,29,30,31,32,33,34] in LC-MS/MS experiments. Also, we are working on the investigation of metabolic transformations of xenobiotics in liver cells and in microbiota using isotopic labels [35,36]. Recently, we studied the metabolism of plants and microalgae grown in isotopically enriched media [37,38]. We also tried to measure the turnover rate of lipids in different organs of mice by administering D_2_O and measuring the deuterium uptake [29].

There are several biochemical reactions in which labile hydrogen from –OH, -SH and -NH groups is incorporated into the C-H bonds. The most important of them is the formation of malate from fumarate by the fumarase enzyme in the Krebs cycle. During this reaction, hydrogen from water attaches to the alpha carbon of malate. Administration of the D_2_O leads to the formation of labeled malate, which undergoes biochemical transformations (leading, for example, to the labeled acetyl-CoA) and serves as a major precursor of deuterated compounds. It means that administration of D_2_O allows for simultaneous assessment of turnover rates across multiple molecular classes, making this approach a promising technology for personalized medicine.

Currently, there exist several medical tests based on the administration of stable isotopes, such as the ^13^C-urea breath test [39], gastric emptying breath test [40], glucose tolerance test [41], etc. Since the administration of D_2_O results in the introduction of deuterium in a wide range of compounds, it can potentially serve as a test to simultaneously measure the rate of many metabolic processes. Another advantage of the use of D_2_O is the possibility of preserving the everyday lifestyle of the patient, including diet, sports activities, psychological conditions, etc. However, it must be acknowledged that due to the kinetic isotope effect, the chemical and biological properties of deuterated compounds may slightly differ from those of the regular compounds [42].

In the case of humans specifically, blood, urine, feces and perhaps a few more fluids (such as saliva) can be easily taken for analysis. So, it is important to determine molecules with high concentrations in those samples, which eventually become noticeably deuterated.

To achieve this, it is important to frequently take samples from the same test animal in order to exclude any personal metabolic or dietary peculiarities. Unfortunately, in the case of laboratory mice, the required amount of blood for analysis can only be taken a few times (tail vein, orbital sinus, etc.), and each blood collection considerably affects the health of the animal [43].

To our knowledge, there is no published research on a single animal or human who was administered a large dose of D_2_O for a long time, and the changes in the deuterium distribution would need to be measured for compounds belonging to different classes.

We administered 20% D_2_O to a guinea pig for 156 days and regularly collected blood (every 8 days), urine and feces (daily). The samples were also regularly collected during 70 days after the end of the D_2_O administration. Using LC-MS/MS, we have measured the deuterium uptake, and its subsequent elimination, for lipids, sterol derivatives, heme B, hemoglobin, coenzyme Q10, hippuric acid, stercobilin, etc. Though the current research is more of a “proof of principle”, we clearly demonstrated the remarkable potential of the long-term D_2_O administration for the investigation of the metabolism of the organism.

## 2. Results

**Figure 1:**
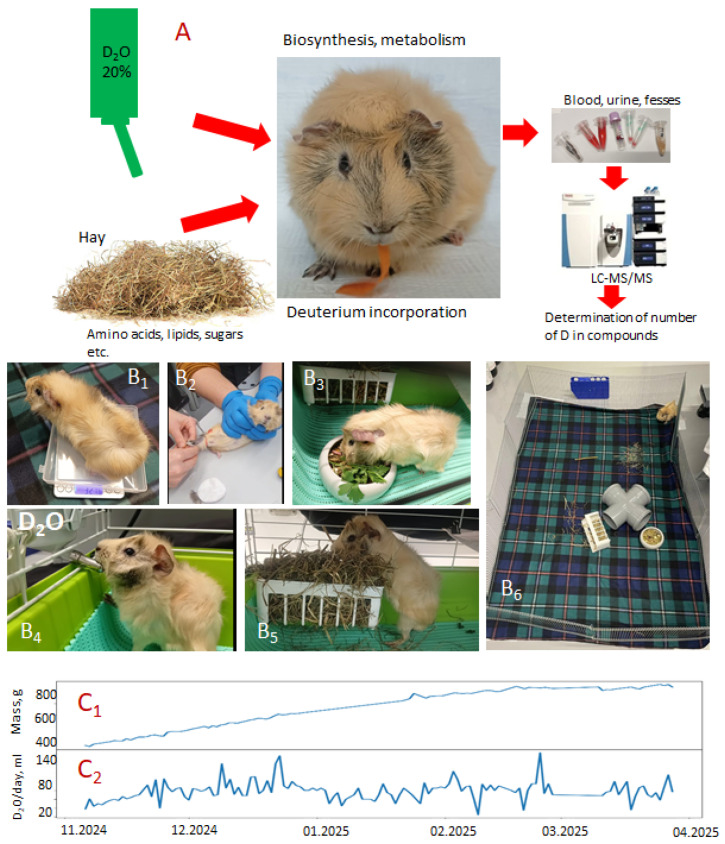
(**A**) The design of the experiment. (**B_1_**–**B_6_**) Live pictures of the guinea pig under study. (**B_2_**) Blood collection. (**B_3_**,**B_5_**) Allowed food. (**B_4_**) Water bottle with D_2_O. (**B_6_**) Enclosure in which the guinea pig spent most of its time. (**C_1_**) The weight of the guinea pig vs. the date. (**C_2_**) The variation of the daily consumption of the D_2_O.

The design of the experiment is presented in Figure 1. A male guinea pig (named Hryun, 3 months old, Figure 1A) started to only drink 20% D_2_O on 6 November 2024. The diet consisted of only dried food and dry hay, and two times a week, 10 g of parsley was given as a source of vitamin C. Images of its everyday life, including feeding, administration of the D_2_O (Figure 1(B_4_)) and blood collection (Figure 1(B_2_)), are shown in Figure 1B.

The dependence of the guinea pig’s weight on time is presented in Figure 1(C_1_). The daily consumption of D_2_O is shown in Figure 1(C_2_). The initial weight of the guinea pig was 337 g and finally reached ~850 g. The daily consumption of D_2_O varied from ~15 mL at the beginning of the experiment to ~120 mL. The average daily consumption of D_2_O was ~50 mL.

The D_2_O was administered from 6 November 2024 to 11 April 2025 (a total of 156 days). During this time, the guinea pig was constantly under observation. We did not observe any problems with the health of the guinea pig; it was active, sociable and was often willing to play with laboratory personnel. The samples of urine and feces were collected each day immediately after defecation or urination, and the blood was collected every 8 days from the paw veins. Normally, 300 μL of blood was collected each time. Samples were analyzed using an LC-MS/MS approach. We tried to avoid collecting urine and feces that spent more than several minutes in the open air to avoid interaction with the atmospheric oxygen.

Because deuterium oxide is an expensive and relatively rare reagent (according to World Bank data, the export of D_2_O by the world’s largest producer, India, was ~100,000 kg [44]), we were only able to accumulate 5 kg of pure deuterium oxide for this research. Our local suppliers were not able to provide a larger quantity. Our intention was to investigate the deuteration process for the same laboratory animal that remained alive and in good health during the whole experiment. The smallest animal capable of enduring frequent blood collection is the guinea pig. Since it is known that the turnover rate of some compounds (such as heme) can reach several months, we had to plan the experiment for at least 150 days. Due to these restrictions, we had to perform all experiments with only a single guinea pig. However, we tried to compensate for the lack of statistics by conducting a detailed personified study.

All samples were analyzed using an LC-MS/MS approach. We used a 30 min LC gradient for the lipid separation and a 40 min gradient for the metabolite separation. Analyzing experimental data, we have encountered a problem that molecules of the same compounds with different quantities of deuterium have slightly different retention times. An increase in the deuterium content leads to faster elution. This phenomenon is called the isotope effect and is well known for gas and liquid chromatography [45]. Unfortunately, this effect complicates the data processing because the observed shape of the deuterium distribution varies with time for the same compounds even within the chromatographic peak. Averaging spectra for a certain time range was found to be a bad solution because it often leads to the overlapping of the target peaks with the peaks corresponding to other compounds with close retention times. We eventually decided to select the deuterium distribution at the time when the LC peak of monoisotopic *m*/*z* reaches its maximum. We have developed a special software that helps improve compound annotation. The software was developed using Python 3.11.5—it is described in the “Materials and Methods” section.

**Figure 2:**
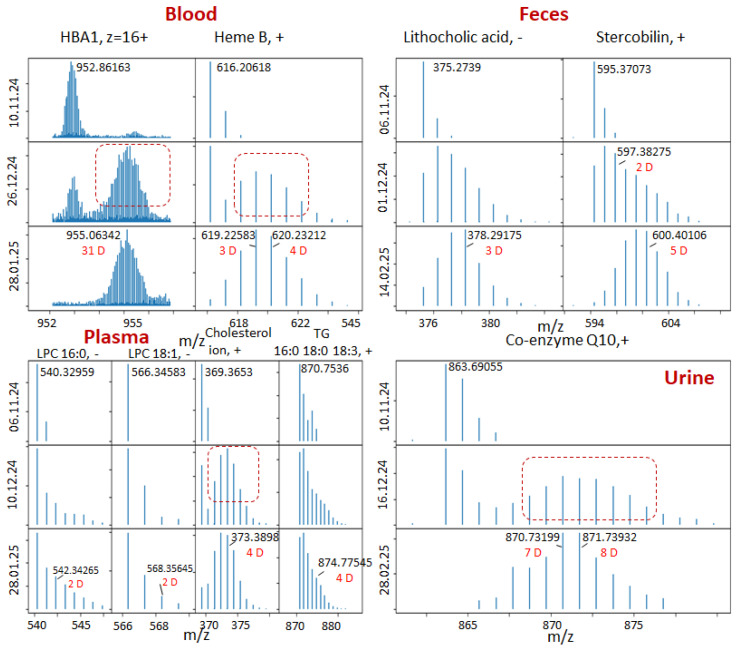
Deuterium distribution for different compounds and different dates. Negative (−) and positive (+) ESI modes. All compounds except for the HBA1 are singly charged. Red box highlights appearing deuterated compounds.

The selected results for the different compounds and different dates are presented in Figure 2. For all compounds on the initial day of the experiment, we see the expected mass spectrum corresponding to the natural isotopic distribution, formed mainly by the ^13^C isotope. For the subsequent days, we clearly see the appearance of peaks corresponding to the inclusion of the deuterium. The use of ultrahigh-resolution mass spectrometry allows us to distinguish between peaks corresponding to ^13^C/^12^C replacement (mass shift 1.0033) and D/H replacement (mass shift 1.006277). The resolving power required to distinguish those peaks for *m*/*z* = 500 is about 400,000. Such resolving power can be achieved using Fourier-transform ion cyclotron resonance mass spectrometers or modern Orbitrap mass spectrometers.

We can see that different compounds demonstrate different behavior. For hemoglobin subunit alpha, heme B, cholesterol and coenzyme Q10, we clearly see that the isotopic distribution becomes bimodal. The second envelope corresponds to the deuterium-labeled compound. The amount of these labeled compounds continuously increases, and some day, it will become equal to the amount of non-labeled compounds, which decreases, and eventually, only the labeled compound remains. For lithocholic acid and stercobilin, the isotope distribution dynamic resembles a continuous, gradual shift to the right, with the distribution remaining monomodal. For phospholipids and triglycerides, the isotopic distribution also remains monomodal but widens and shifts slightly to the right.

**Figure 3:**
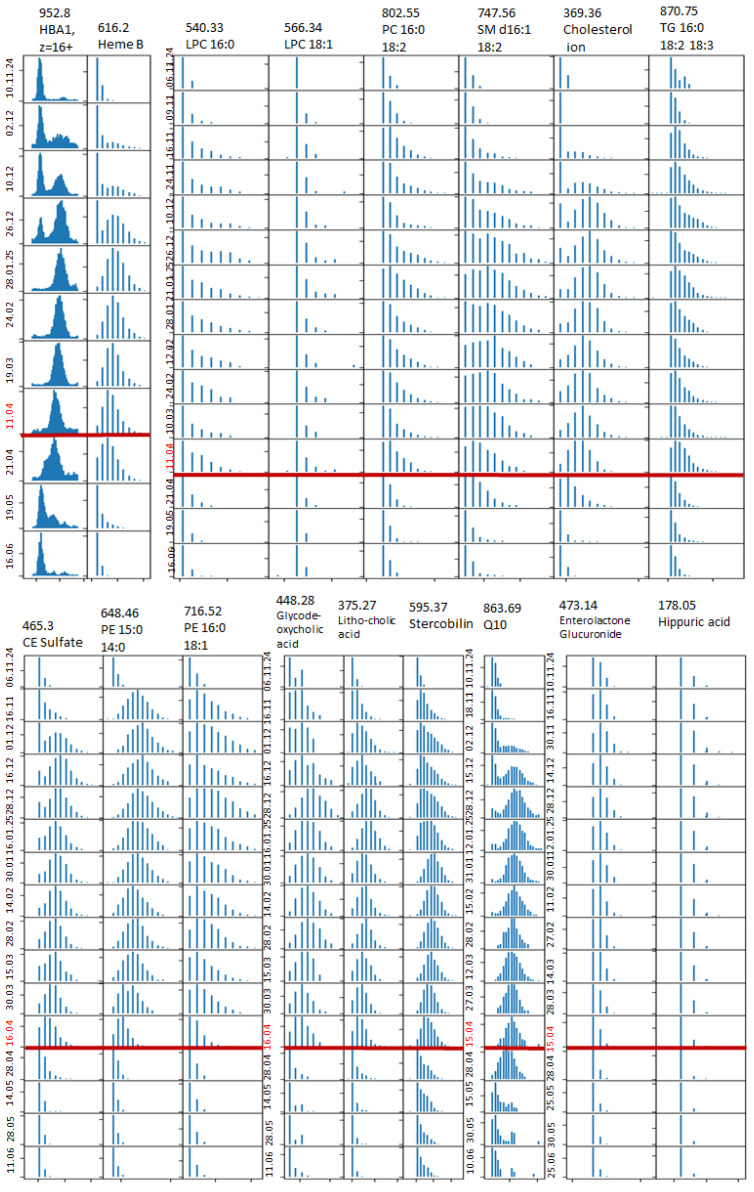
Deuterium distribution for different compounds for all dates. Negative (−) and positive (+) ESI modes. The recorded *m*/*z* and name of the compound are shown. Dates labeled with red color indicate the first day after stopping D_2_O administration. Red line highlights the end of deuterium administration.

The full dynamics of the changes in the deuterium distribution for different compounds, including the dynamics of the deuterium elimination after the stop of the D_2_O administration, are shown in Figure 3. We can see that different compounds clearly demonstrate different turnover rates. The turnover rate can be estimated as the time required for the isotopic distribution to reach its constant state. We see that there are classes of compounds for which the final isotopic distribution corresponds to the binomial distribution. It means that those compounds are generally synthesized by the organism and that the precursors coming from food undergo a considerable number of transformations. If the classical binomial distribution is not reached, it means that those compounds or their immediate precursors originate from food. We can also observe that the elimination of the deuterium occurs faster than the deuterium uptake. The selected compounds for which the turnover rate was obtained are presented in Table 1.

**Figure 4:**
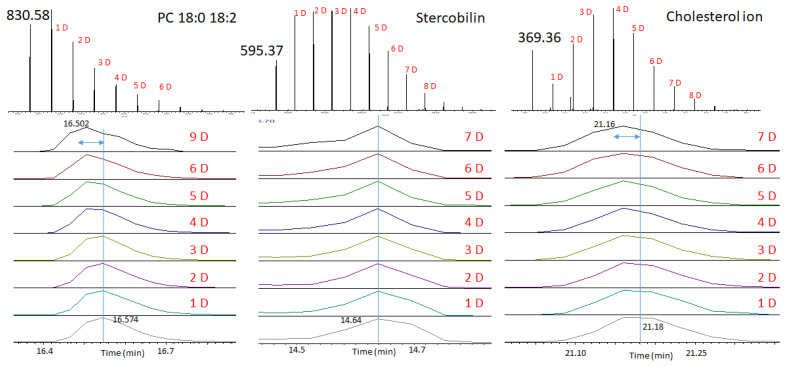
Variation of the retention time with the increase in the quantity of deuterium in the molecule.

As was previously mentioned, the replacement of hydrogen atoms with deuterium leads to the variation of the retention time. Such an effect is demonstrated in Figure 4. In this figure, we show the chromatographic elution peaks for the *m*/*z*’s corresponding to different quantities of deuterium. We can clearly see that for the PC 18:0 18:2 and cholesterol, the retention time decreases with the increase in the quantity of deuterium. For PC 18:0 18:2, the replacement of nine deuterium results in a decrease in the retention time of 0.072 min (4.3 s), and for cholesterol, the replacement of seven deuterium results in a decrease in the retention time of 0.02 min (1.2 s). Such a shift of the retention time is almost equal to the half-width of the chromatographic peak. The shift of the elution time must be taken into account when processing data. However, for the stercobilin, the variation of the retention time is almost negligible.

**Figure 5:**
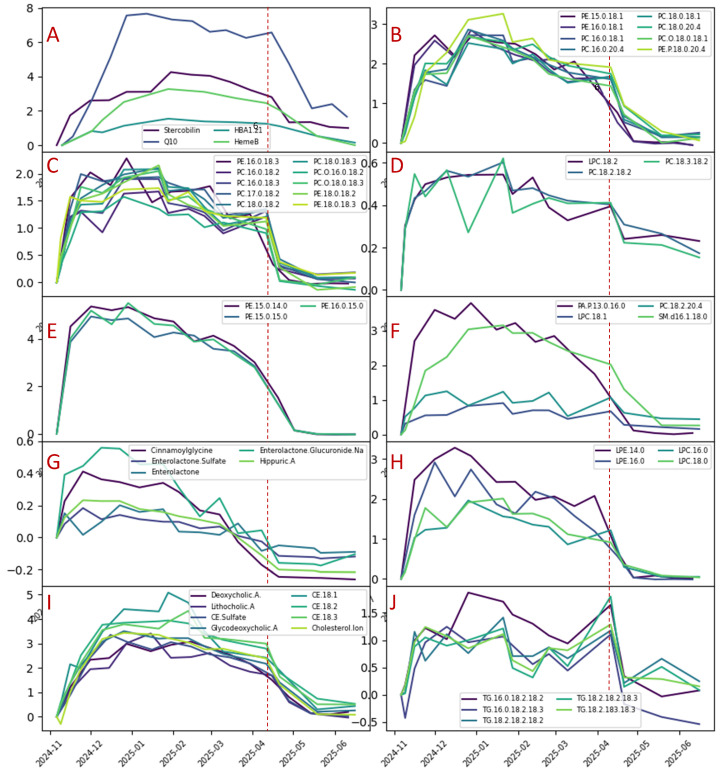
The dynamics of the deuterium uptake for different compounds. Grouping is based on the similarity of the dynamics. (**A**) Stercobilin, Q10, Heme B and HBA1^16+^. (**B**) Phospholipids where 1 FA is saturated and another is 18:1 or 20:4. (**C**) Phospholipids where 1 FA is saturated and another is 18:2 or 18:3. (**D**) Phospholipids where all FAs are 18:2 or 18:3. (**E**) Phospholipids where all FAs are saturated. (**F**) PA.P. 13:0_16:0, LPC 18:1, PC 18:2_20:4 and SMd16:1_18:0. (**G**) Low-molecular-weight metabolites: cinnamoylclycine, enterolactone, enterolactone sulfate, enterolactone glucuronide and hippuric acid. (**H**) LPE where FA is saturated. (**I**) Sterol derivatives. (**J**) TG lipids. The red line indicates the final day of the deuterium administration.

Our results for all reliably analyzed compounds are summarized in Figure 5. In this figure, we demonstrate the dependence of the shift of the center of the deuterium distribution on time. Precisely, for the recorded mass spectrum and a precursor ion *m*/*z*_p_, we select ions with an *m*/*z* that could correspond to the exchange of H for D (mass difference 1.006277) for the specified error (we used 0.001):

Abs(*m*/*z*_i_ − 1.006277n − *m*/*z*_p_) < error. Here, n = 0, 1, 2, 3, etc.

As an upper limit for n, we used 20. Then, for the selected masses, we calculated the average:

M_a_ = Sum (*m*/*z*_i_)/N. Here, i = 0, 1, 2, …N

The grouping of the compounds in Figure 5 is based on the similarity of the deuterium distribution dynamics. In Figure 5A, compounds are presented that demonstrate the longest turnover rates—stercobilin, Q10, heme B and hemoglobin. For those compounds, the maximum deuteration level was reached almost 3 months after the start of the D_2_O administration. For almost all phospholipids, reaching the maximum took 20–30 days; however, the major level of deuteration was reached after 12 days. For the sterol derivatives, reaching the maximum took 2–2.5 months. Cinnamoylclycine, enterolactone glucuronide and hippuric acid reached maximum deuteration after ~20 days. For triglycerides, the maximum was reached after ~20 days.

We can see the following values of the maximum deuterium mass shift: ~28 for hemoglobin, ~8 for Q10, up to 5 for phospholipids with both saturated FAs, 3–4 for the sterol derivatives, 4 for stercobilin, 3 for heme B, ~2.5 for phospholipids with saturated FA and 18:1 or 20:4, ~1.7 for phospholipids with saturated FA and 18:2 or 18:3, ~0.6 for phospholipids with saturated 18:2 or 18:3, ~0.6 for enterolactone glucuronide, 0.4 for cinnamoylclycine, and 0.2 for hippuric acid.

Understanding the obtained results requires a consideration of the digestive process of the guinea pig. During the experiment, the main food was dry hay. After grinding in the mouth, the food enters the stomach, where digestion begins. The enzymes involved in the digestion process are lingual lipase, secreted by the salivary gland, and gastric lipase, secreted by the gastric mucosa [46]. Lingual lipase hydrolyzes neither phosphatidylcholine (PC) nor cholesterol esterase. Lingual lipase preferentially hydrolyzes fatty acids of the TG lipids at the sn-3 position to produce DGs. The lipid emulsion enters the small intestine as fine lipid droplets and mixes with bile and pancreatic secretions. Most of the digestion of TG is performed by pancreatic lipase in the upper part of the intestinal lumen. The pancreatic lipase works at the interface between oil and aqueous phases. Pancreatic lipase acts mainly on the sn-1 and sn-3 positions of the TG. The digestion of PL occurs in the small intestine by pancreatic phospholipase. Eventually, the fat digestion products are taken up by enterocytes. The digestion of fiber occurs in the highly developed cecum [47]. Recently, it was shown that the cholesterol and lipoprotein metabolism in guinea pigs has remarkable similarities to that of human metabolism [48]. Also, it is known that the synthesis of sterols occurs in many organs, including the liver, brain [49], adrenal glands [50], etc. Like humans, guinea pigs have moderate rates of hepatic cholesterol synthesis [48].

**Figure 6:**
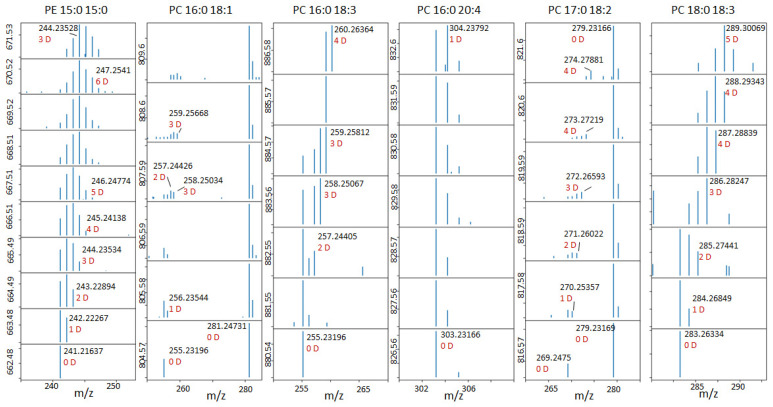
Deuterium distribution in the fatty acids of lipids observed by HCD fragmentation of negatively charged ions.

In Figure 6, we present the data of the deuterium distribution in the fatty acids of several PC lipids obtained using HCD fragmentation. PC and PE lipid anions produce fatty acid fragments, making it convenient to determine the deuterium in them. It is clear that the deuterium is present mainly in saturated fatty acids such as palmitic, stearic, margaric, etc. The 18:2 and 18:3 FAs remain unlabeled—slight labeling occurs in the 18:1 and 20:4 fatty acids.

**Figure 7:**
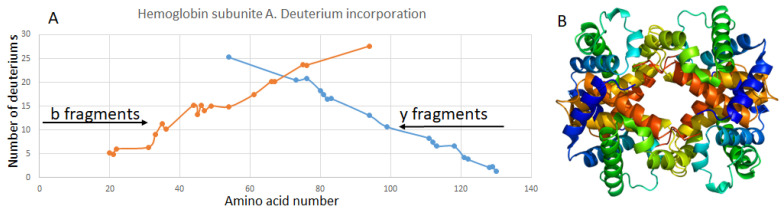
(**A**) The distribution of the deuterium in the b and y fragment ions of HBA1. CID fragmentation. (**B**) structure of hemoglobin.

We have also tried to investigate the deuterium distribution in the hemoglobin by performing a collision-induced dissociation of the HBA1 16+. We have obtained a CID fragmentation of the non-labeled and labeled ions and determined the mass shift for y- and b-fragments. Our results are shown in Figure 7. We can almost see the linear dependence of the number of labels on the length of the fragment.

**Figure 8:**
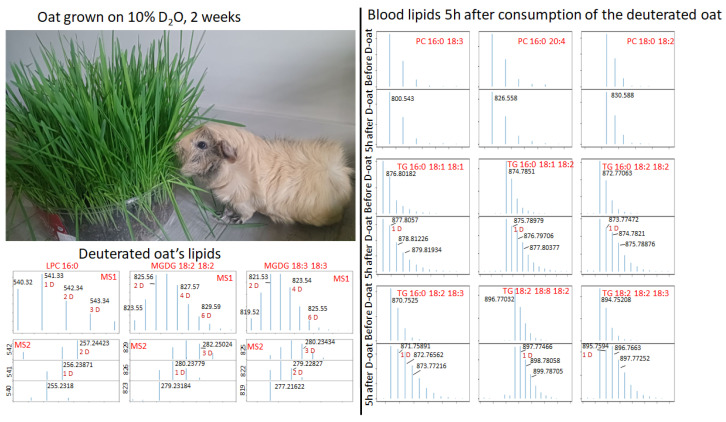
Administration of the deuterated oat to the guinea pig and observation of the deuterated lipids in its blood.

To further investigate the influence of the compounds coming with the food on the deuterium labeling experiment, we have grown a deuterated oat using 10% D_2_O as the only source of water, and the same guinea pig consumed this oat (Figure 8). The level of deuteration of the grown oat is shown in Figure 8. We can see that MGDG lipids of oat clearly demonstrate the inclusion of many deuterium labels, with a maximum of three deuterium. The experiment was performed on 8 July 2025, 3 months after the last D_2_O administration. During this time, all deuterium-labeled compounds were eliminated from the organism.

The guinea pig was starved overnight and then allowed to freely consume deuterated oat. Blood was collected after 5 h. During this time, the guinea pig consumed 50 g of deuterated oat. Our results are presented in Figure 8. It can be seen that the shape of the isotopic distribution of PC lipids did not change, while the isotopic distribution of the TG lipids changed considerably. In other compounds of blood and urine collected 7 h after the start of consumption of deuterated oat, we did not observe any traces of the deuterium.

## 3. Discussion

Our experiments showed the possibility of simultaneously investigating the deuteration rate of biological compounds belonging to different classes. In our study, all experiments were performed with a single animal, which still lives and (at the date of the article writing) remains healthy despite the consumption of ~20 L of 20% D_2_O over 156 days. During the experiment, we did not observe any remarkable changes in the behavior compared to other guinea pigs. We compensated for the lack of statistics by conducting a detailed personified study.

Quickly consumed D_2_O is distributed along the body (in minutes), and deuterium starts to participate in the biochemical reactions along with hydrogen. All labile hydrogen from -OH, -SH and -NH groups exchange their hydrogen for deuterium almost immediately. Therefore, only deuterium in the C-H groups is useful for the turnover rate studies.

There are many biochemical reactions in which hydrogen or deuterium from water can be incorporated into the C-H positions. There are also processes in which certain enzymes, such as cytochromes, activate C-H hydrogens in specific positions and facilitate their exchange with solvents [35,51]. Currently, many databases of metabolic pathways exist that can be used for the determination of the reactions that lead to the synthesis of isotopically labeled compounds. The following databases are known: KEGG [52,53], MetaCyc [54], Reactome [55,56], WikiPathways [57,58], Pathway Commons [59,60] and many others.

**Figure 9:**
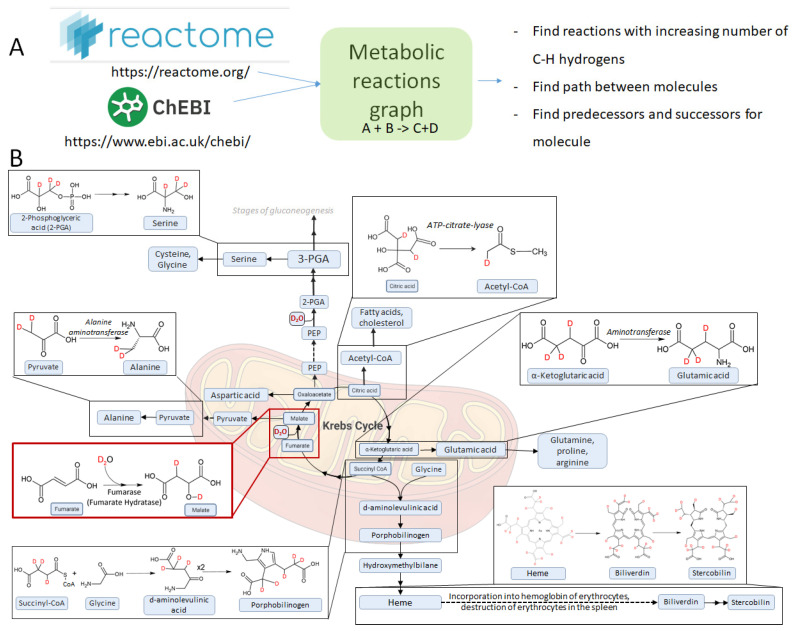
(**A**) Schematic approach for the analysis of metabolic reactions. (**B**) Selected reactions and pathways involved in the deuterium labeling.

We have made an attempt to use a Reactome database to find all pathways in which D_2_O can be included in the C-H hydrogens of endogenous molecules (see Figure 9A). Processing a database of the reactions revealed >900 reactions; among those reactions, H_2_O participates in >300 reactions. We have found several reactions in which the number of C-H hydrogens increases; however, the most important reaction is, expectedly, the synthesis of malate from fumarate in the Krebs cycle. Later, the deuterium from the malate is incorporated into oxaloacetate, then into citrate. From the citrate, the deuterium is incorporated into acetyl-CoA, and since acetyl-CoA participates in many biological reactions, the deuterium is distributed along other biological compounds. From the acetyl-CoA, the deuterium is incorporated into the fatty acids and cholesterol.

Malate is converted into pyruvate, retaining deuterium. Deuterated pyruvate is converted into the deuterated alanine. Deuterated citrate eventually produces deuterated alpha-ketoglutarate, which results in deuterated glutamate and deuterated succinyl-CoA. Succinyl-CoA interacts with glycine, producing aminolevulinate and then porphobilinogen, which eventually produces heme. Heme breaks down and, through the chain of transformations, eventually becomes stercobilin. Metabolism of deuterated cholesterol leads to deuterated bile acids. Almost all of the important pathways are shown in Figure 9B.

## 4. Materials and Methods

**Guinea pig handling.** The healthy male guinea pig (named Hryn) was purchased from local vivarium (initial weight 337 g). The guinea pig was kept either in cage or in enclosure to ensure sufficient physical and social activity. Animal was satisfied with the conventional category. Regular rodent food (standard granular compound feed for laboratory animals (extruded)) and dry hay were provided. For the labeling experiments, the water bottle was regularly filled with deuterated water (20%). Small slices of carrot and parsley were provided 2 times a week (10 g) as the source of vitamins. The climate was maintained at a room temperature of 22 °C (+/− 2), room humidity of 60–70% and a 12/12 light/dark cycle regimen. All experiments were carried out in accordance with the ethical principles and regulations recommended by the European Convention for the Protection of Vertebrate Animals used for Experiments.

**Sample collection.** Feces and urine were collected several times a day immediately after bowel movements. Blood was collected every 8 days from paw veins. Between first blood collection (before the start of D_2_O administration) and second (D_2_O was administered), 4 days passed. Blood was immediately centrifuged at 3000 rpm, and plasma was collected and stored at −25 °C. Feces and urine were also stored in Eppendorf tubes at −25 °C.

**Sample preparation.** For the sample preparation, the following protocol was used:

1. A total of 300 μl of cold methanol was added to 40 ul of aliquots (plasma or urine) of the sample and vigorously shaken on a shaker for 1 min. In case of feces, 30 mg of sample was taken and homogenized in the solvent;
2. A total of 1 mL of cold MTBE was added, and the mixture was treated with ultrasound for 10 min and incubated for 40 min at 4 °C with stirring;
3. A total of 250 ul of water was added to the extract to separate the phases. The extract was shaken for 1 min at 4 °C, then centrifuged for 10 min at 13,000 rpm at 4 °C;
4. An aliquot of 1000 µL of the upper layer containing nonpolar components was collected in a separate vial. Low layer containing polar compounds was used for measuring metabolites;
5. A total of 400 µL of buffer (MeOH:MTBE:H_2_O = 3:10:2.5) was added to the lower phase for repeated extraction;
6. The sample was shaken and centrifuged for 10 min at 13,000 rpm at 4 °C. The upper fractions were combined (a total of 1300 µL) and evaporated until dry in a vacuum concentrator at room temperature;
7. The dry residue was re-dissolved in 200 ul of a mixture of acetonitrile/isopropanol cooled to 0 °C (7:3 (vol/vol));
8. The sample was shaken for 10 min, kept in an ice-cooled ultrasonic bath for 10 min and centrifuged for 5 min at 13,000 rpm;
9. Before the HPLC MS analysis, the samples were diluted 1:5 and 1:2 with a mixture of acetonitrile/isopropanol (7:3 (vol/vol)) for measurements in the registration mode of positively and negatively charged ions, respectively.

For the measurement of the hemoglobin and heme b, 10 ul of blood was dissolved in 1ml of 1:1 MeOH:H_2_O with addition of 1% formic acid and shaken. Addition of formic acid is important for the shifting of the charge distribution of hemoglobin to the region of *m*/*z* 500–1500. Without the addition of formic acid, the charge distribution spans the *m*/*z* region 1500–3500, and the peaks considerably widen.

**LC-MS/MS analysis** was performed using Acela HPLC system (Thermo, Waltham, MA, USA) and Velos Orbitrap (Thermo) mass spectrometer in positive and negative polarities. Ions were generated in HESI source. Reversed-phase Bridged Ethyl Hybrid (BEH) C18 column (100 mm × 2.1 mm, 1.7 µm) was coupled with a Vanguard pre-column with the same solid phase. A binary solvent system was used for the chromatographic separation.

For the separation of lipids, the following conditions were used: Buffer A (water containing 10 mM ammonium acetate, 0.1% formic acid) and Buffer B (acetonitrile/isopropanol (7:3 (*v*:*v*)) containing 10 mM ammonium acetate, 0.1% formic acid). The gradient for separation was programmed as follows: 0 min—30% B; 0.5 min—70% B; 16 min—linear gradient from 30% to 100% B; 24 min—100% B; 25 min—linear gradient from 100% to 30% B; and 30 min—30% B. The flow rate was kept at 150 µL/min during the whole 30 min run, and the column temperature was set at 25 °C.

For the separation of metabolites, the following conditions were used: Buffer A (water containing 0.1% formic acid) and Buffer B (acetonitrile, containing 0.1% formic acid). The gradient for separation was programmed as follows: 0 min—5% B; 5 min—5% B; 25 min—linear gradient from 5% to 75% B; 26 min—100% B; 33 min—100% B; 35 min—linear gradient from 100% to 5% B; and 40 min—5% B. The flow rate was kept at 250 µL/min during the whole 40 min run, and the column temperature was set to 25 °C.

HESI source tune parameters for ionization were set as follows: heater temperature: 280 °C; capillary temperature: 350 °C; HESI voltage: 4.0 kV (−3.5 kV); sheath gas flow rate (N_2_): 35 arbitrary units (a.u.); auxiliary gas flow rate (N_2_): 35 a.u.; sweep gas flow rate (N_2_): 0 a.u.; and S-lens RF level: 60. Data acquisition was performed in data-dependent mode in positive and negative polarities separately. For the full scan events, operating parameters were set as listed: resolution: 60,000 at *m*/*z* 200; automatic gain control (AGC target): 5^5^; maximum injection time (IT): 50 ms; and scan range: 100 to 2000 Da. For DDA mode, topN: 5; resolution 15,000 at *m*/*z* 200; and mass isolation window: 1.5 Da. Injection volume of 3 μL in both positive and negative ionization modes was used.

**Data processing.** The processing was performed using Thermo Xcalibur 4.7 software, Thermo Compound Discoverer 3.3 and mzCloud database. For the lipid identification, we used Lipid Maps database and fragmentation rules described in the review by Murphy and Axelsen [61]. Lipids were annotated according to their accurate precursor and fragment characteristic ions. All identified lipids were manually curated, and then expected lipid elution patterns were considered for further confirmation. Namely, retention time should increase for the series of homologues and decrease with addition of double bonds.

Analysis of the deuterium distribution was performed semi-manually. For each identified compound, we have extracted spectra at the corresponding retention time in the vicinity of the corresponding *m*/*z* and visually analyzed the shape of the isotopic distribution and the mass difference between peaks. The main problem affecting the analysis was short elution time and the overlapping of deuterium peaks with ^13^C peaks and peaks corresponding to other lipids.

For convenient and reliable data processing, especially processing deuterium labeling data, a series of software was developed.

**Single LC-MS/MS data viewer**. For the panoramic data processing and detection of the compounds that engaged in the deuterium labeling, we have developed a special software using Python language. The software can read .raw files using open-source fisher-py package. The interactive screen consists of several parts (see Supplementary Figure S1): LC view, a MS^1^ spectrum view and several parts for the viewing of the zoomed parts of the MS^1^ spectrum. Red dots at the bottom of the peak indicate that for this *m*/*z*, there is an MS^2^ spectrum recorded in the vicinity of 10 scans from the current scan. Clicking on the red dot creates a separate pop-up window showing, for the selected *m*/*z*, the nearest MS^2^ spectrum, extracted ion chromatogram (EIC), zoomed EIC (with the line indicating the time of the MS^2^ recording), MS^1^ spectrum at selected scan and zoomed MS^1^ spectrum. The developed software allowed for the quick analysis of the LC-MS spectra visually and for the visual detection of the compounds whose isotopic distribution changed. It is also very convenient to quickly check the MS^2^ spectra of the compounds that aroused interest. The developed software allows for making sure that even for the narrow LC peaks, the recorded MS^2^ s spectrum corresponds to the selected MS1 *m*/*z* peak. Simultaneous observation of the several zoomed parts of the MS^1^ spectrum allows for the visual detection of compounds arising at the same time, such as dimers, adducts and in-source fragmentation products.

**Multiple LC-MS/MS data viewer**. During the analysis of the deuterium isotopic distribution changes, we faced a problem that even slight variation of the elution time for the compounds between LC runs requires very accurate selection of the time points at which MS^1^ spectra are selected for further analysis of the changes in the isotopic distribution. We have developed a software that, for a given *m*/*z*, allows for the simultaneous opening of several LC-MS/MS spectra and manually choosing the suitable time point for the selection of spectra for further deuterium distribution analysis. The software also shows *m*/*z* for which there exists an MS^2^ spectrum in the vicinity of 10 scans from the current scan. This allows for verifying the compound under study and avoiding mistakes in the selection of time points for further analysis.

The screenshot of the developed multiple LC-MS/MS data viewer is shown in Supplementary Figure S2. The developed software also allows us to select for the *m*/*z* under study only in compounds with mass difference that is equal to 1.006277*n (n—number of H/D exchanges). This allows us to even analyze compounds with low intensity.

To increase the reliability of the data processing, for each compound, we create a special picture unifying MS^2^ spectrum, EIC for this compound for all dates, MS spectra showing the changes in the deuterium distribution for all dates and a shift of the center of the deuterium distribution depending on the date. The example is shown in Supplementary Figure S3. Such pictures allow for easy comparison of data for different compounds, analysis of many compounds simultaneously and, most importantly, considerable decreases in the mistakes in the compound identification or choosing incorrect time point. Pictures generated for all compounds are also provided in the Supplementary Materials.

**Reactome database processing**. All data were downloaded in the Biopax 3 format and parsed using Python. Chemical structures (SMILES) were assigned by mapping ChEBI identifiers to the compounds in the ChEBI database. For the reaction analysis, we used Python and NetworkX and PyDot libraries. For the visualization, we used Graphviz software (https://graphviz.org/ accessed 1 July 2025). All scripts are placed in the Supplementary Materials.

## 5. Conclusions

We have successfully determined compounds in blood, urine and feces, which may serve as good candidates for the turnover rate measurement for medical analysis and determination of its deuteration time. Application of the deuterium oxide allows for simultaneous assessment of the deuteration rate for compounds of multiple biological classes. However, its application has several limitations, one of which is the slight difference in the chemical properties between hydrogen and deuterium.

The other limitation arises from the fact that the deuteration rate of the compound depends on the number of stages (and its kinetic properties) of the metabolic pathway connecting this compound and the nearest compound that can include deuterium from water in its C-H bonds. For many compounds, this precursor is malate. It means that the rate of deuteration of a protein actually reflects the rate of all stages of the metabolic pathway connecting this protein to malate, including amino acid synthesis, its transport, protein assembly, etc. Therefore, the turnover rate of protein measured by D_2_O administration will always be longer than that measured by administration of labeled amino acids.

Though the current research is more of a “proof of principle”, it is evident that long-term D_2_O administration has remarkable potential for the investigation of metabolism. We hope to perform future experiments with human volunteers using considerably low doses of D_2_O (~2%), but we will try to increase the time of the experiment as long as possible.

## Figures and Tables

**Table 1:** Selected compounds for which the turnover rate was determined. The difference between metabolic and lipid LC gradients is discussed in the “Materials and Methods” section. For heme B, the accurate mass corresponds to the most abundant isotopic peak.

Polarity	Gradient	Sample	Compound Name	Precursor *m*/*z* [Da]	Ionization Type	Molecular Formula of Ions	Accurate Mass [Da]	Delta [Da]	Retention Time [min]
Neg	Lipids	feces	Cholesterol sulfate	465.304	[M − H]-	C27H45O4S	465.303	0.001	12.24
Neg	Lipids	feces	Lithocholic acid	375.290	[M − H]-	C24H39O3	375.289	0.001	6.35
Neg	Lipids	feces	PE 15:0 15:0	662.477	[M − H]-	C35H69NO8P	662.476	0.001	15.68
Neg	Lipids	feces	PE 14:0 16:0	662.477	[M − H]-	C35H69NO8P	662.476	0.001	15.68
Neg	Lipids	feces	PE 15:0 14:0	648.461	[M − H]-	C34H67NO8P	648.460	0.002	15.2
Neg	Lipids	feces	PE 16:0 18:3	712.492	[M − H]-	C39H71NO8P	712.491	0.001	15.28
Neg	Lipids	feces	PE 15:0 18:1	702.507	[M − H]-	C38H73NO8P	702.507	0.000	16.24
Neg	Lipids	feces	PE 16:0 18:1	716.523	[M − H]-	C39H75NO8P	716.522	0.001	16.64
Neg	Lipids	feces	PE 16:0 15:0	676.492	[M − H]-	C36H71NO8P	676.491	0.001	16.04
Neg	Lipids	feces	LPG 16:0	483.273	[M − H]-	C22H44O9P	483.272	0.001	6.59
Neg	Lipids	feces	LPE 16:0	452.278	[M − H]-	C21H43NO7P	452.277	0.001	7.8
Neg	Lipids	feces	PA P-13:0 16:0	589.451	[M − H]-	C32H62O7P	589.423	0.028	15.48
Neg	Lipids	feces	LPE 14:0	424.246	[M − H]-	C19H39NO7P	424.246	0.000	5.21
Neg	Lipids	urine	Cholesterol sulfate	465.305	[M − H]-	C27H45O4S	465.303	0.001	12.19
Neg	Lipids	urine	PE P-16:0 20:4	722.511	[M − H]-	C41H73NO7P	722.512	−0.001	16.07
Pos	Lipids	urine	Q10	863.690	[M + H]+	C59H91O4	863.691	−0.001	19.26
Pos	Lipids	plasma	TG 18:2 18:3 18:3	892.737	[M + NH4]+	C57H98O6N	892.739	−0.001	18.65
Pos	Lipids	plasma	TG 16:0 18:1 18:1	876.801	[M + NH4]+	C55H106O6N	876.801	−0.001	20.8
Pos	Lipids	plasma	TG 16:0 18:1 18:2	874.784	[M + NH4]+	C55H104O6N	874.786	−0.002	20.33
Pos	Lipids	plasma	TG 16:0 18:2 18:2	872.769	[M + NH4]+	C55H102O6N	872.770	−0.001	19.95
Pos	Lipids	plasma	TG 16:0 18:2 18:3	870.753	[M + NH4]+	C55H100O6N	870.755	−0.001	19.5
Pos	Lipids	plasma	TG 18:2 18:2 18:2	896.769	[M + NH4]+	C57H102O6N	896.770	−0.001	19.44
Pos	Lipids	plasma	TG 18:2 18:2 18:3	894.752	[M + NH4]+	C57H100O6N	894.755	−0.003	19.03
Pos	Lipids	plasma	Cholesterol ion	369.365	M+	C27H45	369.352	0.014	21.07
Pos	Lipids	plasma	CE 18.1	673.589	[M + Na]+	C45H78O2Na	673.589	0.000	21.83
Pos	Lipids	plasma	CE 18.2	671.574	[M + Na]+	C45H76O2Na	671.574	0.000	21.21
Pos	Lipids	plasma	CE 18.3	669.558	[M + Na]+	C45H74O2Na	669.558	0.000	20.78
Neg	Lipids	plasma	PC O-16:0 18:2	788.543	[M + Formate]-	C43H83NO9P	788.580	−0.037	15.167
Neg	Lipids	plasma	PE P-18:0 20:4	750.543	[M − H]-	C43H77NO7P	750.543	0.000	16.8
Neg	Lipids	plasma	PE P-18:0 18:2	726.543	[M − H]-	C41H77NO7P	726.543	0.000	17.048
Neg	Lipids	plasma	PC 17:0 18:2	816.575	[M + Formate]-	C44H83NO10P	816.575	0.000	16.048
Neg	Lipids	plasma	PC O-18:0 18:3	814.560	[M + Formate]-	C44H81NO10P	814.559	0.001	15.447
Neg	Lipids	plasma	LPC 18:2	564.330	[M + Formate]-	C27H51NO9P	564.330	0.001	6.15
Neg	Lipids	plasma	PE 18:0 18:3	740.523	[M − H]-	C41H75NO8P	740.522	0.000	16.048
Neg	Lipids	plasma	PE 18:0 18:2	742.538	[M − H]-	C41H77NO8P	742.538	0.000	16.64
Neg	Lipids	plasma	PE 18:2 18:2	738.506	[M − H]-	C41H73NO8P	738.507	−0.001	15.04
Neg	Lipids	plasma	PE 18:0 18:1	744.551	[M − H]-	C41H79NO8P	744.554	−0.003	17.3
Neg	Lipids	plasma	PC 18:2 18:2	826.558	[M + Formate]-	C45H81NO10P	826.559	−0.001	14.8
Neg	Lipids	plasma	PC 18:2 20:4	850.559	[M + Formate]-	C47H81NO10P	850.559	0.000	14.52
Neg	Lipids	plasma	LPC 16:0	540.330	[M + Formate]-	C25H51NO9P	540.330	0.001	7.813
Neg	Lipids	plasma	LPC 18:0	568.361	[M + Formate]-	C27H55NO9P	568.361	0.001	10.06
Neg	Lipids	plasma	LPC 18:1	566.345	[M + Formate]-	C27H53NO9P	566.345	0.000	7.93
Neg	Lipids	plasma	PC 18:0 18:2	830.590	[M + Formate]-	C45H85NO10P	830.591	0.000	16.44
Neg	Lipids	plasma	PC 18:0 18:2	770.570	[M − CH3]-	C43H81NO8P	770.569	0.000	16.44
Neg	Lipids	plasma	PC 16:0 18:2	802.559	[M + Formate]-	C43H81NO10P	802.559	0.000	15.608
Neg	Lipids	plasma	PC 18:0 18:1	832.606	[M + Formate]-	C45H87NO10P	832.606	0.000	17.08
Neg	Lipids	plasma	PC 16:0 18:1	804.575	[M + Formate]-	C43H83NO10P	804.575	0.000	16.32
Neg	Lipids	plasma	PC 18:3 18:2	824.544	[M + Formate]-	C45H79NO10P	824.544	0.000	14.08
Neg	Lipids	plasma	PC 18:0 18:3	828.575	[M + Formate]-	C45H83NO10P	828.575	0.000	15.848
Neg	Lipids	plasma	PC 18:0 18:3	768.555	[M − CH3]-	C43H79NO8P	768.554	0.001	15.84
Neg	Lipids	plasma	PC 16:0 18:3	800.544	[M + Formate]-	C43H79NO10P	800.544	0.000	14.96
Neg	Lipids	plasma	PC 16:0 20:4	826.560	[M + Formate]-	C45H81NO10P	826.559	0.000	15.36
Neg	Lipids	plasma	PC 18:0 20:4	854.592	[M + Formate]-	C47H85NO10P	854.591	0.001	16.208
Neg	Lipids	plasma	PC O-18:0 18:1	818.591	[M + Formate]-	C44H85NO10P	818.591	0.001	16.727
Neg	Lipids	plasma	Cholesterol sulfate	465.304	[M − H]-	C27H45O4S	465.303	0.000	12.09
Neg	Lipids	plasma	Deoxycholic acid	391.285	[M − H]-	C24H39O4	391.284	0.000	3.97
Neg	Lipids	plasma	Glycodeoxycholic acid	448.306	[M − H]-	C26H42NO5	448.306	0.000	2.8
Neg	Lipids	plasma	SM d16:1 18:0	687.543	[M-CH3]-	C38H76N2O6P	687.544	0.000	15.94
Neg	Lipids	plasma	SM d16:1 18:0	747.565	[M + Formate]-	C40H80N2O8P	747.565	0.000	15.94
Pos	Direct	blood	Heme B	616.207	M+	C34H32FeN4O4	616.177	0.030	
Pos	Direct	blood	Heme C	684.197	M+	C34H36O4N4S2Fe	684.152	0.045	
Pos	Direct	blood	HBA1 18+	847.160	[M + 18H]18+				
Pos	Metab.	feces	Stercobilin	595.371	[M + H]+	C33H47N4O6	595.349	0.022	14.66
Neg	Metab.	urine	Hippuric acid	178.051	[M − H]-	C9H8NO3	178.050	0.001	4.08
Neg	Metab.	urine	Cinamoyl glycine	204.066	[M − H]-	C11H10NO3	204.066	0.001	10.62
Neg	Metab.	urine	Enterolactone glucuronide	473.145	[M − H]-	C24H25O10	473.144	0.001	12.66
Neg	Metab.	urine	Cinamoyl glycine	272.054	[M − H + Na.Formate]-	C12H11NO5Na	272.053	0.001	10.62
Neg	Metab.	urine	Enterolactone sulfate	377.069	[M − H]-	C18H17O7S	377.069	0.000	12.58
Neg	Metab.	urine	Enterolactone	297.112	[M − H]-	C18H17O4	297.112	0.000	14.62
Pos	Metab.	urine	Hippuric acid	180.065	[M + H]+	C9H10NO3	180.066	0.000	4.13
Pos	Metab.	urine	Enterolactone glucuronide	497.146	[M + Na]+	C24H26O10Na	497.142	0.005	12.76
Neg	Metab.	feces	Lithocholic acid	375.274	[M − H]-	C24H39O3	375.289	−0.015	26.41
Neg	Metab.	feces	Deoxycholic acid	391.268	[M − H]-	C24H39O4	391.284	−0.016	19.69
Neg	Metab.	feces	Glycodeoxycholic acid	448.287		C26H42NO5	448.306	−0.019	19.35
Neg	Metab.	feces	Stercobilin	593.308	[M − H]-	C33H45N4O6	593.333	−0.025	14.81
Neg	Lipids	d-oat	MGDG 18:3 18:3	819.524	[M + Formate]-	C46H75O12	819.525	−0.001	14.08
Neg	Lipids	d-oat	LPC 16:0	540.329	[M + Formate]-	C25H51NO9P	540.330	0.000	8.25
Neg	Lipids	d-oat	MGDG 18:2 18:2	823.556	[M + Formate]-	C46H79O12	823.557	0.000	15.57

## Data Availability

The data will be provided upon requests sent to the authors.

## Supplementary Materials

The following supporting information can be downloaded at https://www.mdpi.com/article/10.3390/ijms27041944/s1.
