# Supplementary material for: Turnover Rate of Lipids, Metabolites and Proteins Revealed by 156-Day-Long D2O Administration in a Guinea Pig
Source: Int J Mol Sci. 2026 Feb 18;27(4):1944. doi: 10.3390/ijms27041944 (PMC12941004; doi:10.3390/ijms27041944)

## Slide 1
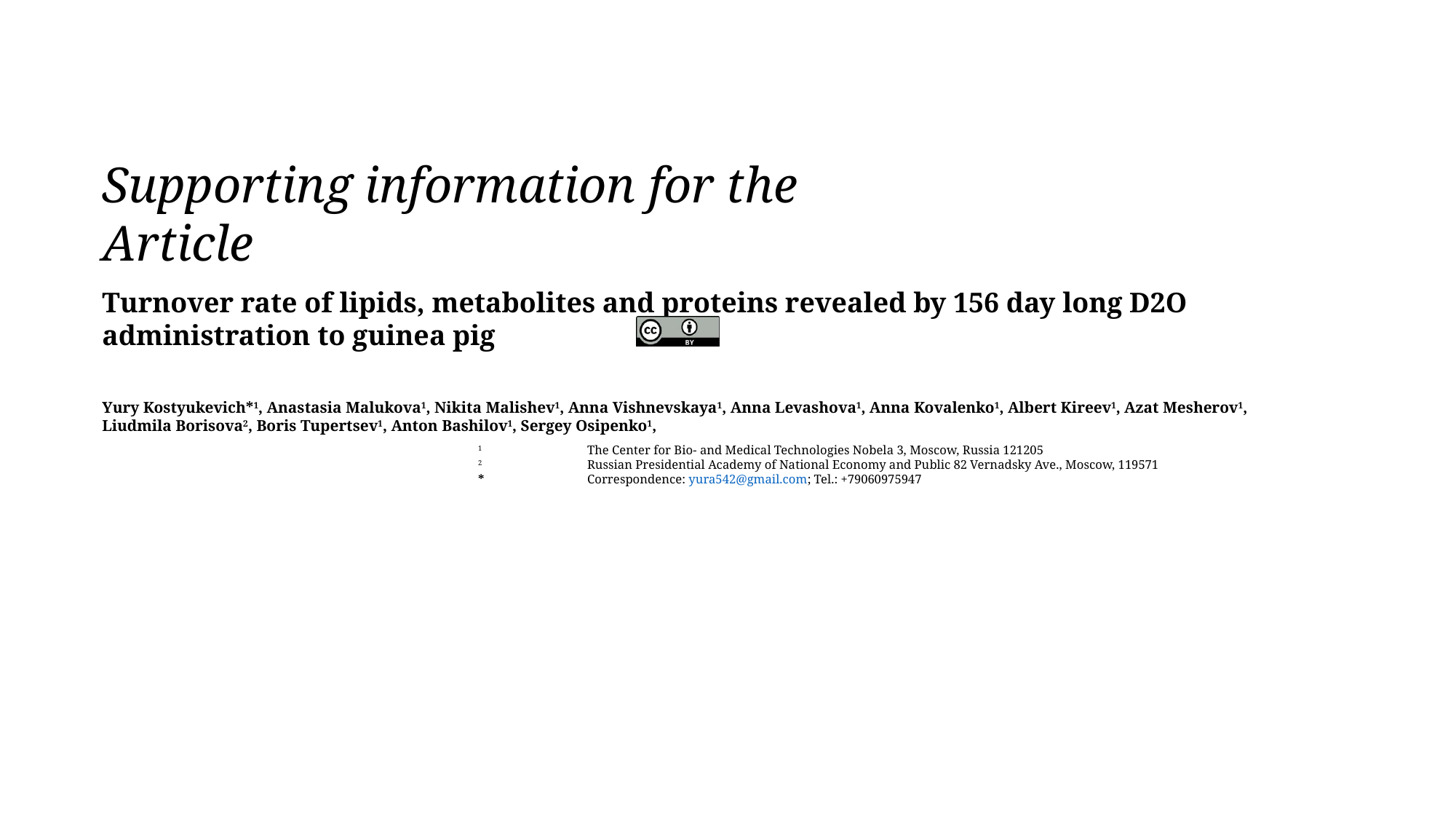

Supporting information for the
Article
Turnover rate of lipids, metabolites and proteins revealed by 156 day long D2O administration to guinea pig
Yury Kostyukevich*1, Anastasia Malukova1, Nikita Malishev1, Anna Vishnevskaya1, Anna Levashova1, Anna Kovalenko1, Albert Kireev1, Azat Mesherov1, Liudmila Borisova2, Boris Tupertsev1, Anton Bashilov1, Sergey Osipenko1,
1	The Center for Bio- and Medical Technologies Nobela 3, Moscow, Russia 121205
2	Russian Presidential Academy of National Economy and Public 82 Vernadsky Ave., Moscow, 119571
*	Correspondence: yura542@gmail.com; Tel.: +79060975947

## Slide 2
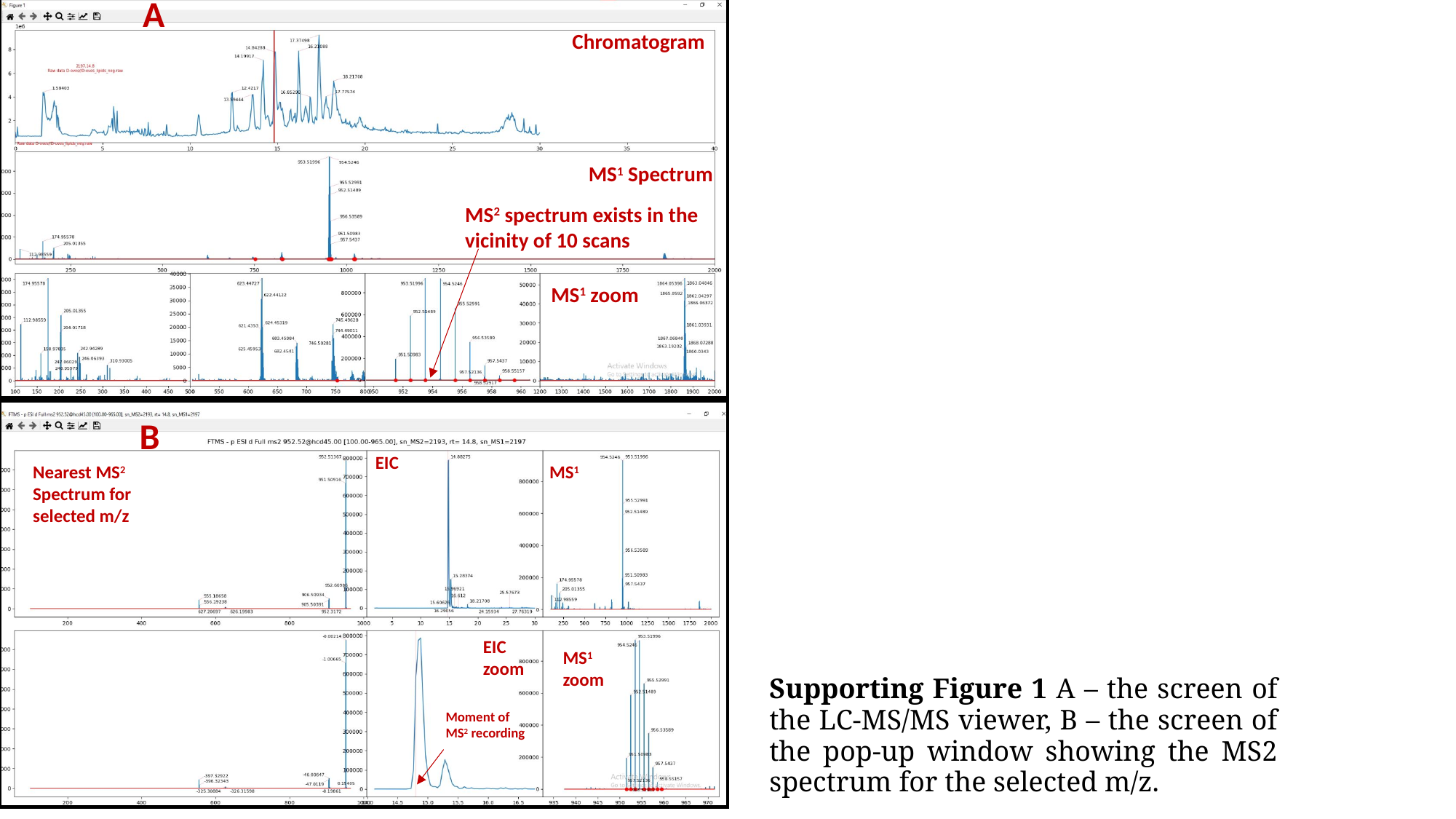

A
Chromatogram
MS1 Spectrum
MS2 spectrum exists in the vicinity of 10 scans
MS1 zoom
B
EIC
Nearest MS2 Spectrum for selected m/z
MS1
EIC zoom
MS1 zoom
Supporting Figure 1 A – the screen of the LC-MS/MS viewer, B – the screen of the pop-up window showing the MS2 spectrum for the selected m/z.
Moment of MS2 recording

## Slide 3
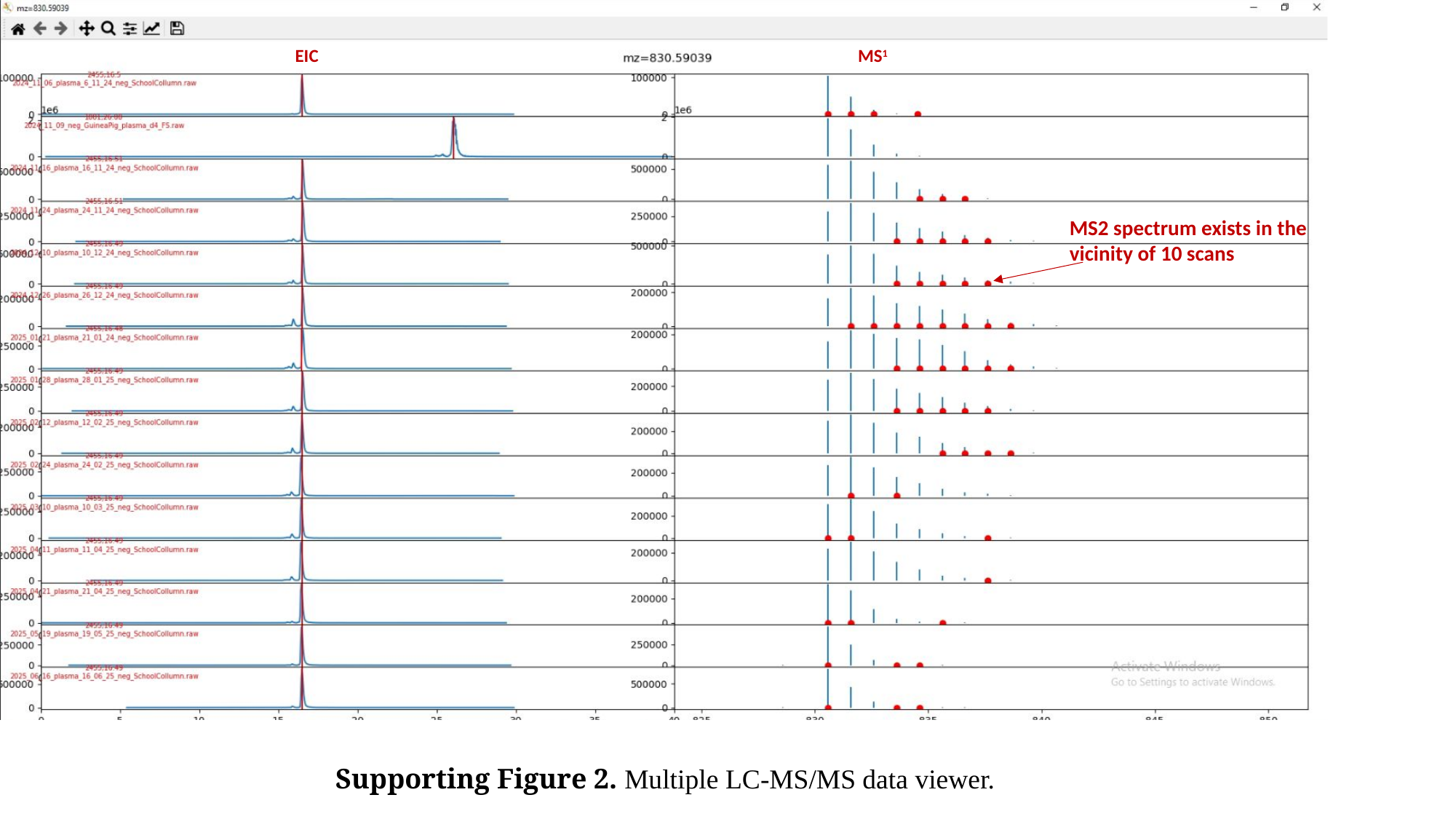

EIC
MS1
MS2 spectrum exists in the vicinity of 10 scans
Supporting Figure 2. Multiple LC-MS/MS data viewer.

## Slide 4
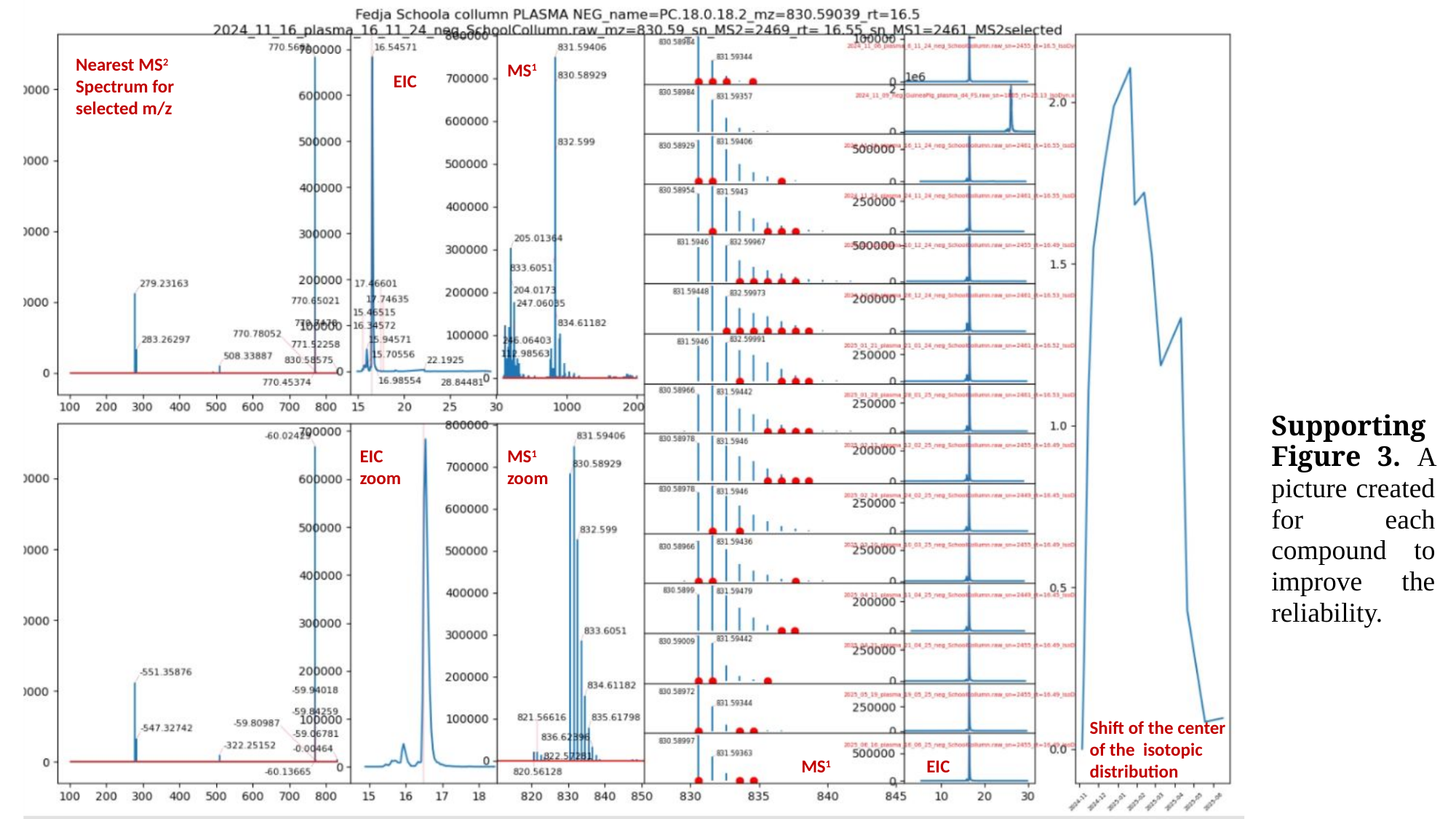

Nearest MS2 Spectrum for selected m/z
MS1
EIC
Supporting Figure 3. A picture created for each compound to improve the reliability.
MS1 zoom
EIC zoom
Shift of the center of the isotopic distribution
MS1
EIC

## Slide 5
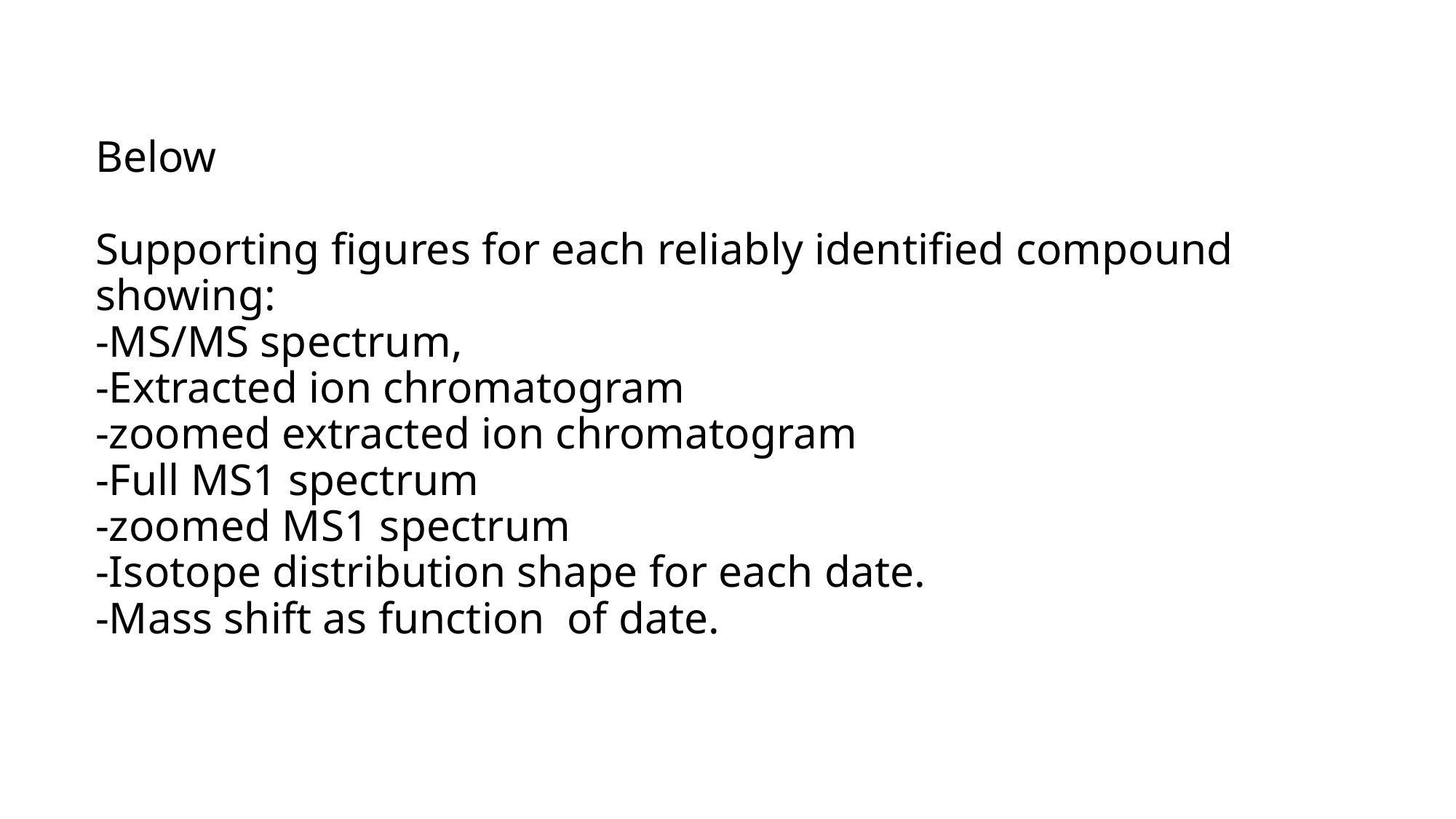

# Below Supporting figures for each reliably identified compound showing: -MS/MS spectrum, -Extracted ion chromatogram -zoomed extracted ion chromatogram -Full MS1 spectrum -zoomed MS1 spectrum -Isotope distribution shape for each date. -Mass shift as function of date.

## Slide 6
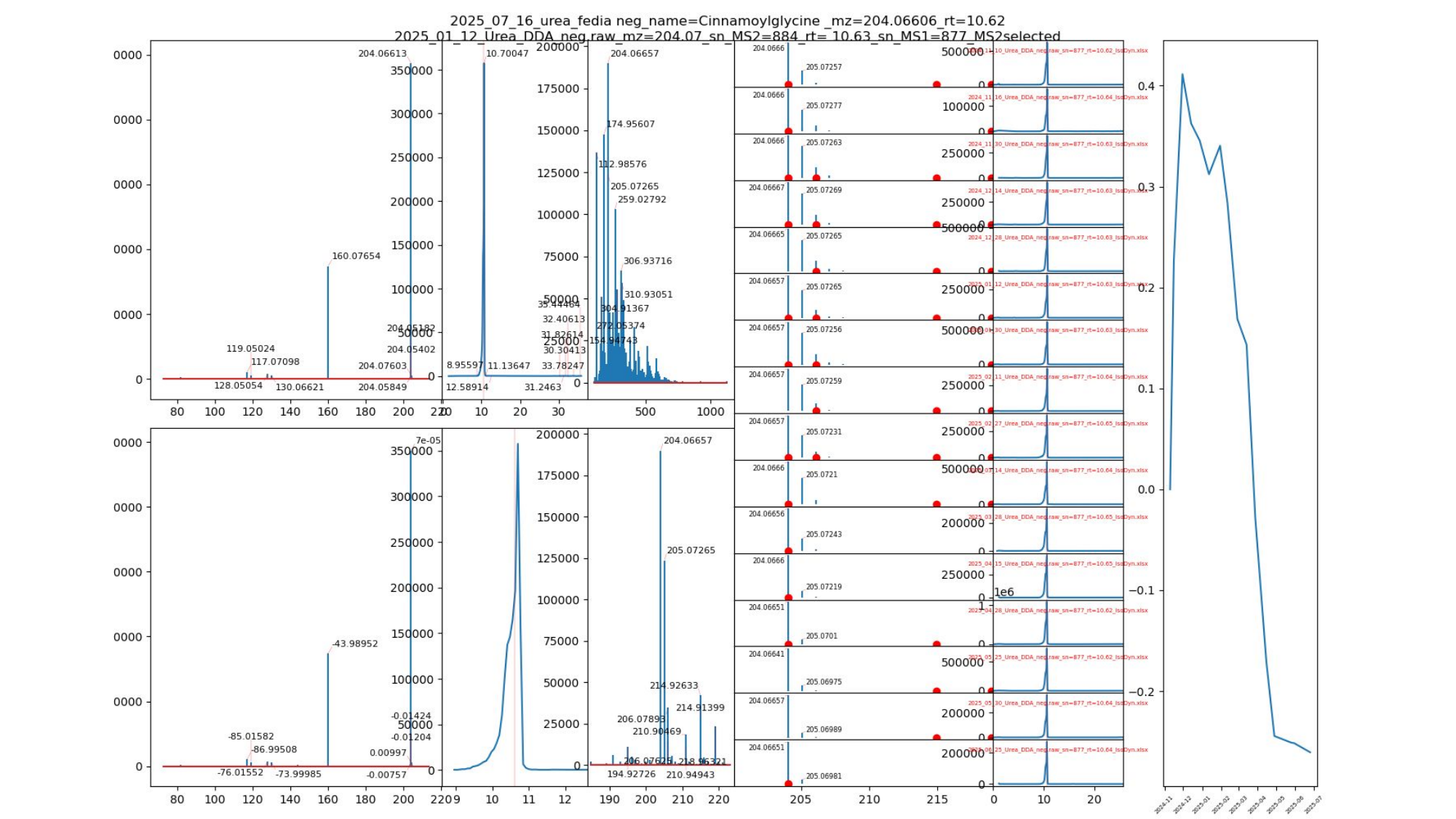

## Slide 7
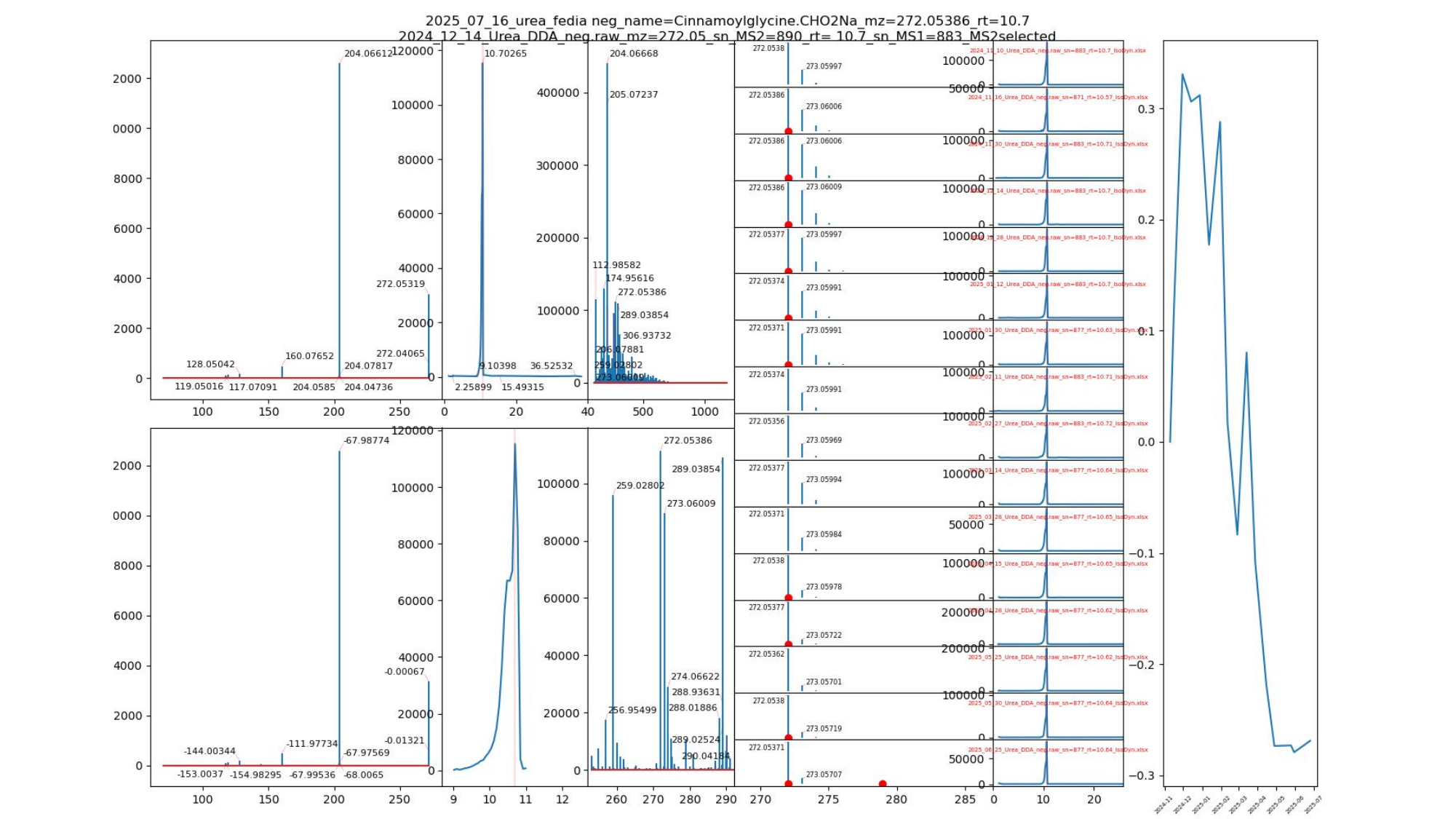

## Slide 8
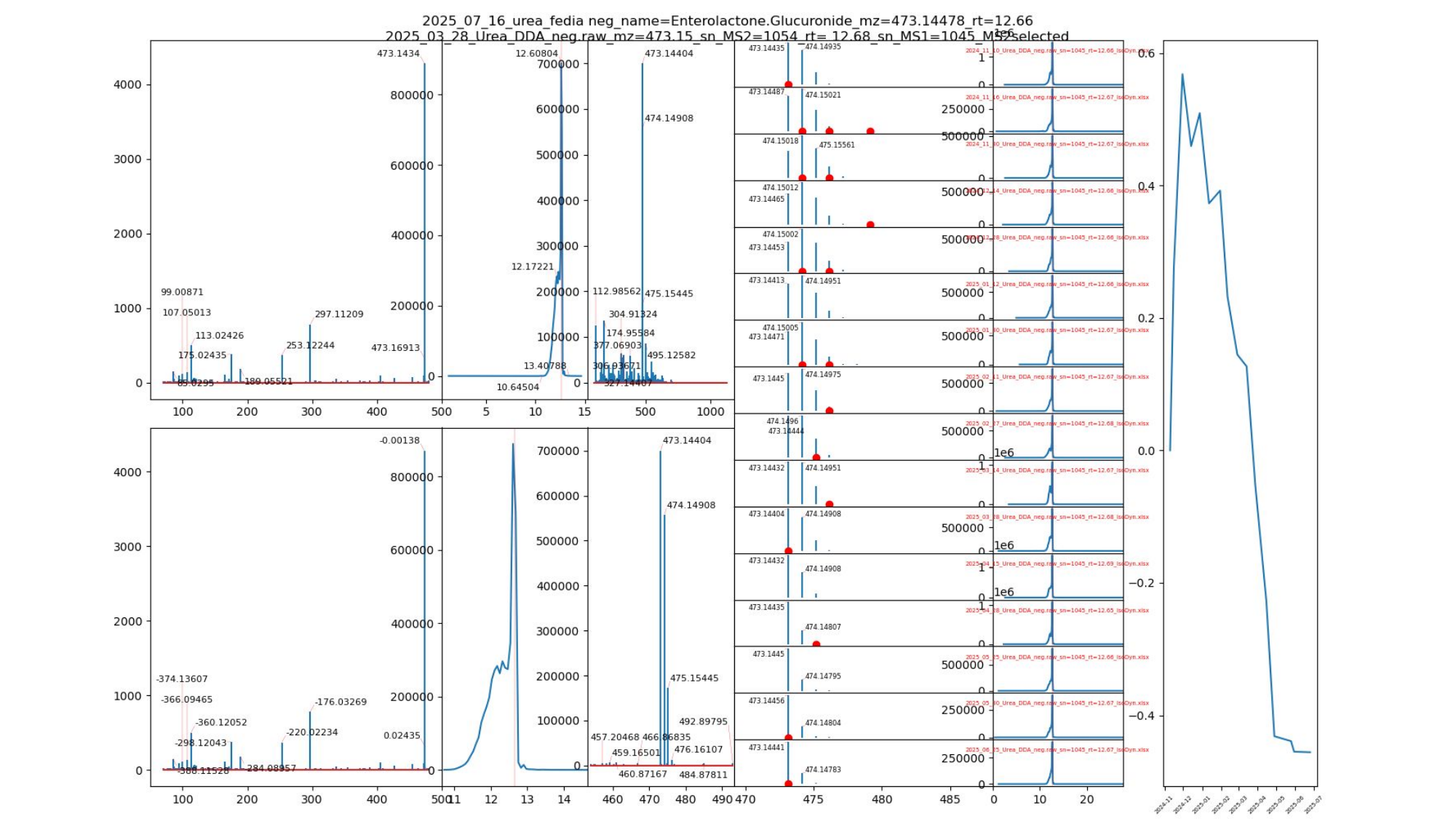

## Slide 9
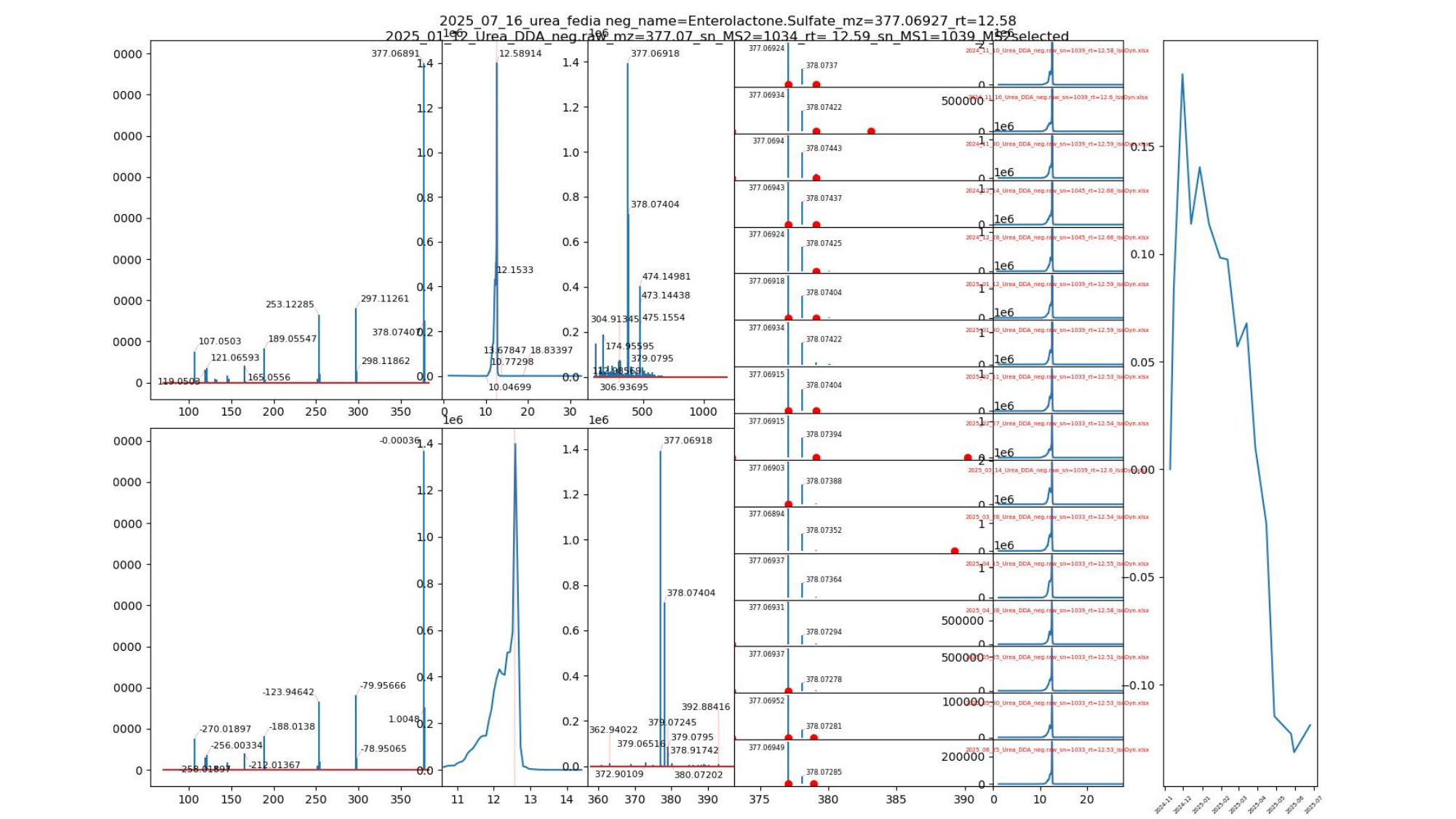

## Slide 10
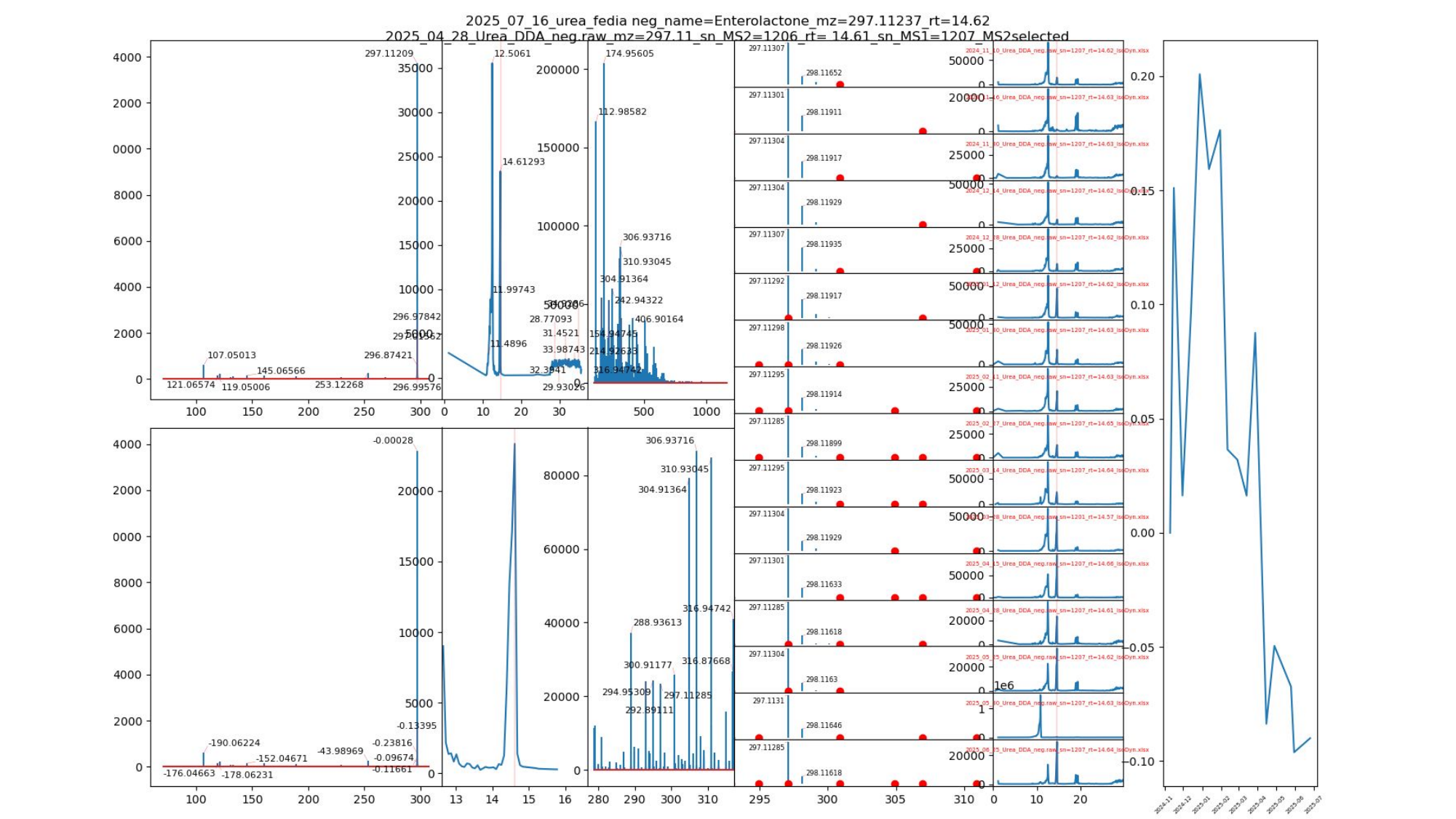

## Slide 11
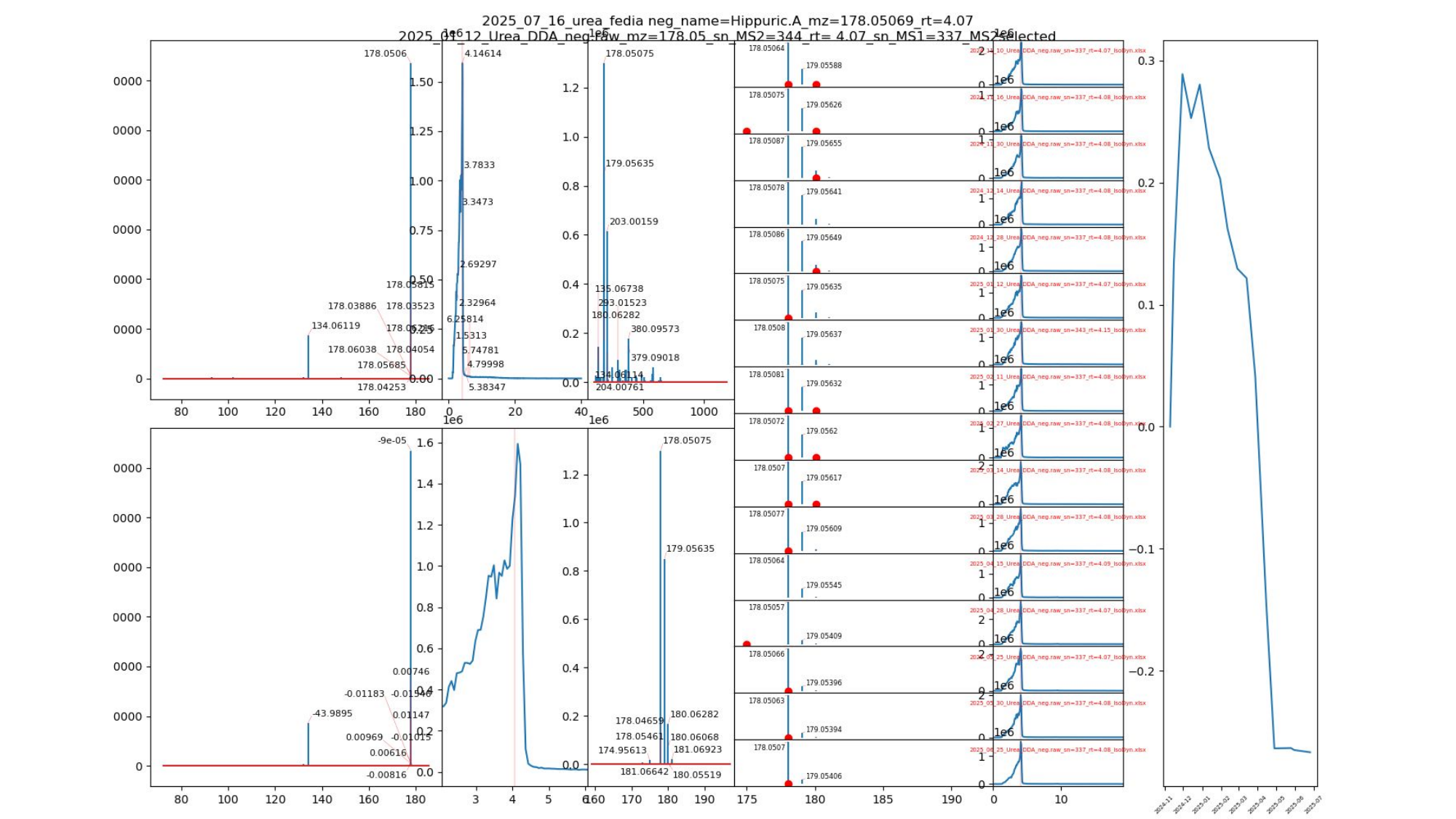

## Slide 12
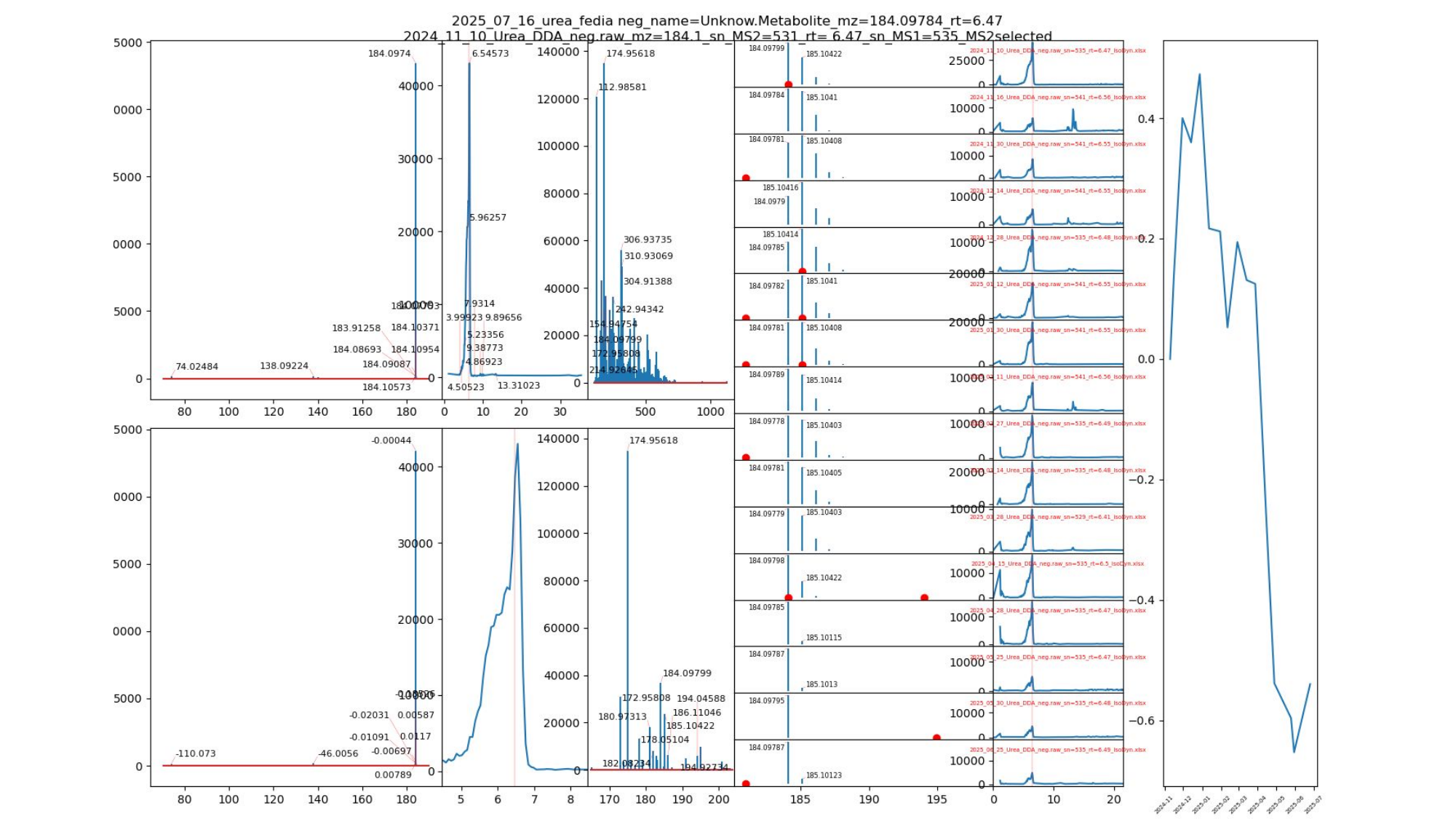

## Slide 13
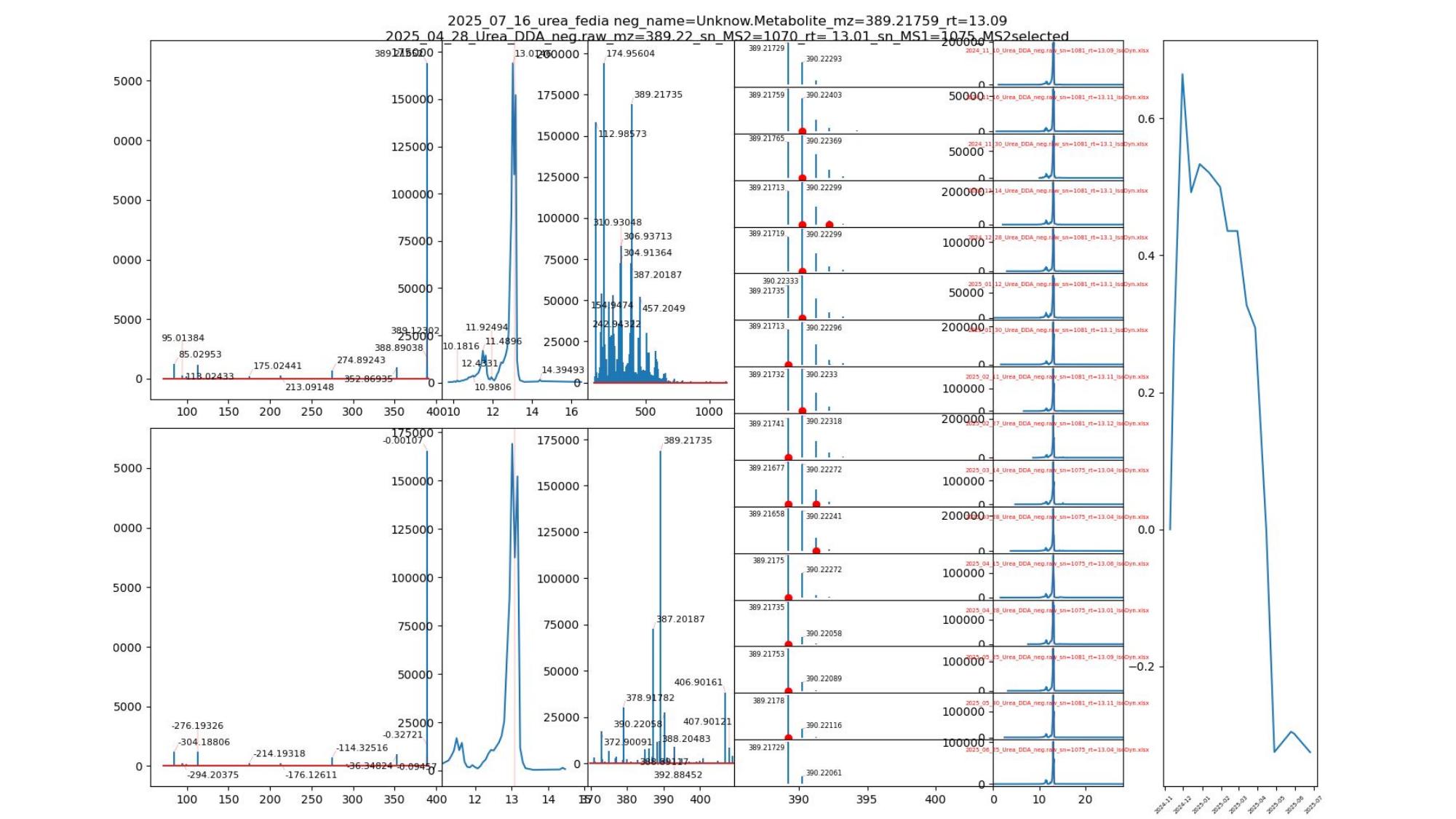

## Slide 14
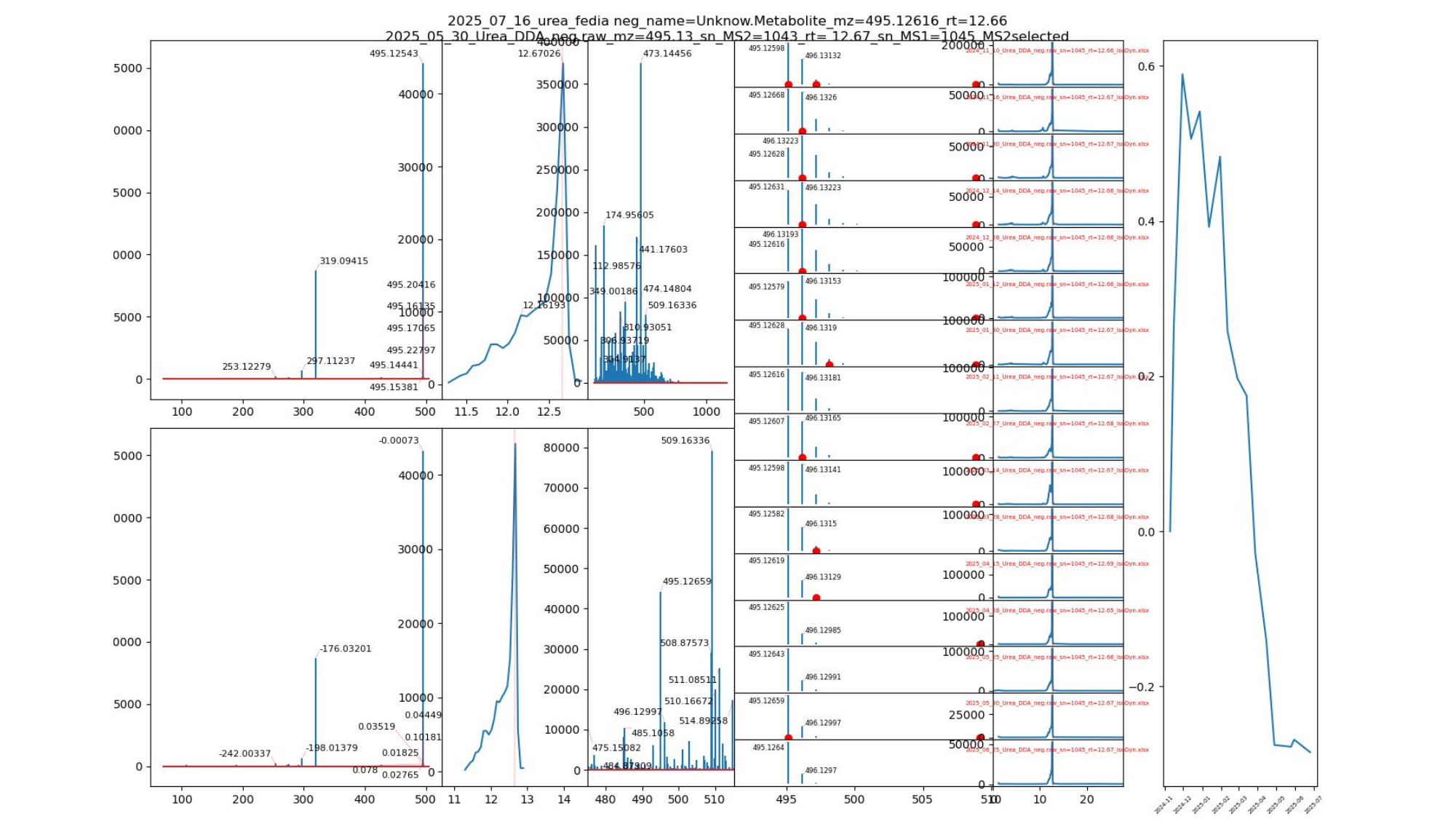

## Slide 15
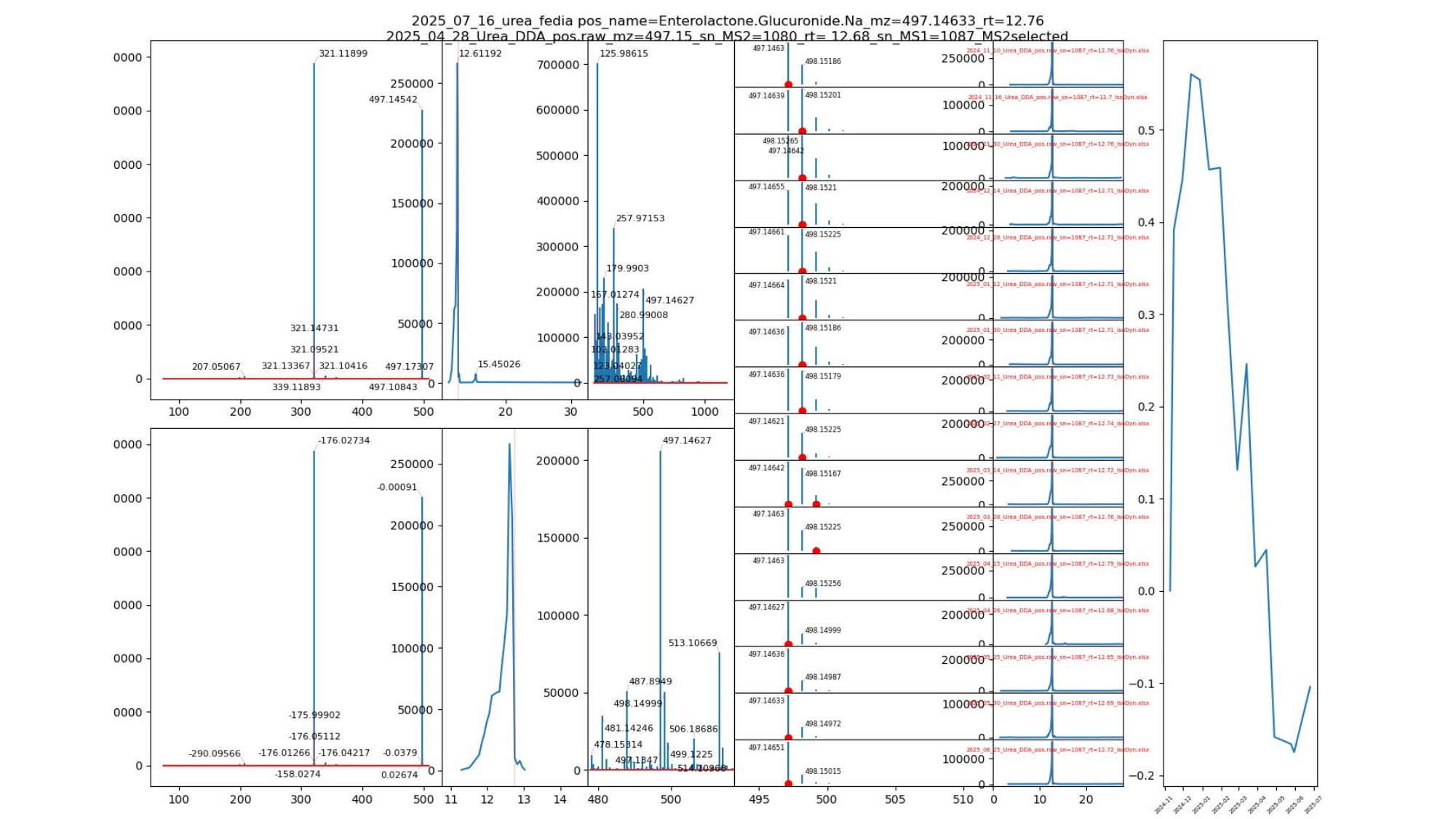

## Slide 16
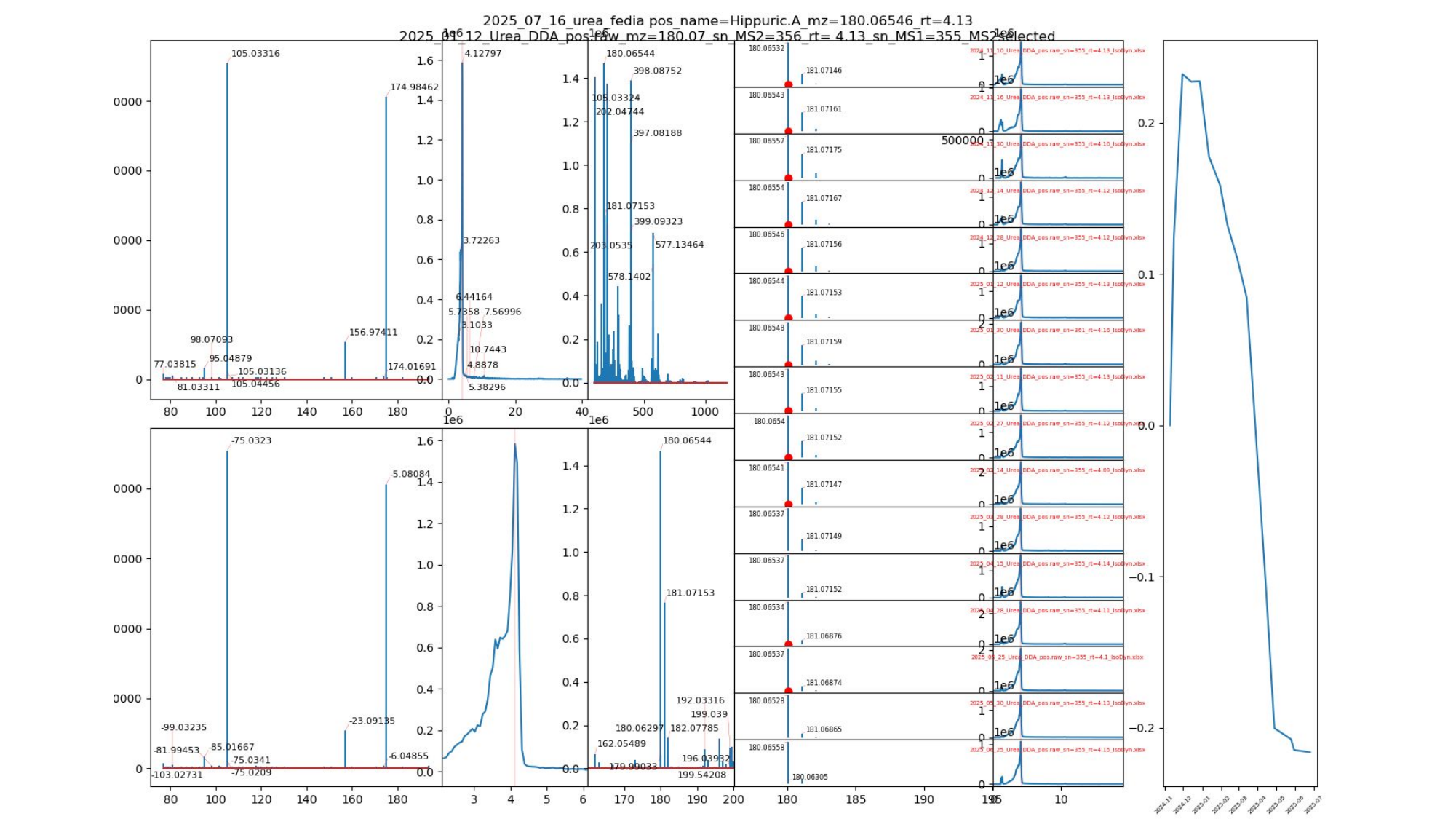

## Slide 17
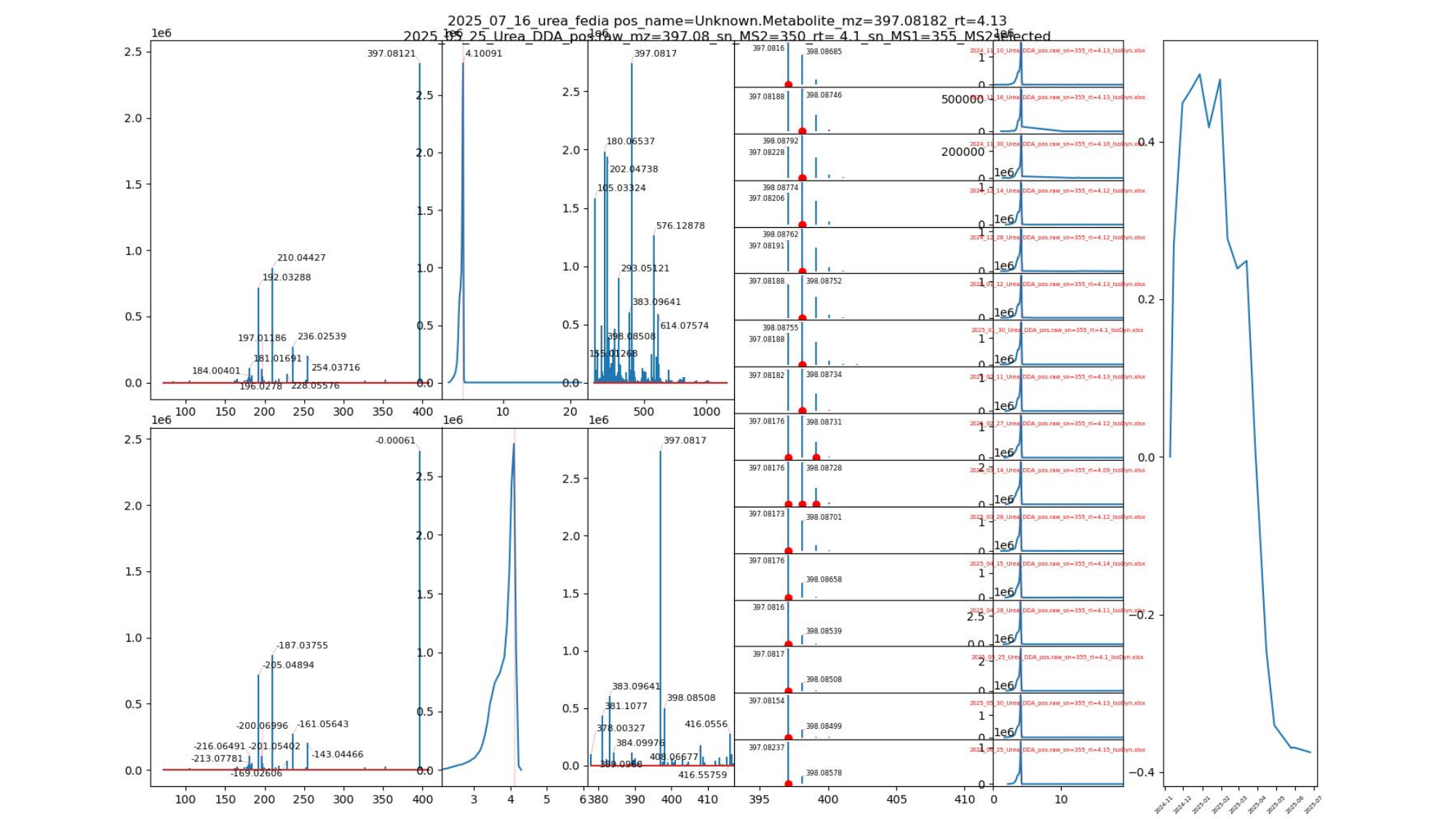

## Slide 18
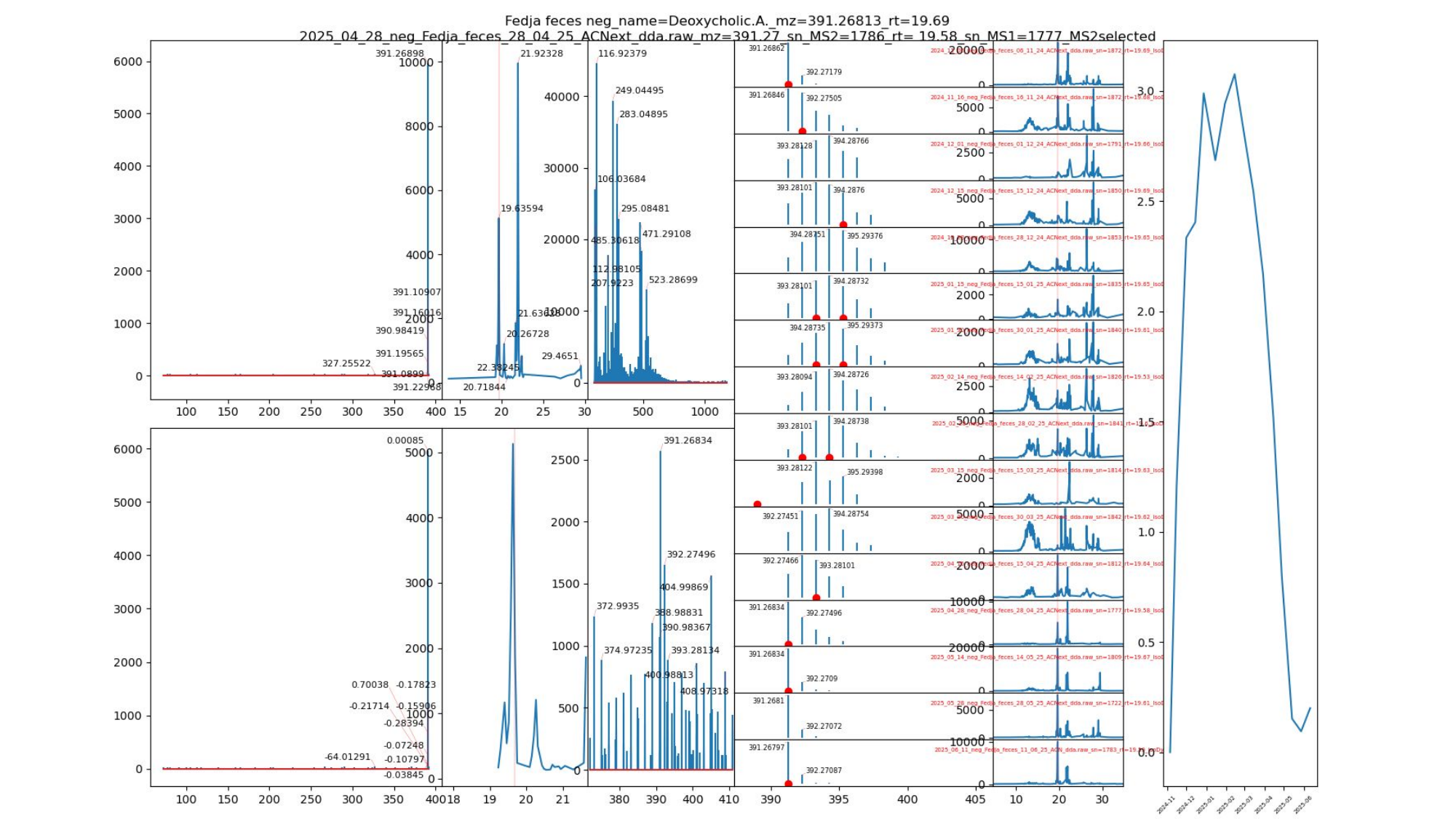

## Slide 19
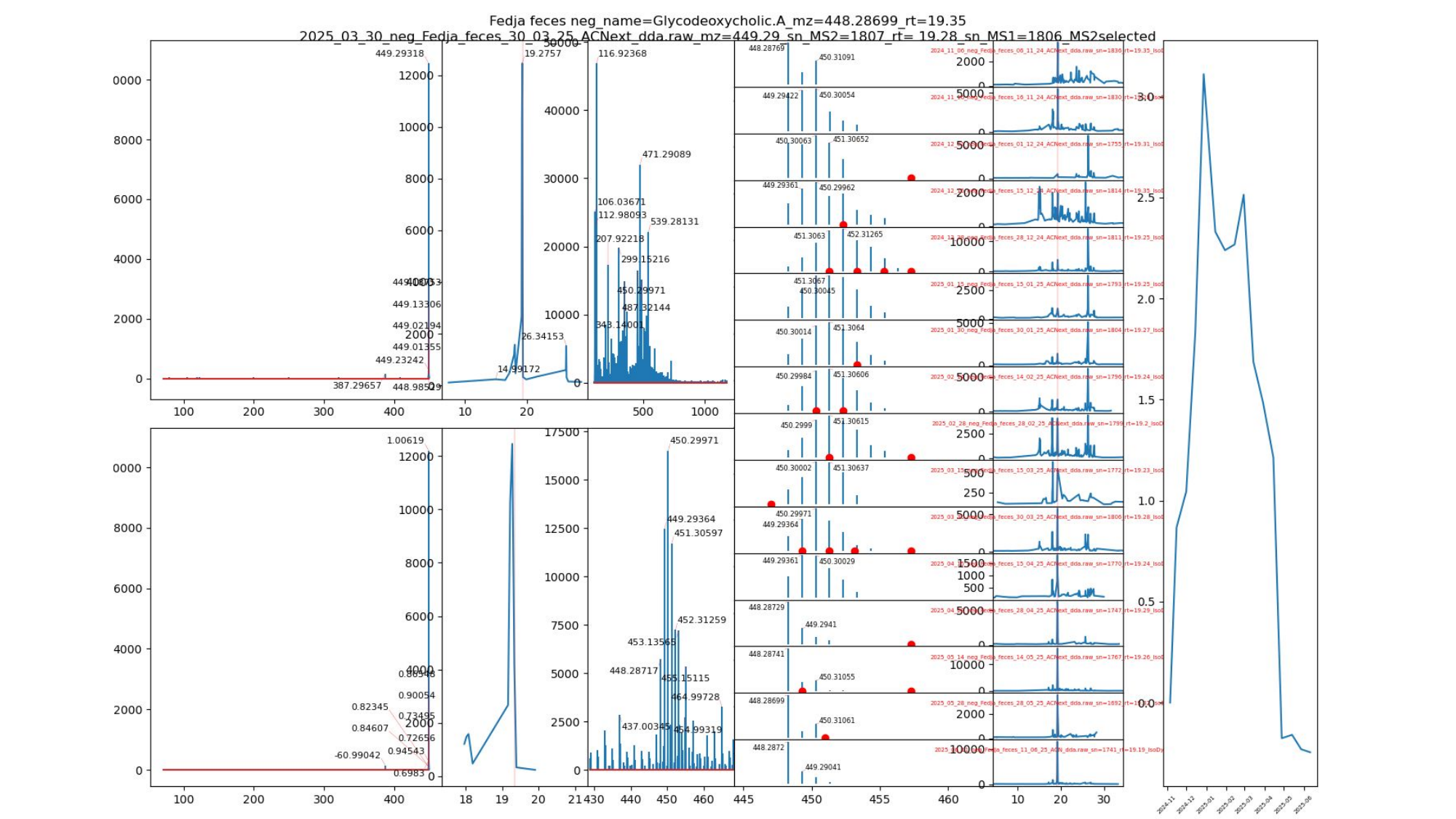

## Slide 20
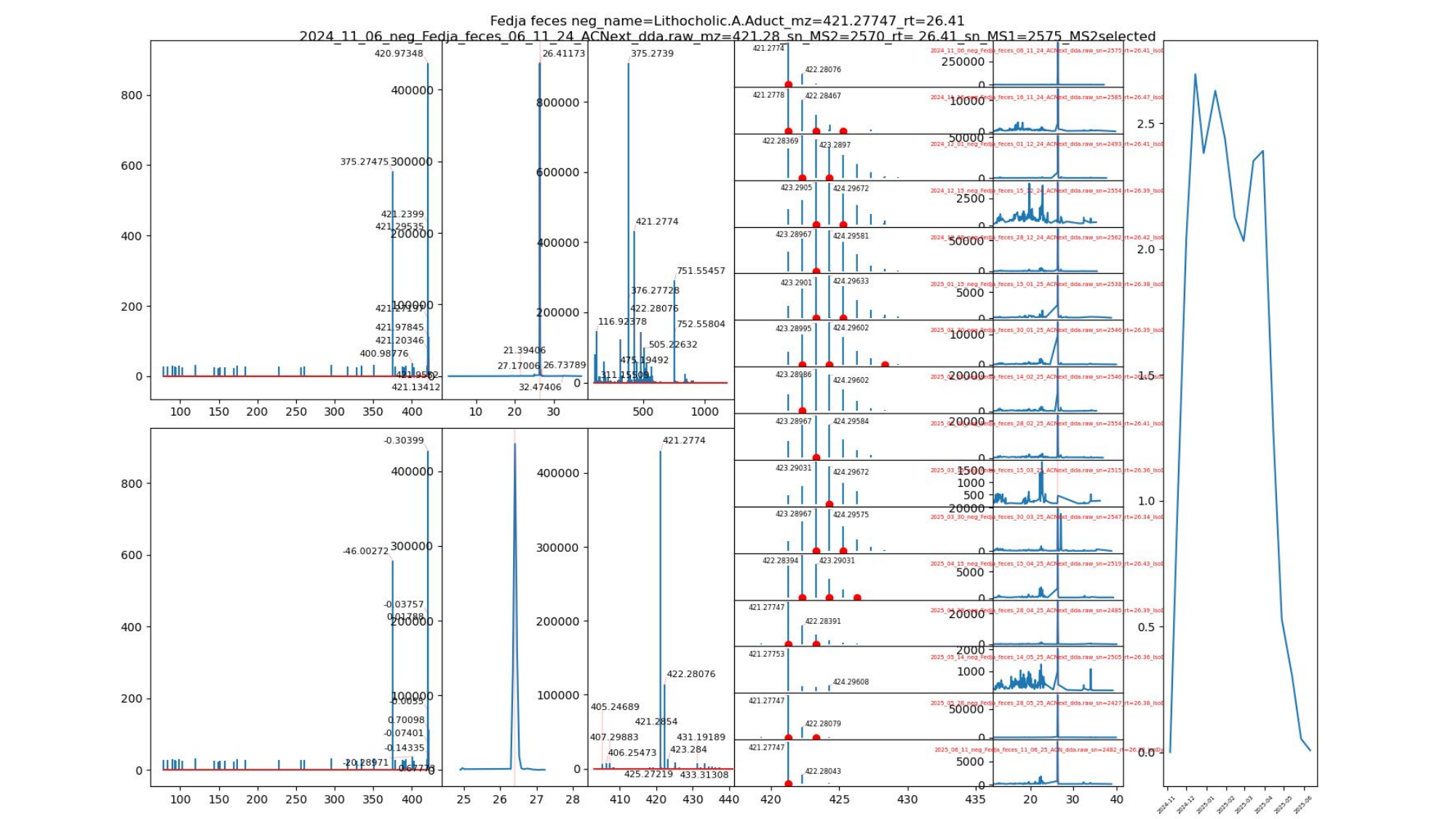

## Slide 21
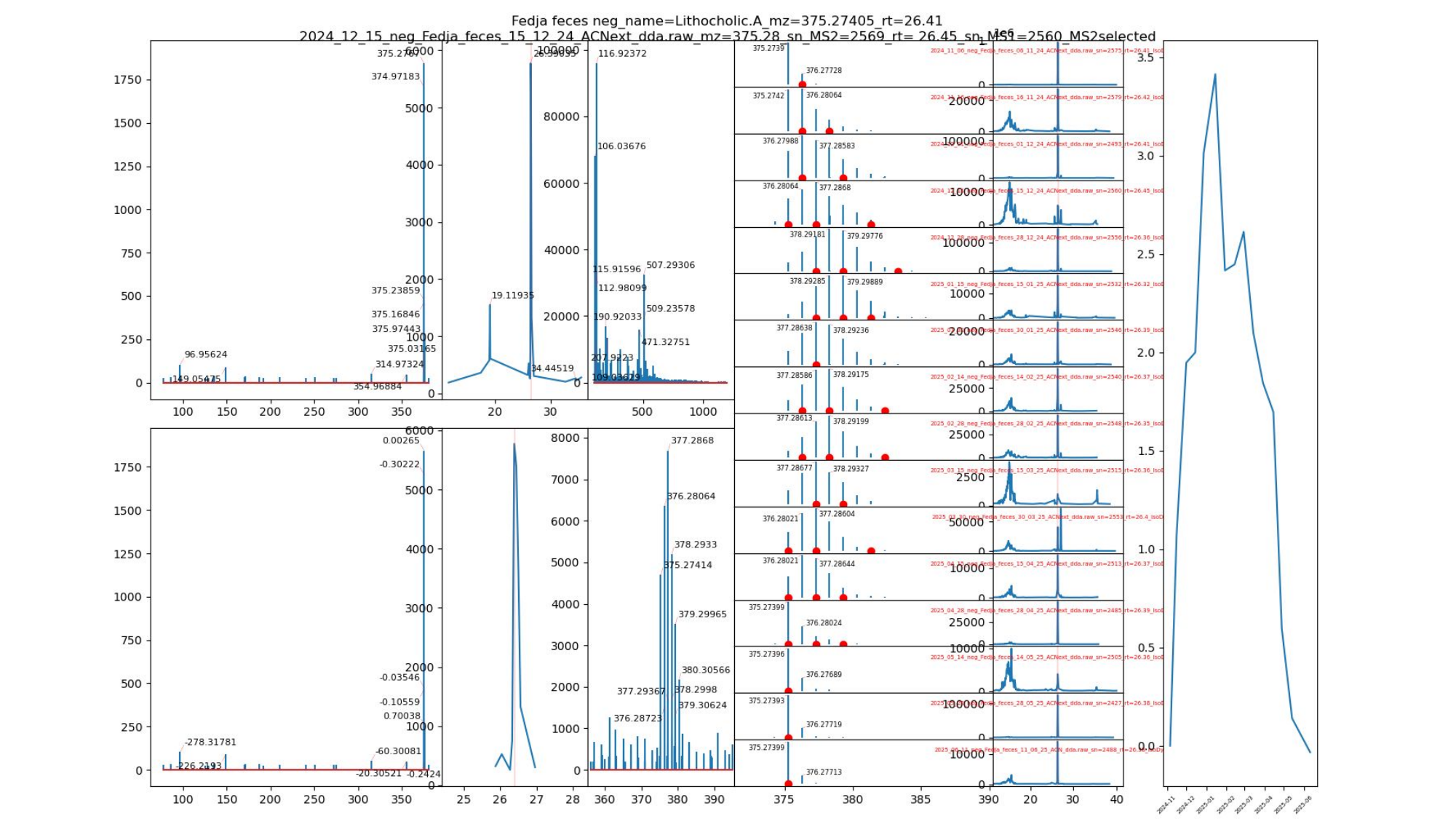

## Slide 22
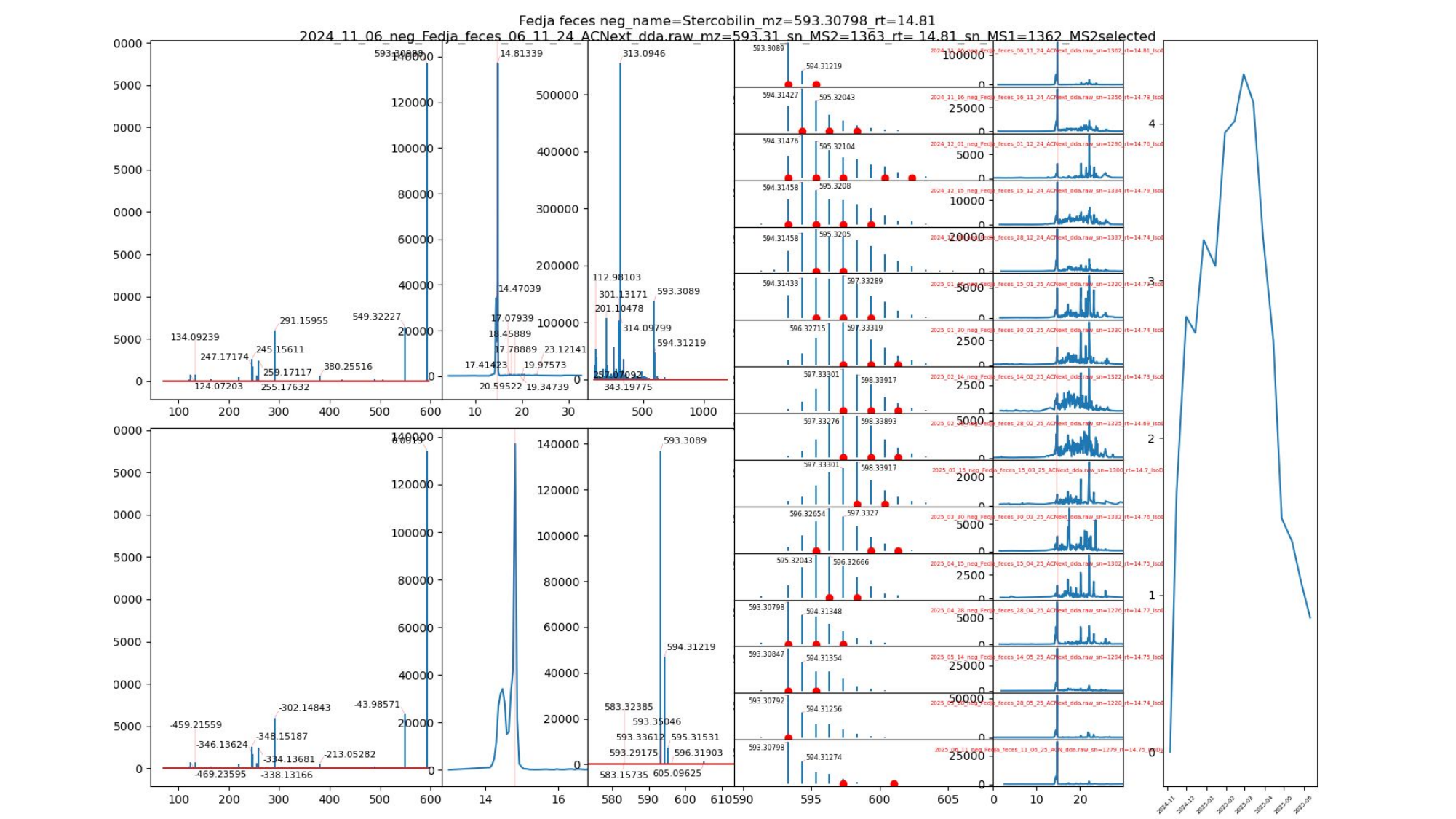

## Slide 23
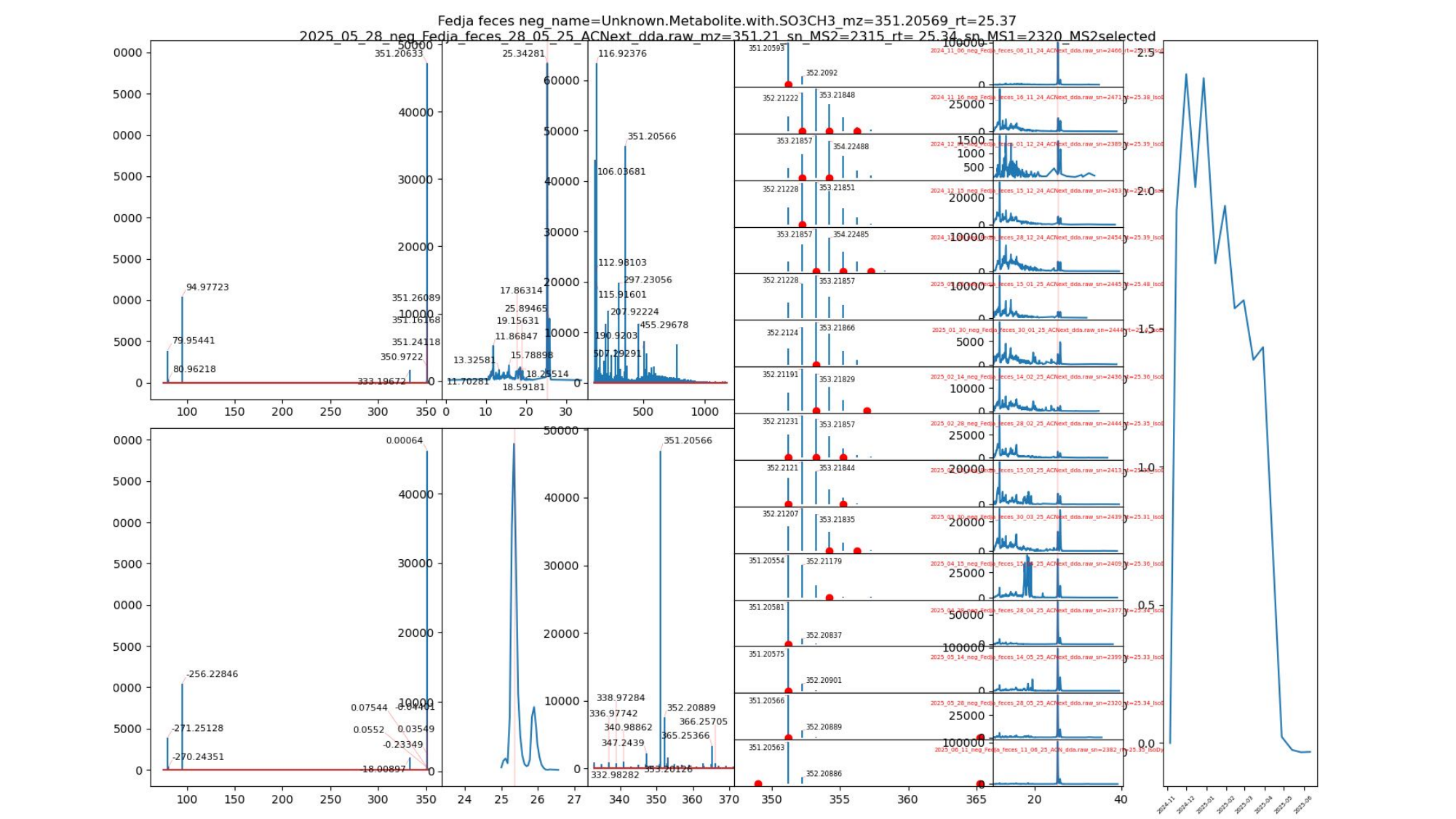

## Slide 24
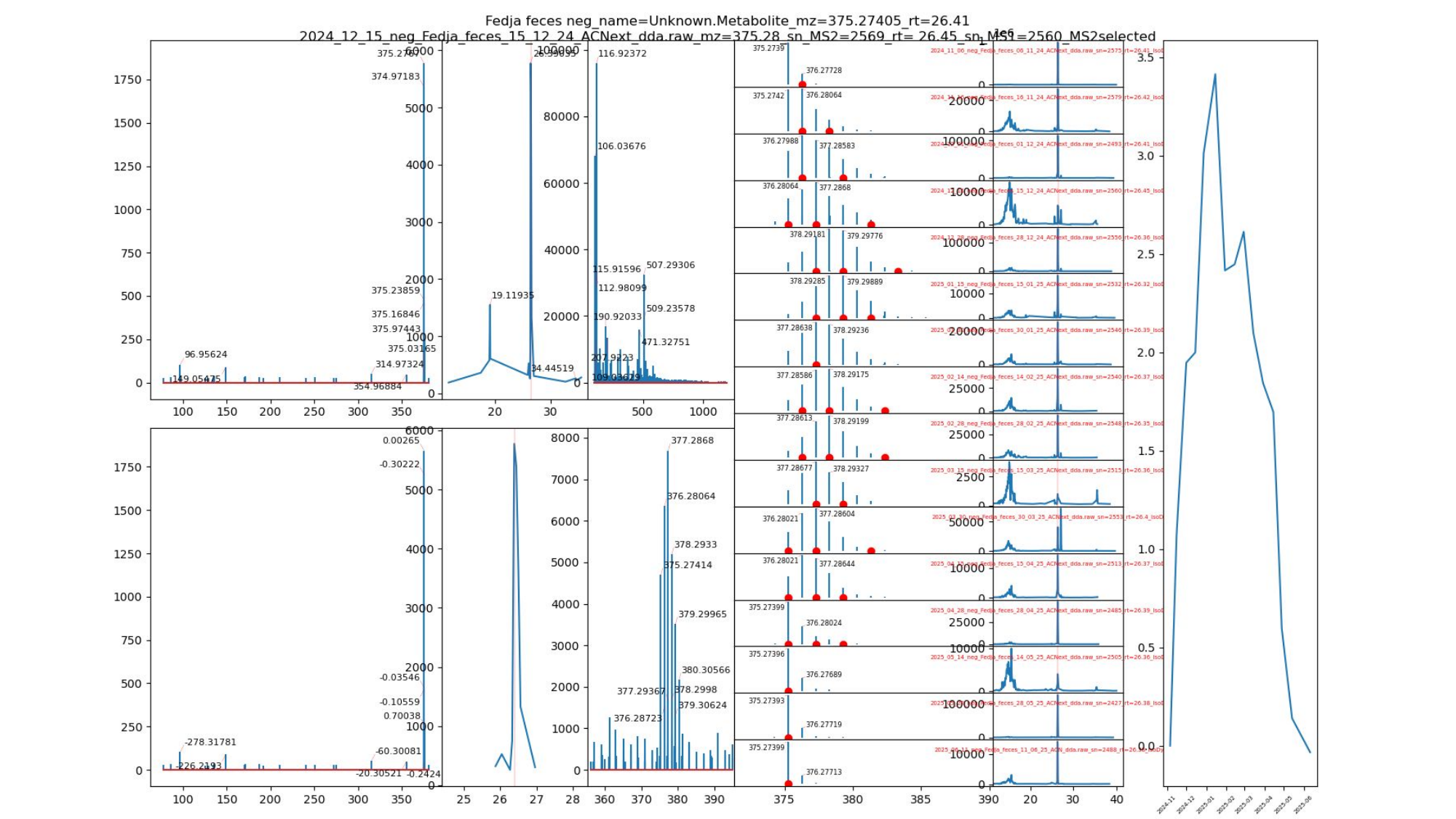

## Slide 25
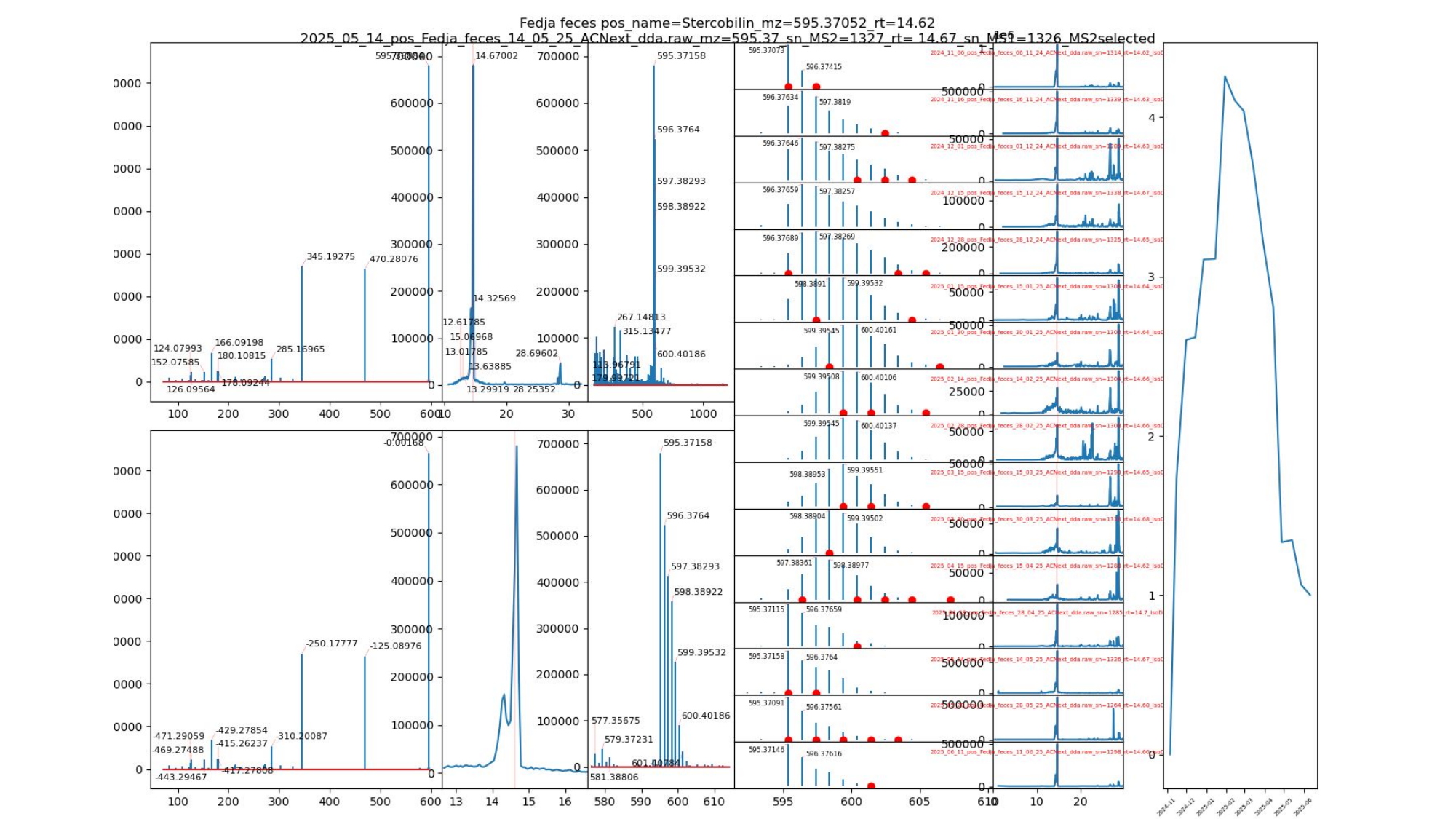

## Slide 26
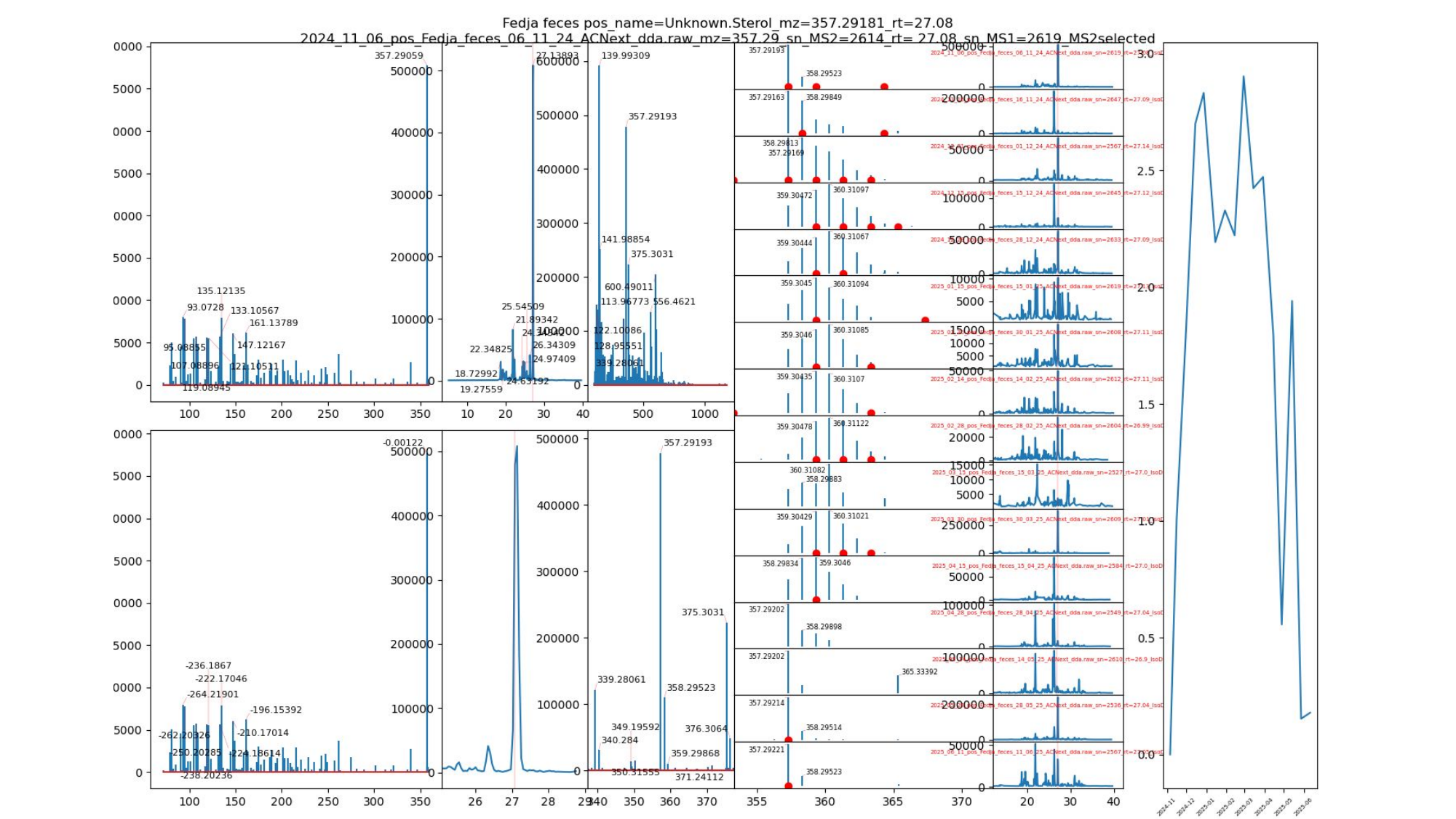

## Slide 27
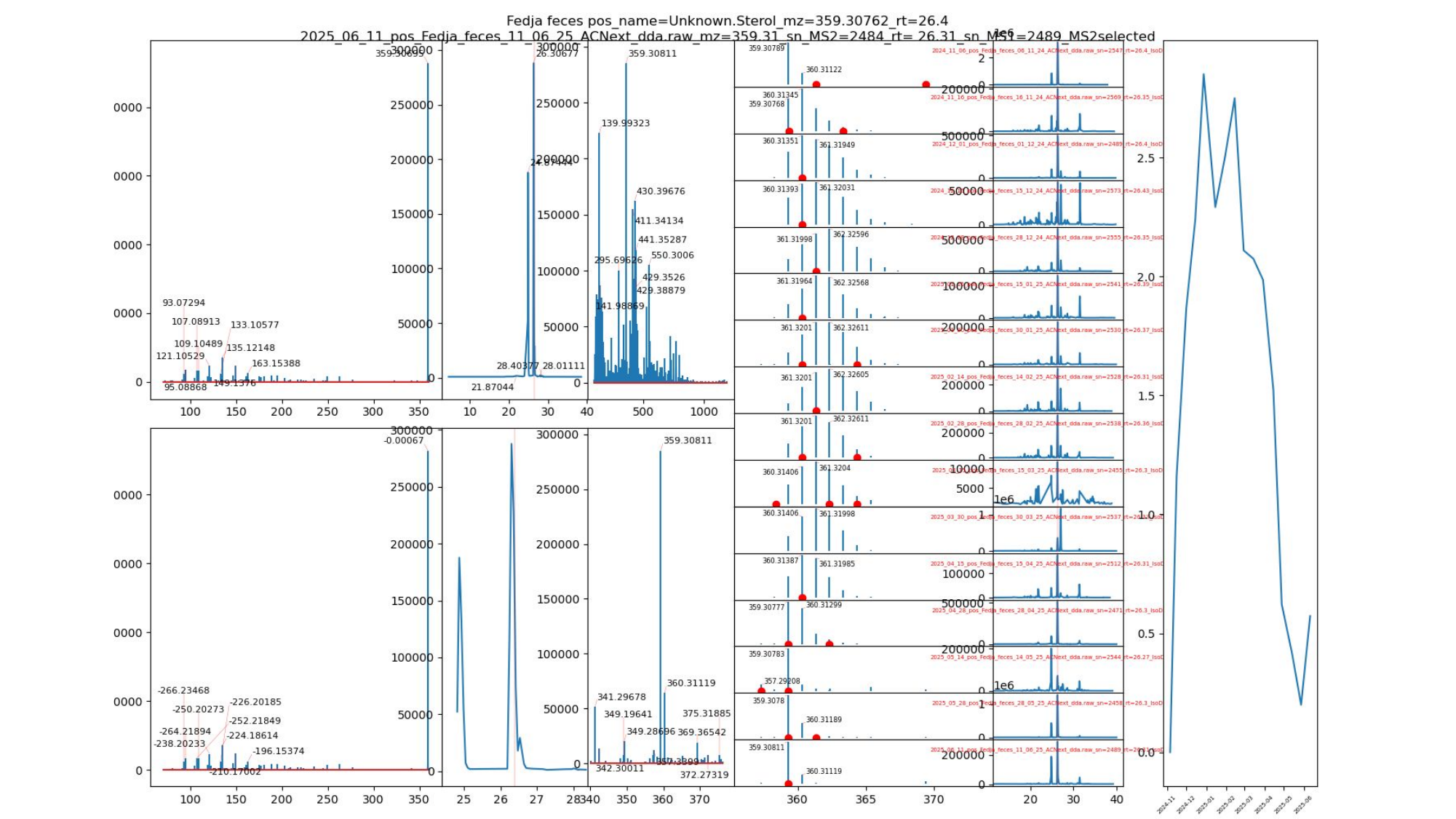

## Slide 28
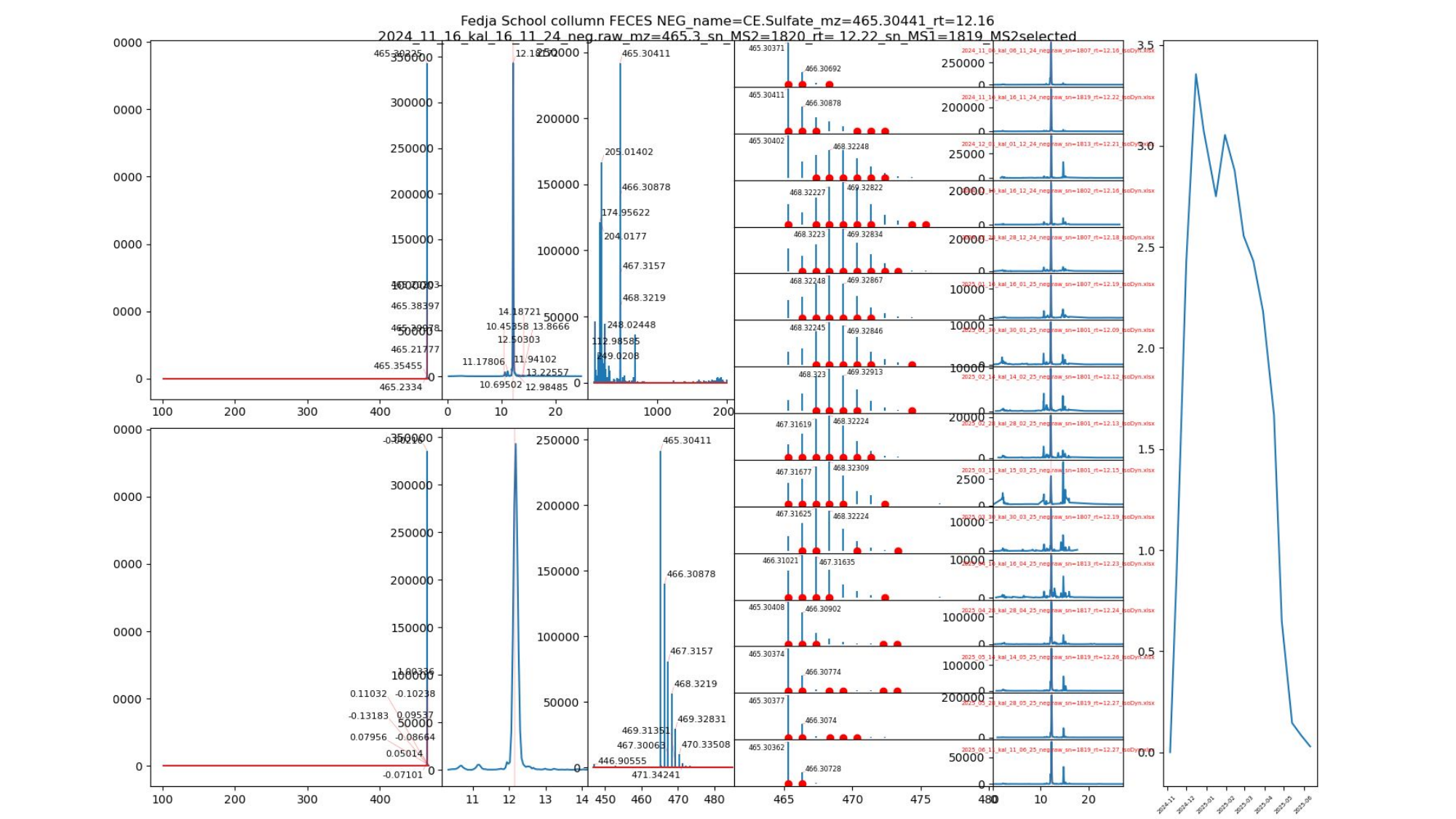

## Slide 29
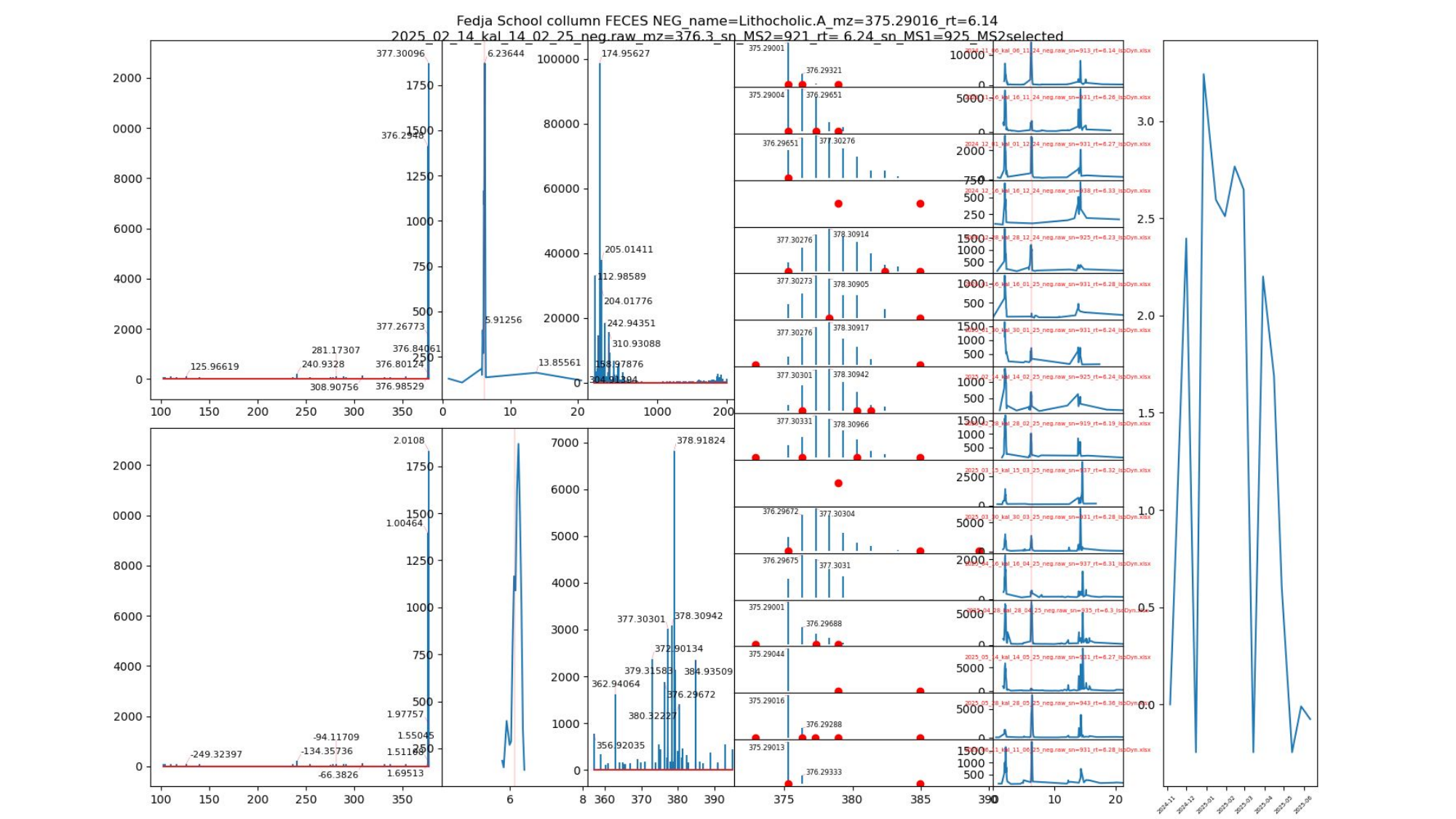

## Slide 30
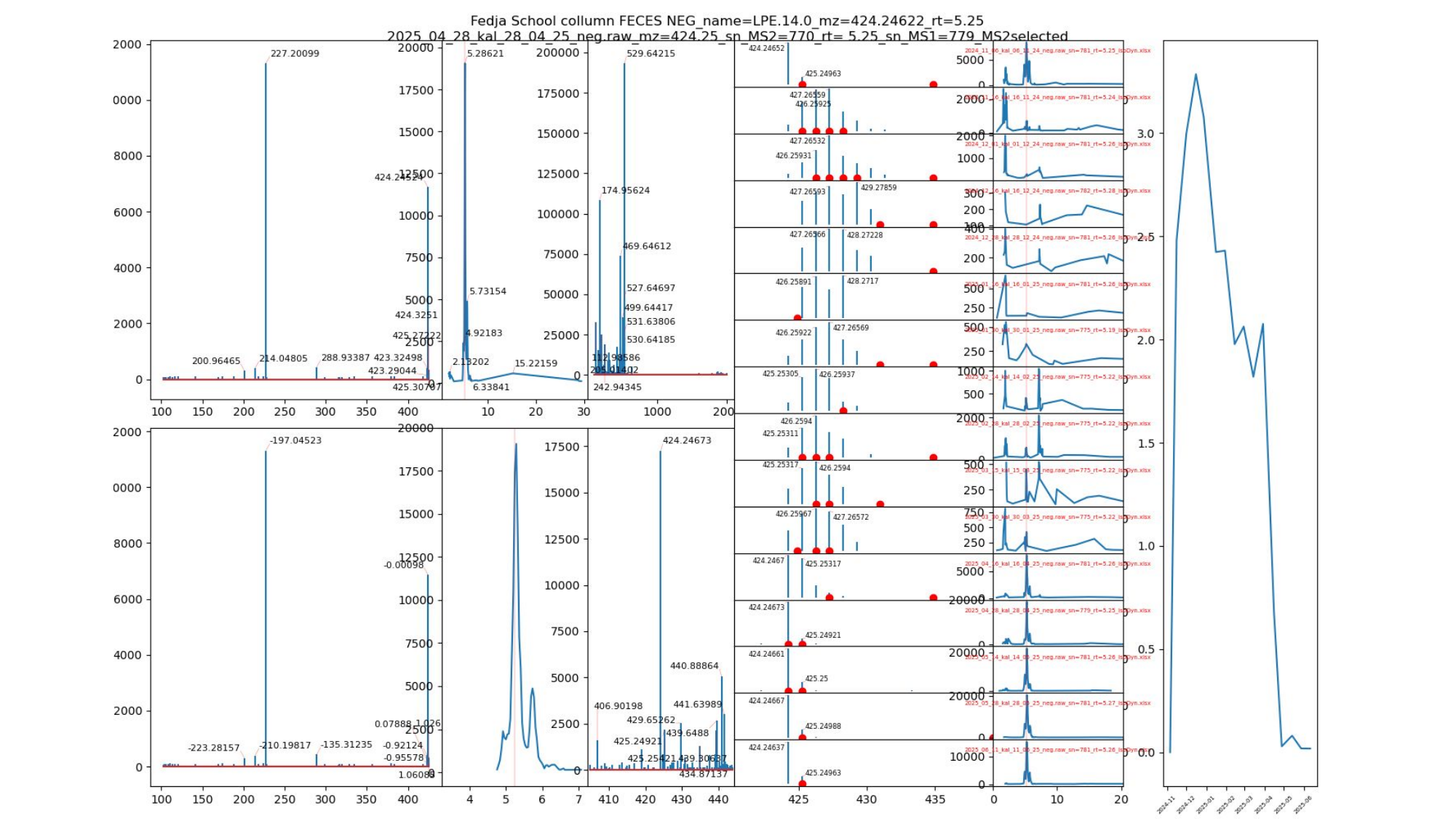

## Slide 31
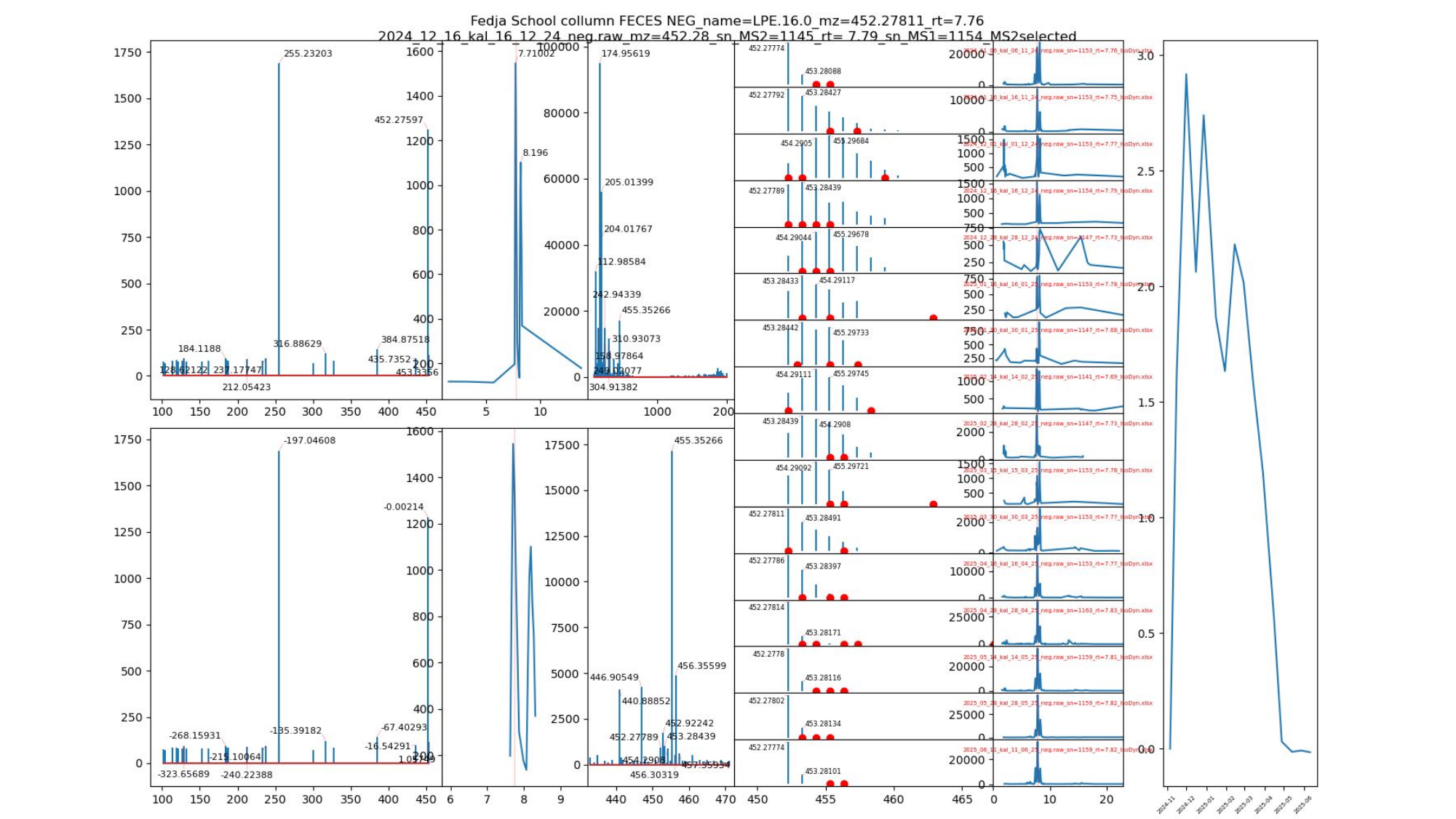

## Slide 32
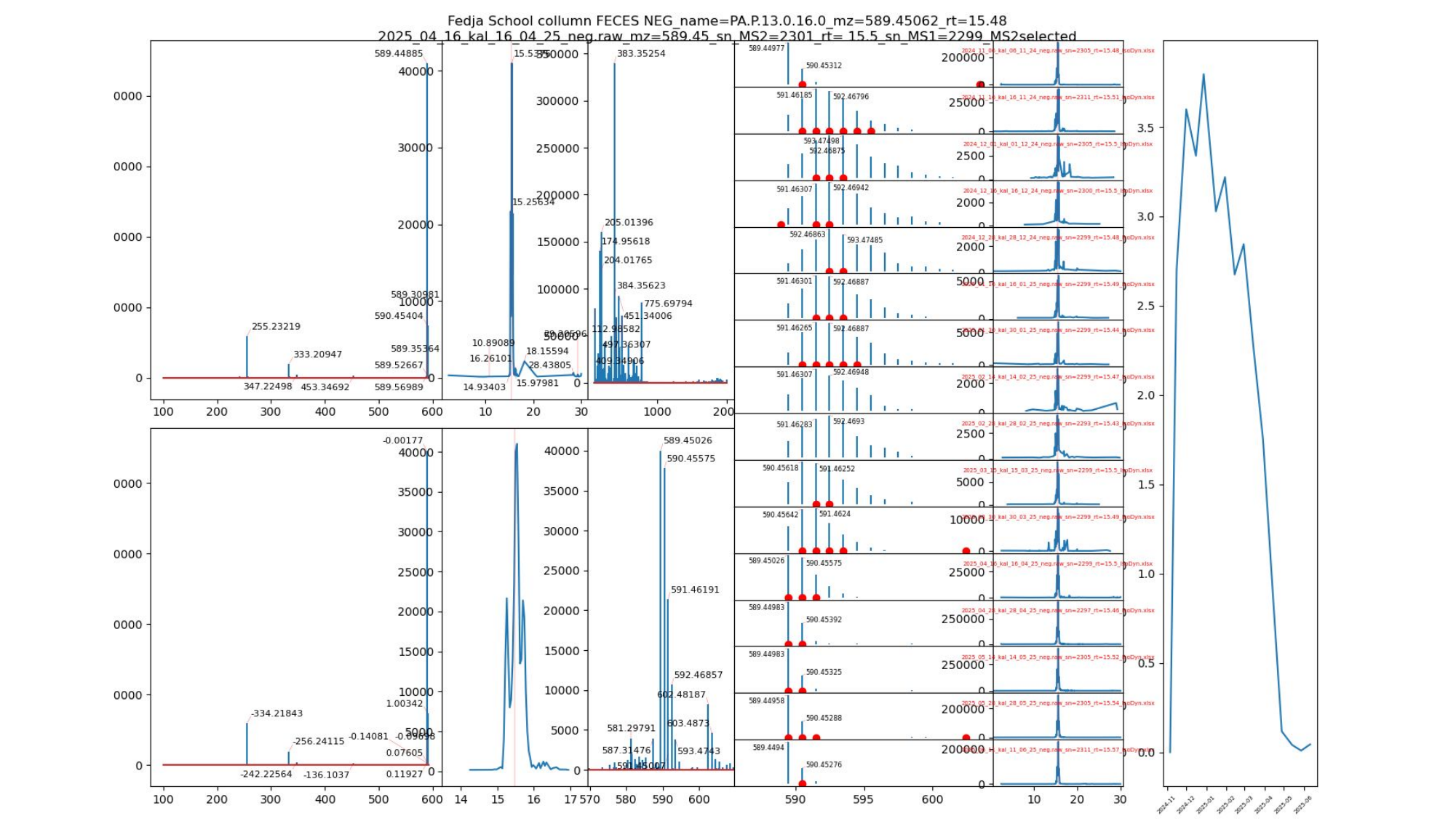

## Slide 33
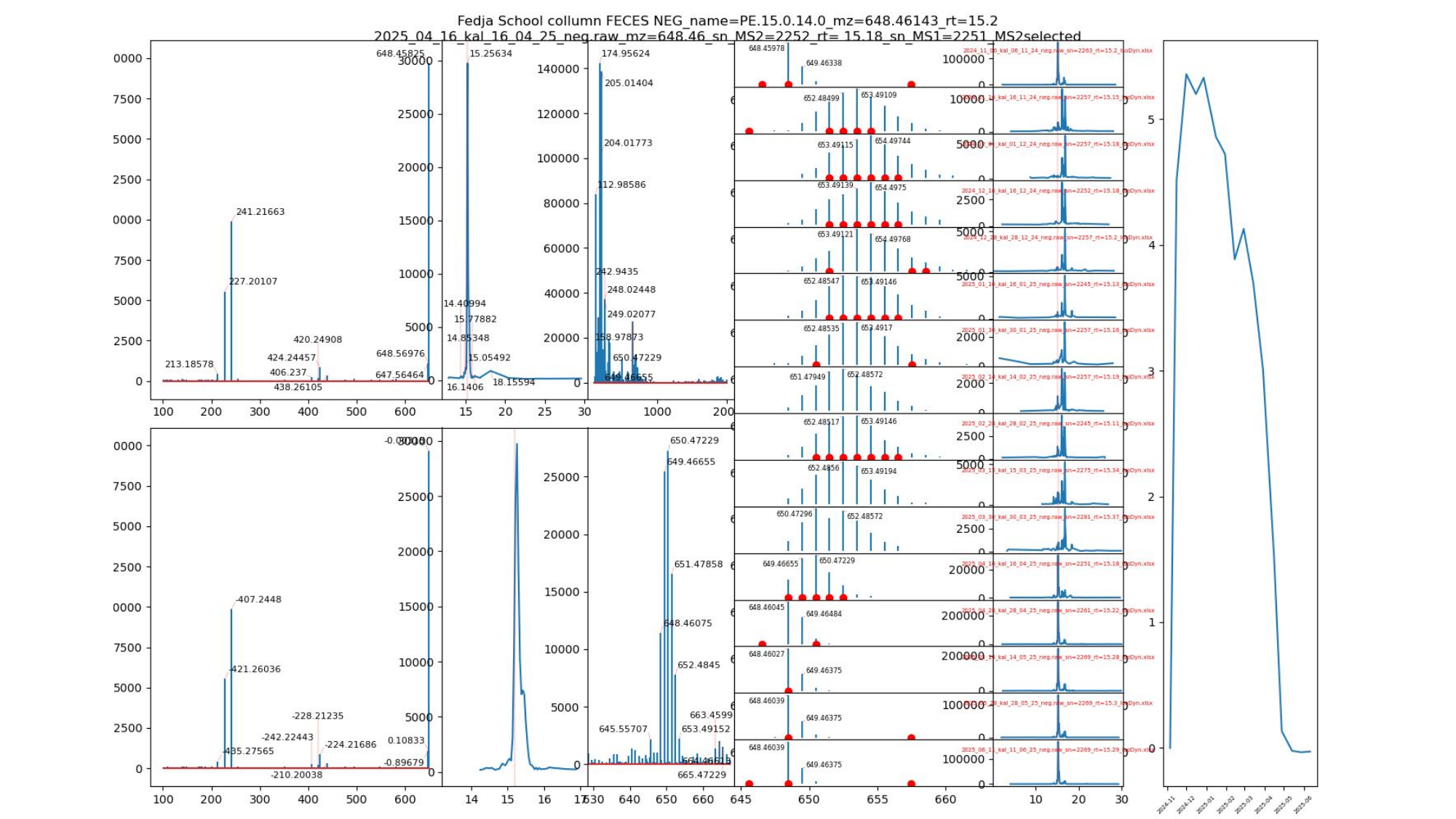

## Slide 34
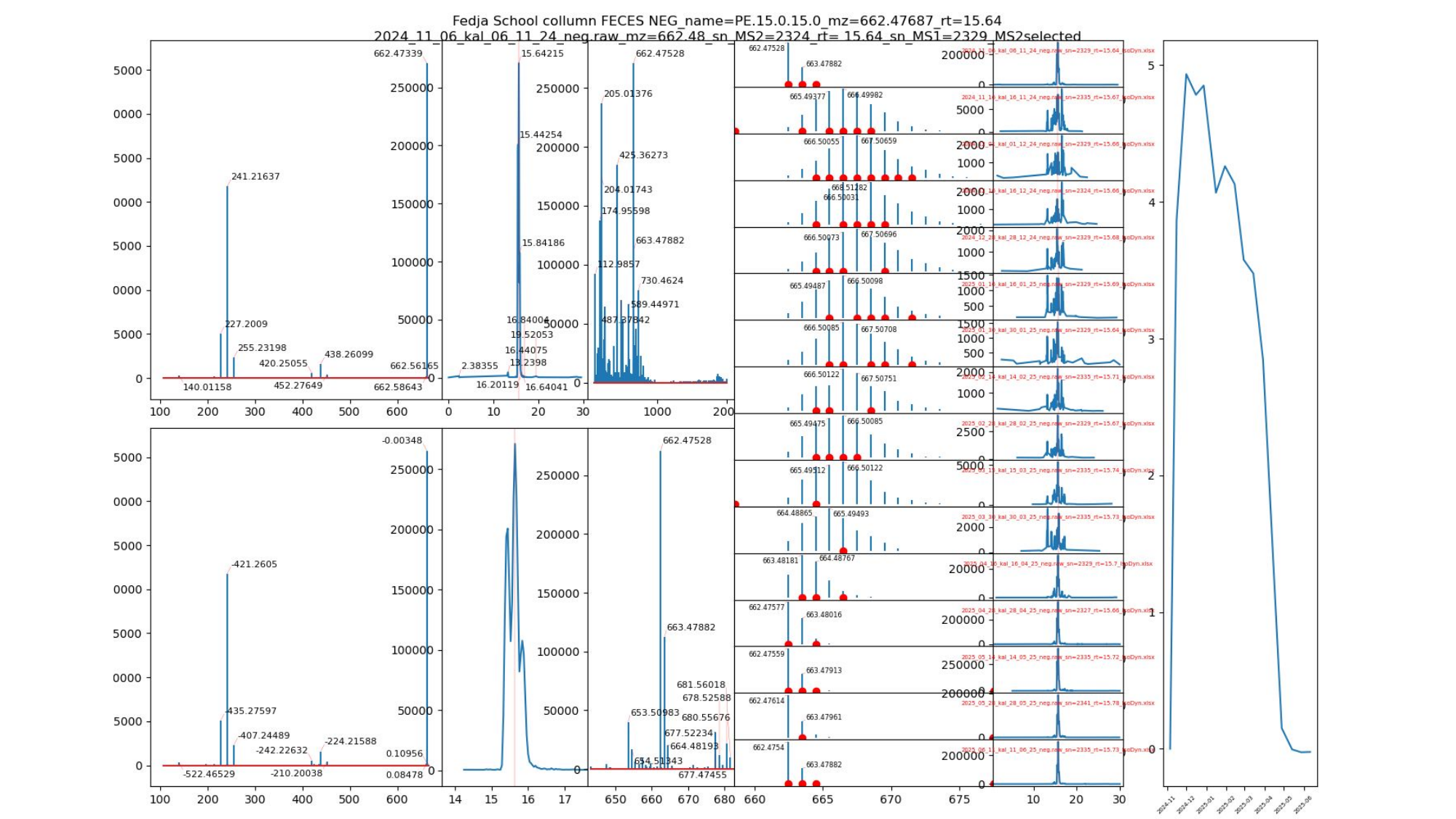

## Slide 35
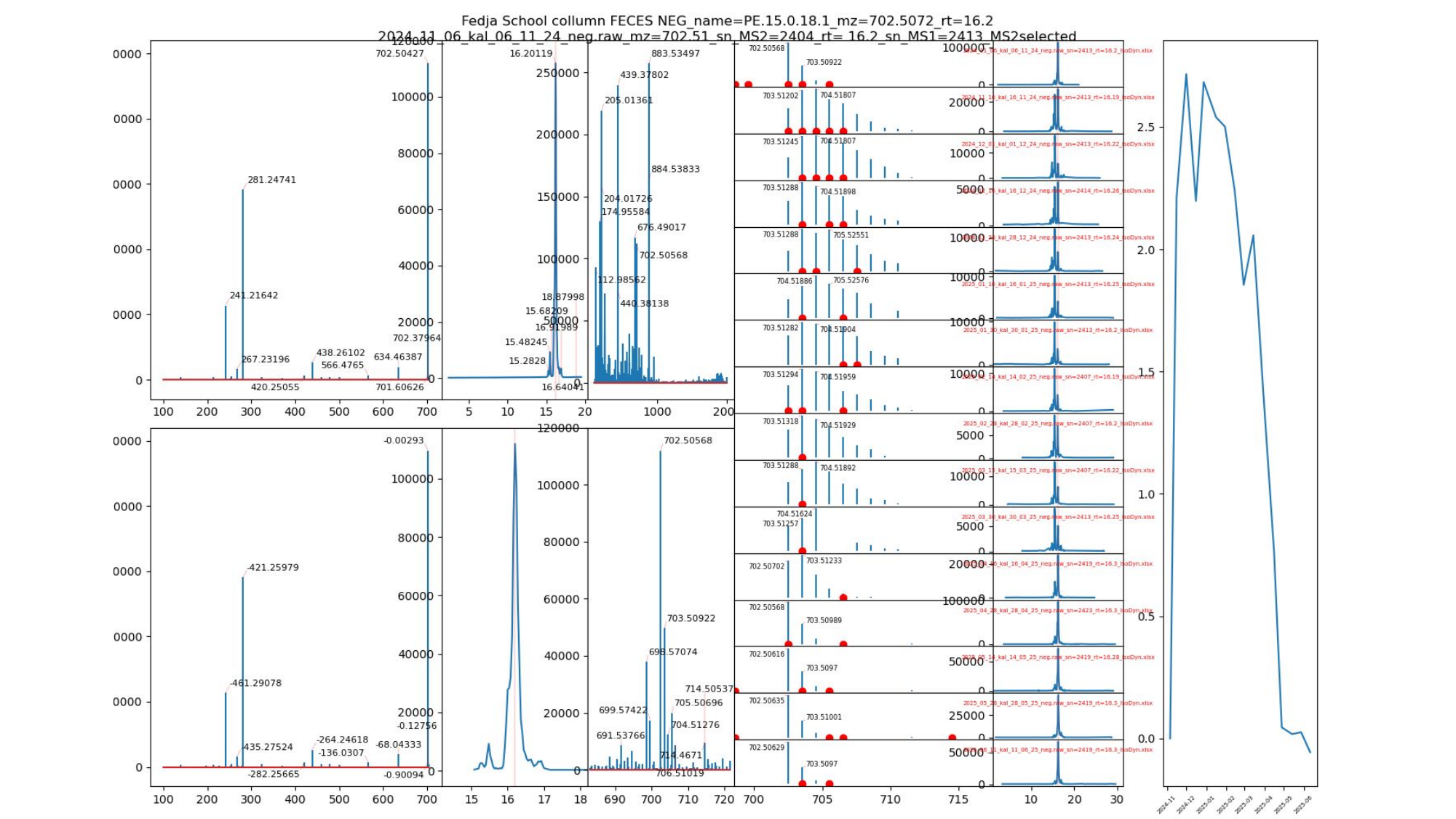

## Slide 36
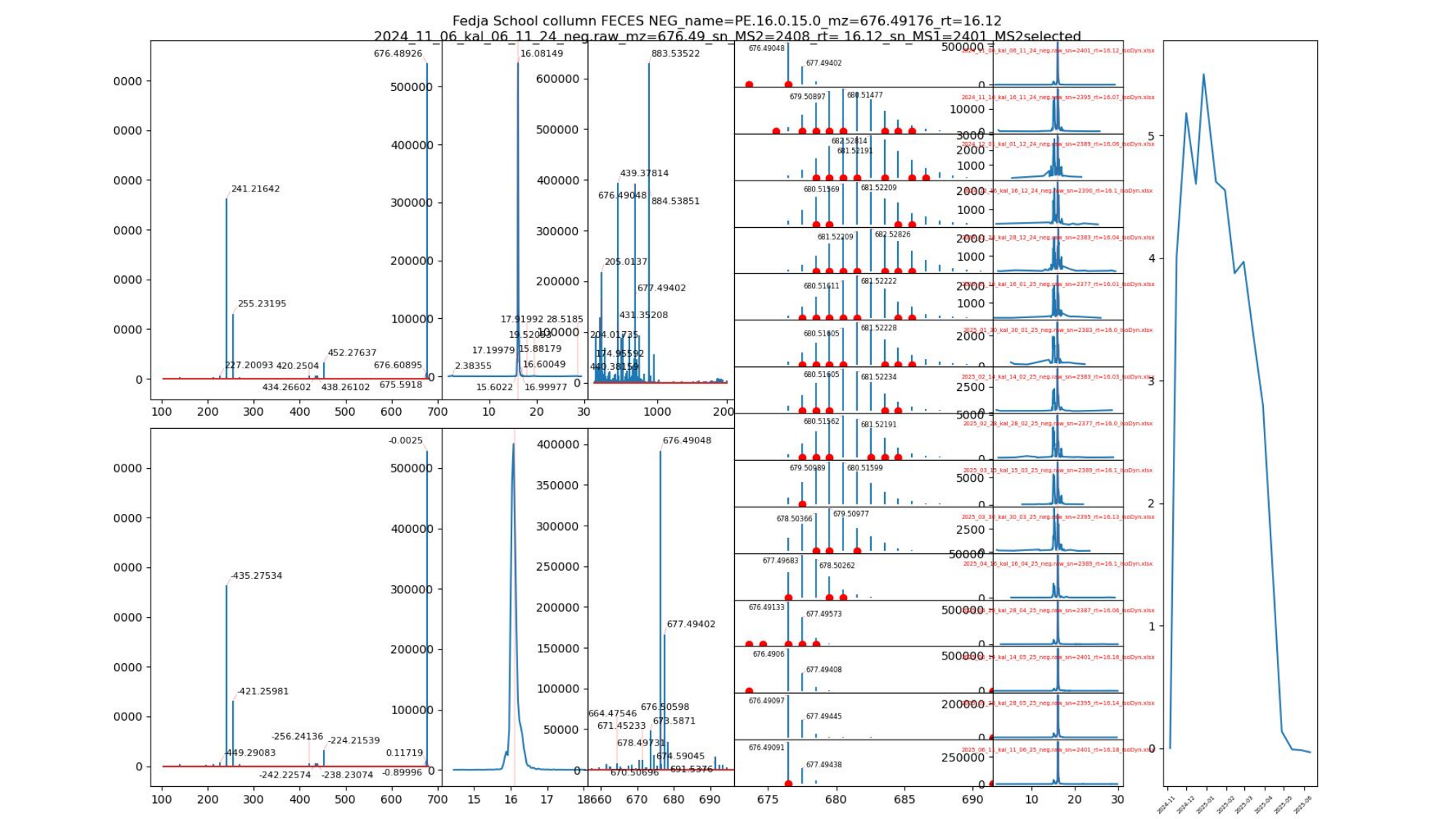

## Slide 37
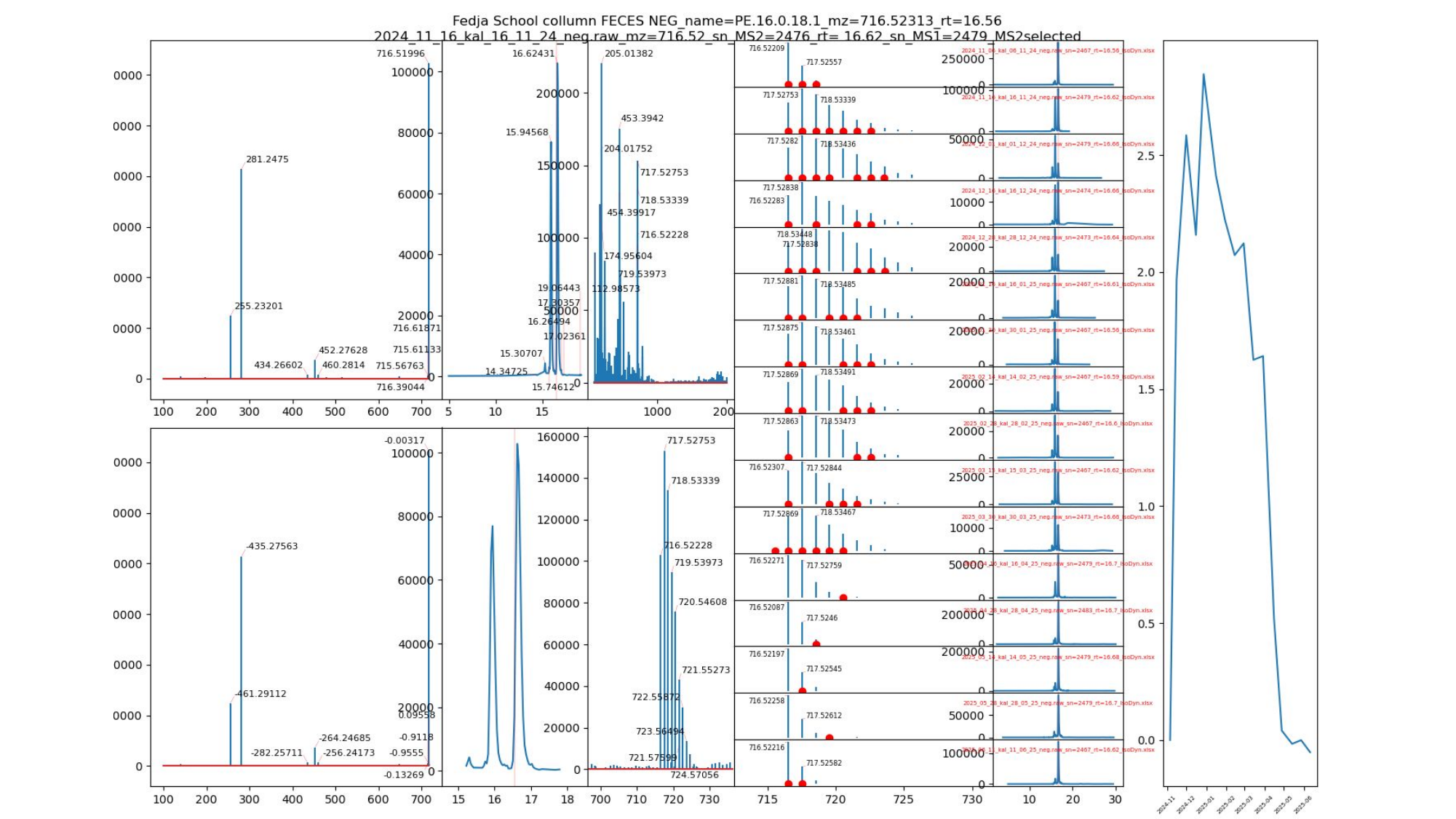

## Slide 38
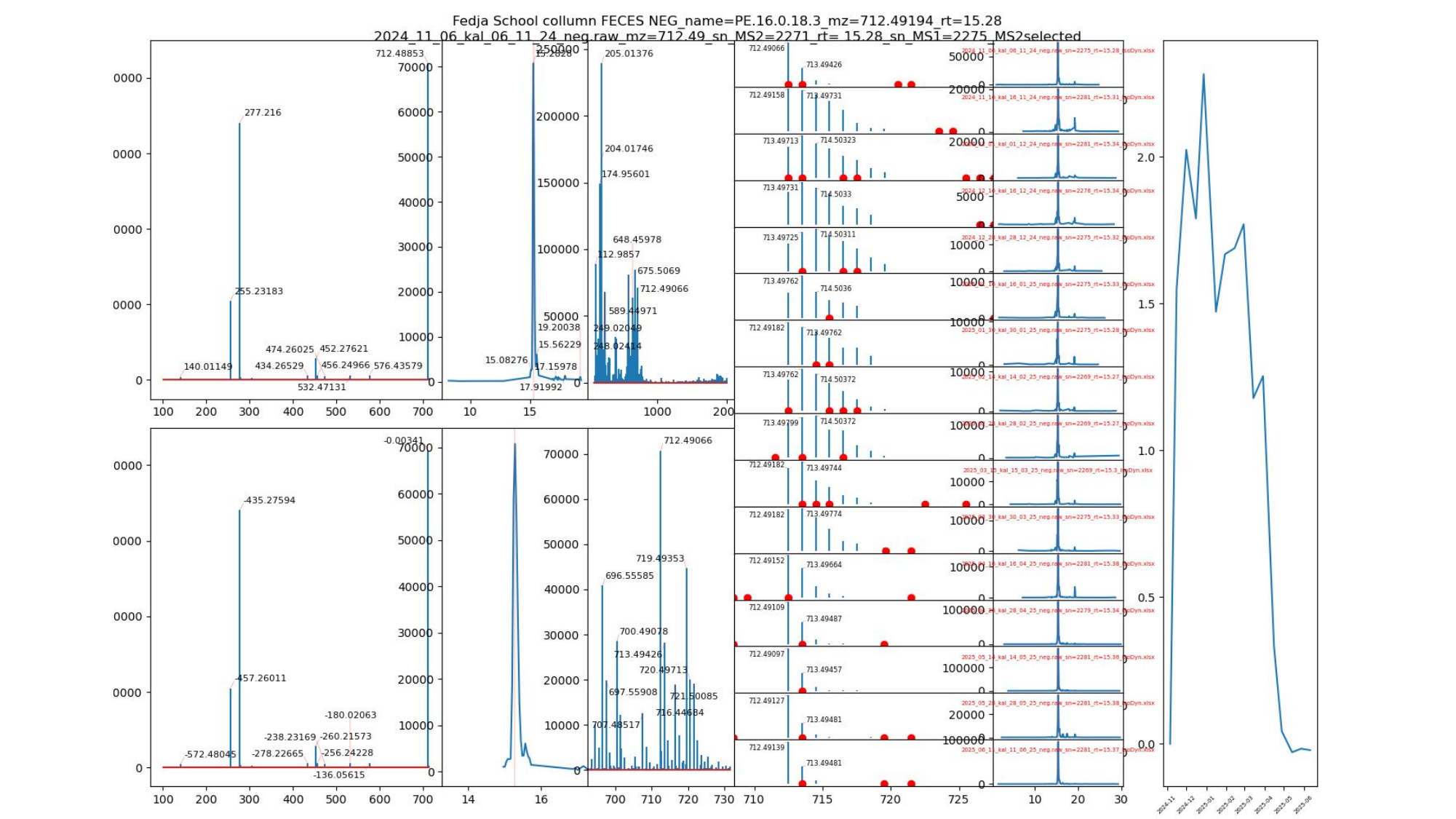

## Slide 39
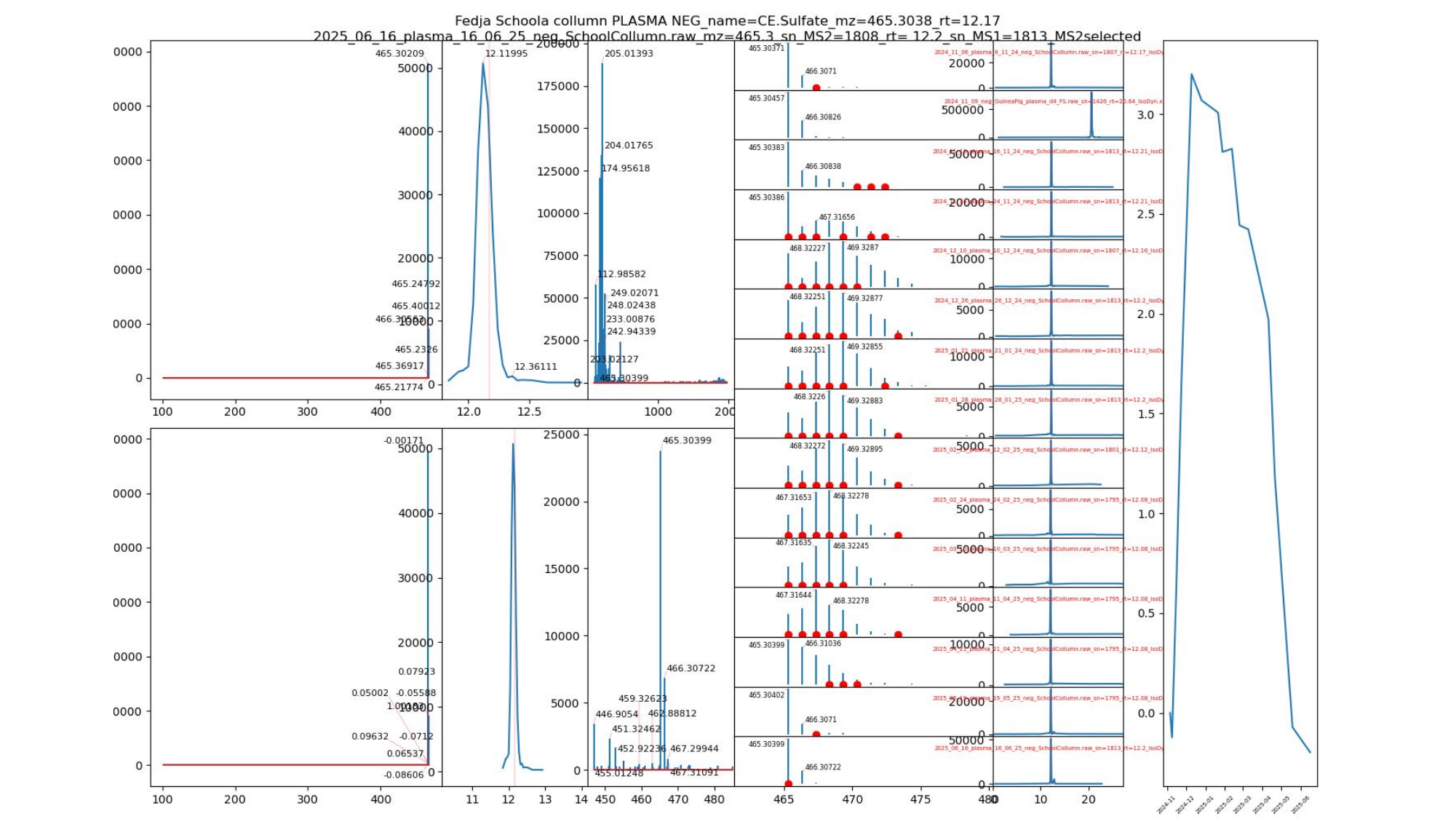

## Slide 40
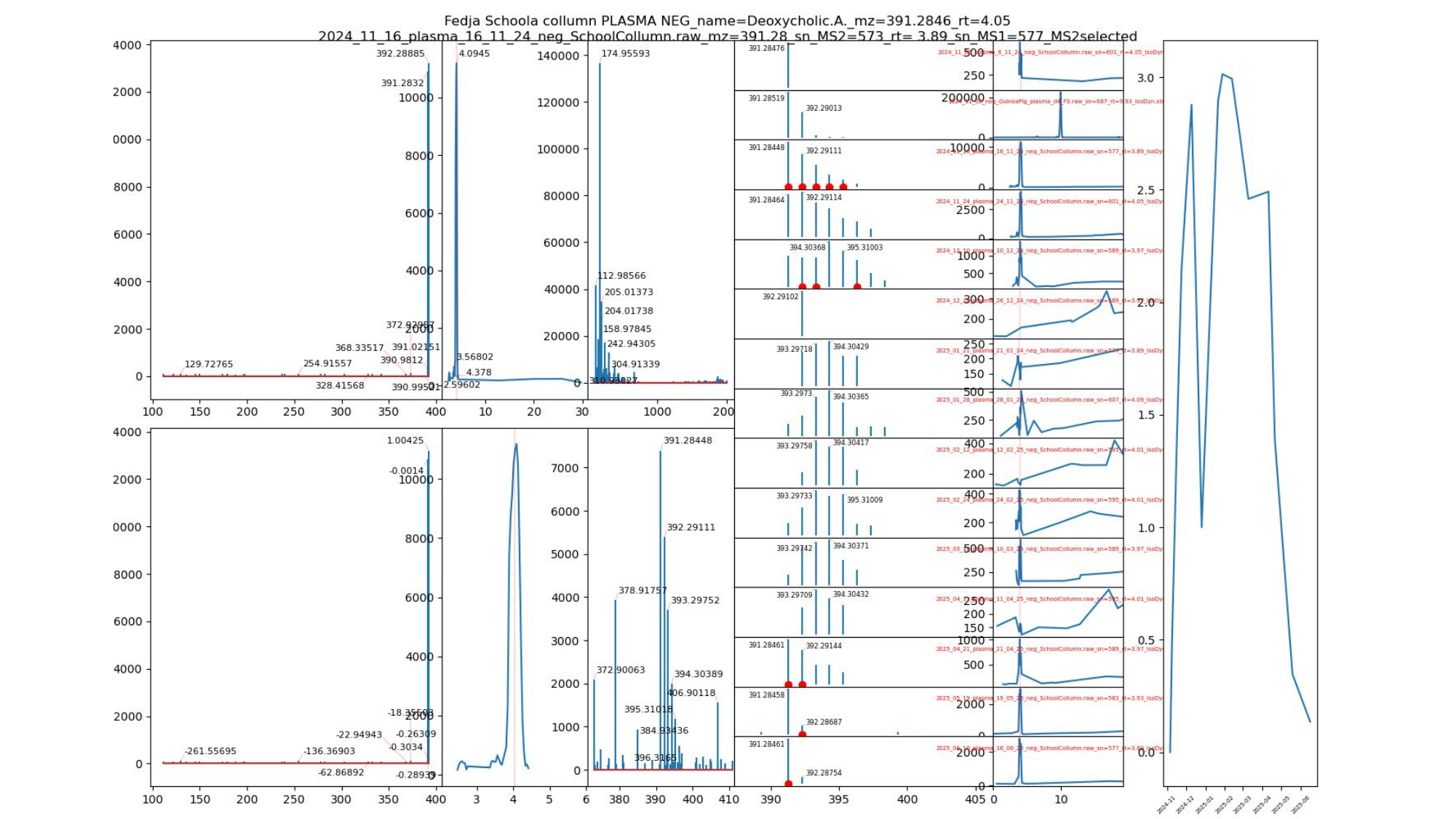

## Slide 41
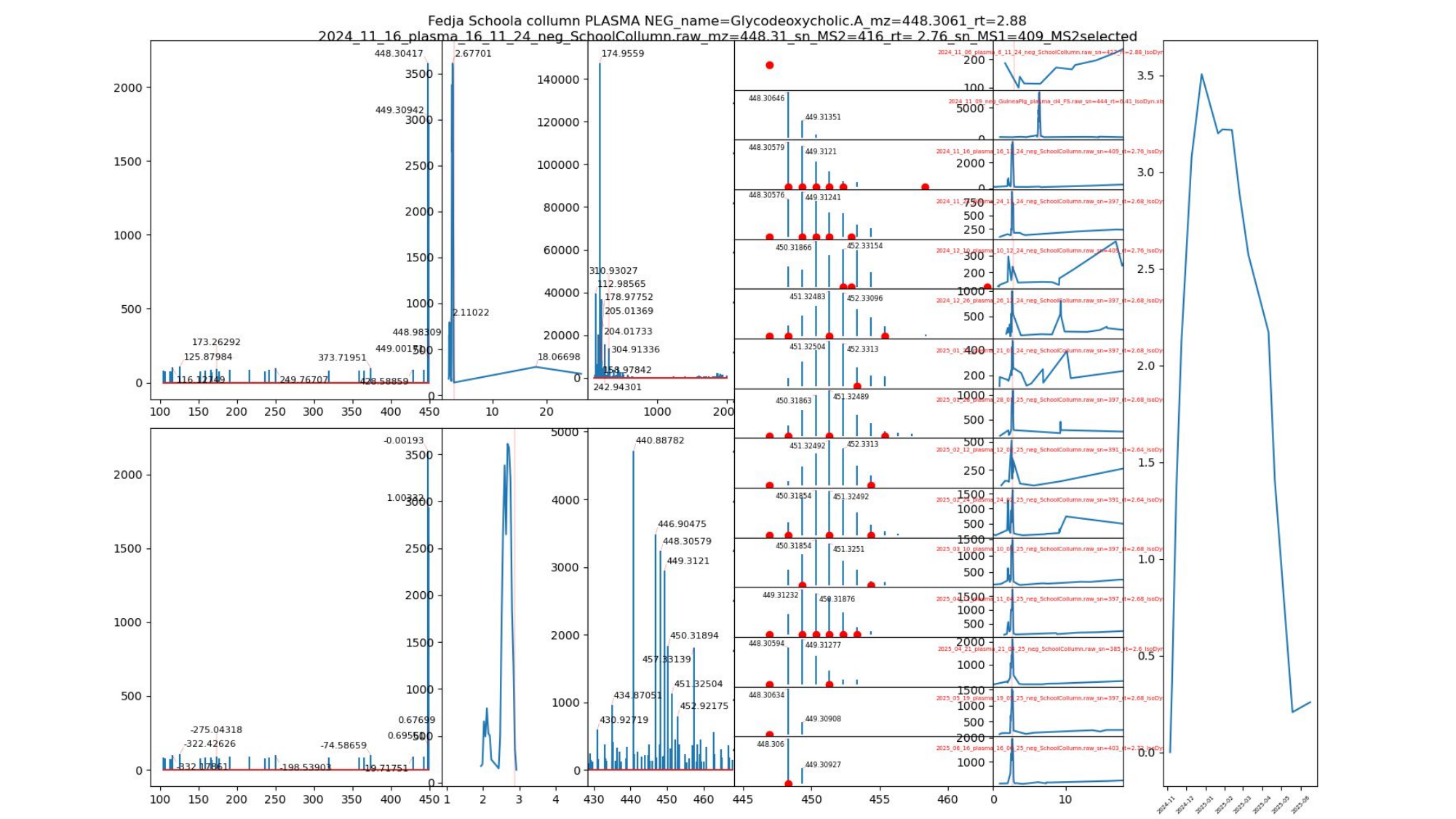

## Slide 42
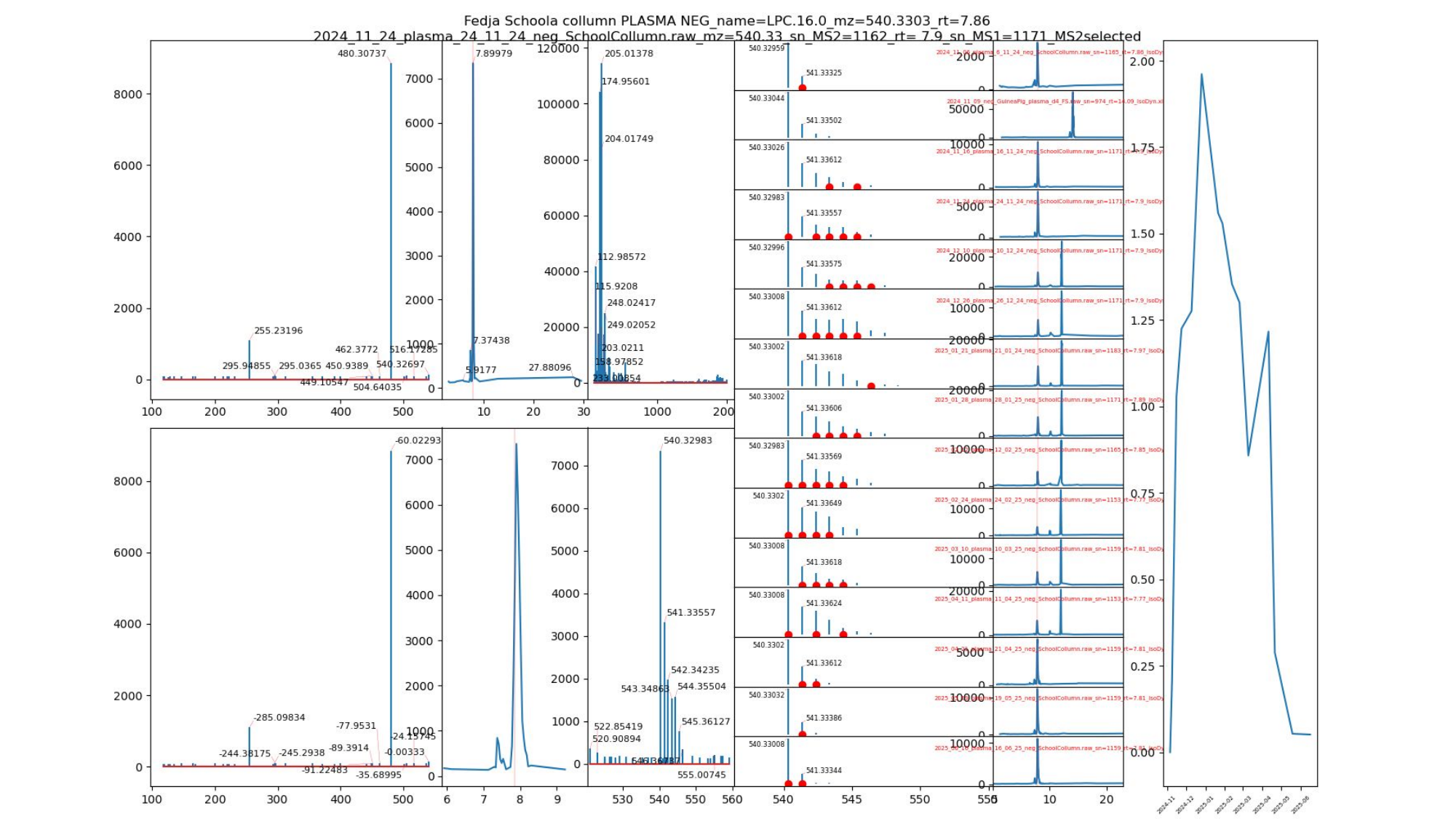

## Slide 43
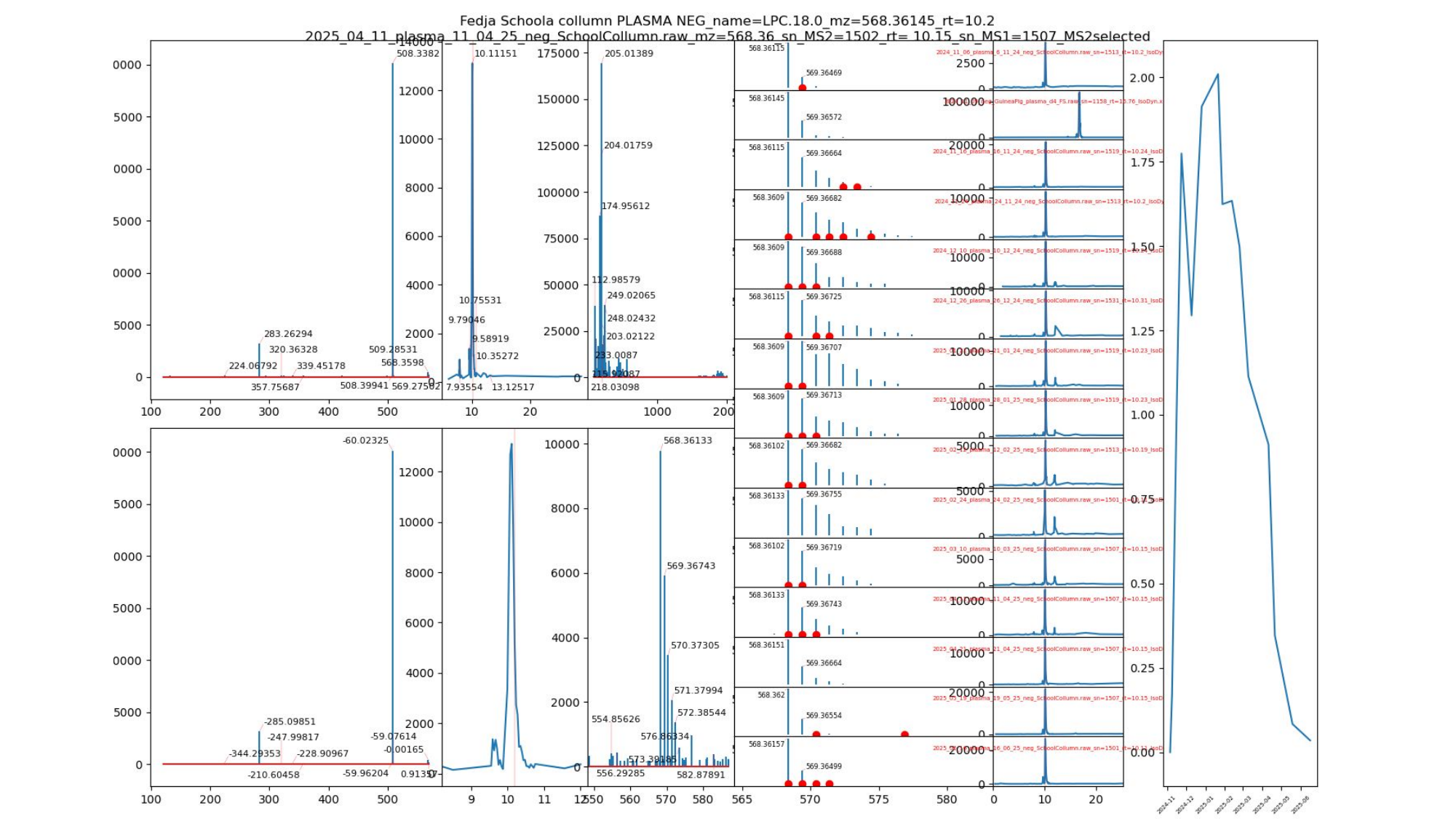

## Slide 44
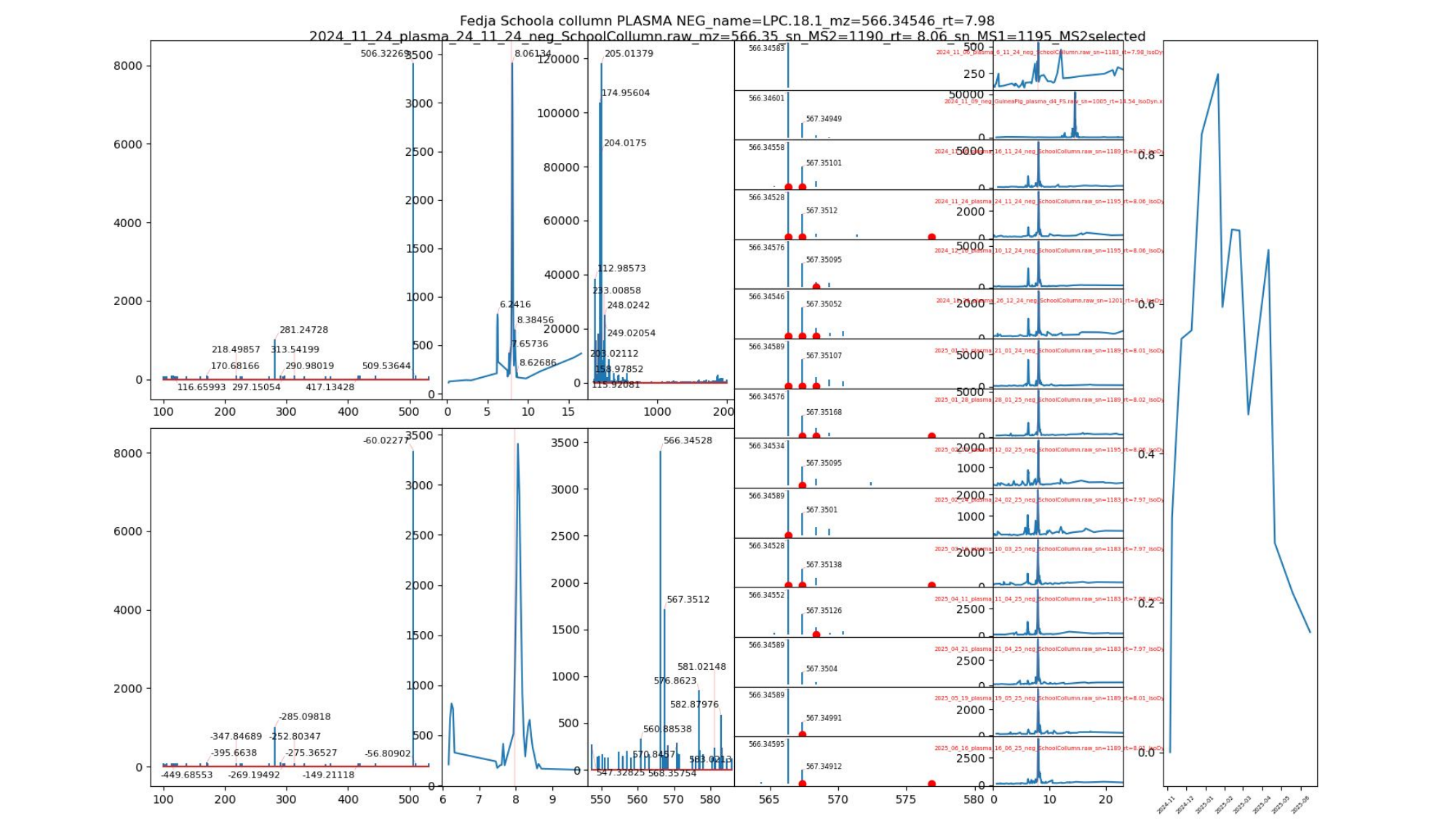

## Slide 45
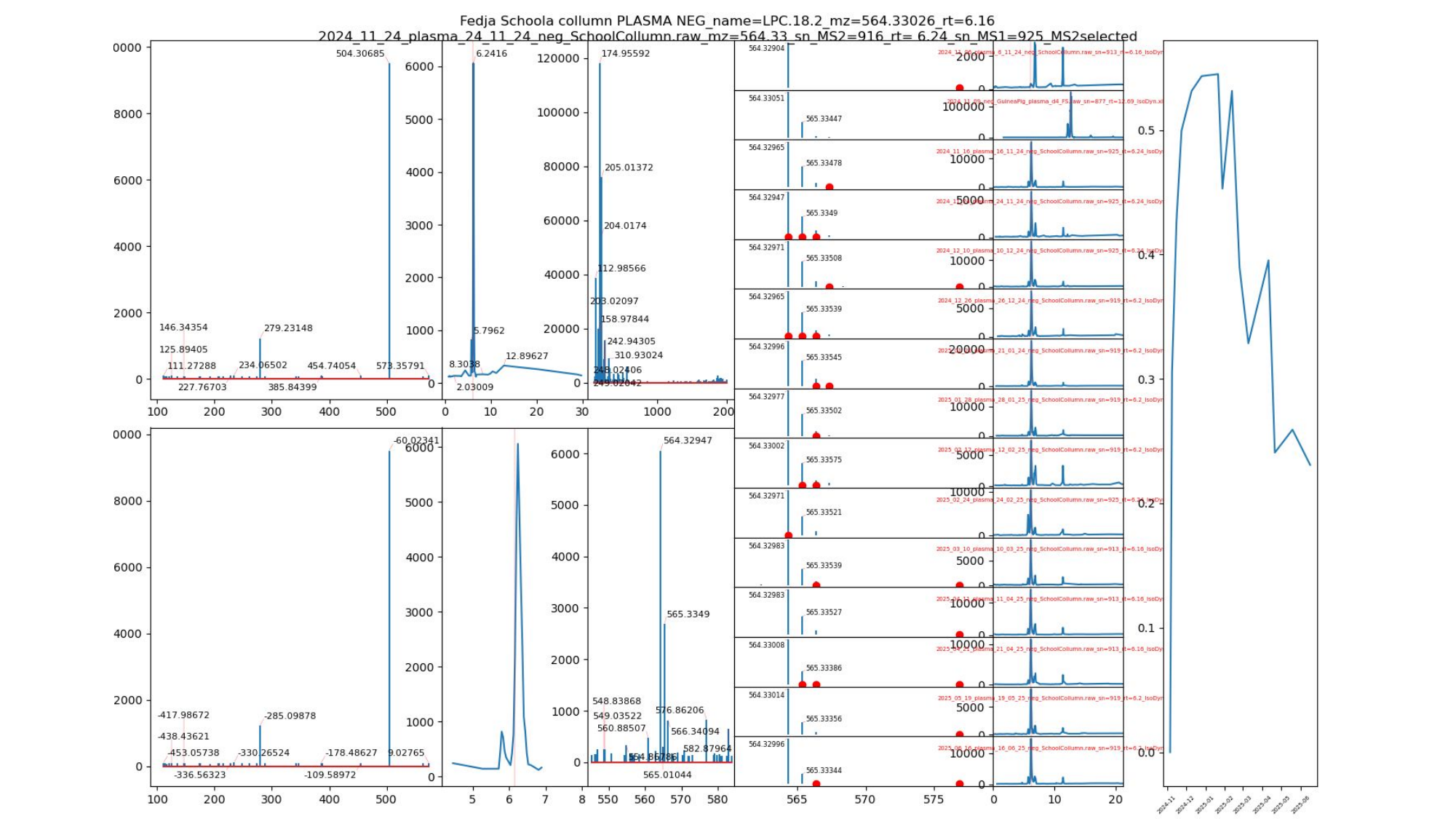

## Slide 46
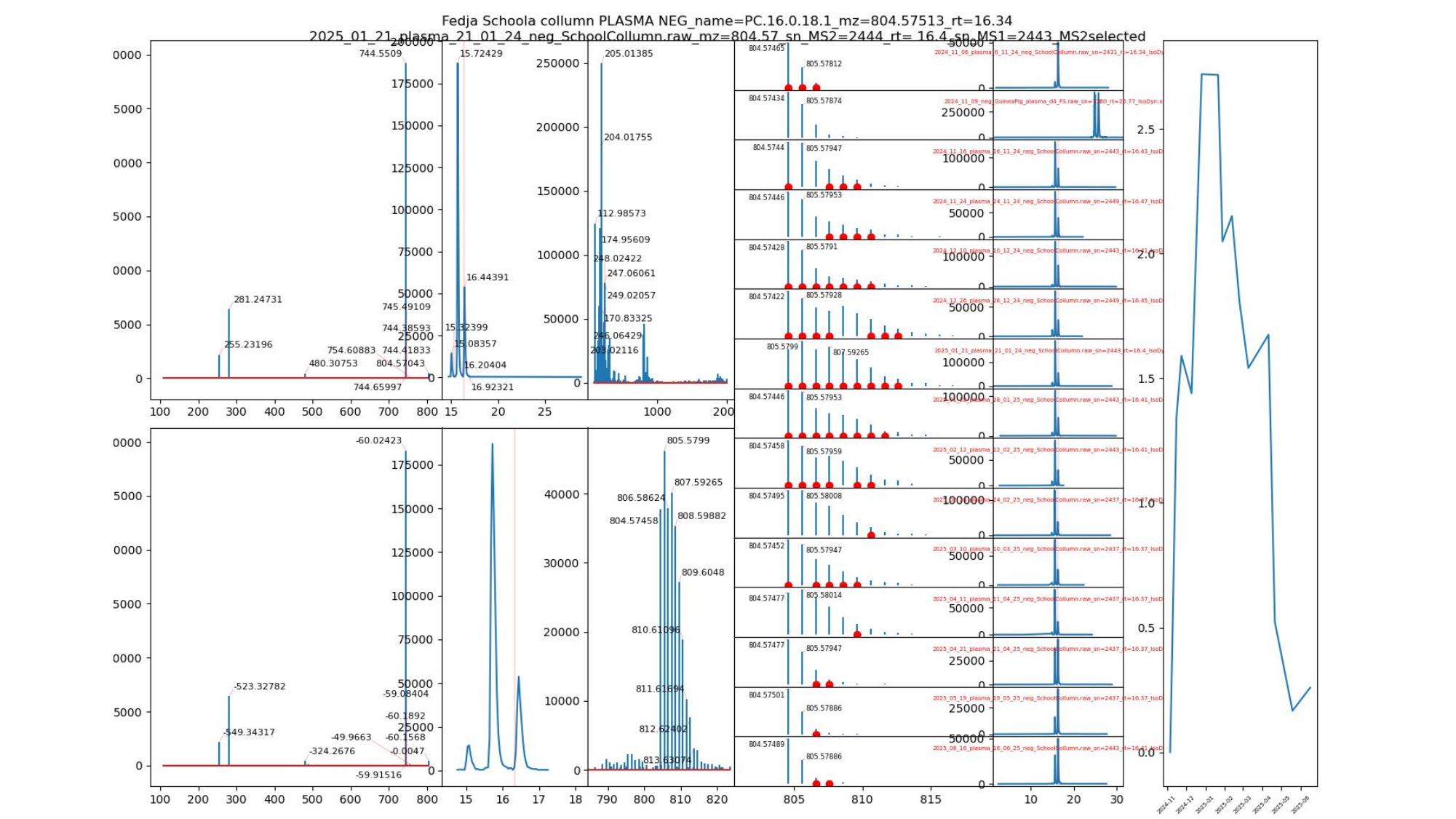

## Slide 47
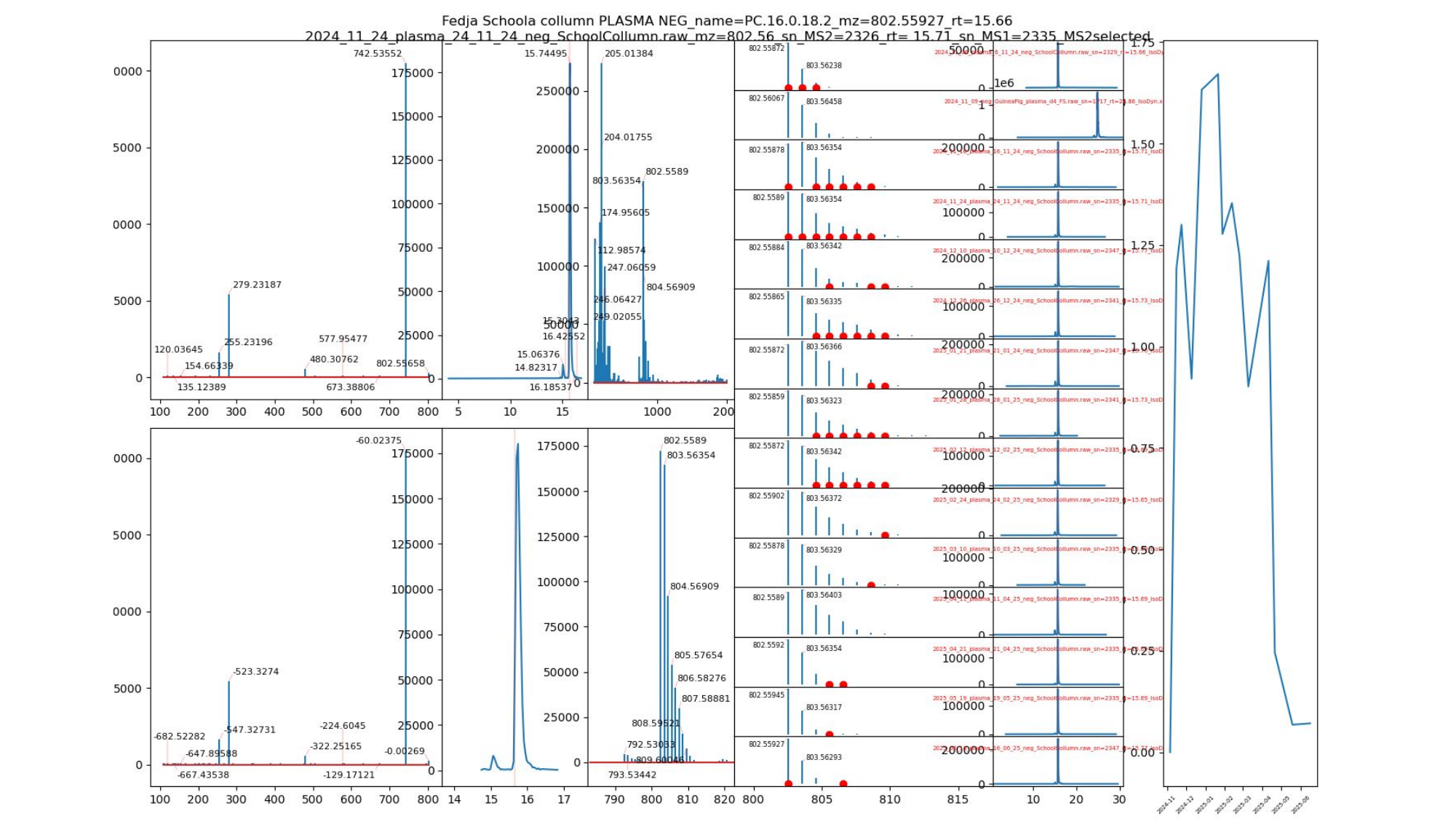

## Slide 48
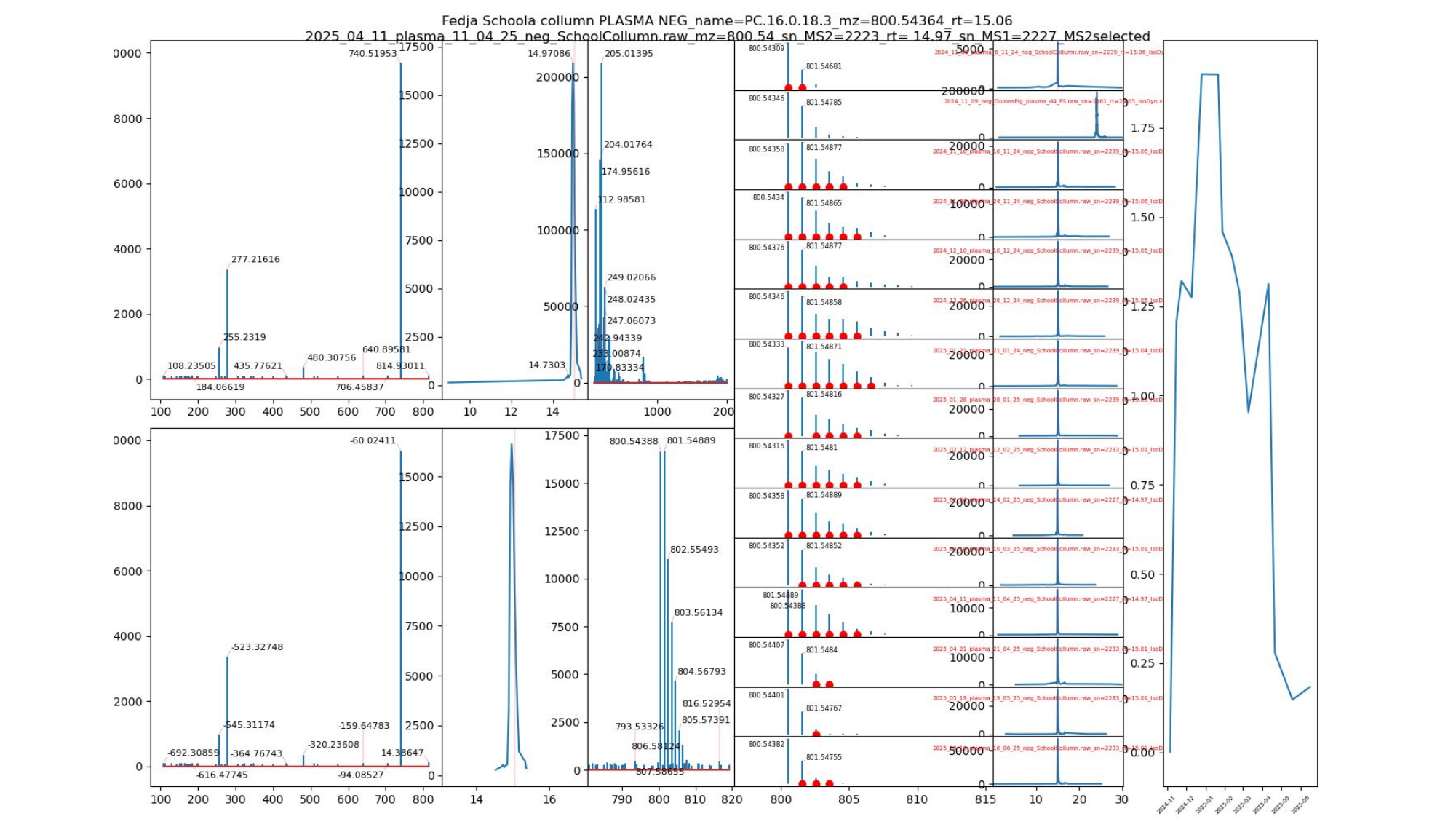

## Slide 49
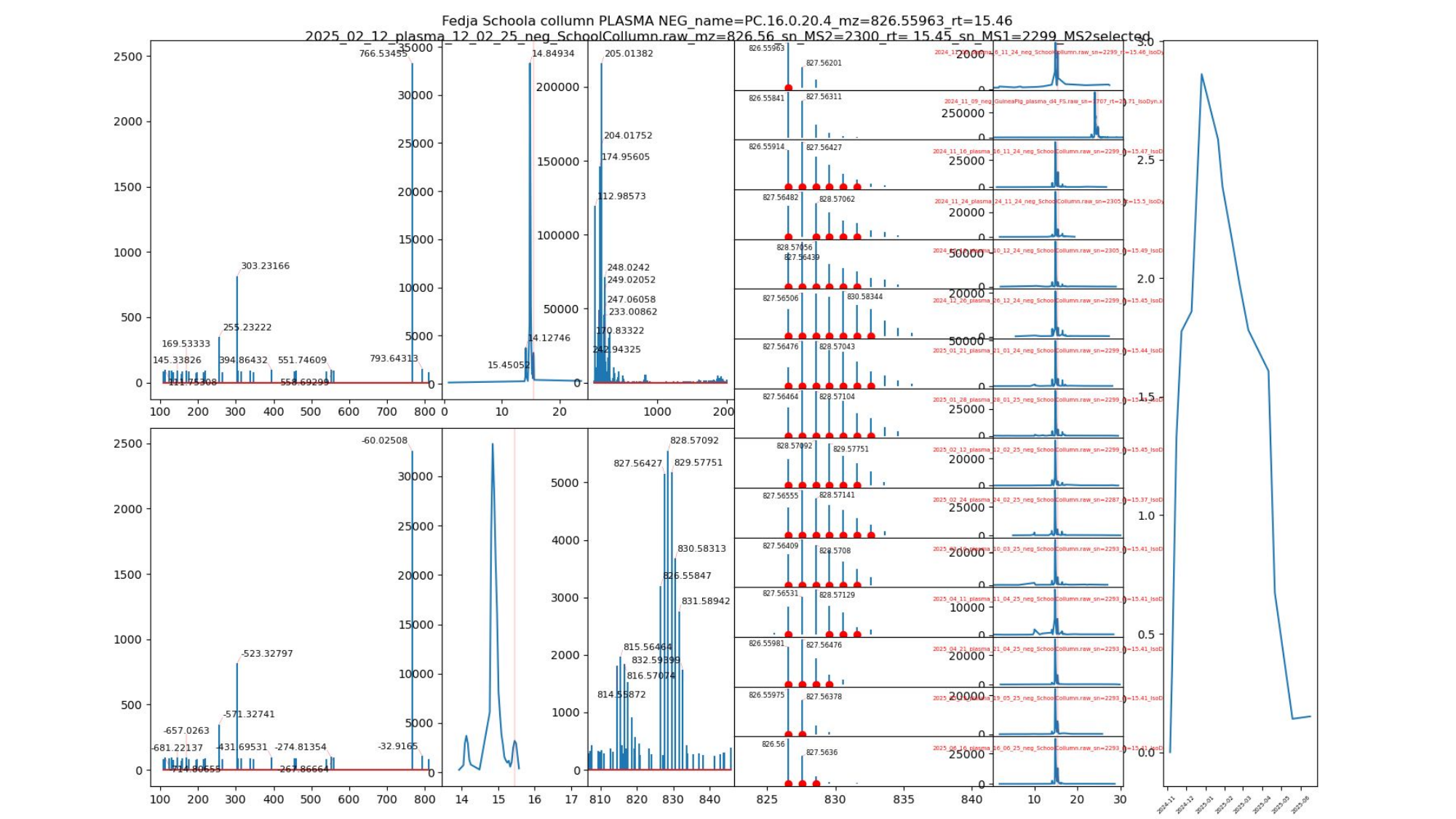

## Slide 50
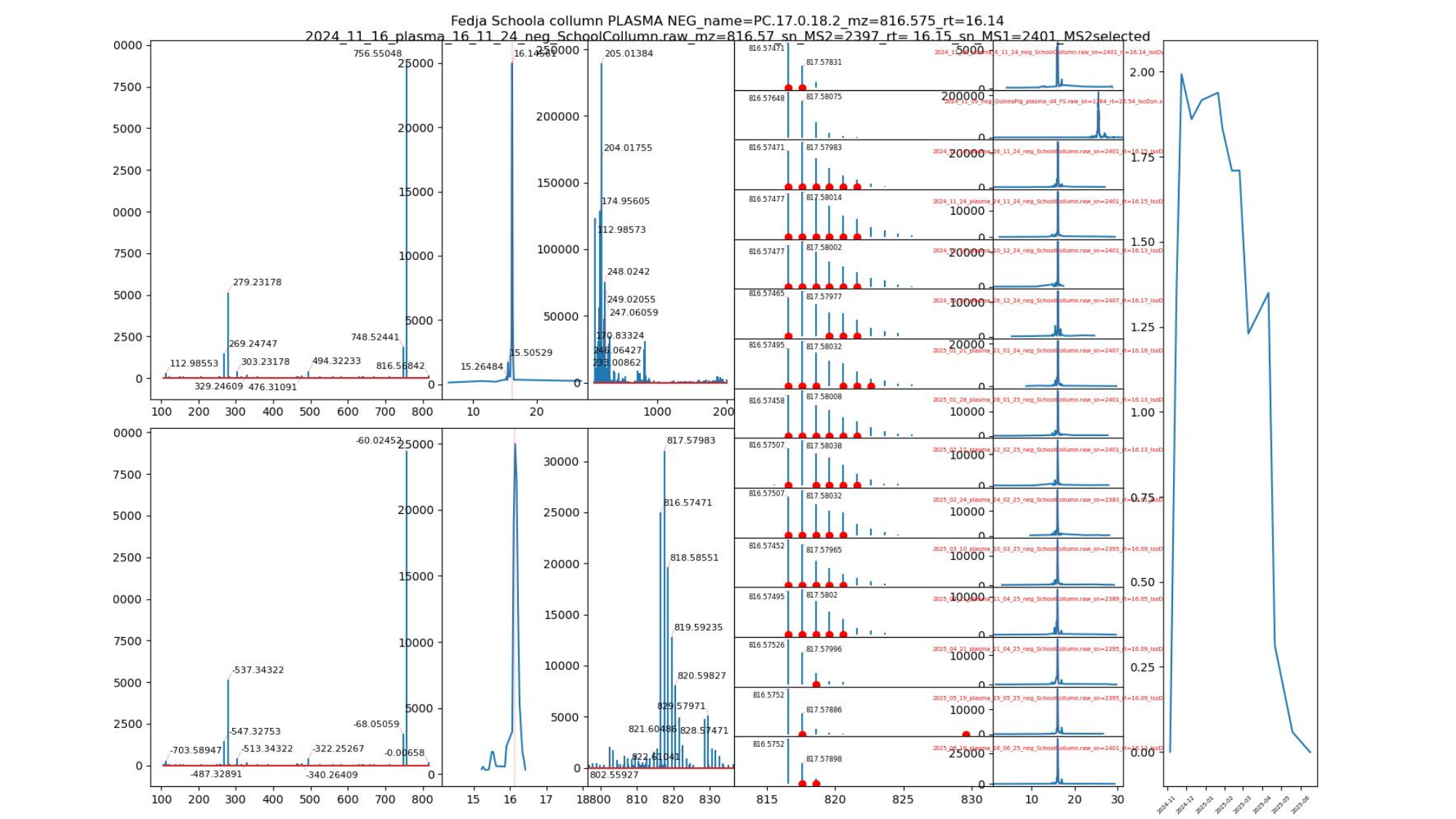

## Slide 51
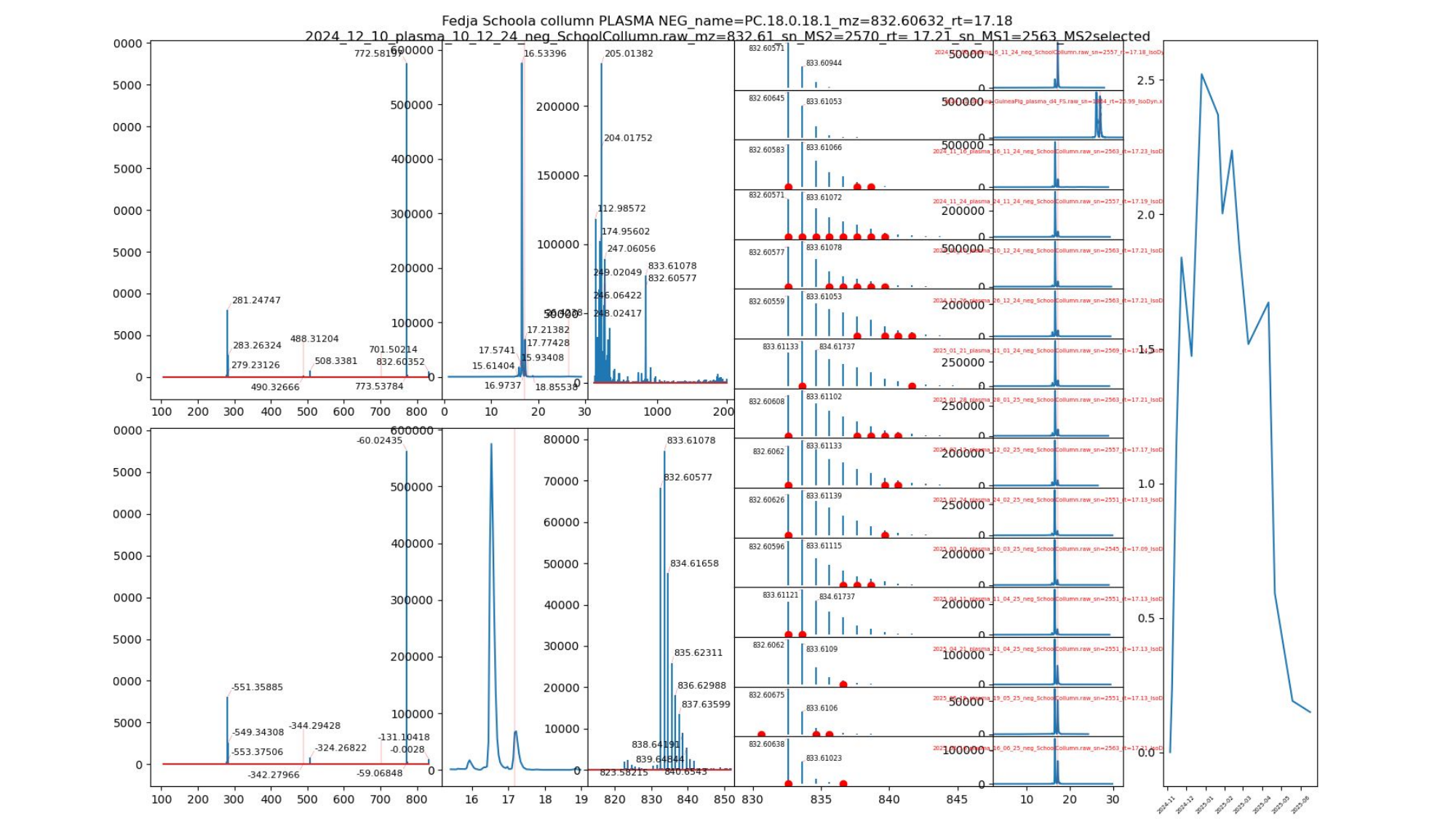

## Slide 52
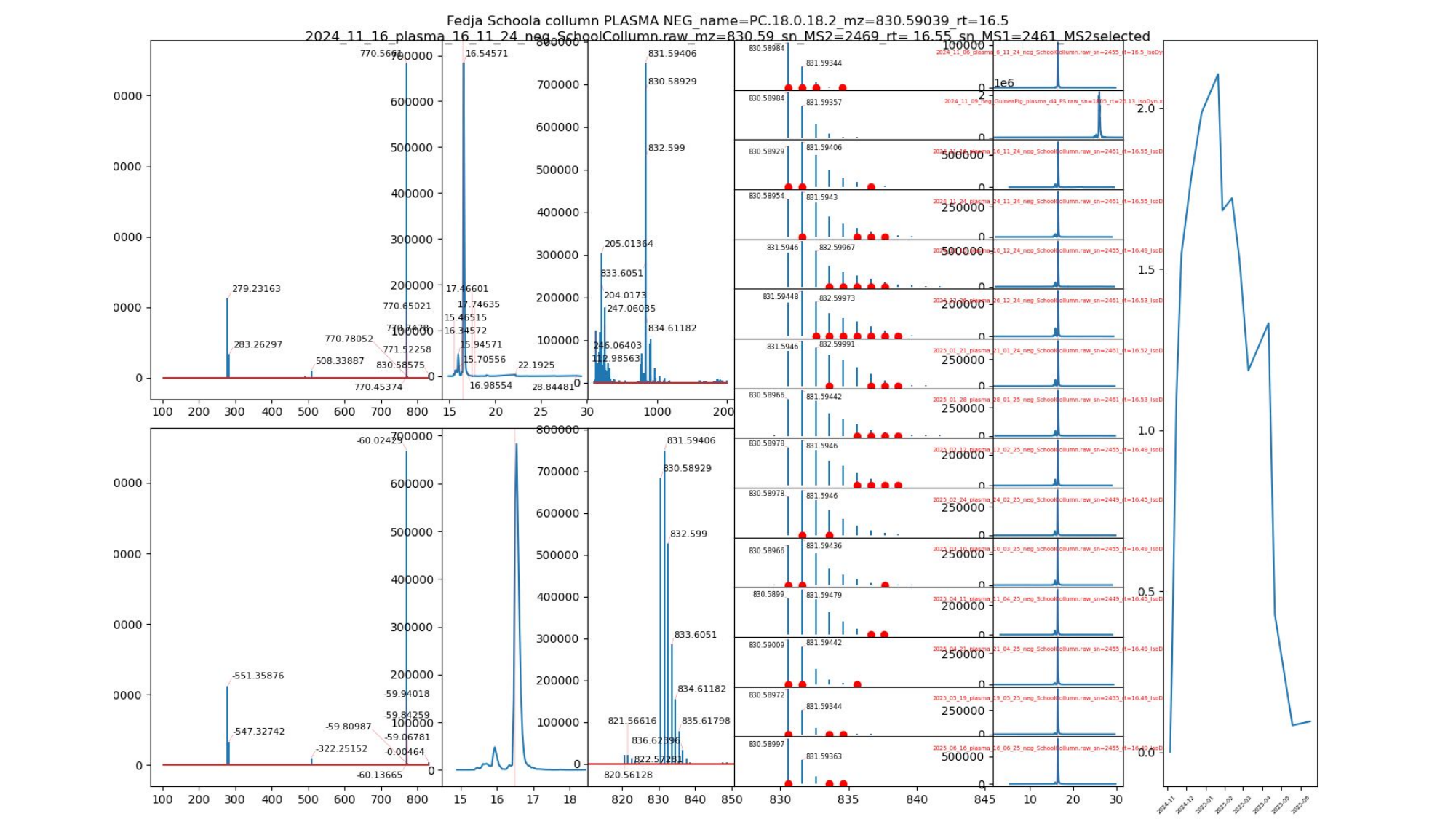

## Slide 53
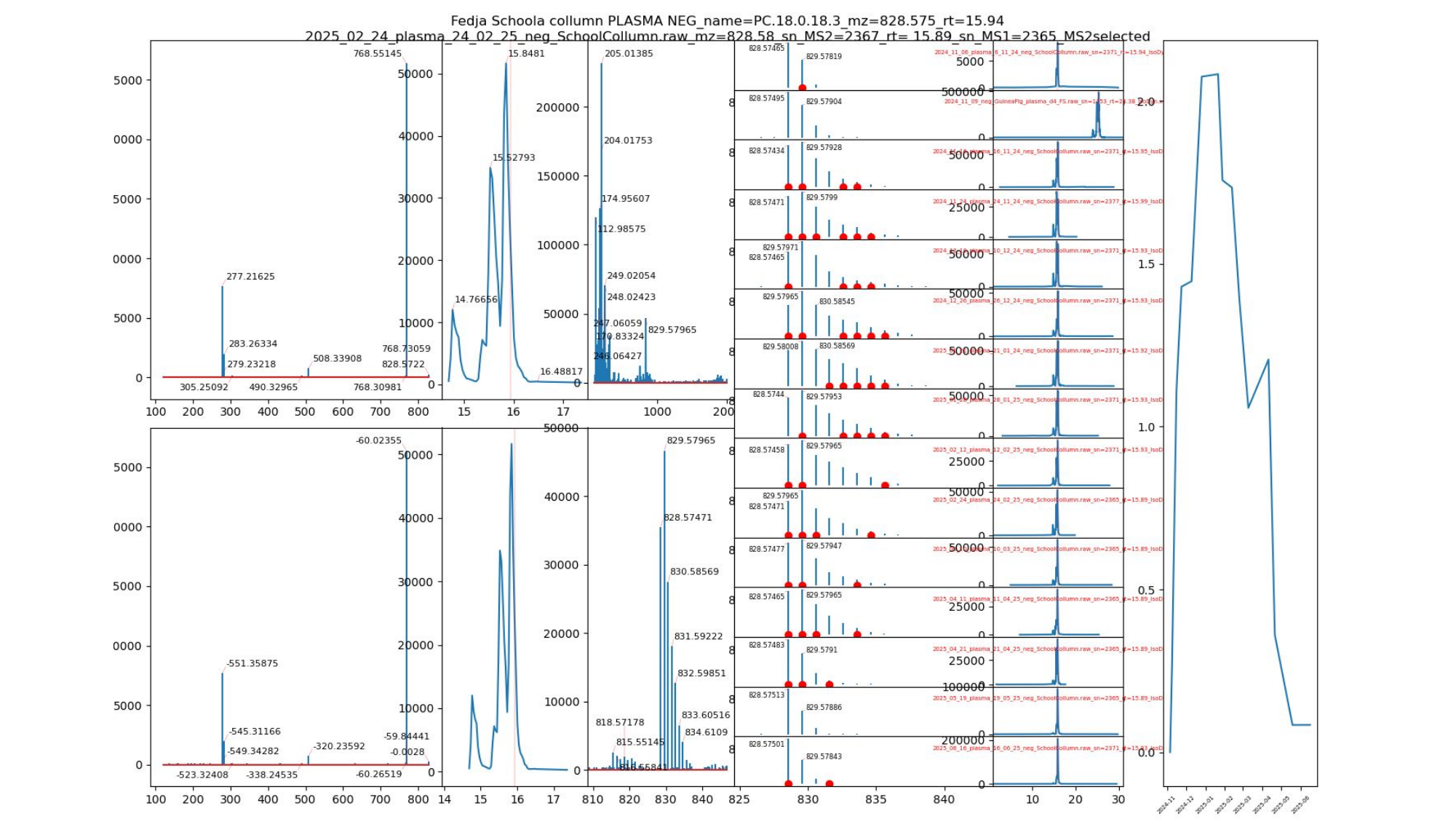

## Slide 54
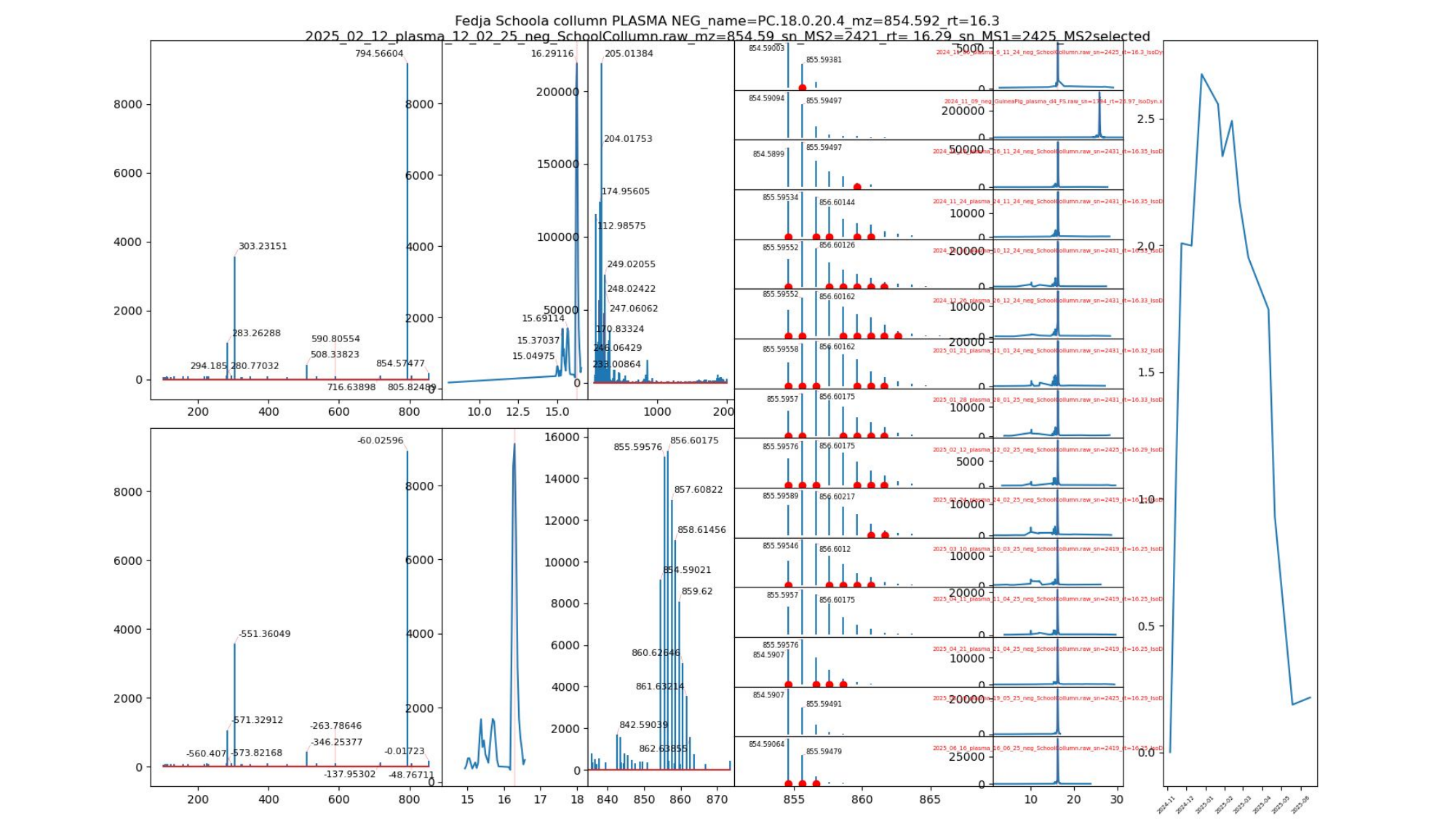

## Slide 55
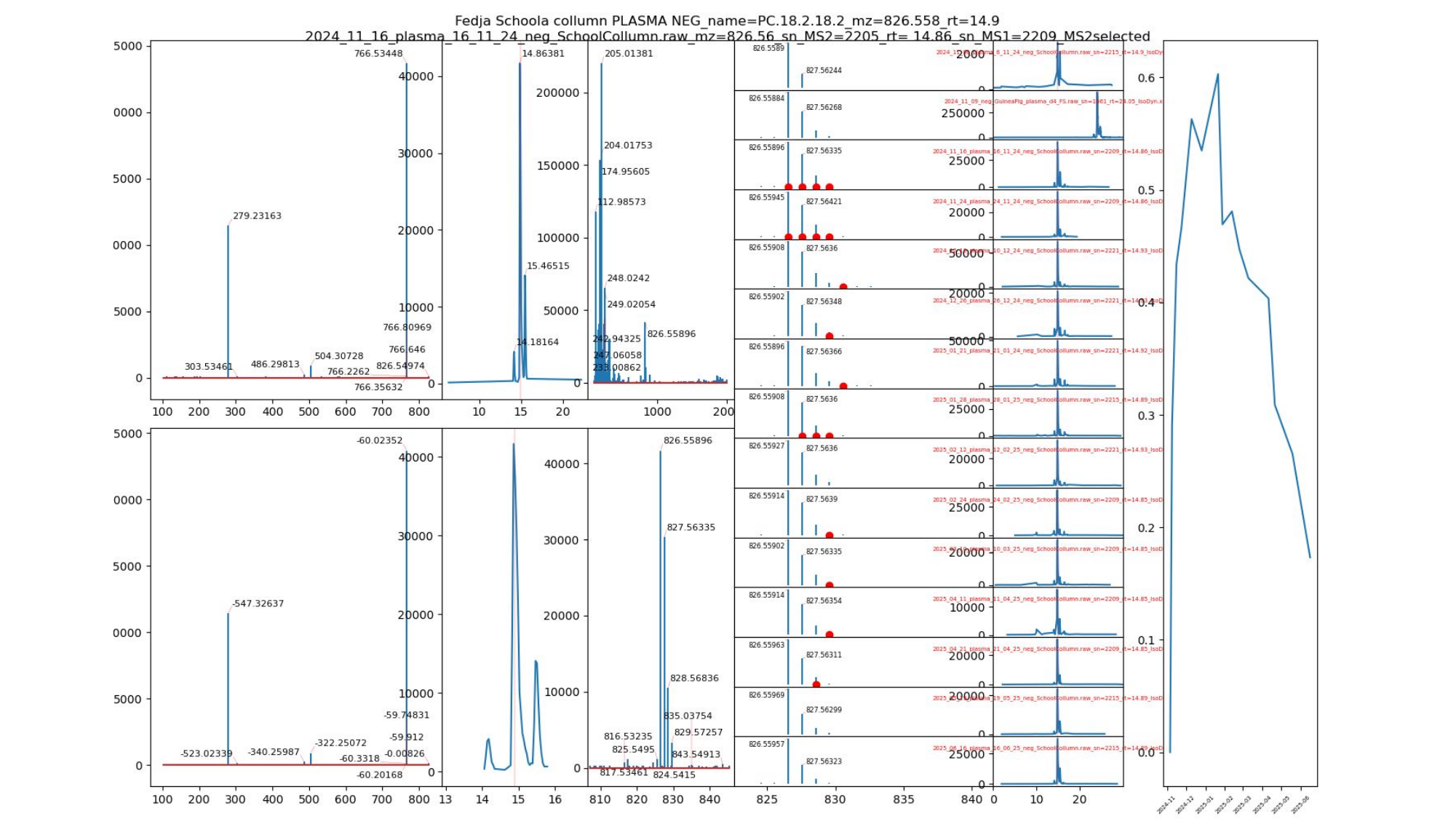

## Slide 56
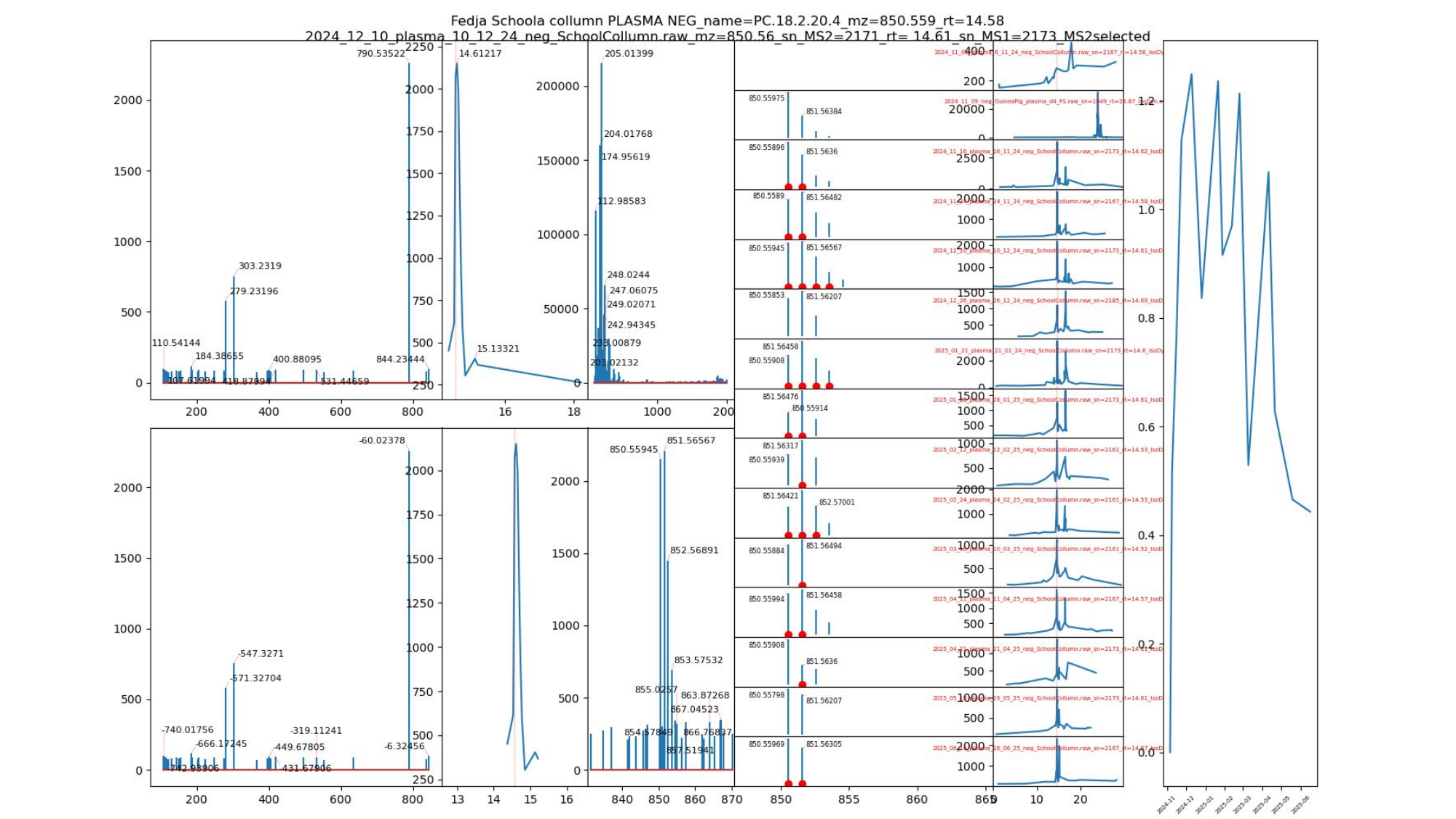

## Slide 57
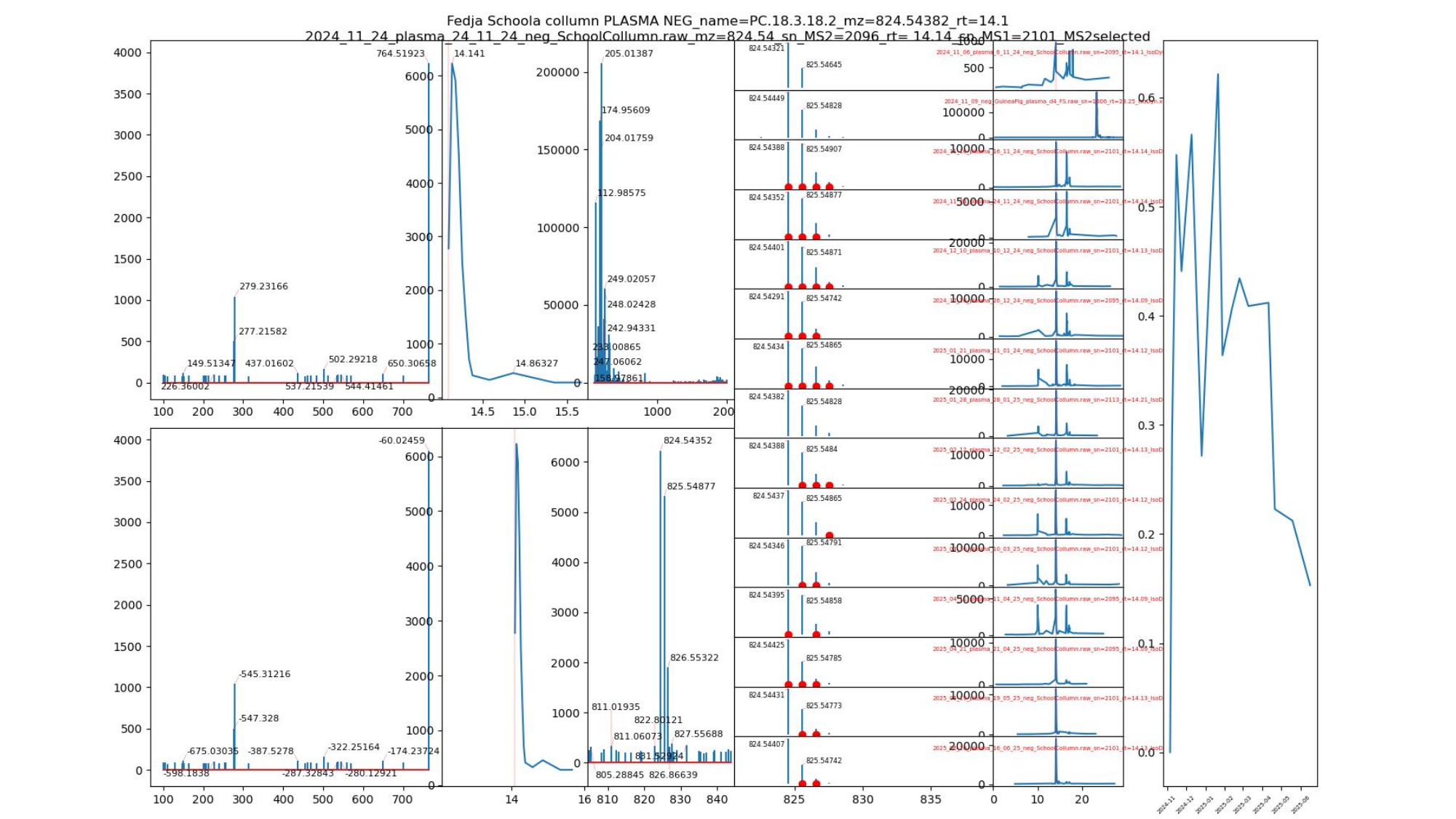

## Slide 58
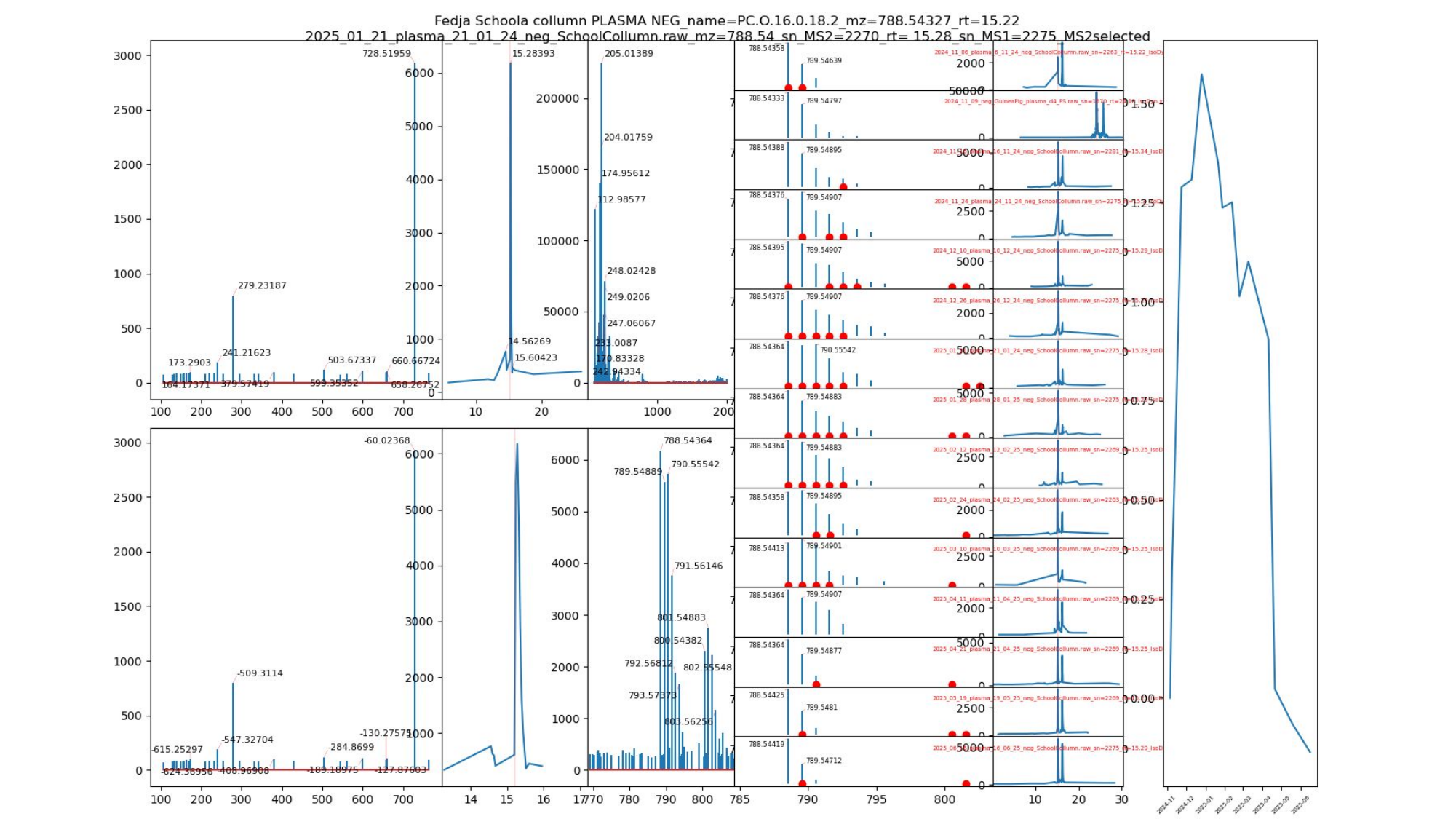

## Slide 59
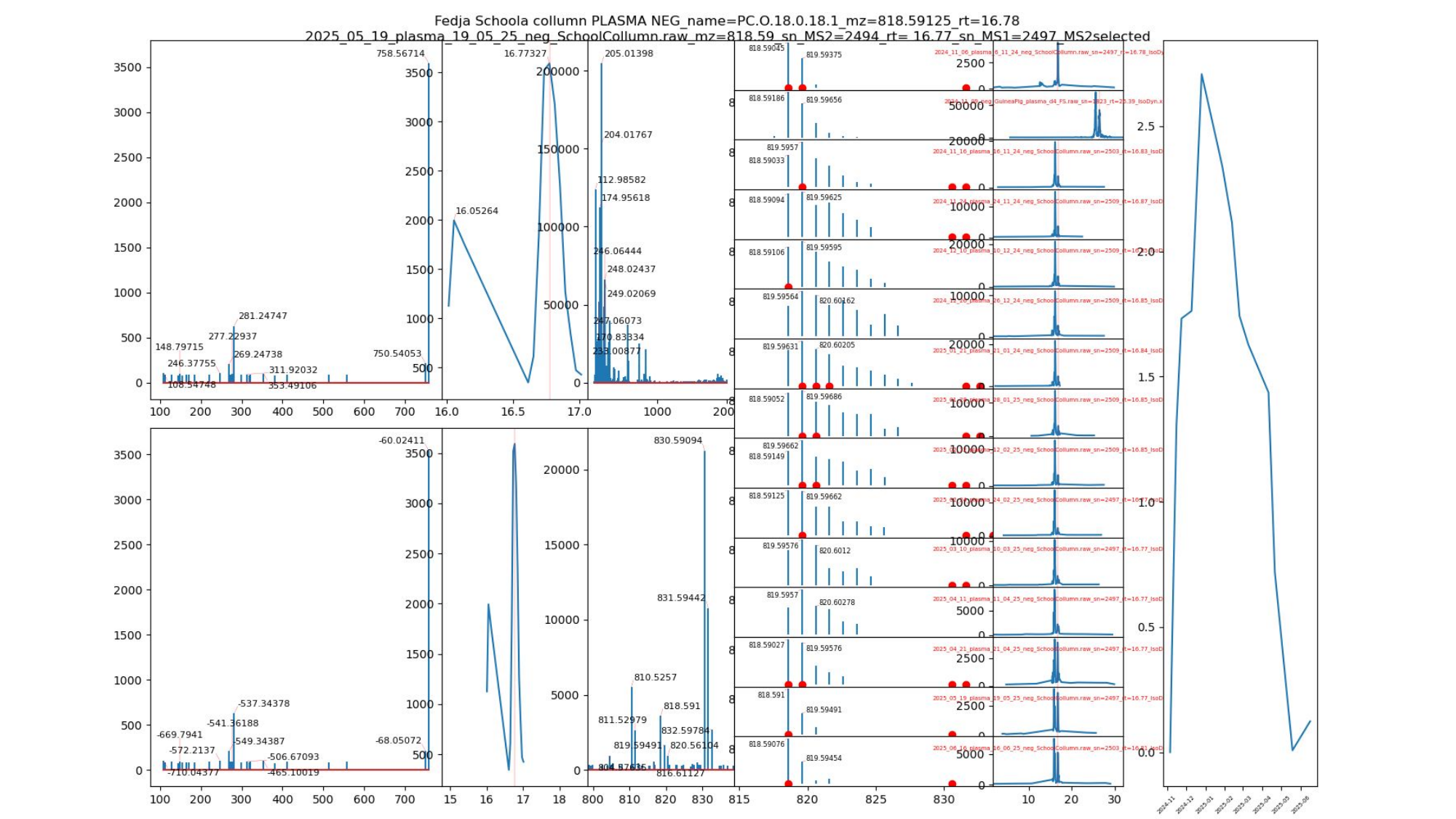

## Slide 60
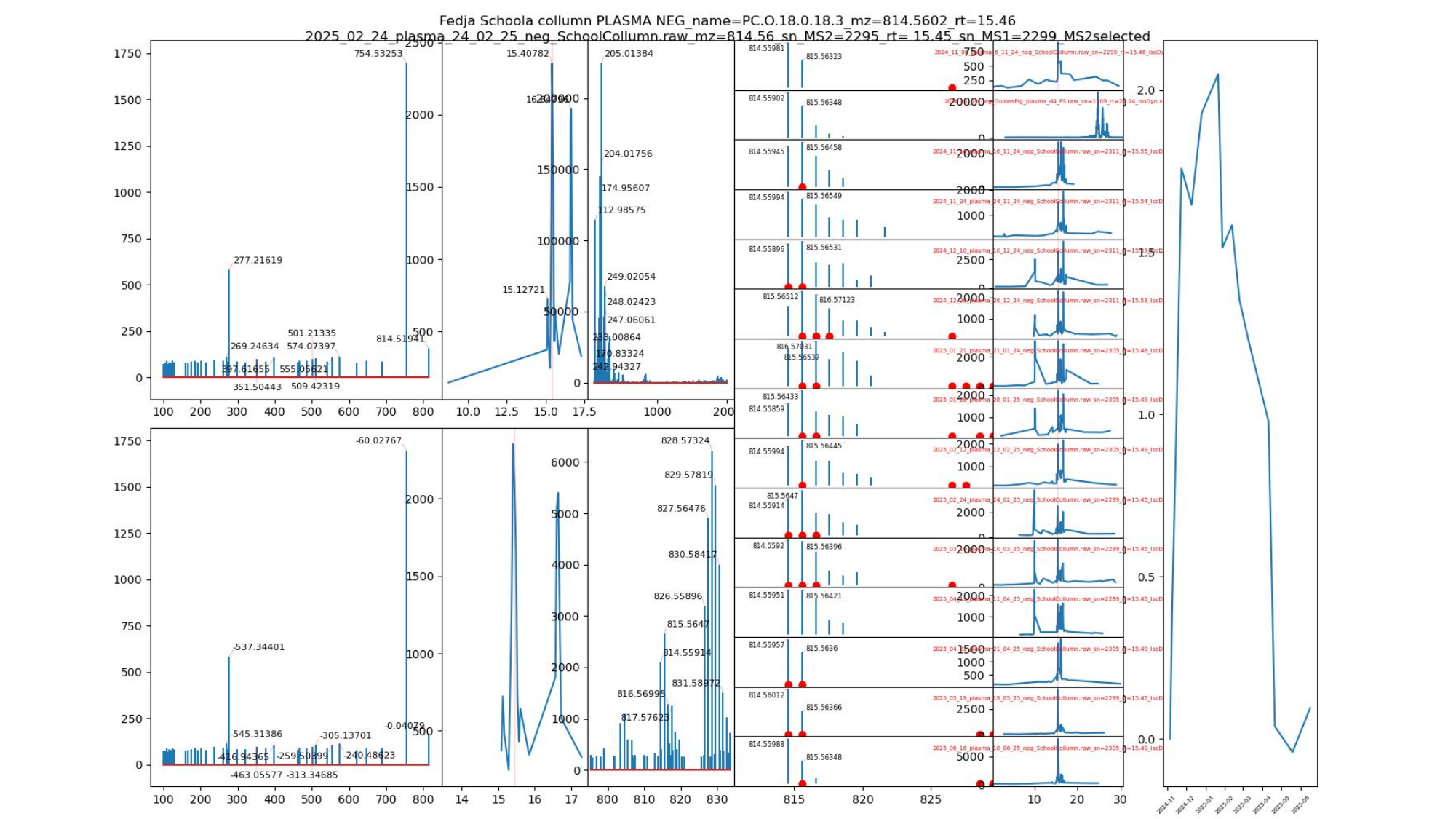

## Slide 61
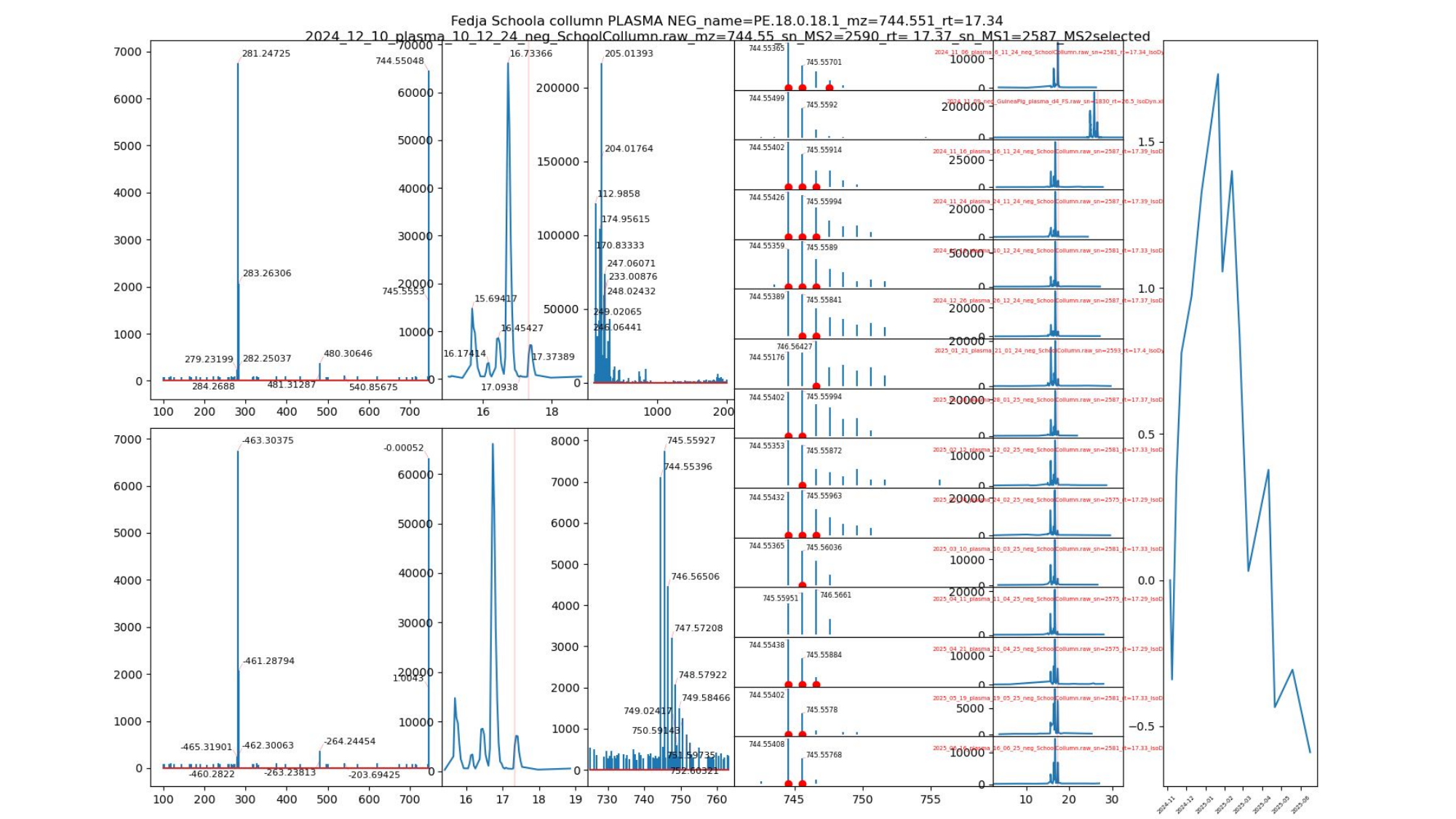

## Slide 62
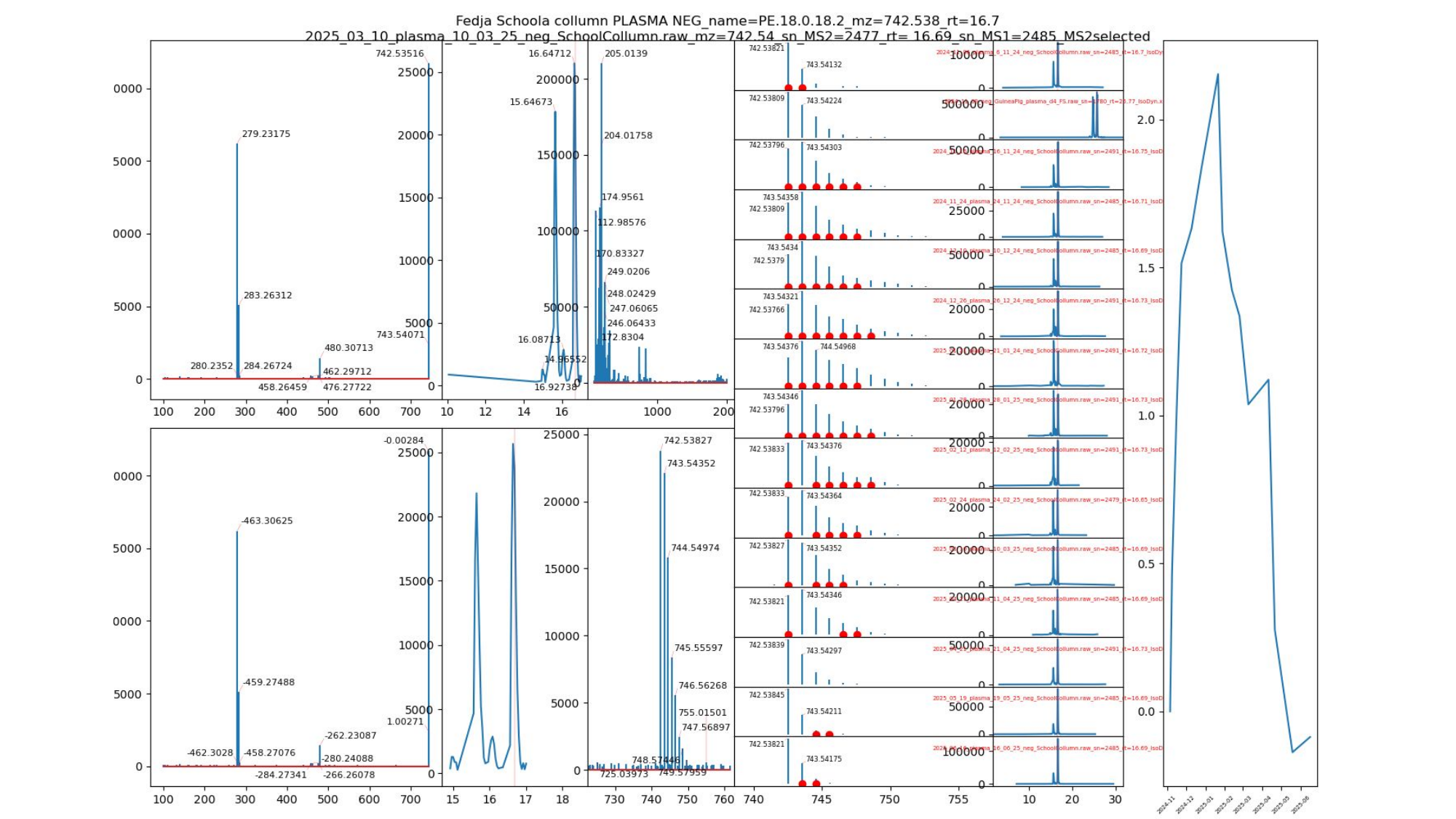

## Slide 63
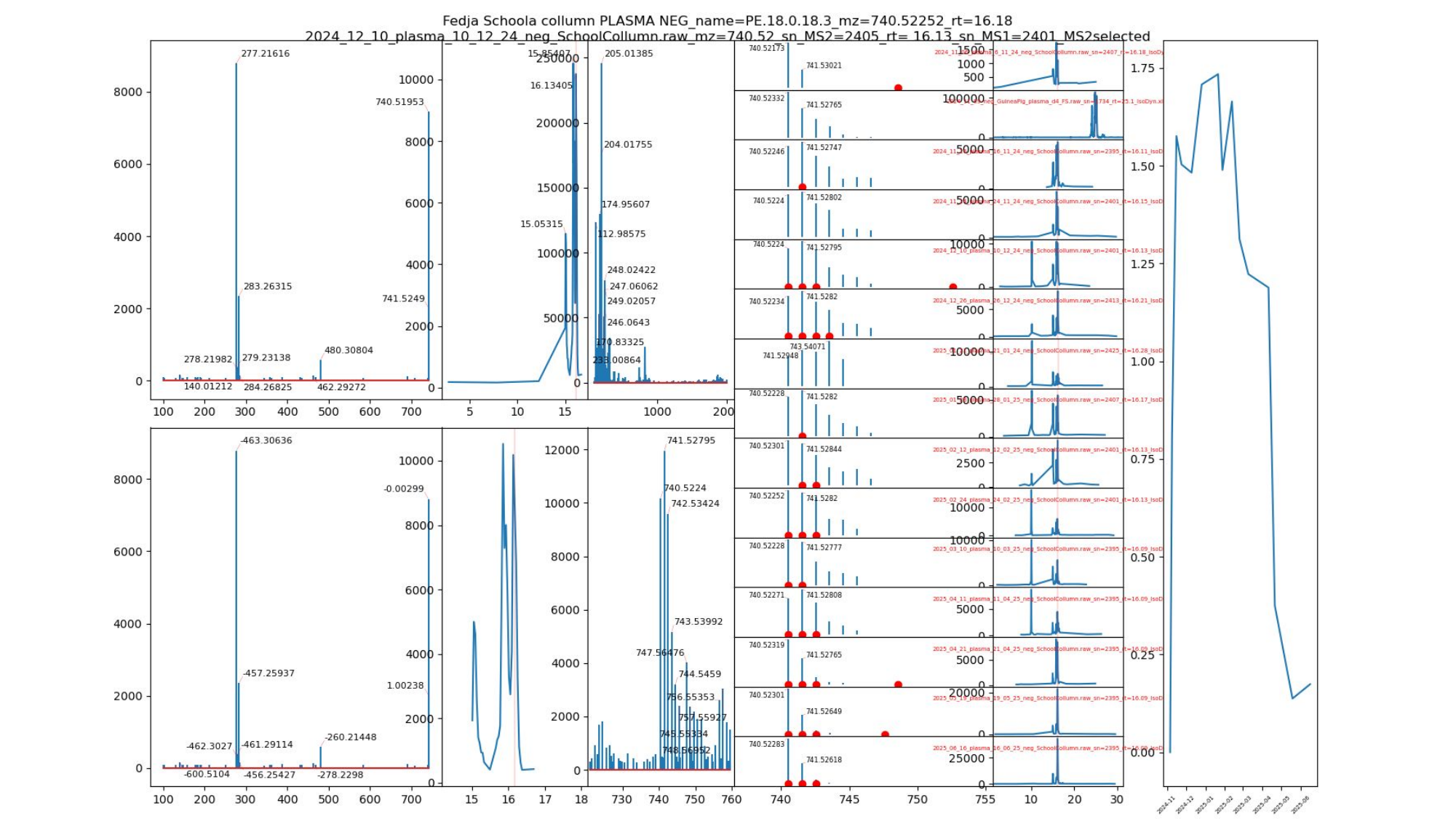

## Slide 64
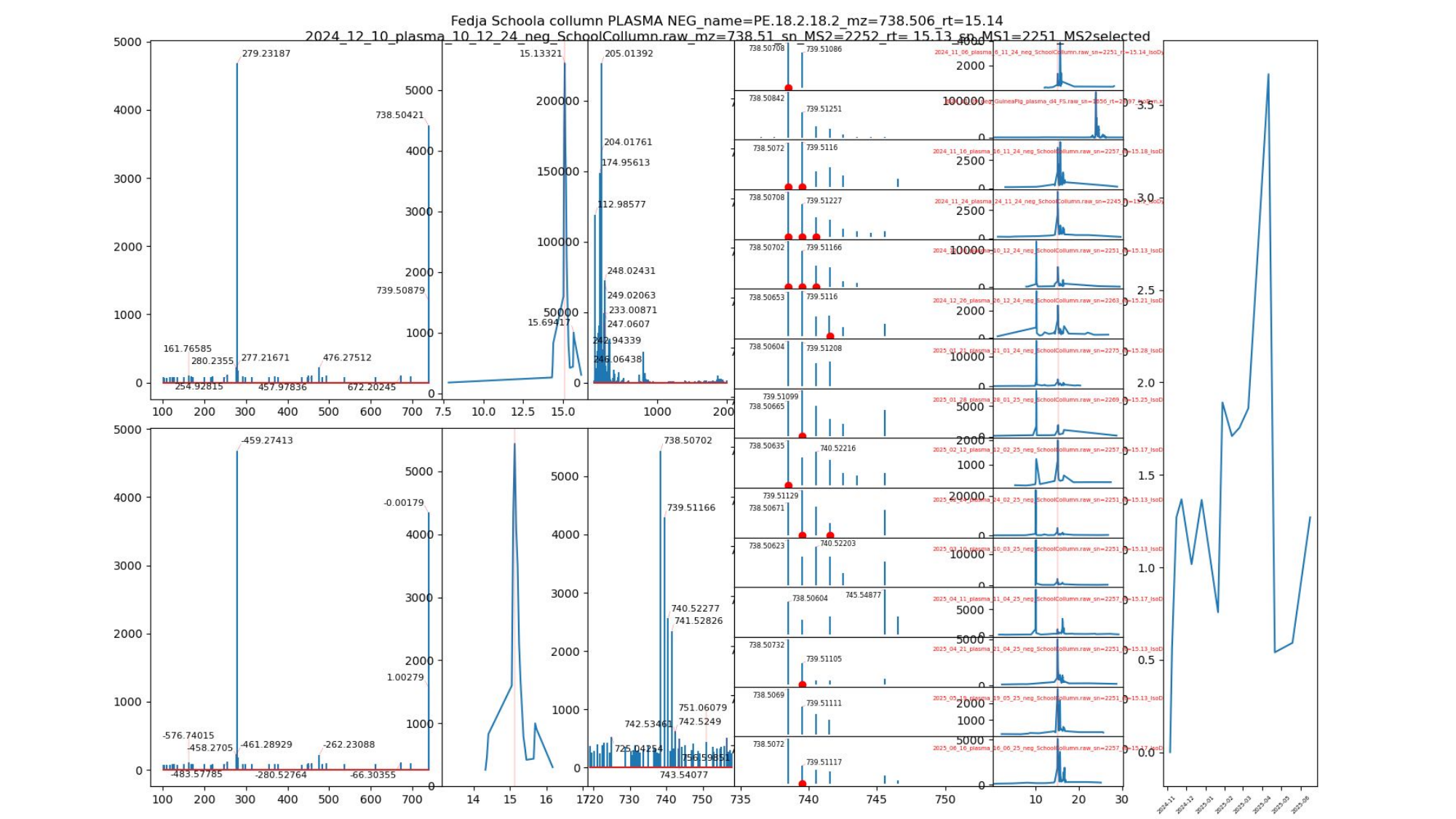

## Slide 65
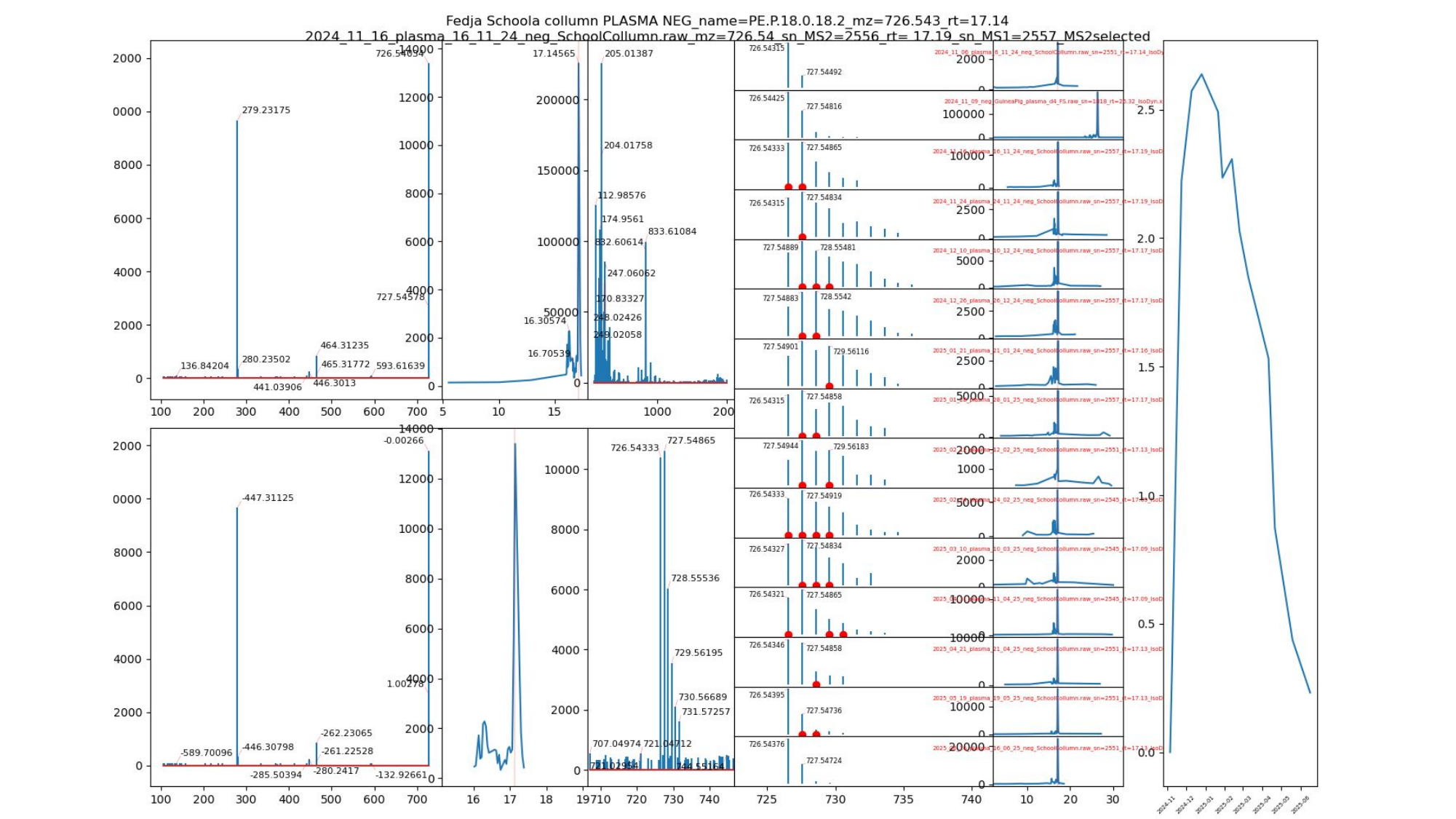

## Slide 66
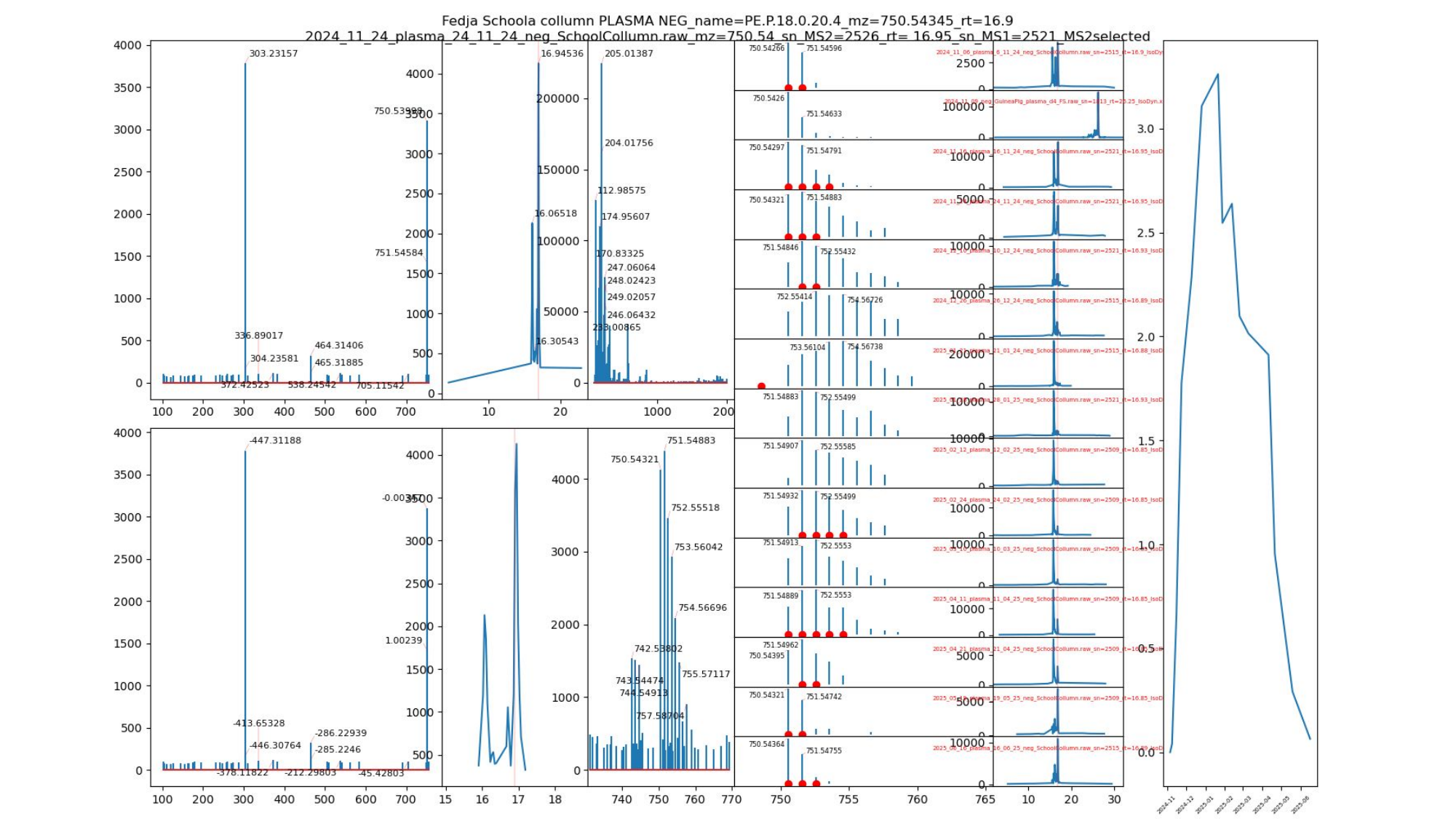

## Slide 67
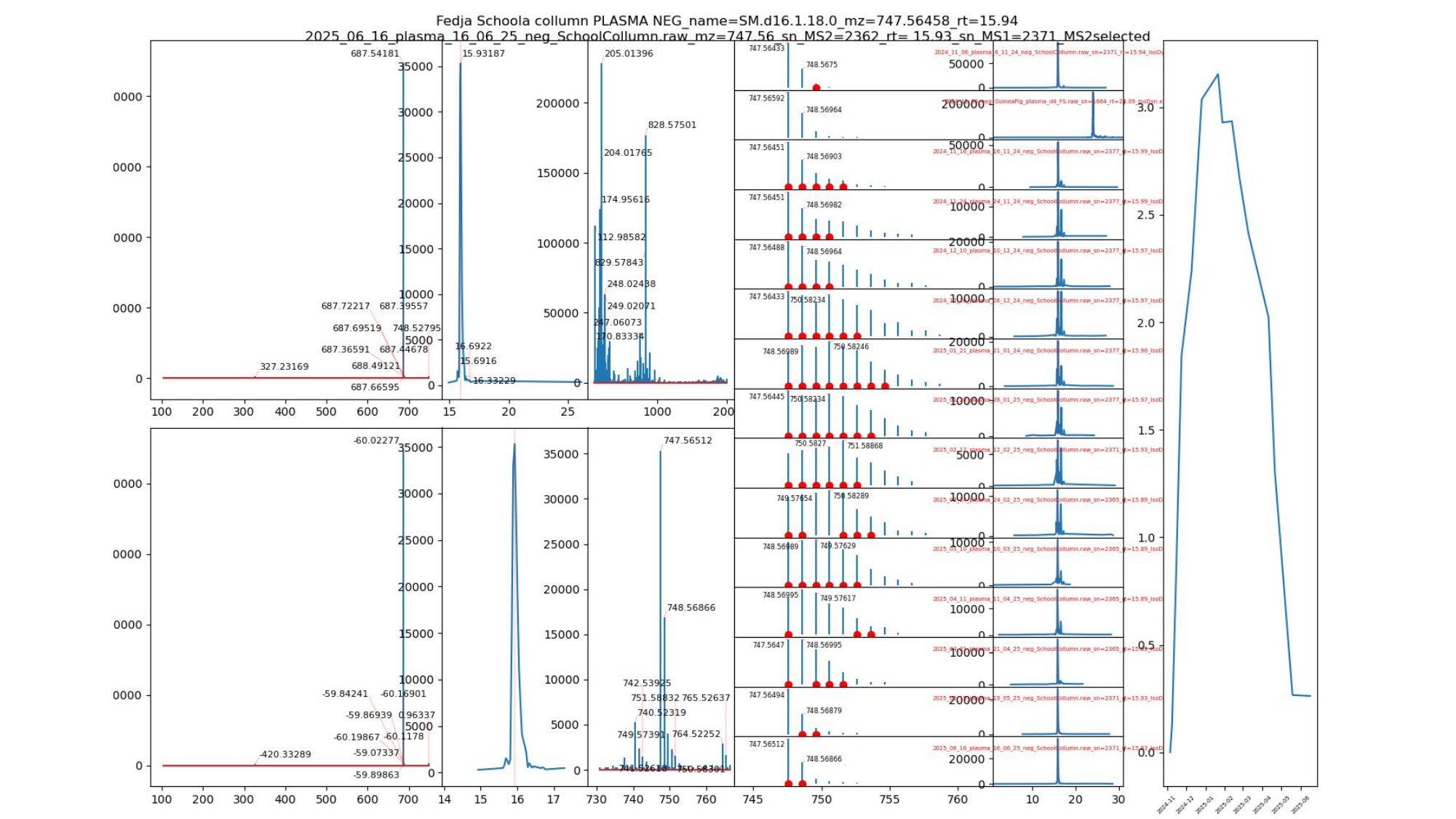

## Slide 68
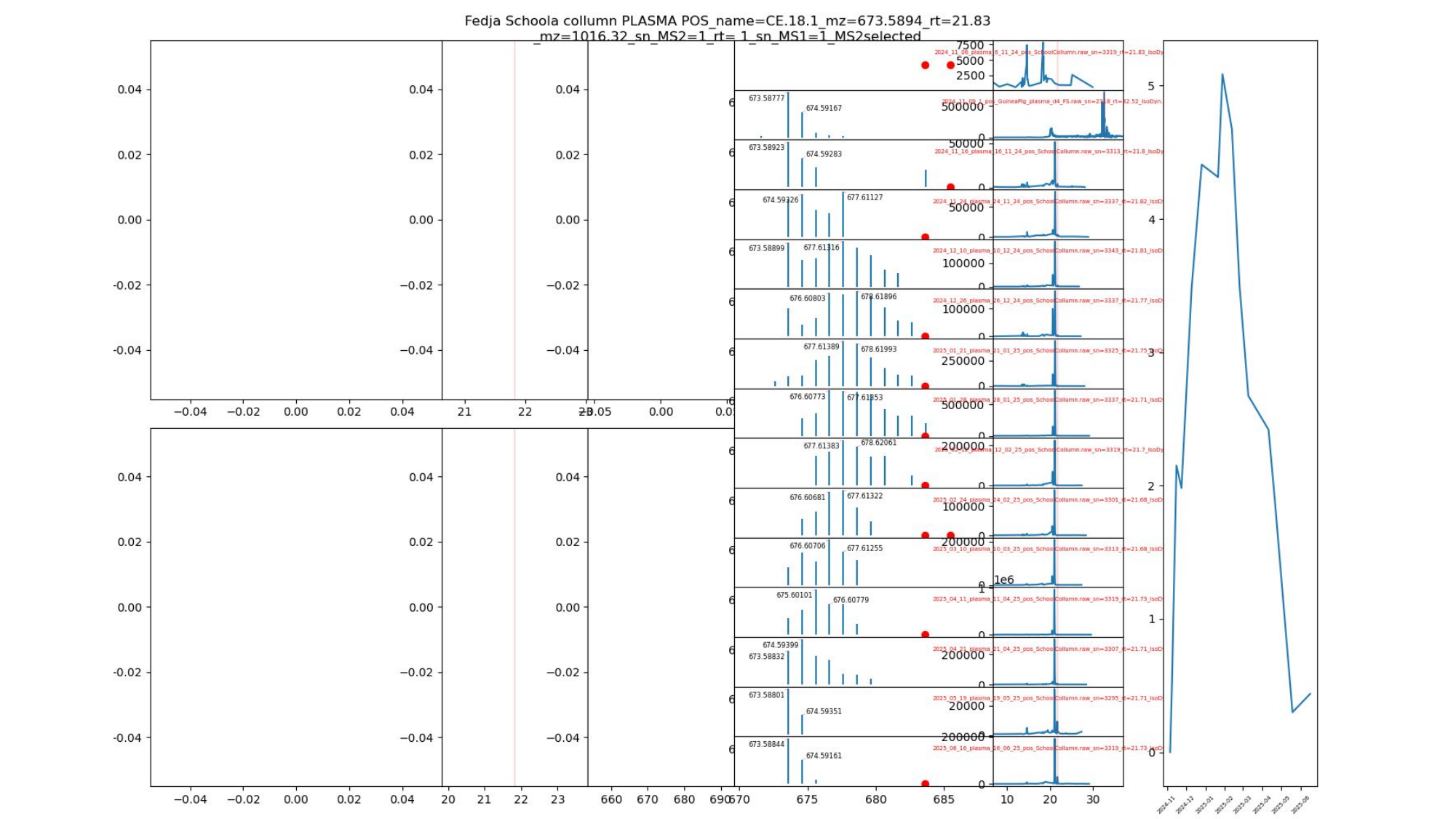

## Slide 69
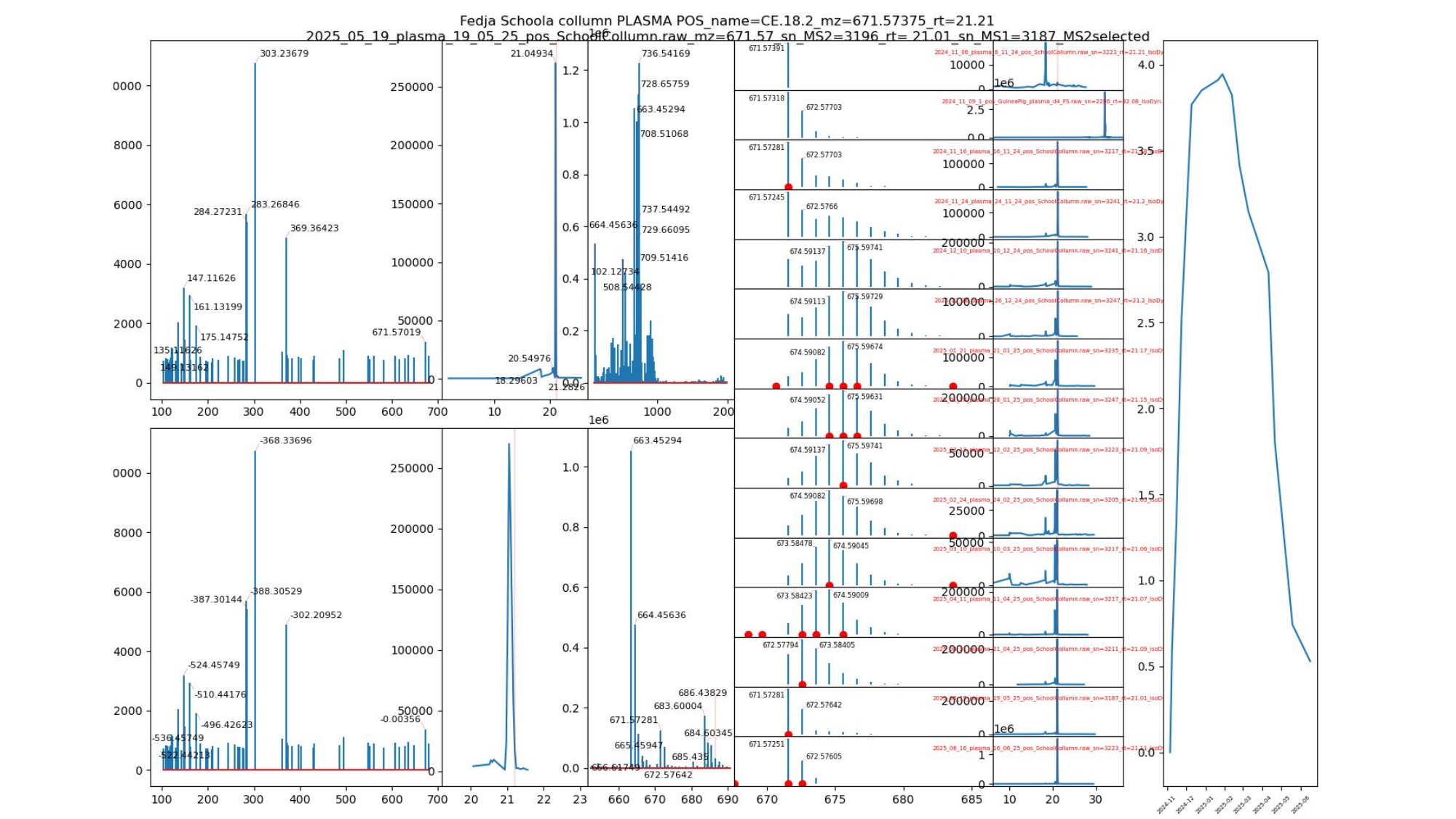

## Slide 70
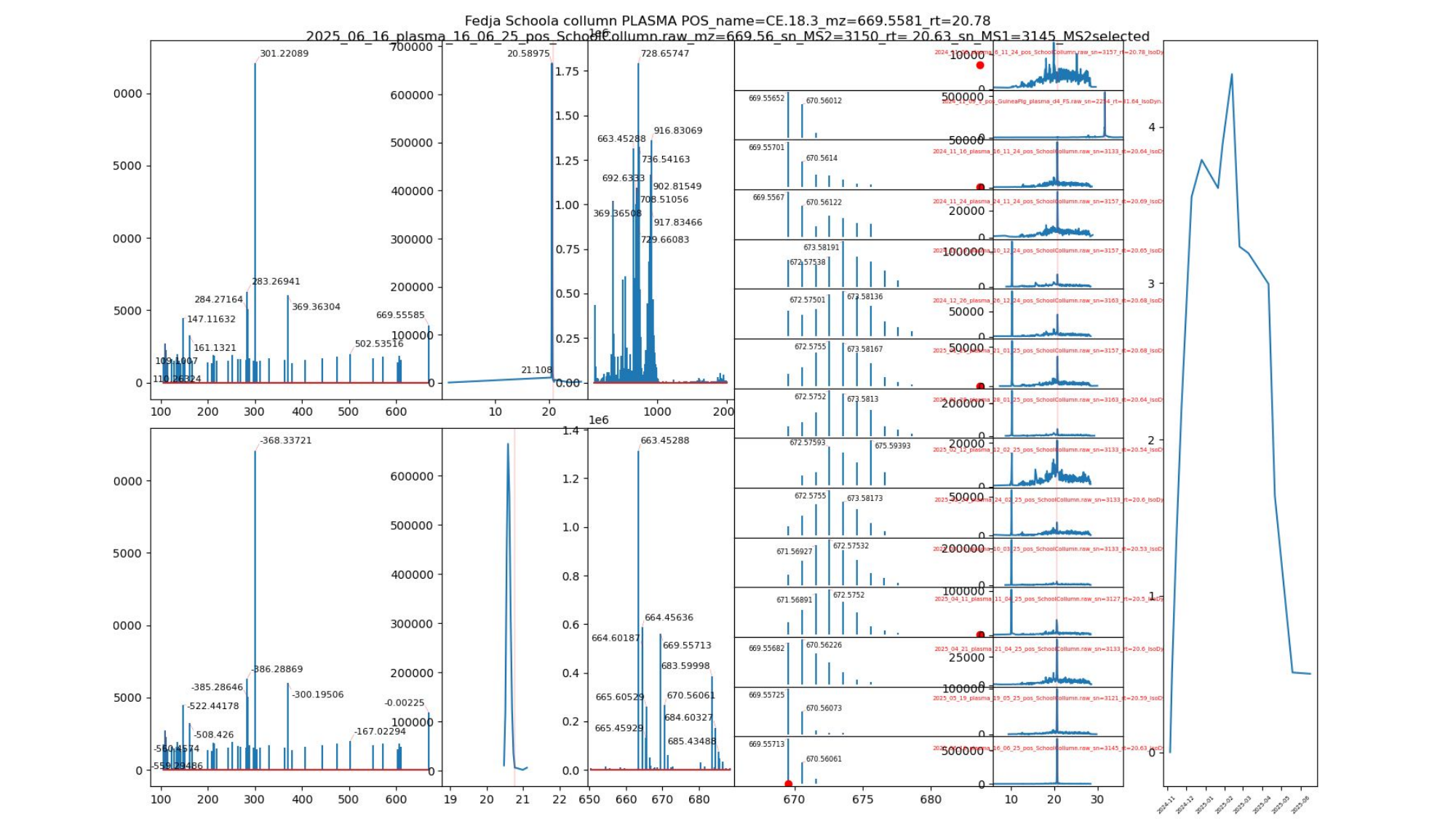

## Slide 71
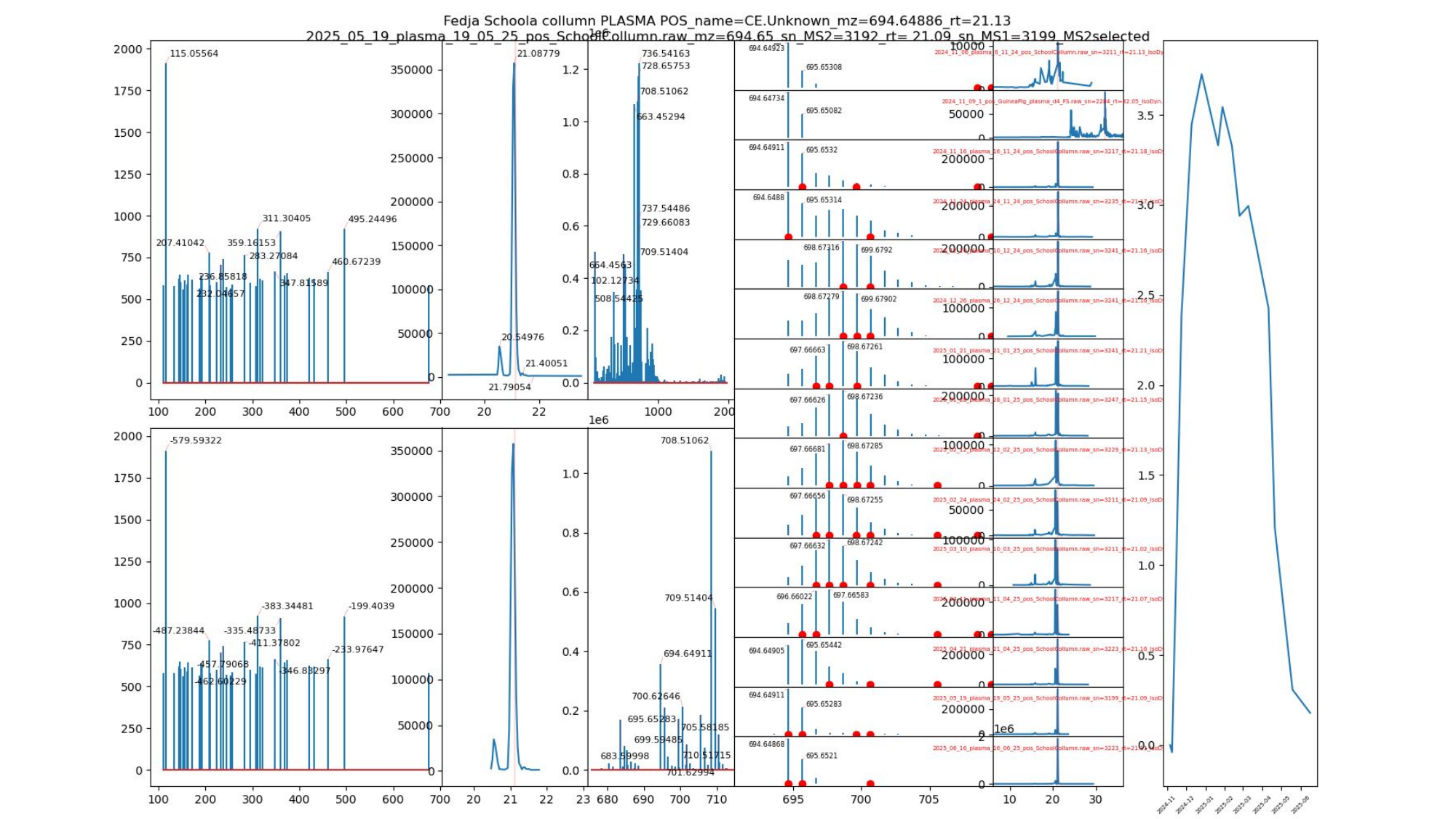

## Slide 72
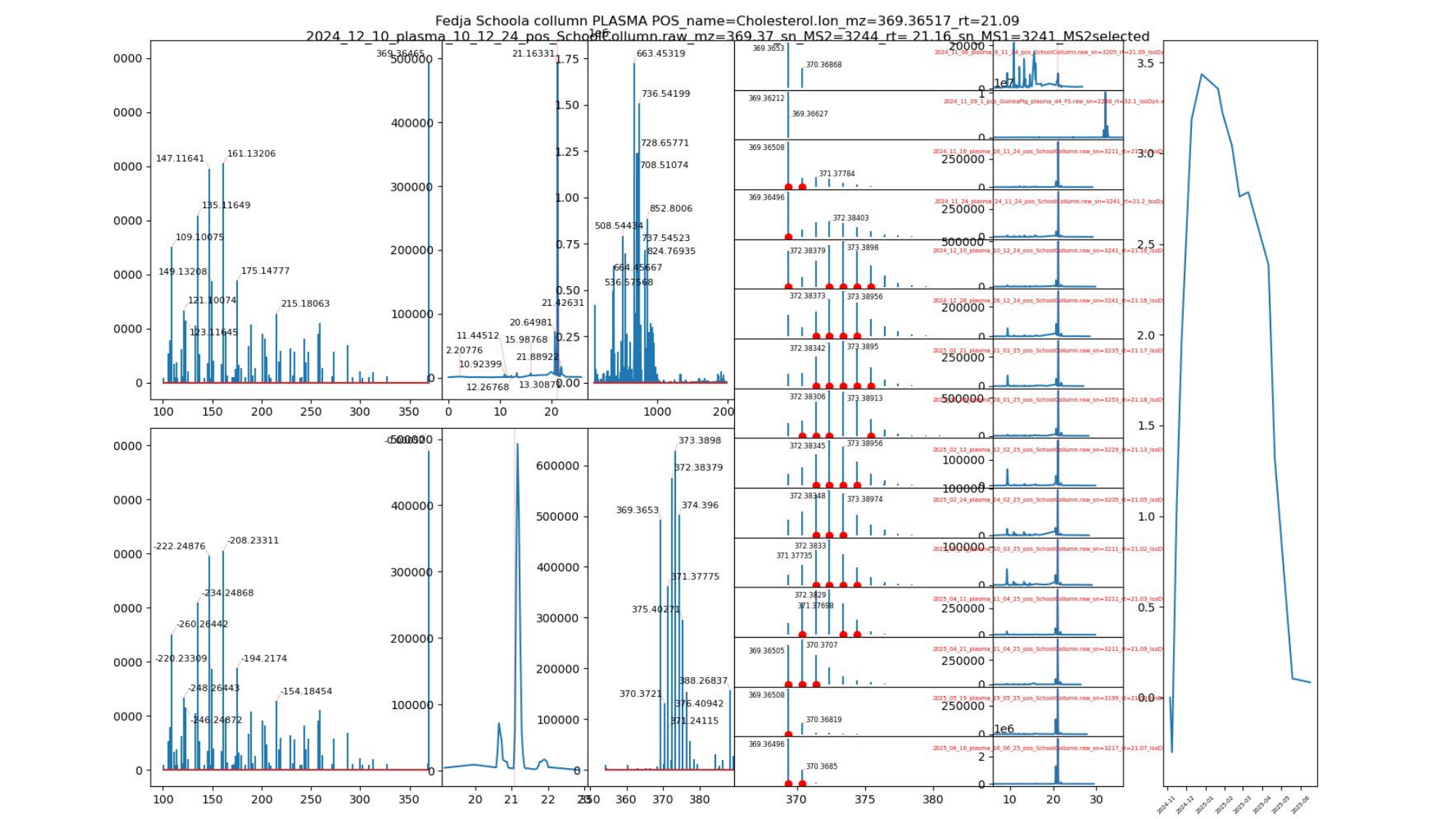

## Slide 73
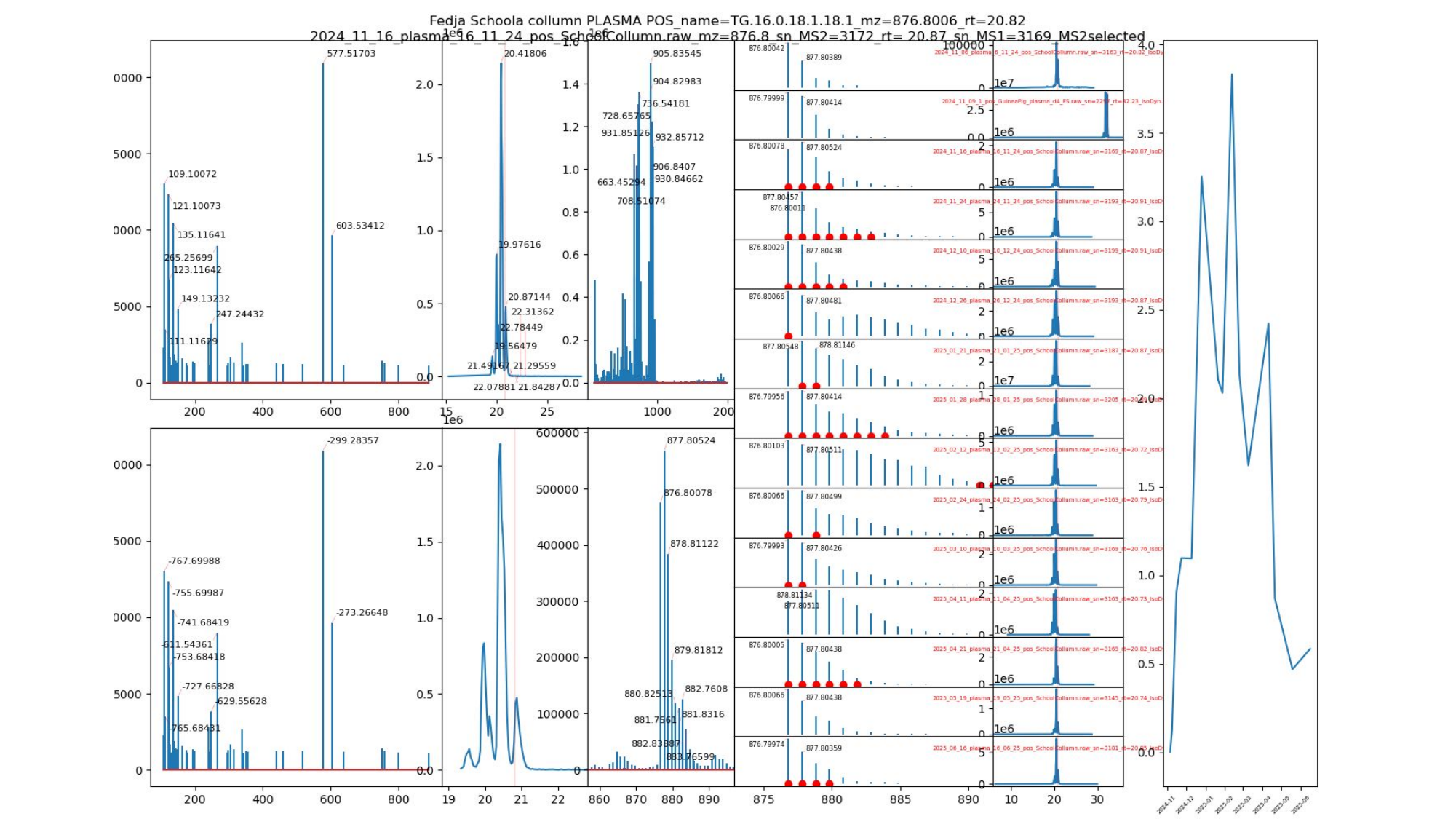

## Slide 74
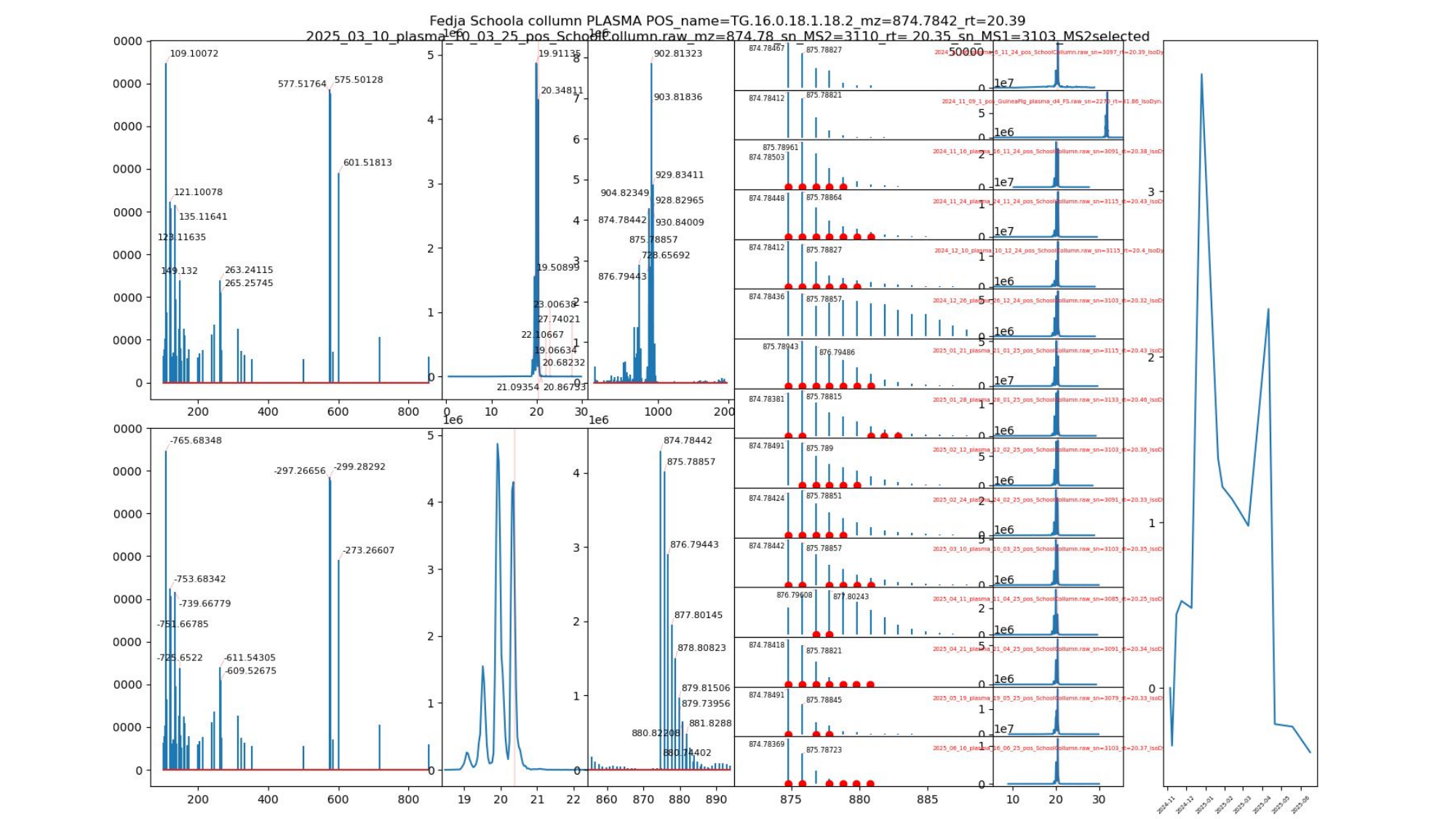

## Slide 75
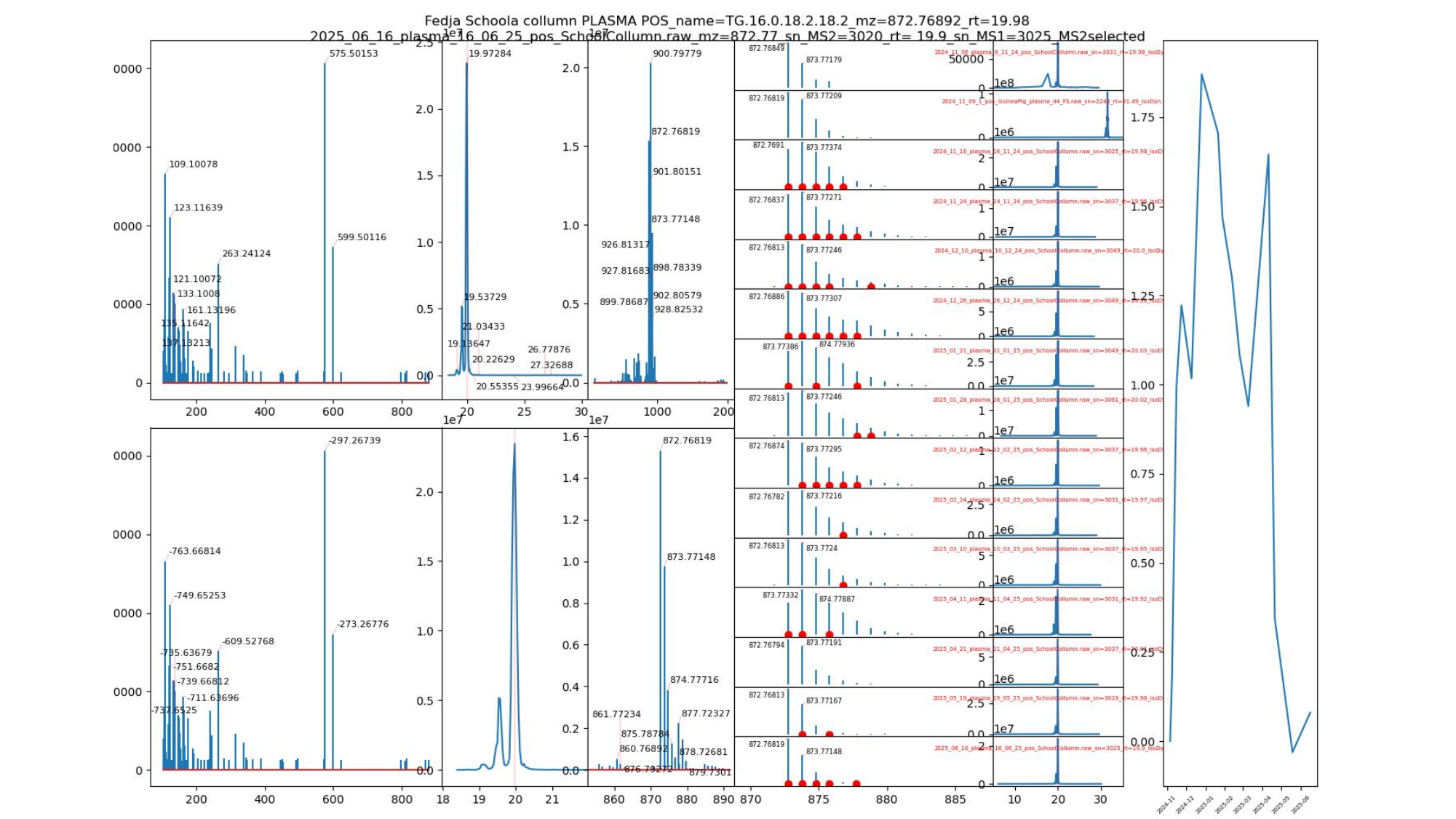

## Slide 76
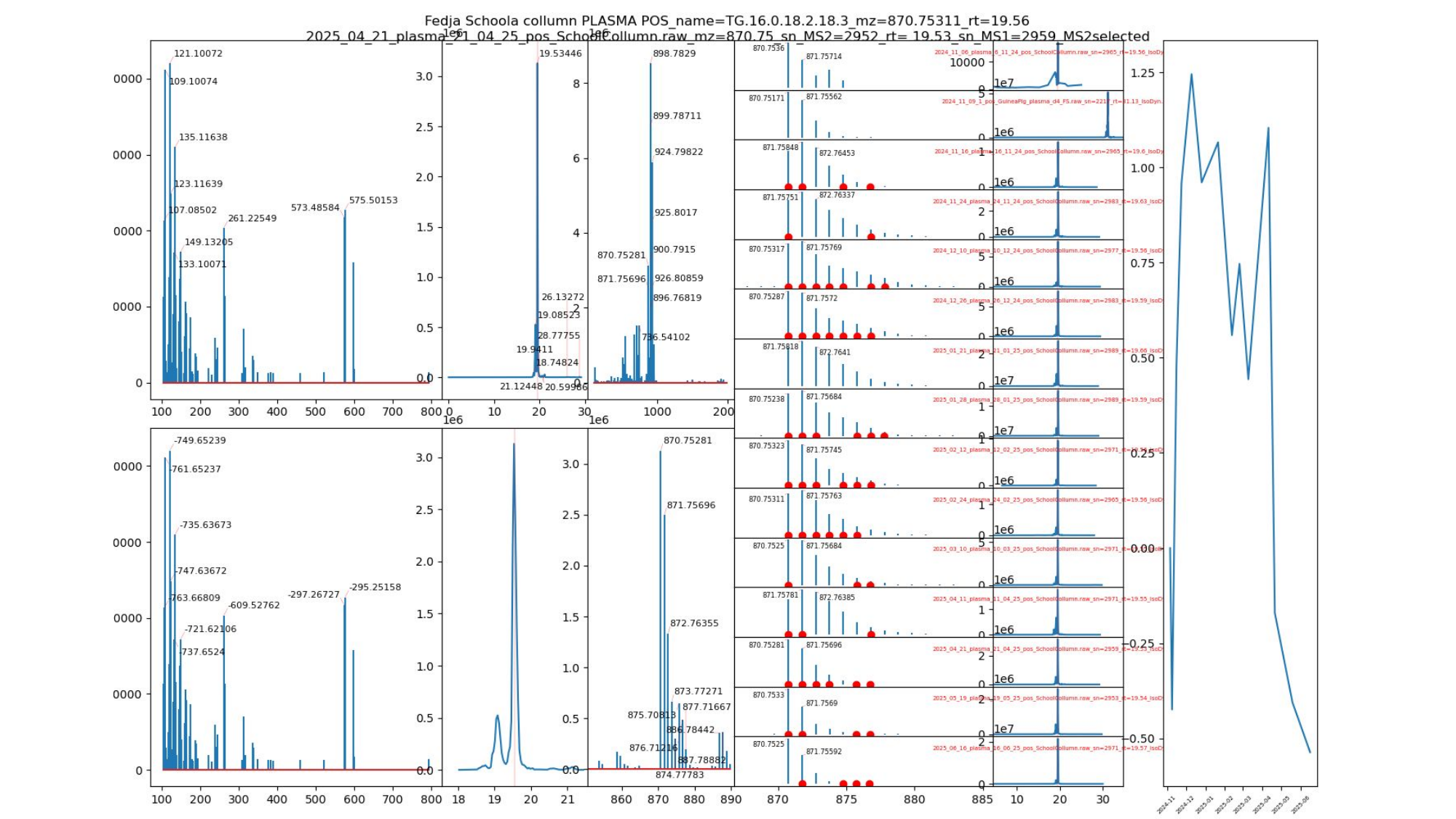

## Slide 77
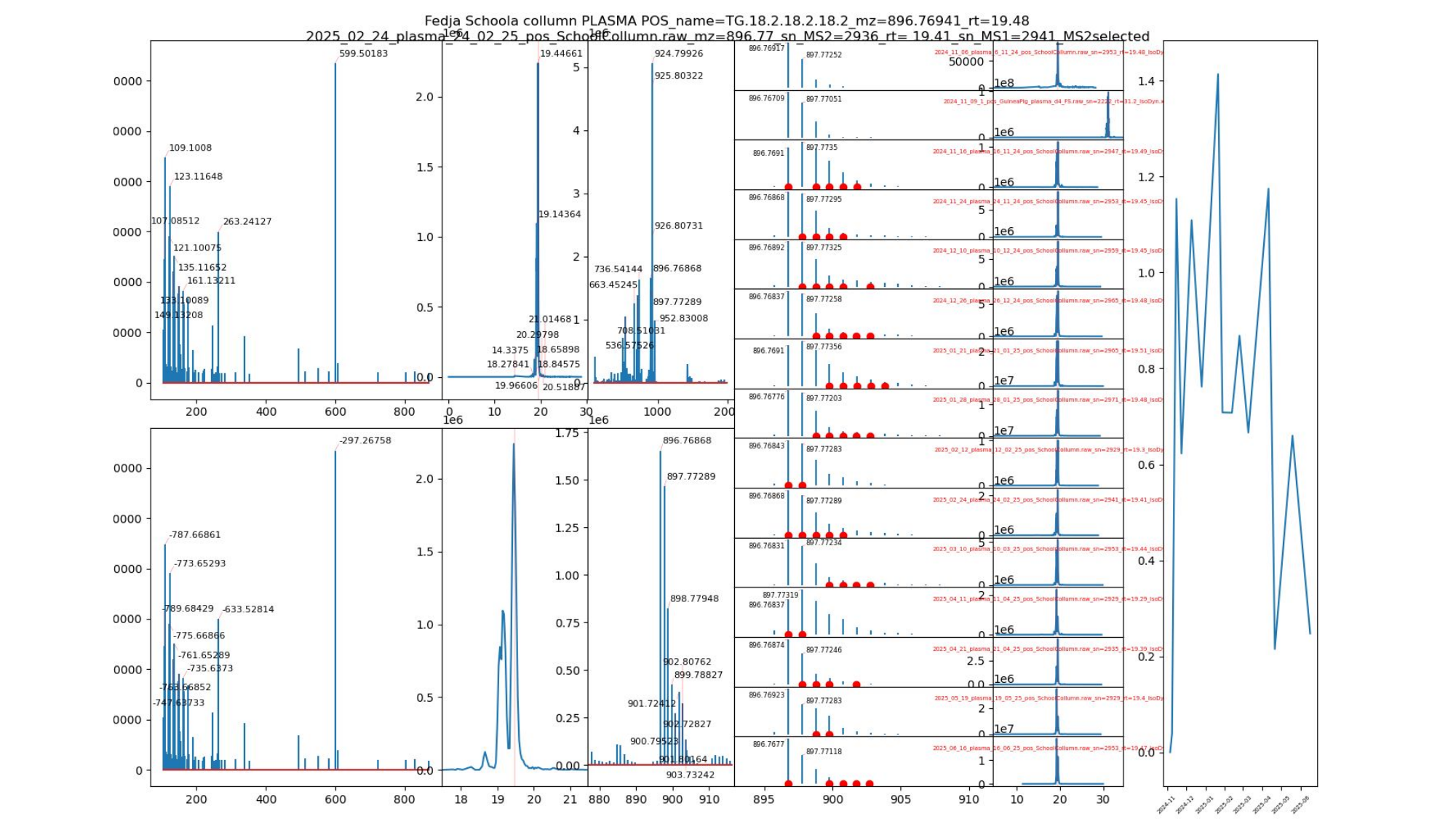

## Slide 78
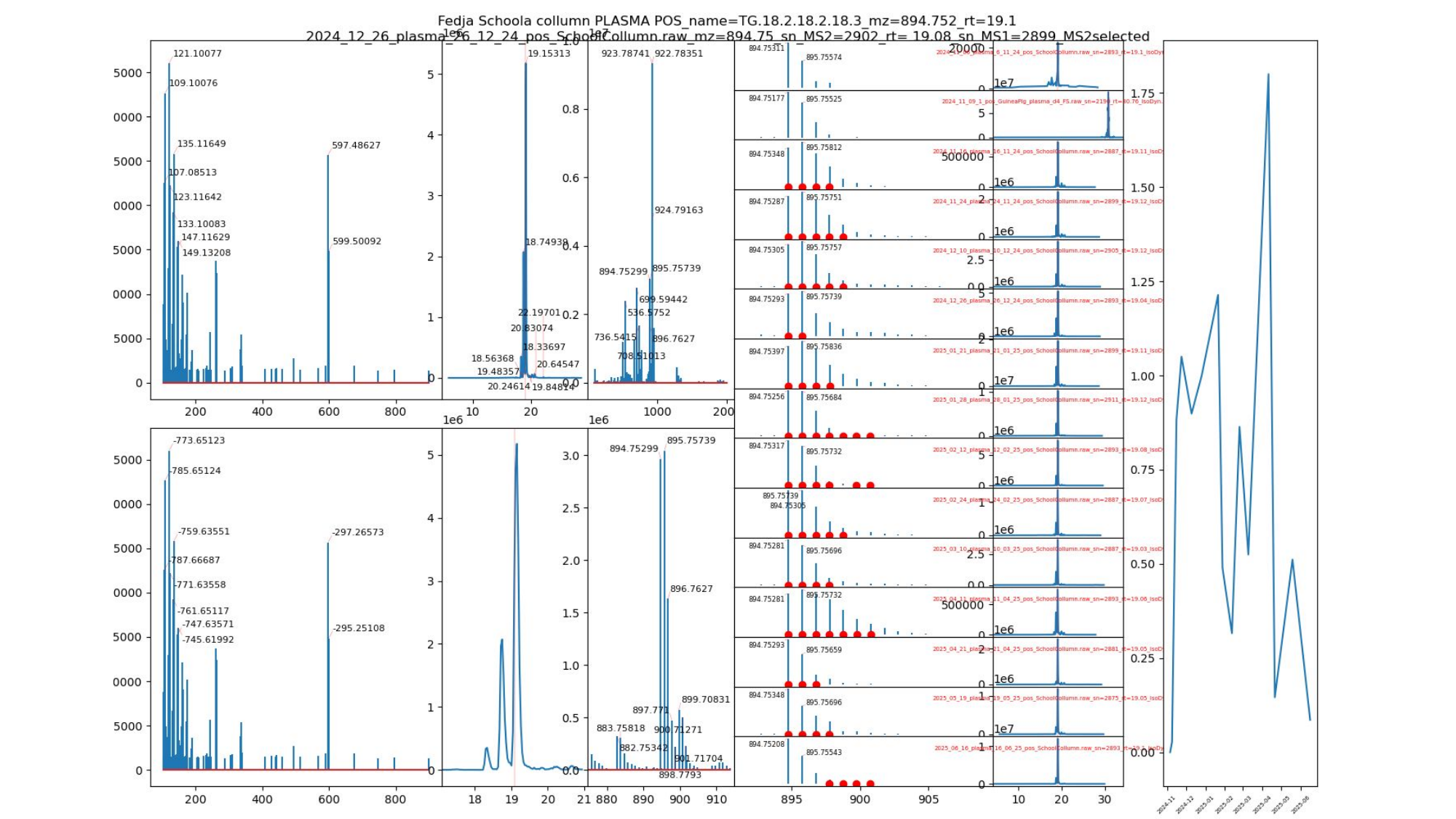

## Slide 79
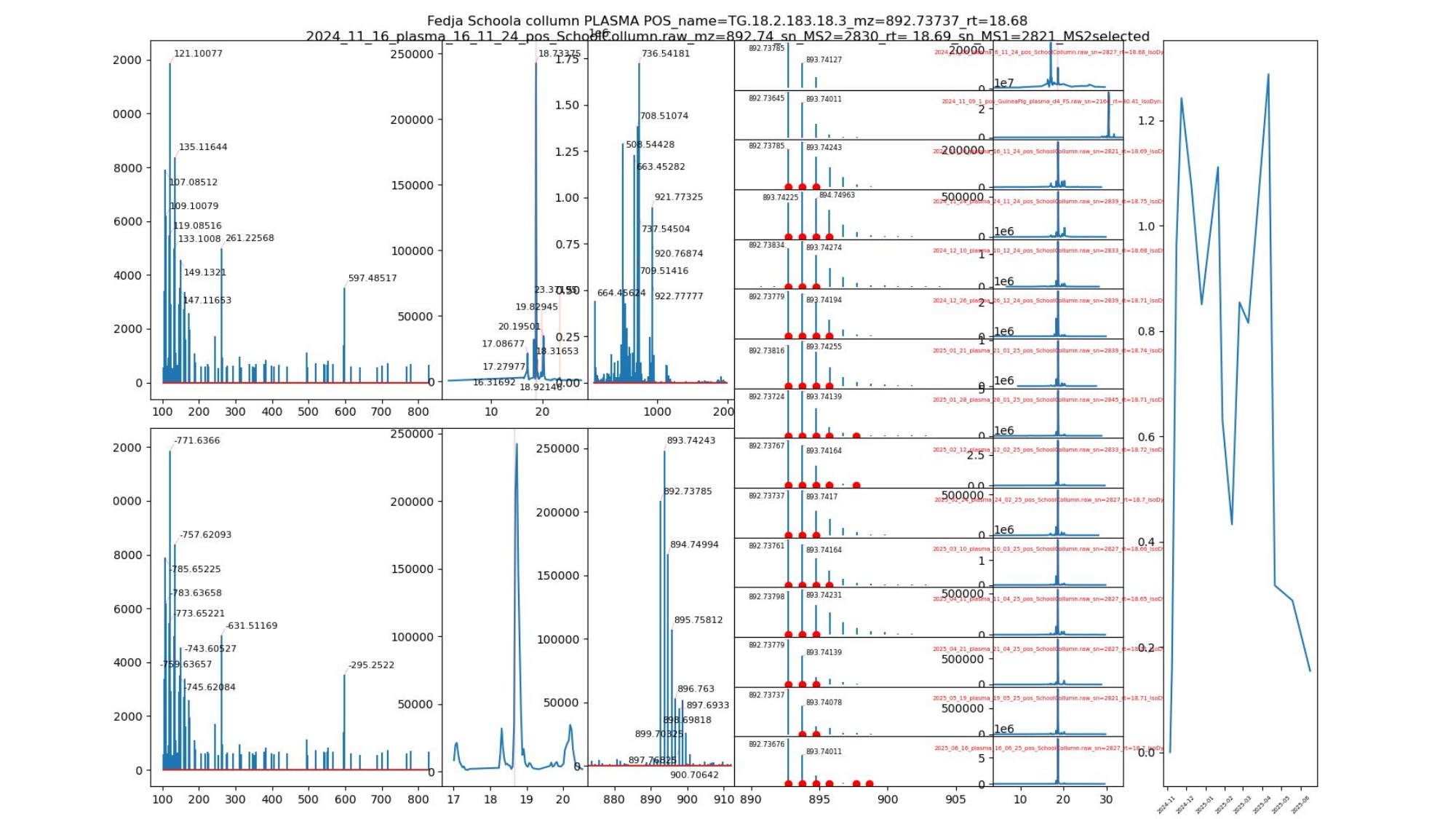

## Slide 80
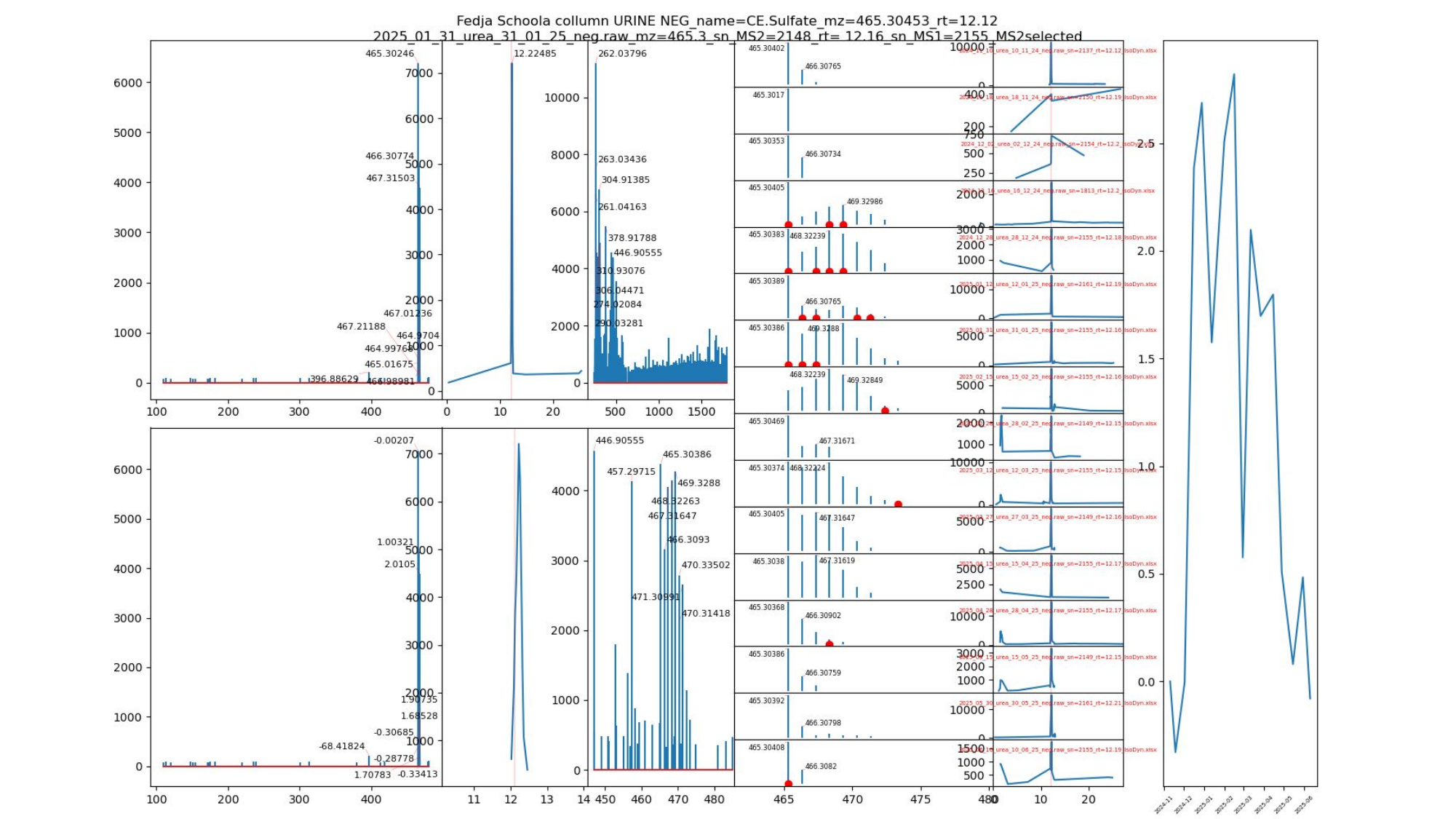

## Slide 81
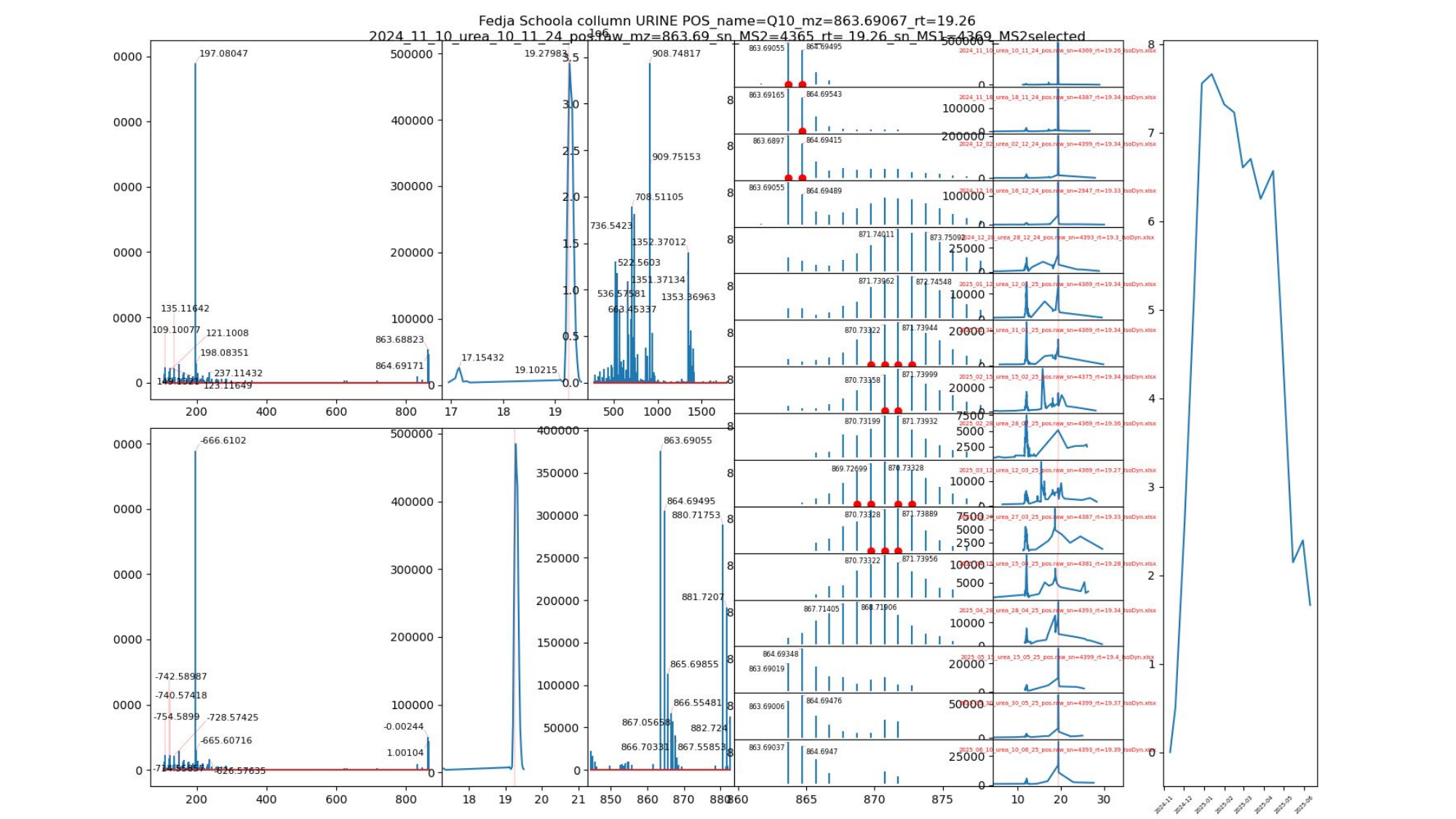

## Slide 82
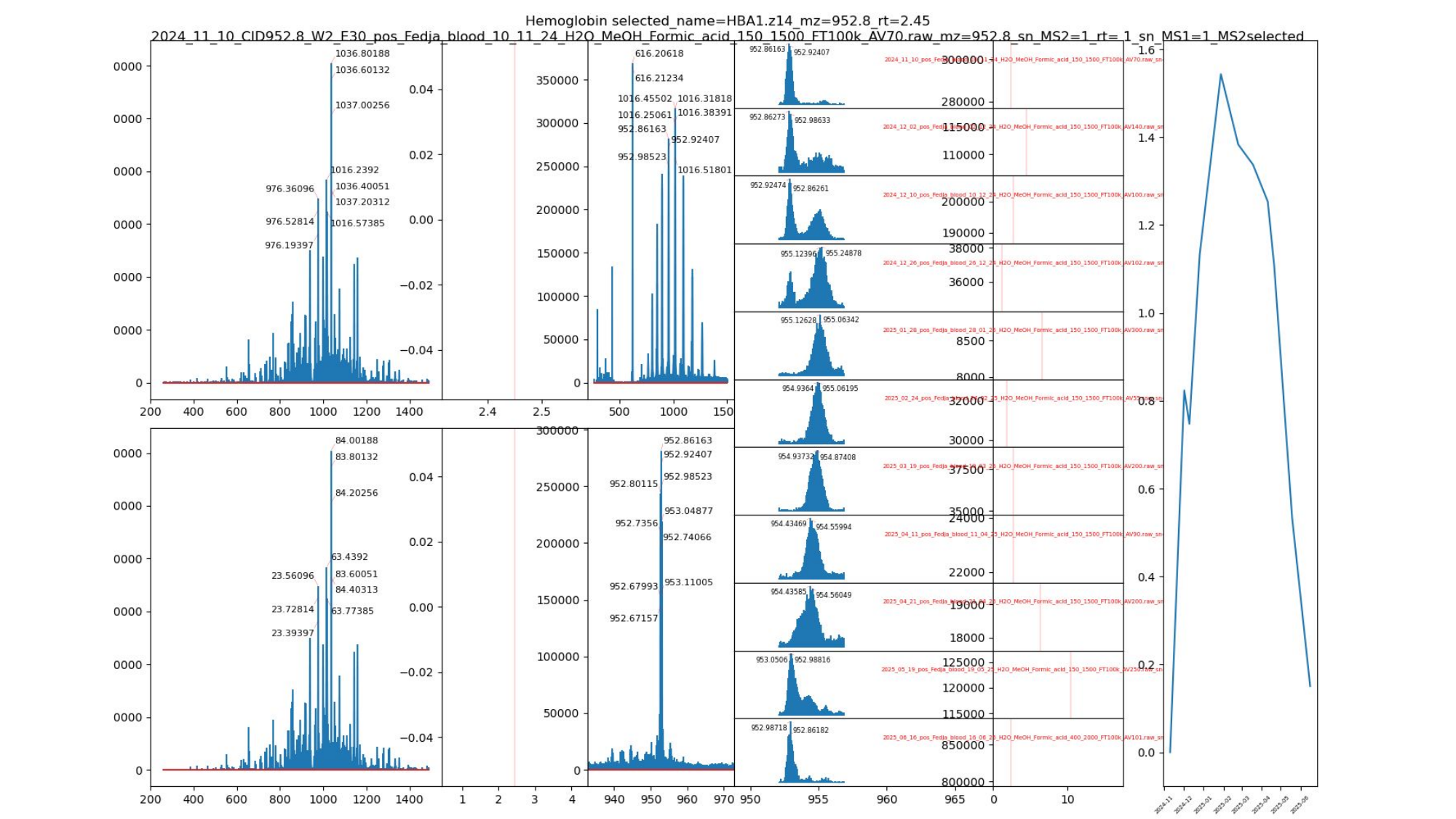

## Slide 83
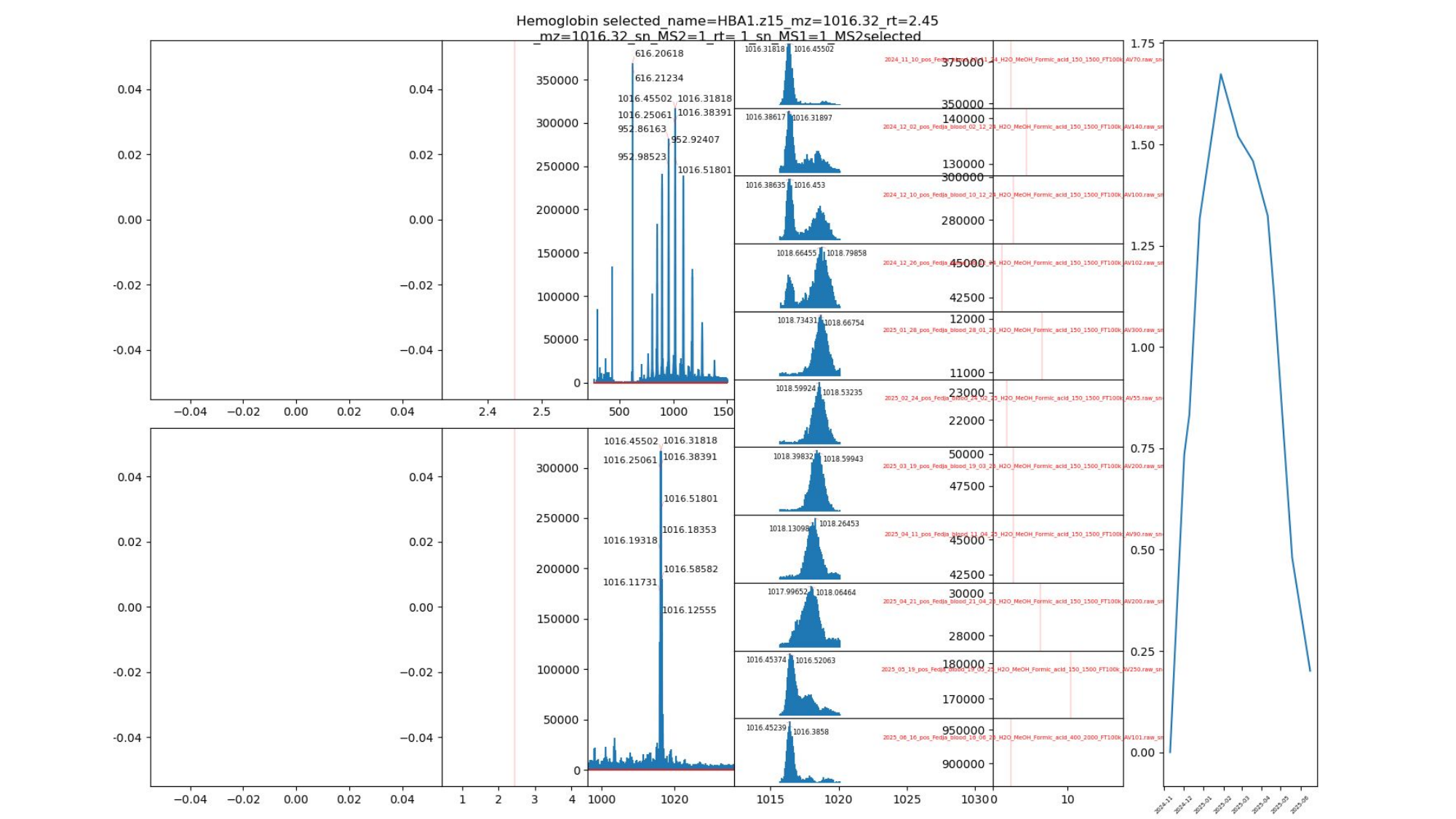

## Slide 84
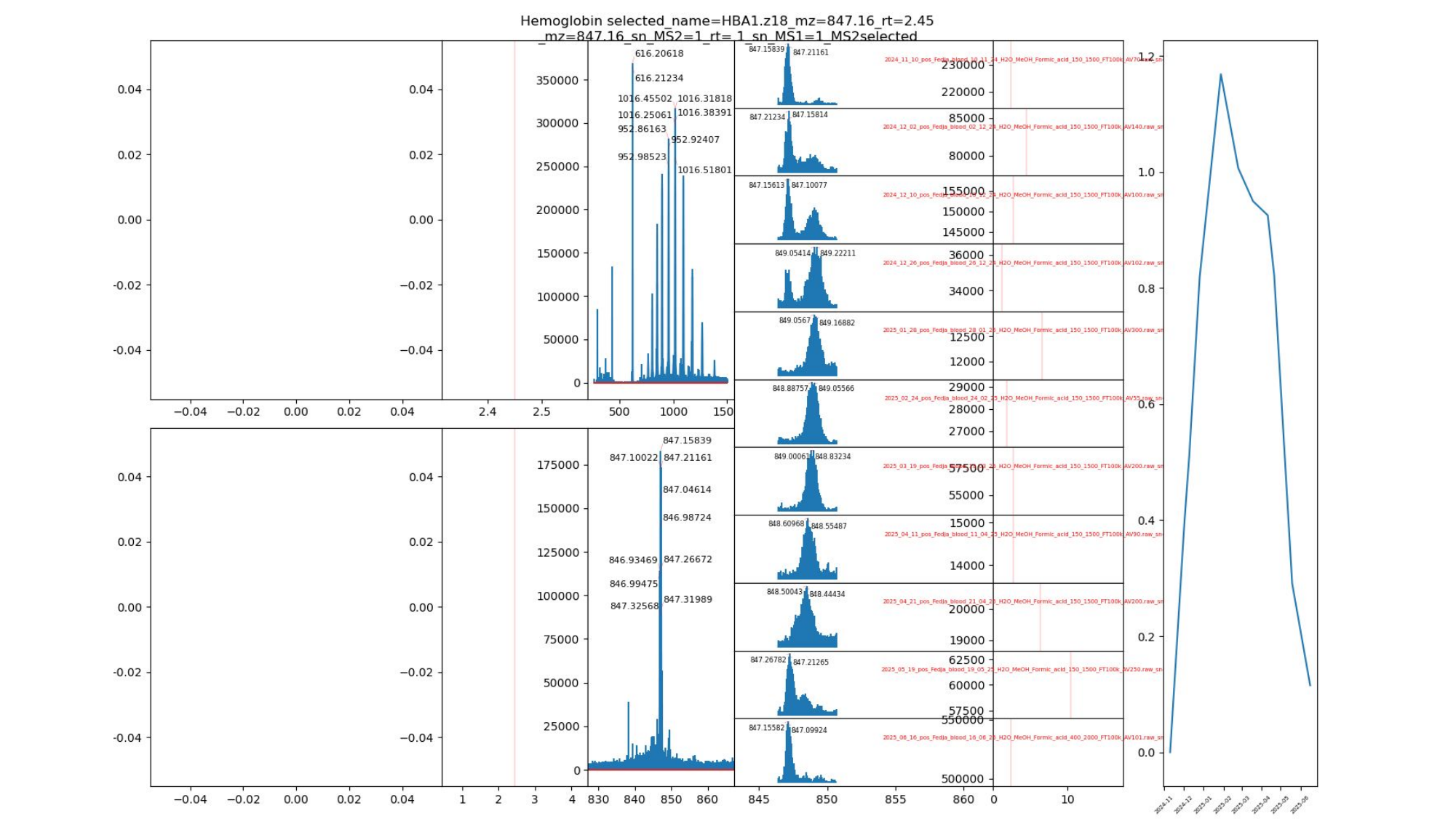

## Slide 85
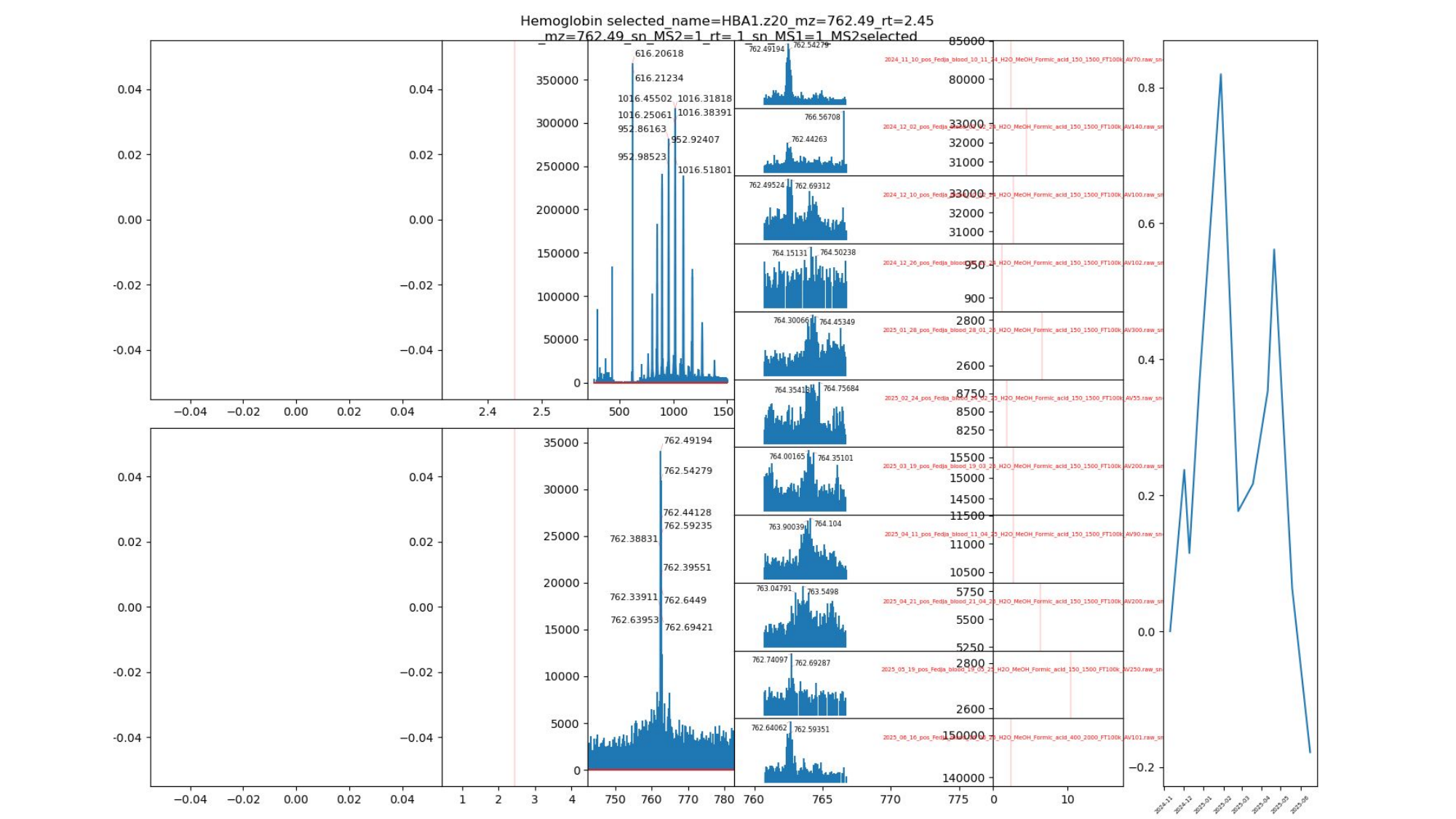

## Slide 86
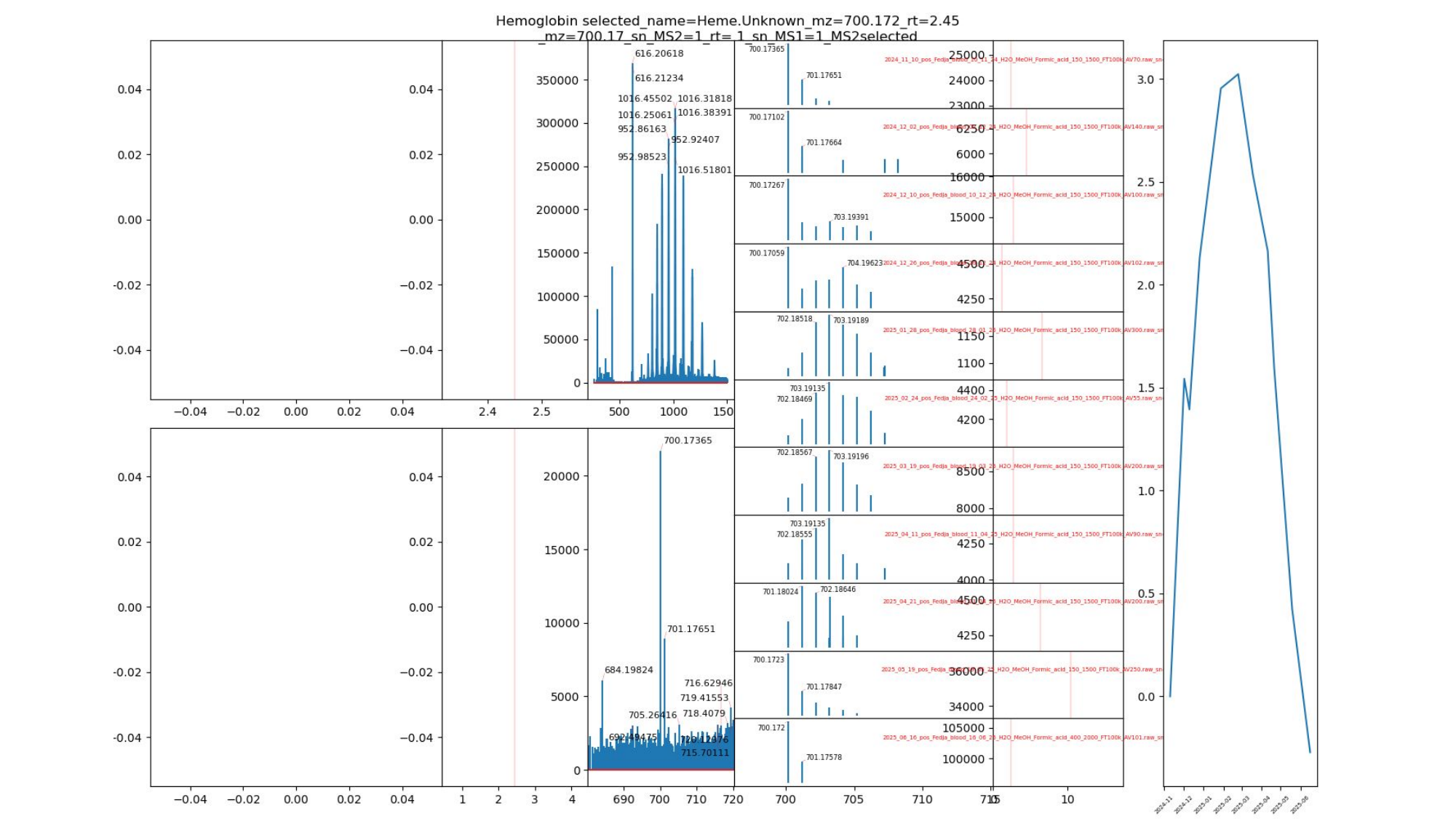

## Slide 87
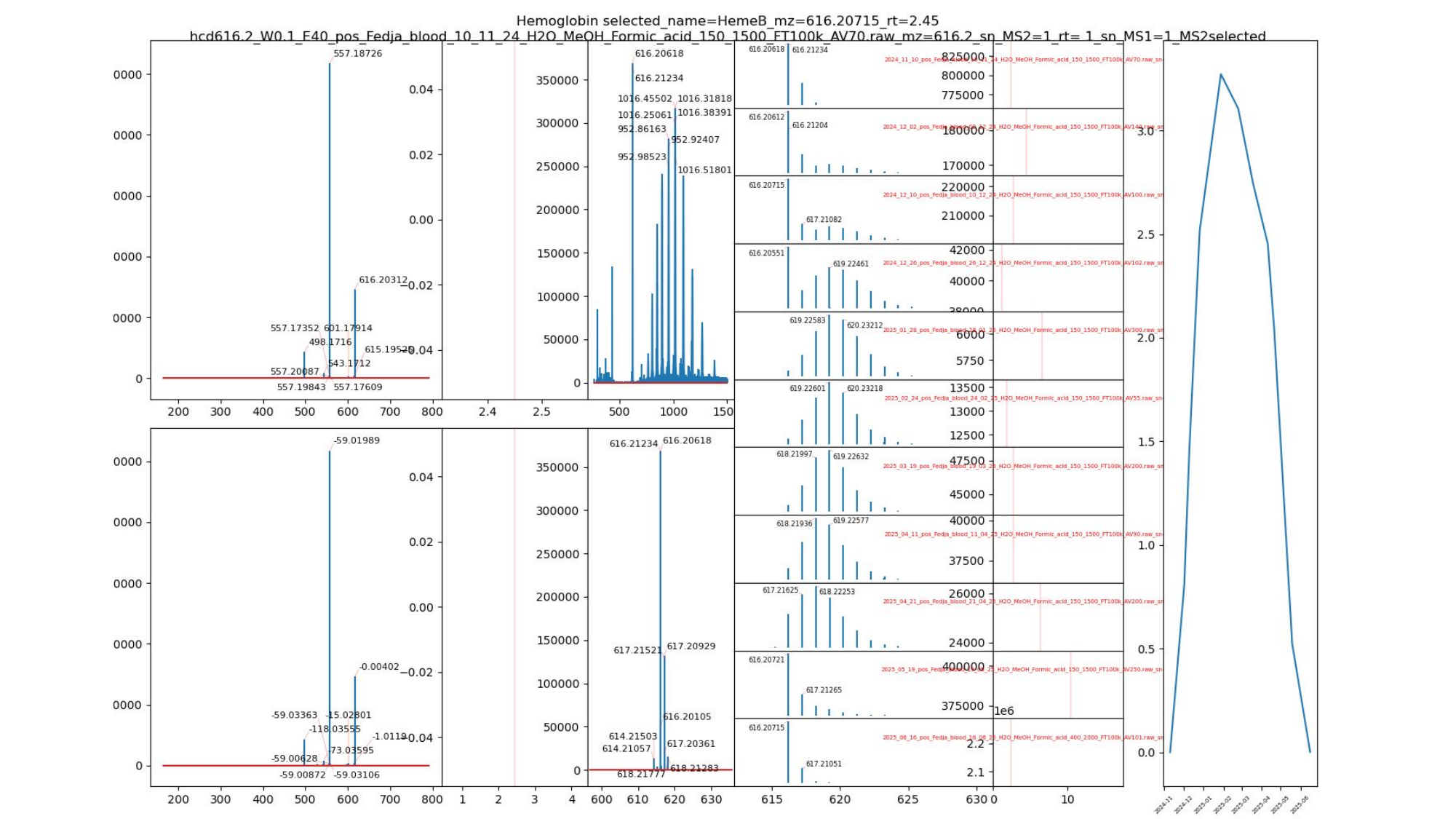

## Slide 88
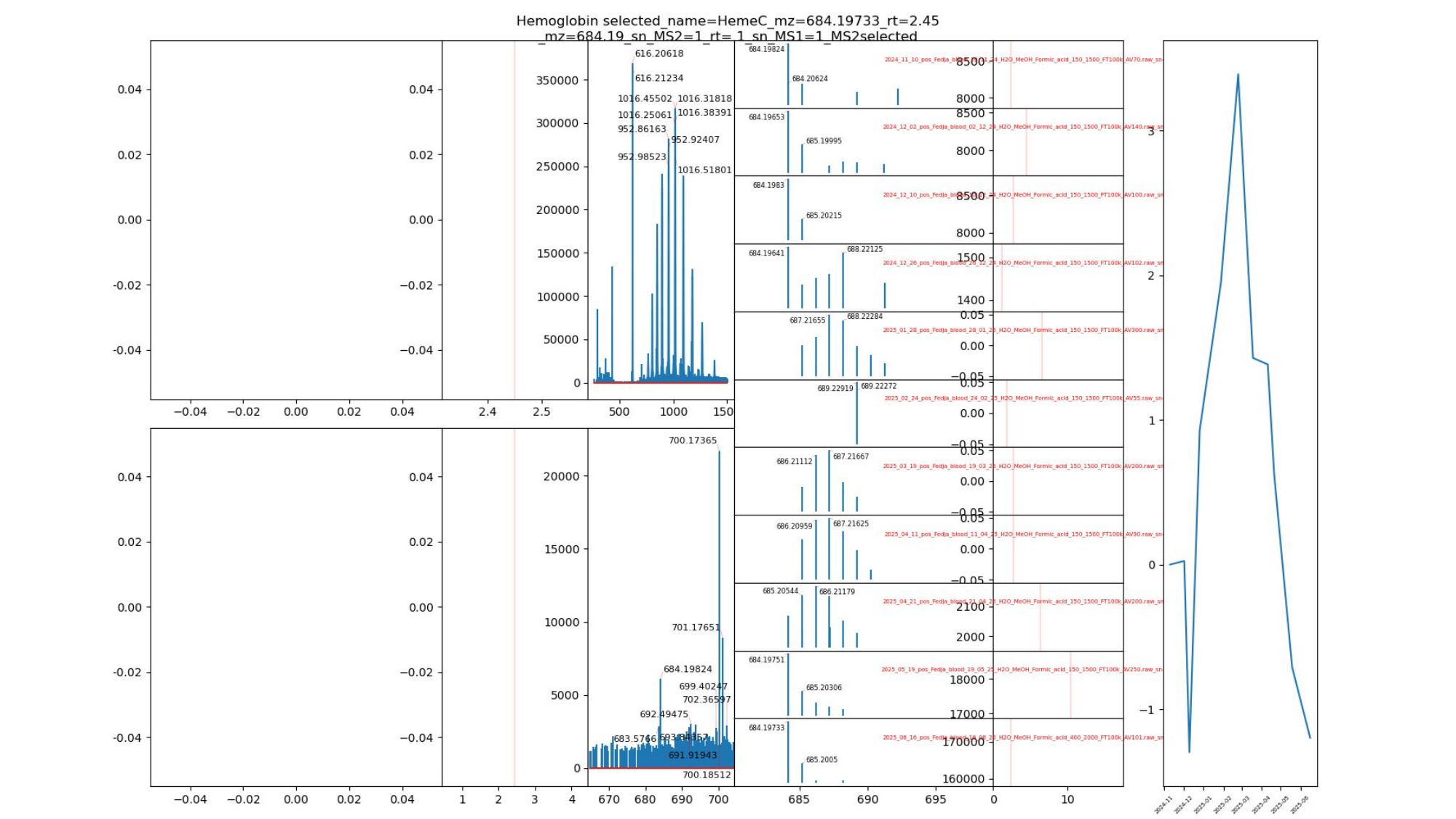

## Slide 89
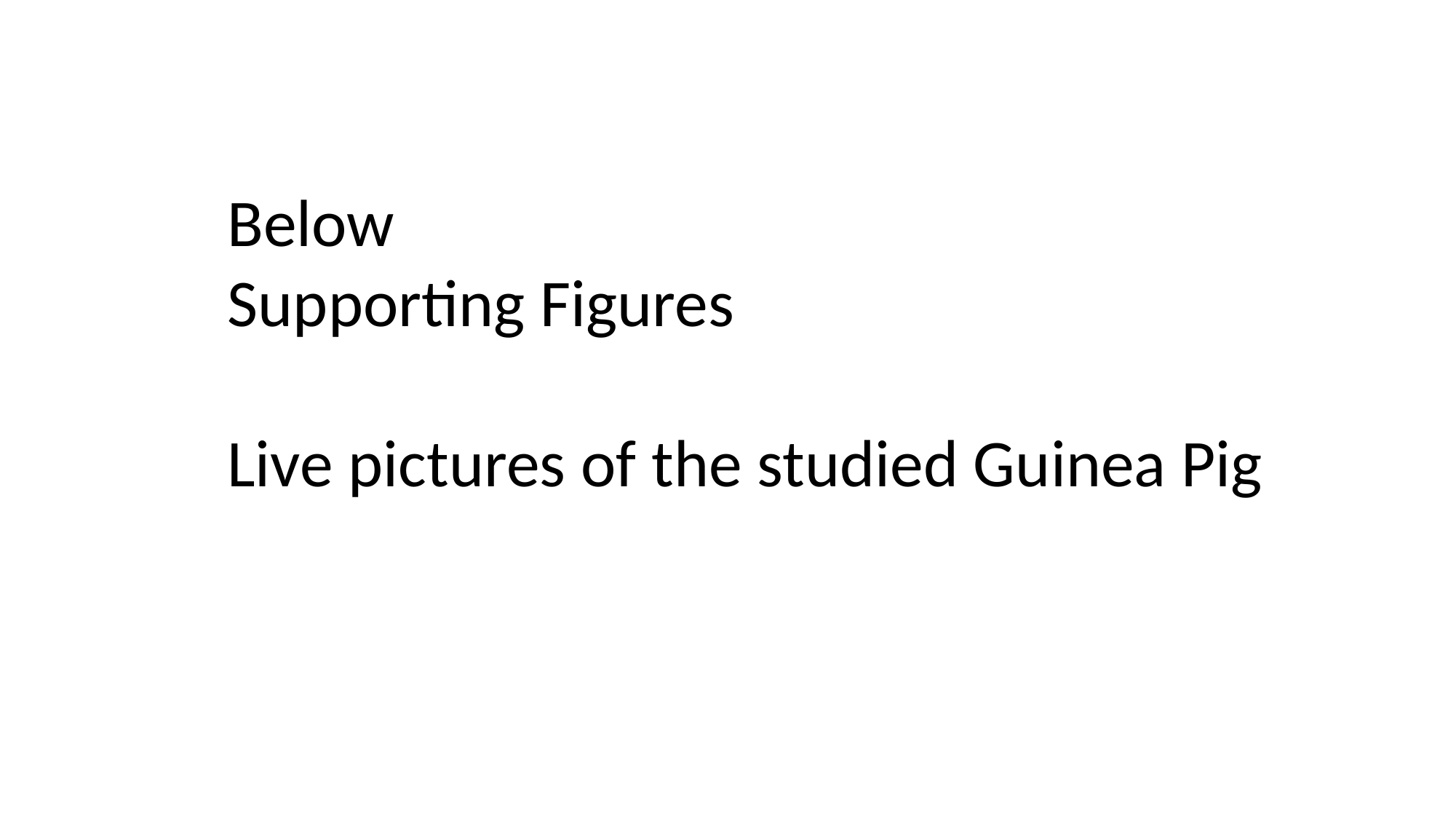

Below
Supporting Figures
Live pictures of the studied Guinea Pig

## Slide 90
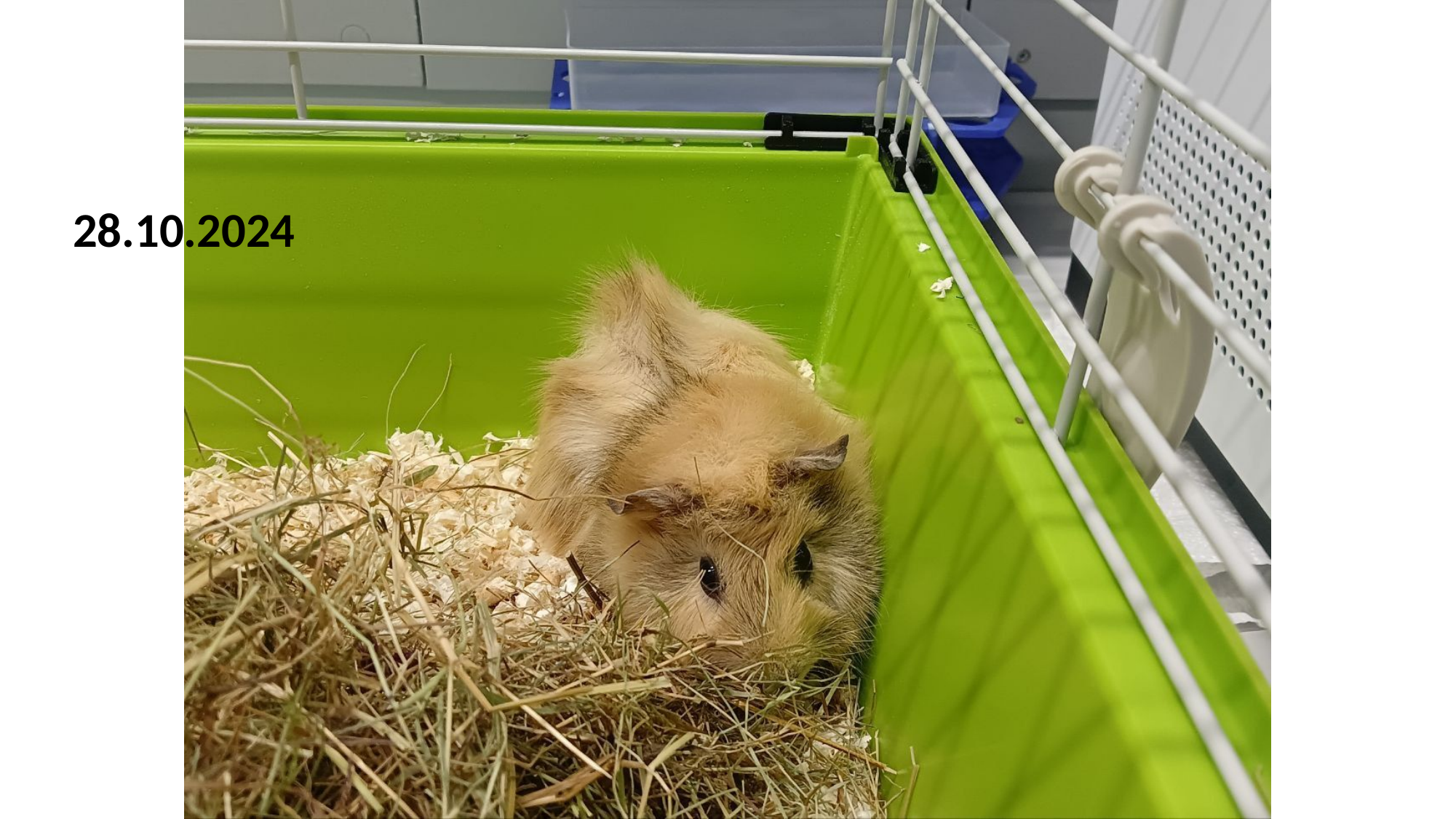

28.10.2024

## Slide 91
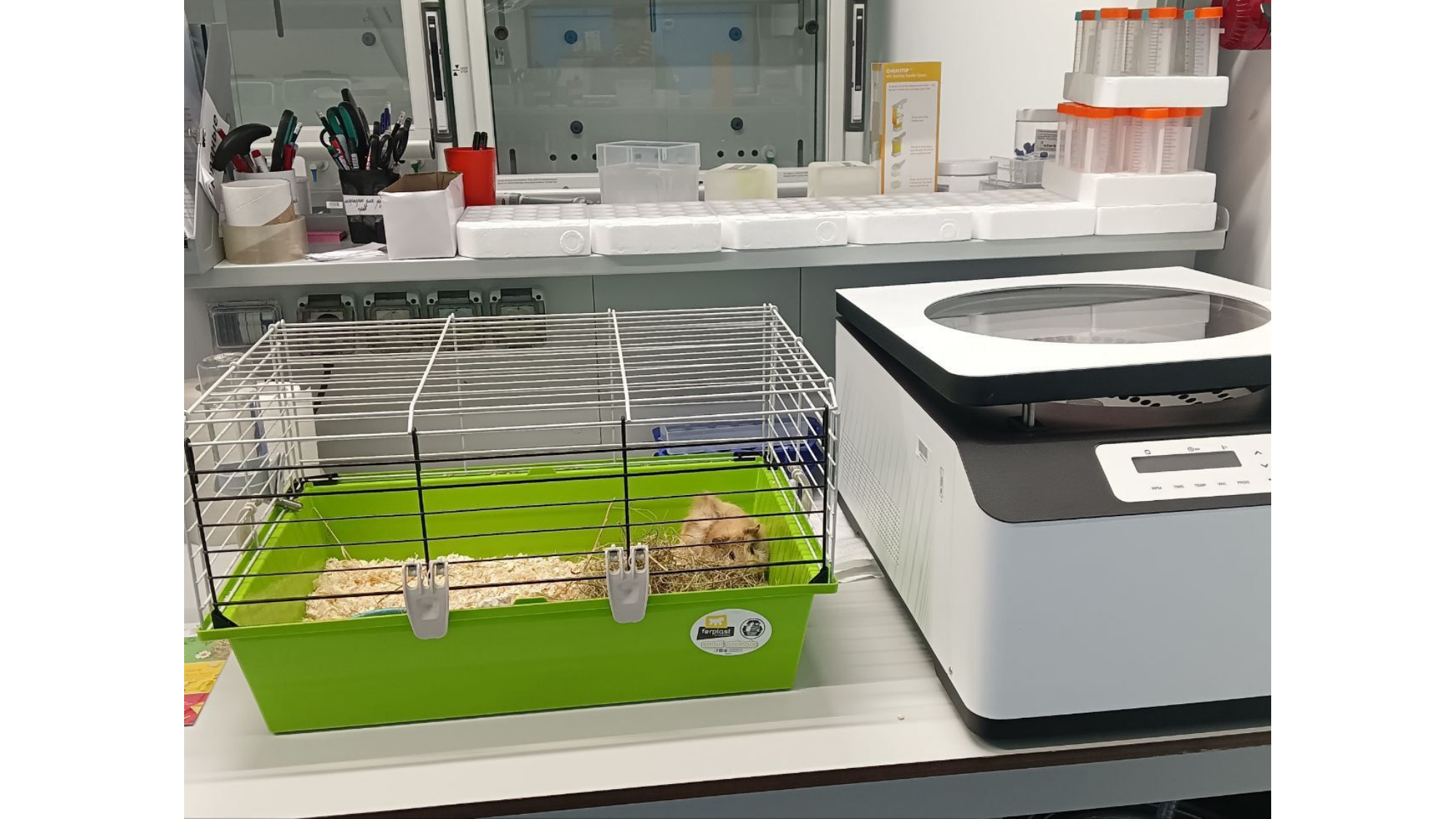

## Slide 92
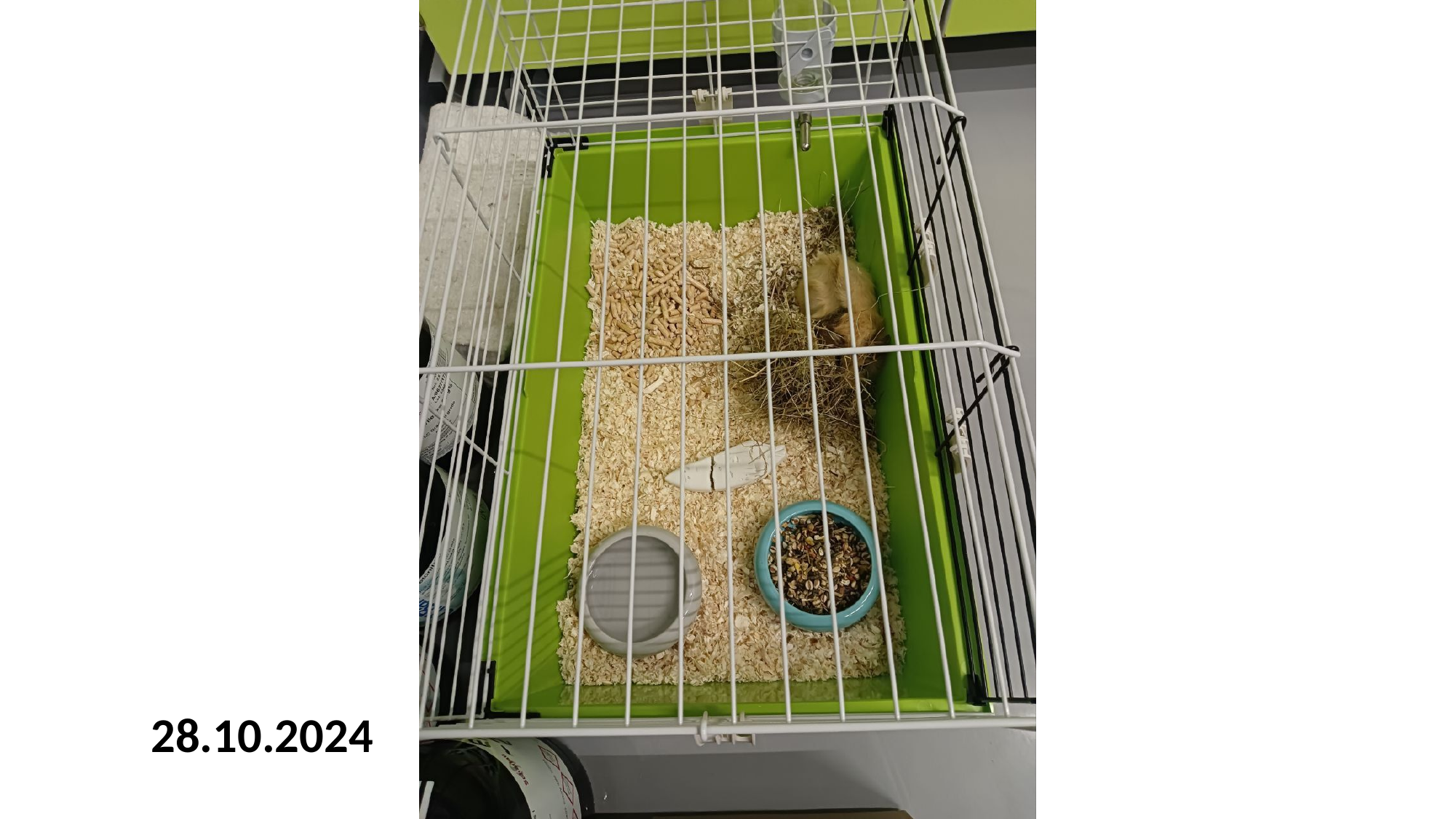

28.10.2024

## Slide 93
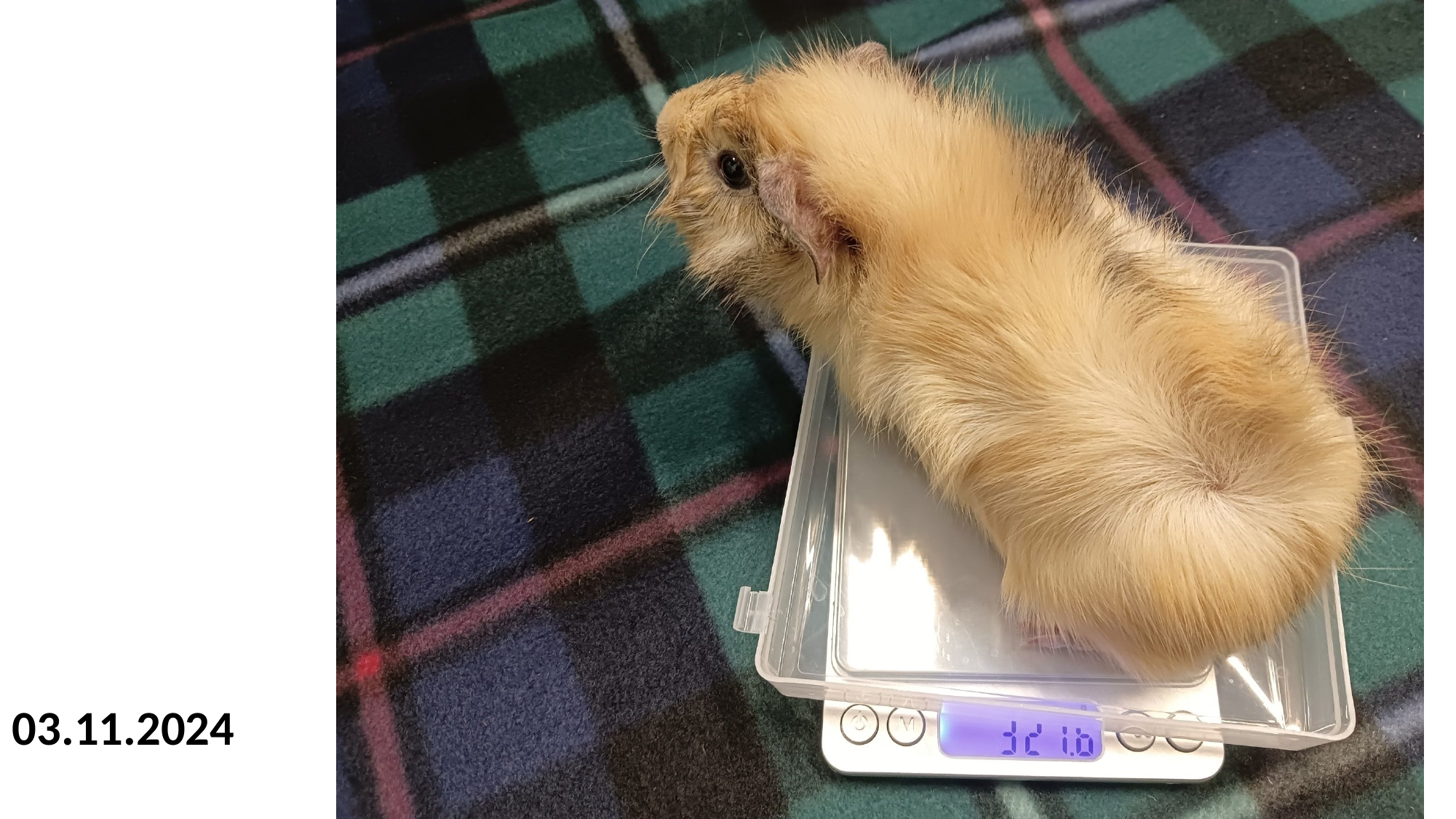

03.11.2024

## Slide 94
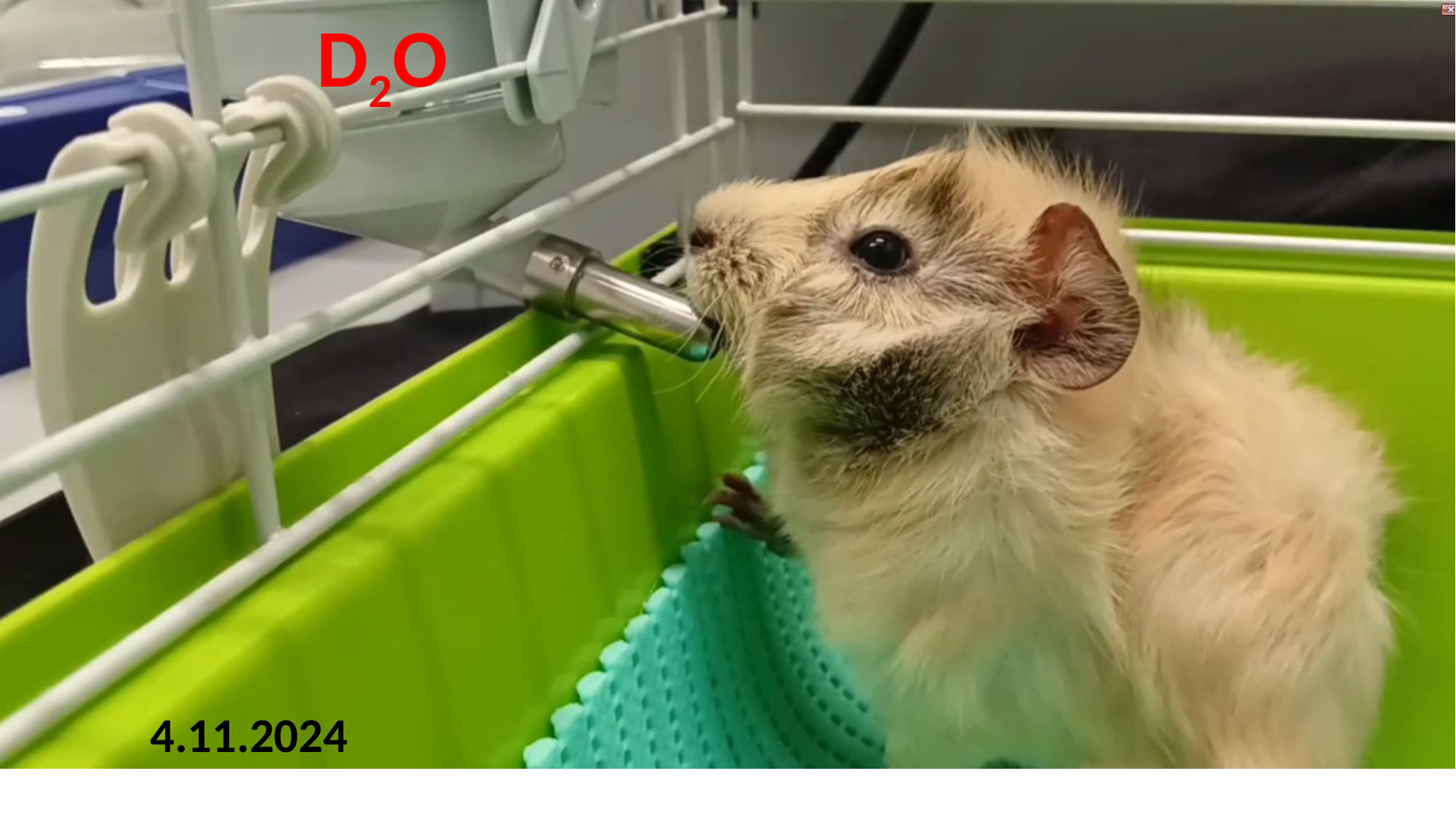

D2O
4.11.2024

## Slide 95
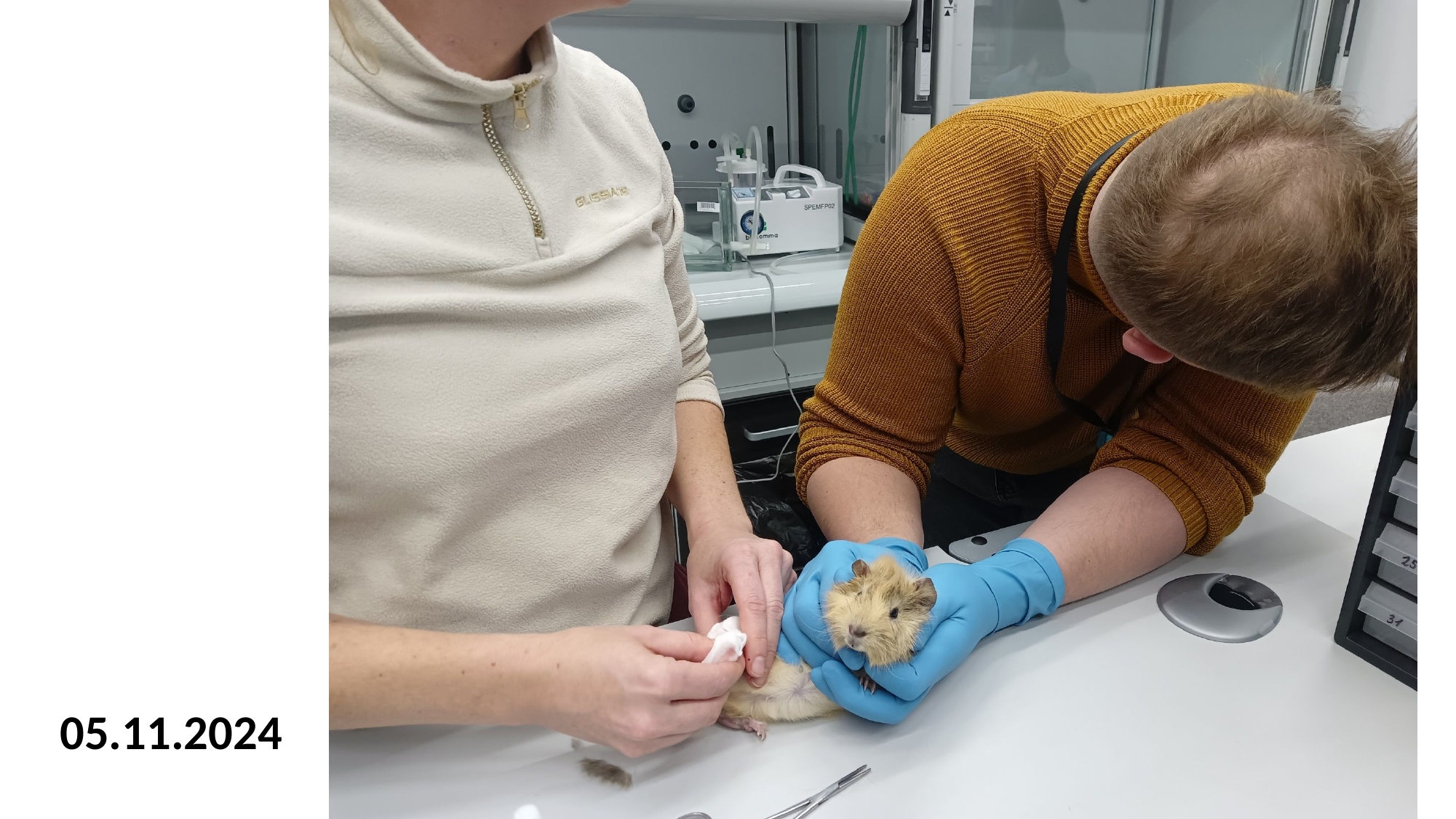

05.11.2024

## Slide 96
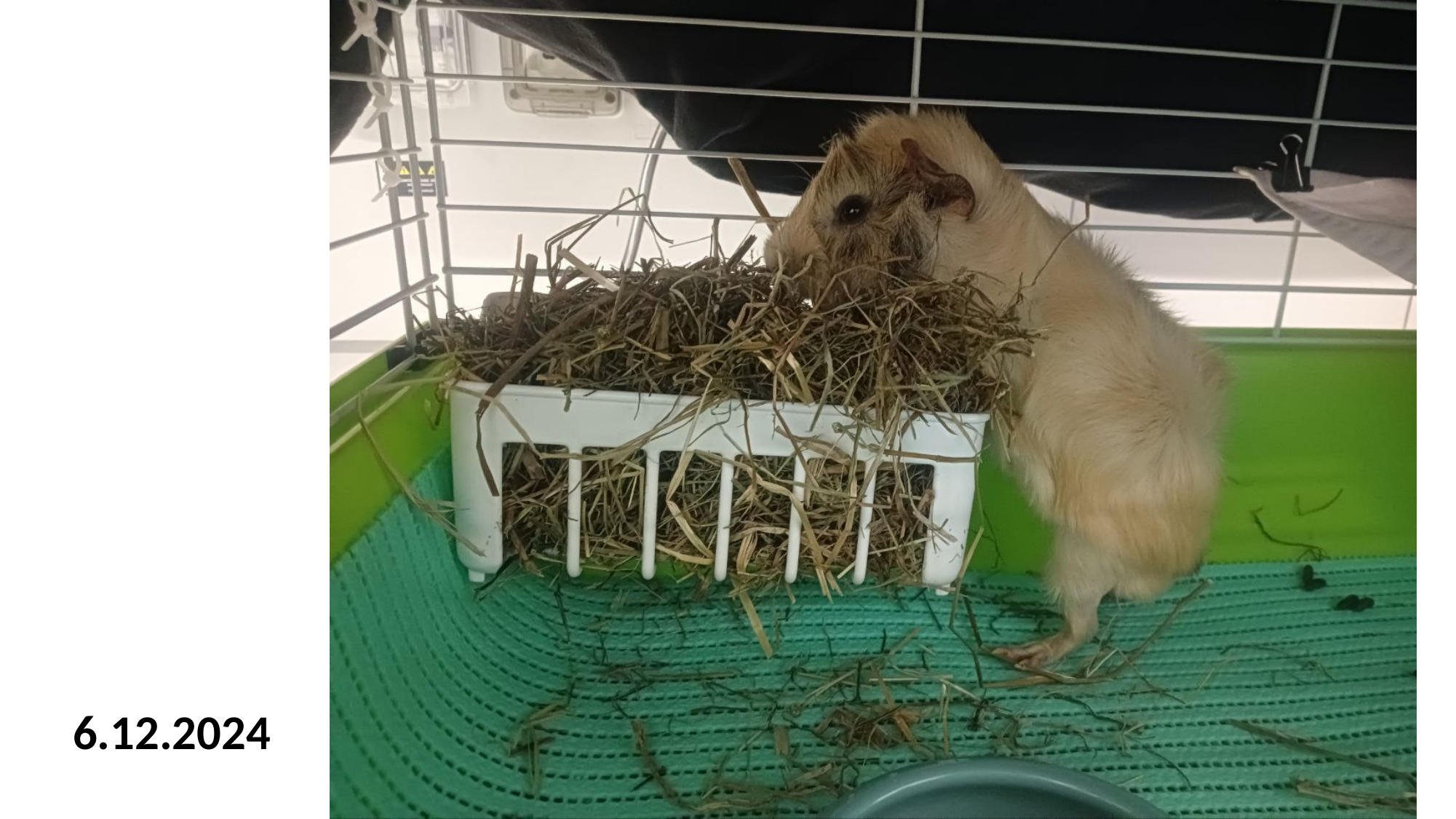

6.12.2024

## Slide 97
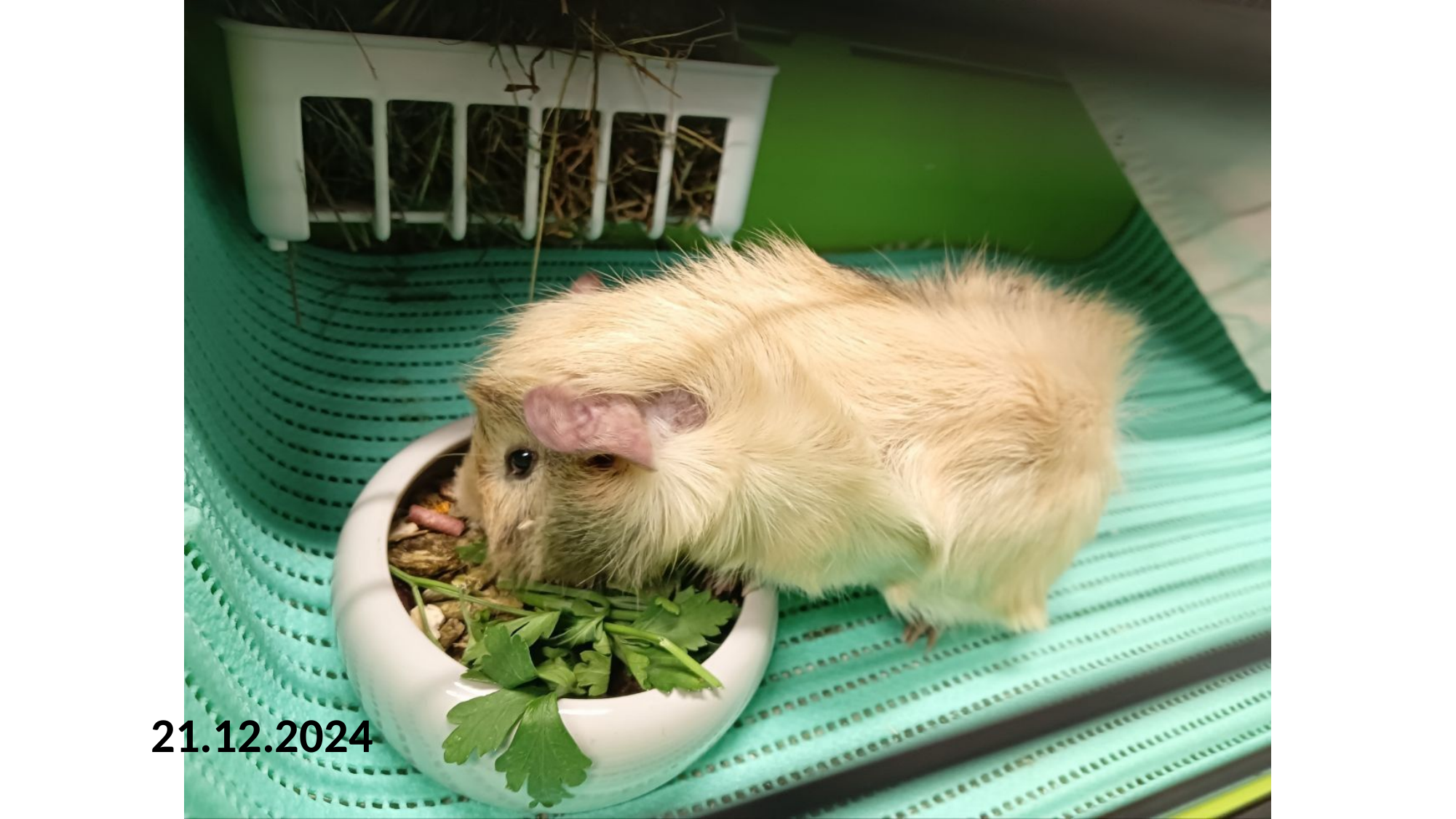

21.12.2024

## Slide 98
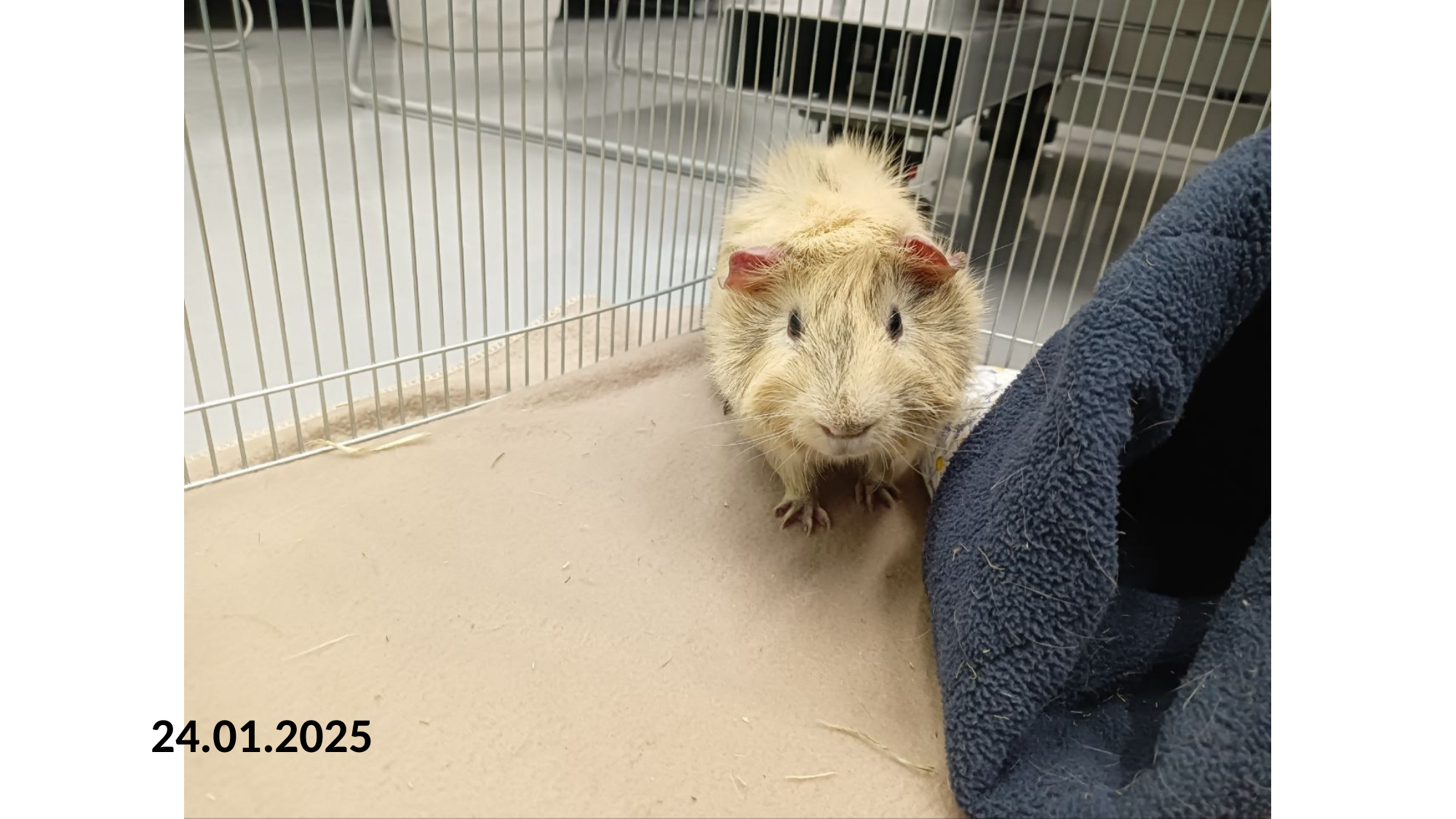

24.01.2025

## Slide 99
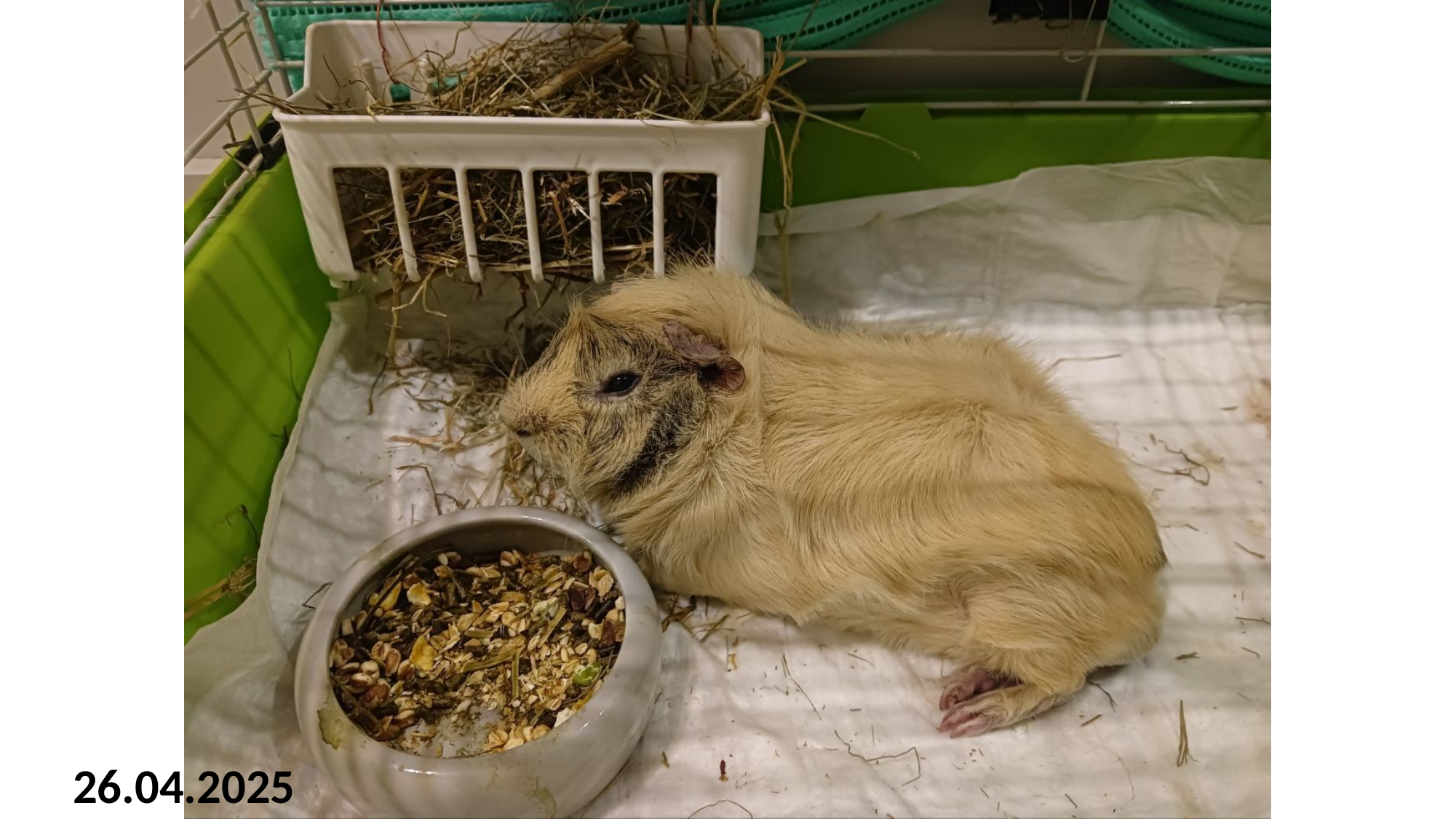

26.04.2025

## Slide 100
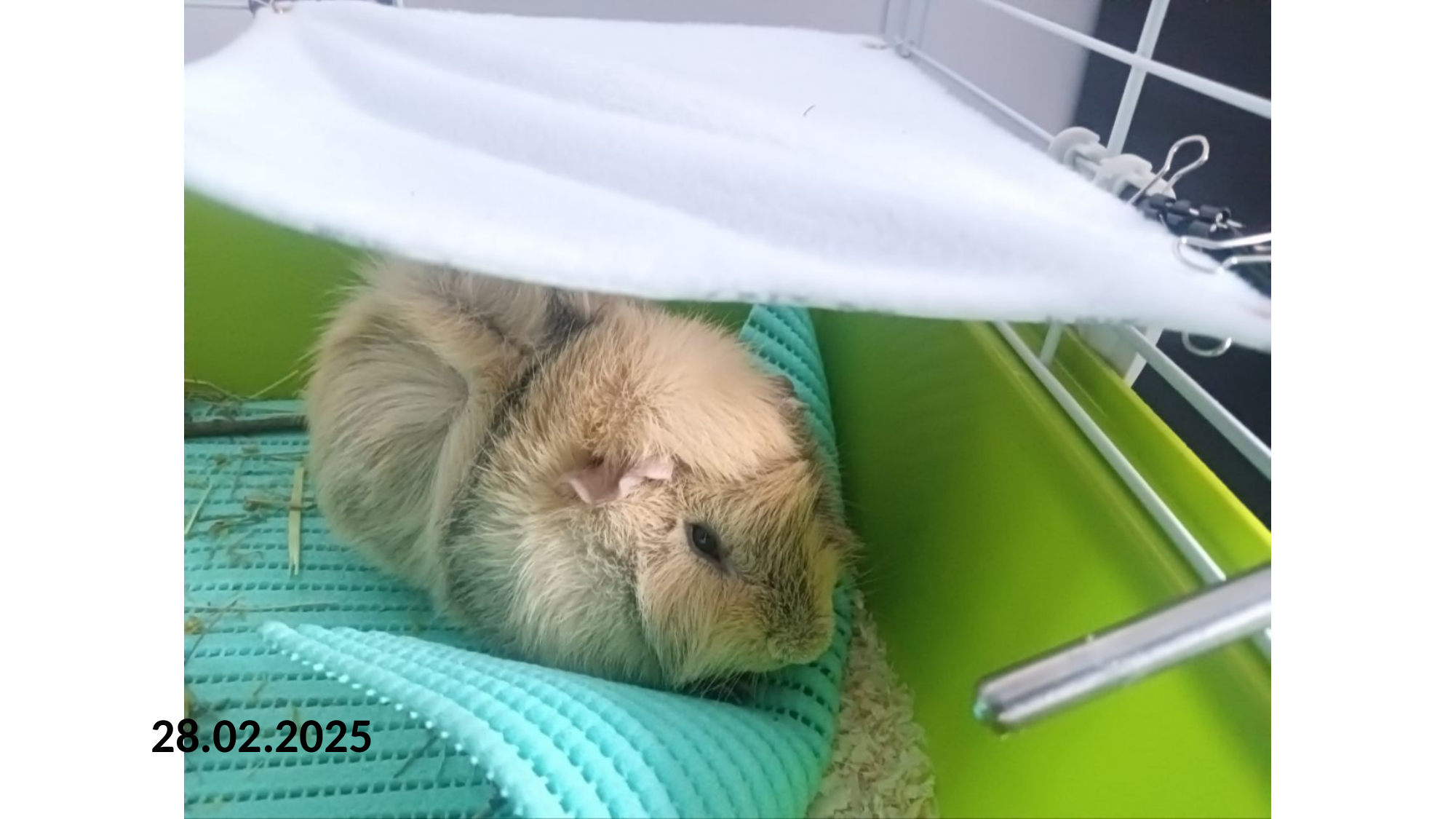

28.02.2025

## Slide 101
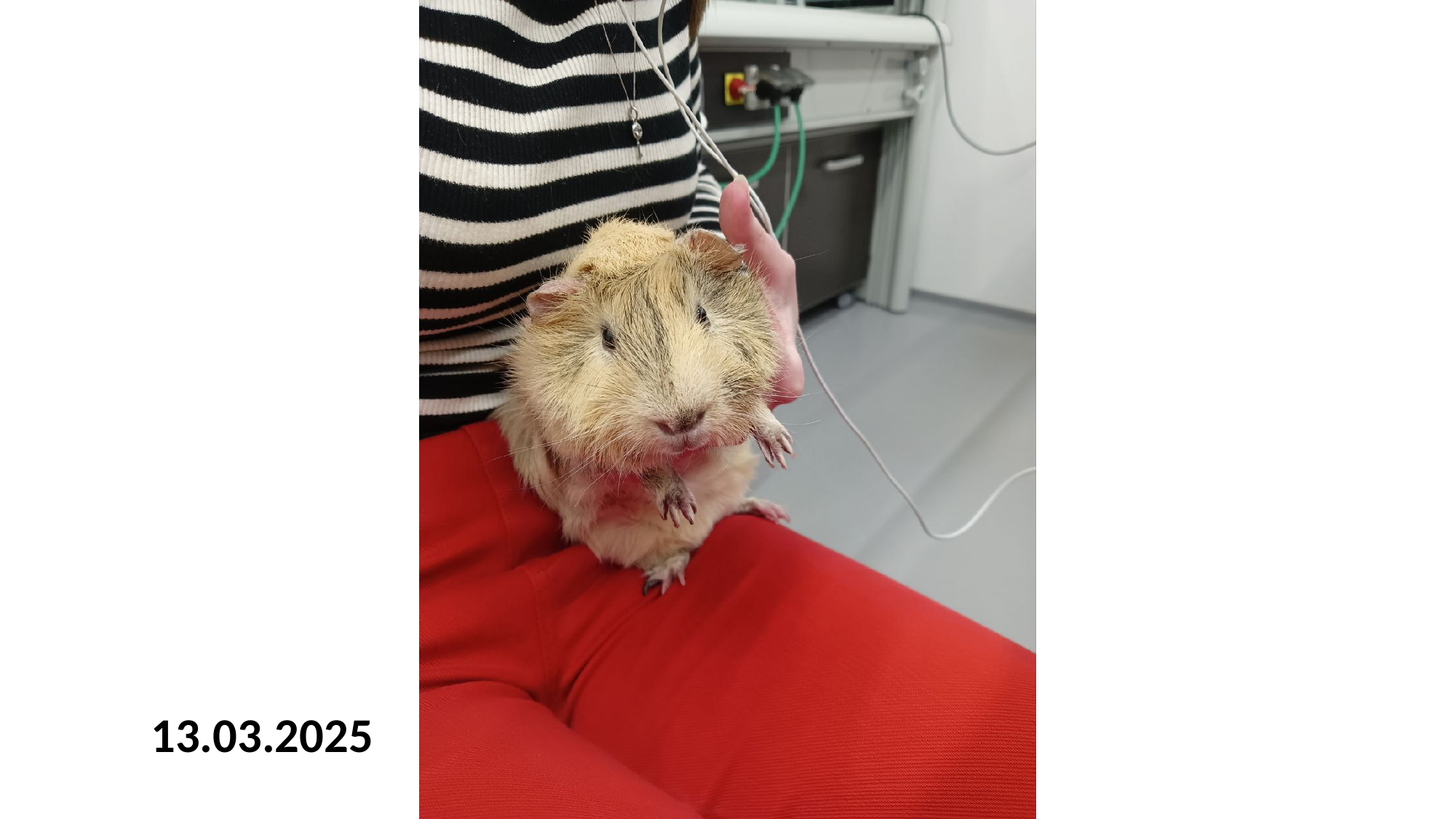

13.03.2025

## Slide 102
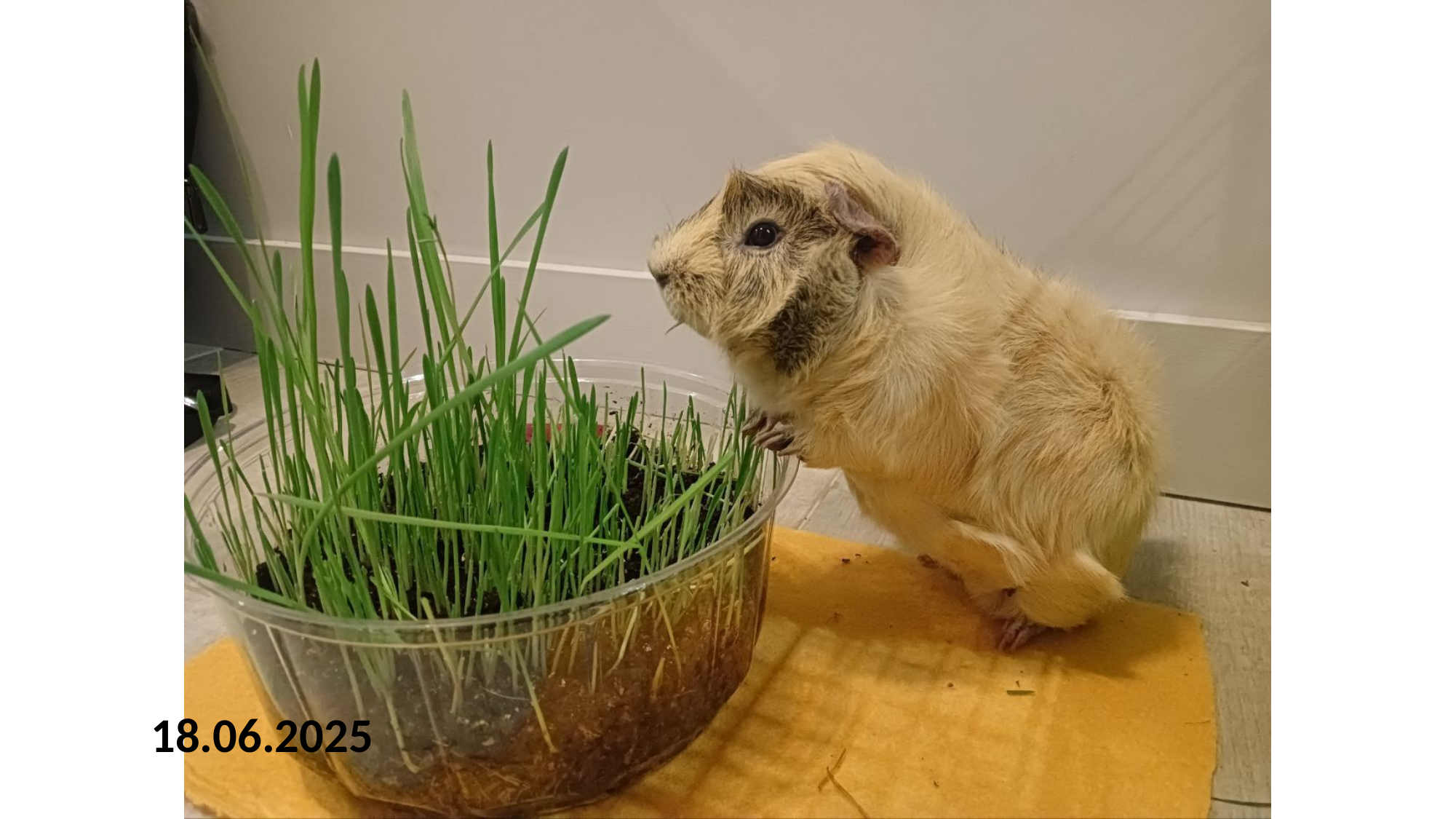

18.06.2025

## Slide 103
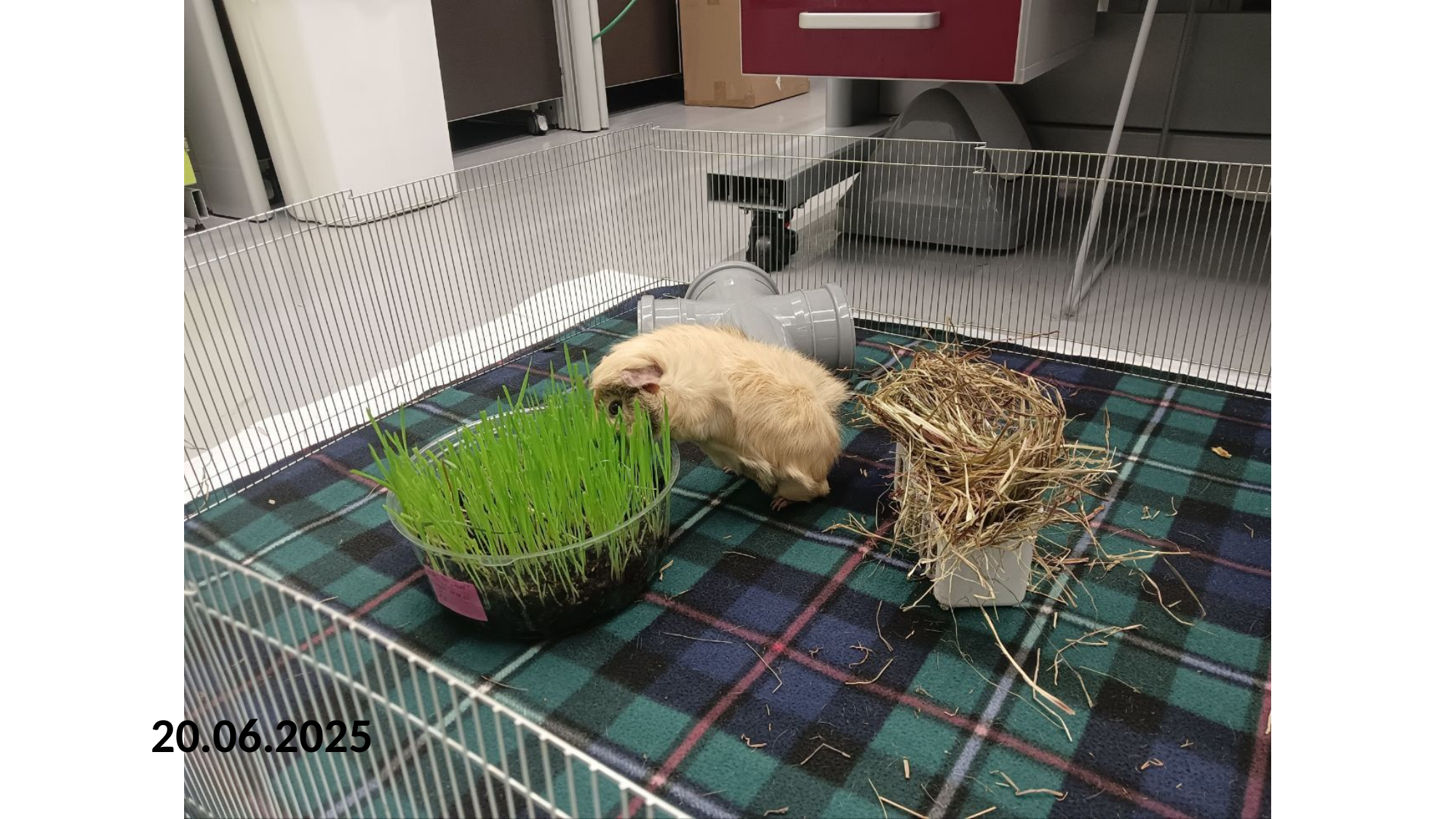

20.06.2025

## Slide 104
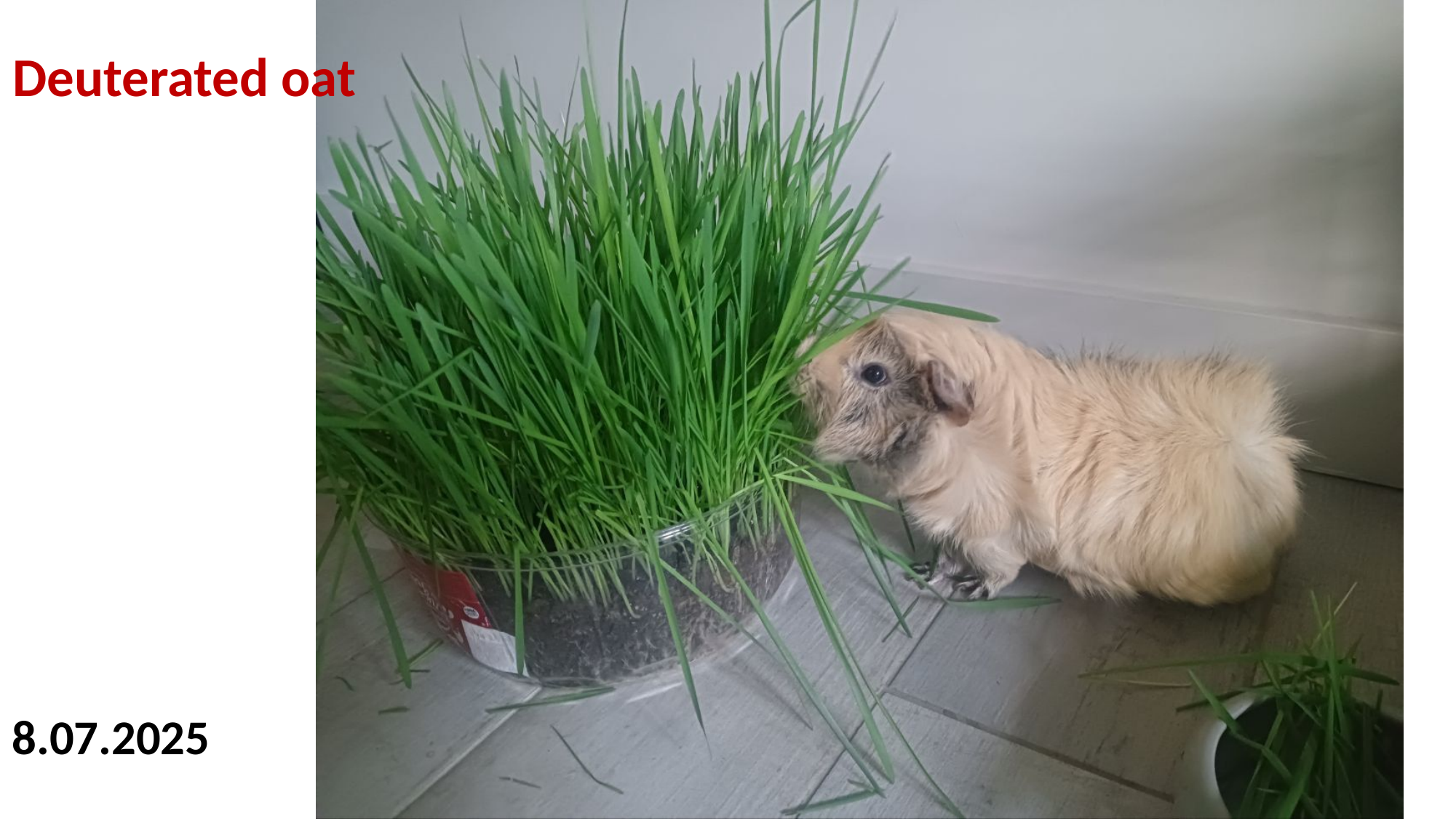

Deuterated oat
8.07.2025

## Slide 105
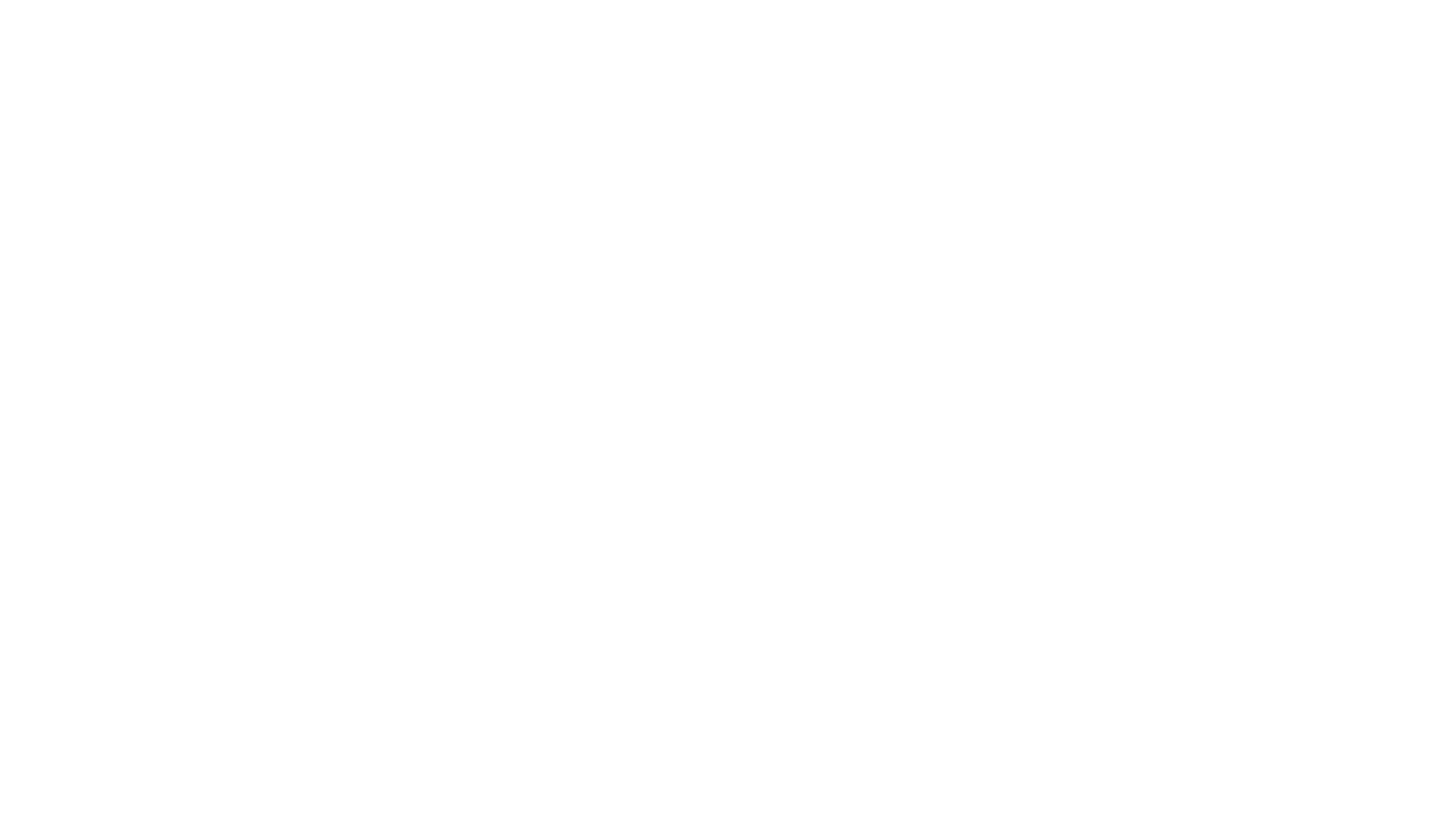

## Slide 106
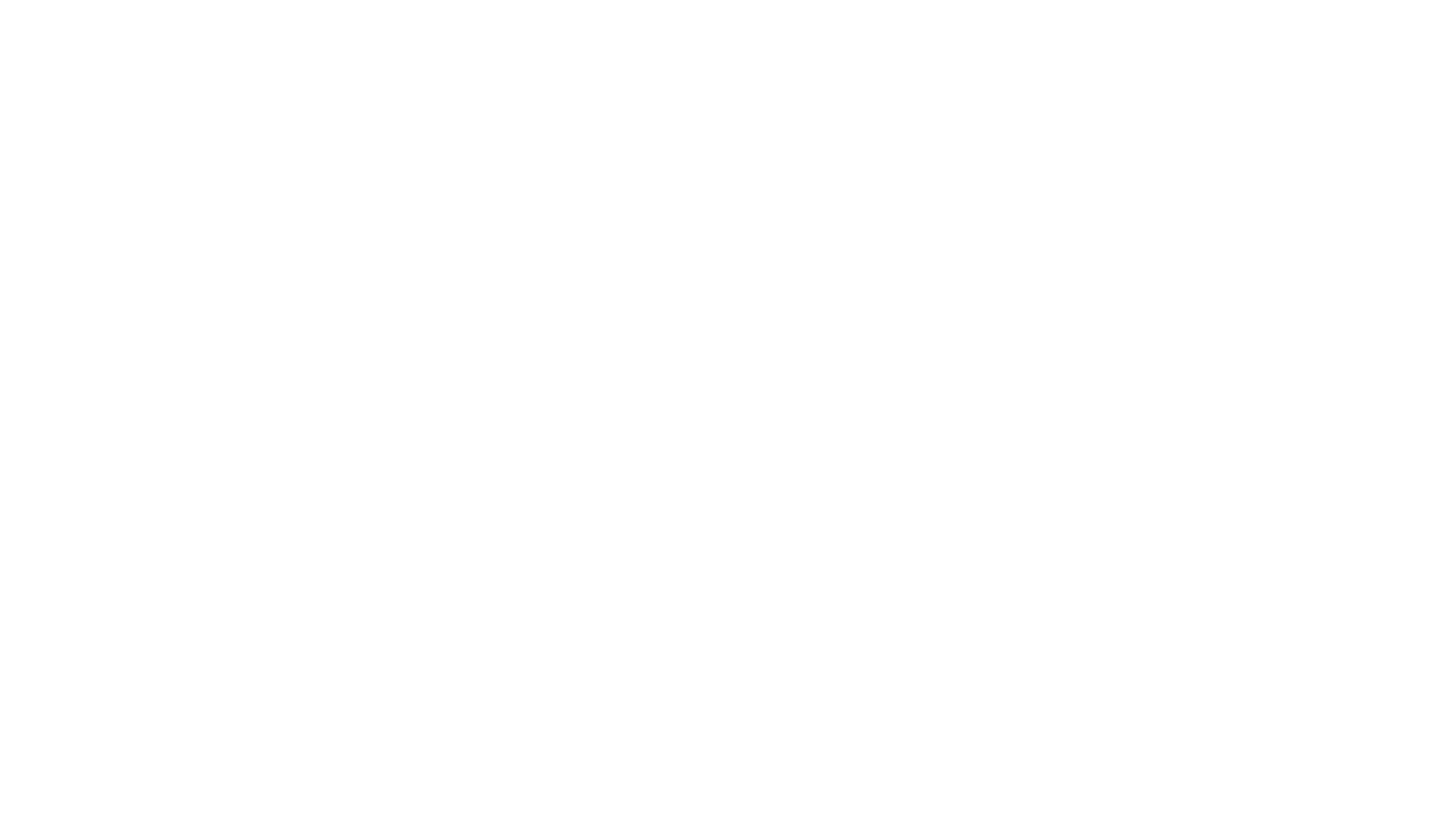

Supplement: Supplementary file 1 [file ijms-27-01944-s001.zip › !!! Figures for paper SUPPORTING INFO v1.pptx]
